# Sequence information transfer using covalent template-directed synthesis†
†Electronic supplementary information (ESI) available: Materials and methods, synthetic procedures, full characterization of all compounds, UPLC traces for full replication cycle and molecular modelling are available. See DOI: 10.1039/c9sc01460h


**DOI:** 10.1039/c9sc01460h

**Published:** 2019-04-26

**Authors:** Diego Núñez-Villanueva, Maria Ciaccia, Giulia Iadevaia, Elena Sanna, Christopher A. Hunter

**Affiliations:** a Department of Chemistry , University of Cambridge , Lensfield Road , Cambridge , CB2 1EW , UK . Email: herchelsmith.orgchem@ch.cam.ac.uk

## Abstract

Template-directed synthesis is the biological method for the assembly of oligomers of defined sequence, providing the molecular basis for replication and the process of evolution. To apply analogous processes to synthetic oligomeric molecules, methods are required for the transfer of sequence information from a template to a daughter strand. We show that covalent template-directed synthesis is a promising approach for the molecular replication of sequence information in synthetic oligomers. Two monomer building blocks were synthesized: a phenol monomer and a benzoic acid monomer, each bearing an alkyne and an azide for oligomerization *via* copper catalyzed azide alkyne cycloaddition (CuAAC) reactions. Stepwise synthesis was used to prepare oligomers, where information was encoded as the sequence of phenol (**P**) and benzoic acid (**A**) units. Ester base-pairing was used to attach monomers to a mixed sequence template, and CuAAC was used to zip up the backbone. Hydrolysis of the ester base-pairs gave back the starting template and the sequence complementary copy. When the **AAP** trimer was used as the template, the complementary sequence **PPA** was obtained as the major product, with a small amount of scrambling resulting in **PAP** as a side-product. This covalent base-pairing strategy represents a general approach that can be implemented in different formats for the replication of sequence information in synthetic oligomers.

## Abstract 2

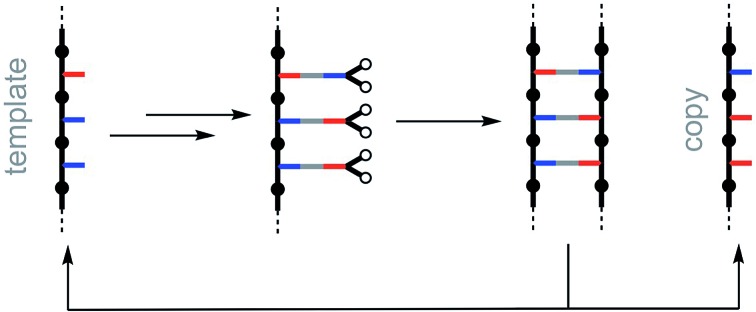
Kinetically inert ester bonds were used to attach monomers to a template, dictating the sequence of the polymer product.

## Introduction

Template-directed synthesis is the method used in biology for production of oligomers with defined sequence. The information encoded in DNA templates is copied into DNA duplicates, RNA transcripts and translated proteins, and these information transfer processes are the molecular basis for the evolution of living systems.^1^–^3^ The development of template-directed methods for synthesising non-natural oligomers of defined sequence would open the way for exploitation of directed evolution to identify synthetic oligomer sequences with useful properties, Orgel's Holy Grail.^4^ Nucleic acids are the only materials currently capable of sequence information transfer, so the use of evolution to explore chemical space is limited to systems that interface with DNA.^5^–^8^ Here we describe a template-directed approach to synthetic oligomers that uses covalent base-pairing as the basis for sequence information transfer between parent and daughter strands. This chemical templating process represents a first step towards the application of evolution to tackle the vast chemical space constituted by synthetic oligomer sequences.

**Fig. 1:**
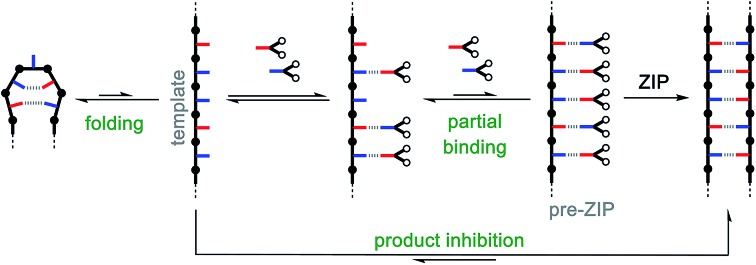
Non-covalent template-directed synthesis of mixed sequence oligomers. The red and blue bars represent two different bases that form a base-pair *via* non-covalent interactions. Reversible binding of monomer building blocks to the template followed by a zipping up process covalently couples adjacent monomers to yield a duplex with a product oligomer that is a complementary copy of the template. Competing equilibria that interfere with formation of the key pre-ZIP complex are highlighted in green.

The information transfer that takes place on nucleic acid templates is based on selective H-bonding interactions that hold the correct monomer unit in place for attachment to a growing oligomer chain. Non-covalent templating is well-established in the field of supramolecular chemistry and has been widely applied in the synthesis of macrocycles^9^–^12^ and mechanically interlocked molecules.^13^–^18^ Although non-covalent templates have been used for homo-oligomerisation reactions,^19^–^24^ template-directed synthesis of mixed sequence oligomers remains exclusively the domain of nucleic acids.^25^–^27^ Some of the reasons are outlined in Fig. 1. Ideally, the monomers would bind to the template, and then covalent bond formation in the **ZIP** step would give the complementary sequence as the product. However, when the base-pairing interactions are reversible, there are additional equilibria that compete with assembly of the key pre-ZIP intermediate: incomplete binding of monomers to the template, intramolecular folding of the template, and formation of a stable duplex that limits the availability of template due to product inhibition. Non-enzymatic experiments using nucleic acid templates show that even using DNA, it is difficult to achieve the process shown in Fig. 1.^28^–^30^ In biology, all of the competing equilibria are prevented by complex enzymatic machinery that controls each step of the process to attach the correct monomers one by one.^31^–^33^

## Approach

**Fig. 2:**
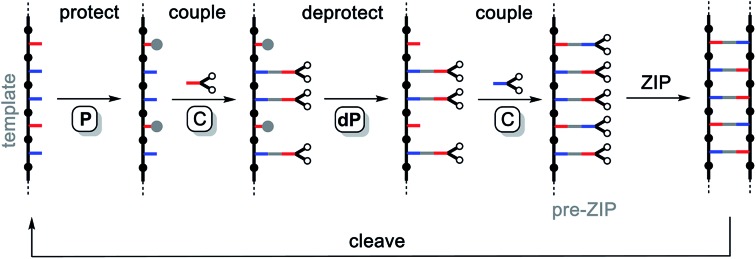
Covalent template-directed synthesis of mixed sequence oligomers. The red and blue bars represent bases that pair *via* a kinetically inert covalent bond. The pre-ZIP intermediate is assembled using a sequence of irreversible chemical reactions. Selective protection of the red bases (with grey balls, **P**), followed by covalent coupling of the blue bases with red monomers (**C**), deprotection of the red bases (**dP**), and covalent coupling of the red bases with blue monomers (**C**) yields the key intermediate for the **ZIP** step with no competing processes. The product oligomer and starting template are recovered by cleaving the covalent bonds connecting the base-pairs in the duplex.

Fig. 2 illustrates an alternative chemical approach to assembly of the pre-ZIP intermediate that avoids the competing equilibria shown in Fig. 1. In this case, the monomers are covalently attached to the template using kinetically stable bonds that can be cleaved again after the **ZIP** step, regenerating the starting template along with the templated product. The key to the success of the **ZIP** step in Fig. 2 is that the reactions on the template are intramolecular and hence more favourable than competing intermolecular reactions, which can be suppressed by operating at high dilution. Covalent template-directed synthesis is relatively unexplored compared with non-covalent approaches. There are some reports on the synthesis of macrocycles and mechanically interlocked molecules by covalent templating.^34^–^41^ A cored dendrimer has been prepared by using a porphyrin as a covalent template for cross-linking the external arms of the dendrimer.^42^,^43^ Covalent templating has also been used for the synthesis of polydisperse homo-polymers suggesting that the key **ZIP** step in Fig. 2 is applicable to the synthesis of macromolecules.^44^–^46^ However, implementation of the scheme in Fig. 2 requires efficient orthogonal chemistry for selective attachment of two different types of monomer, zipping up the backbone, and cleavage of the product duplex. Here we describe one promising solution and demonstrate the utility by using a mixed sequence trimer as a template for synthesis of the complementary sequence.

## Results

### Covalent base-pairing chemistry

**Fig. 3:**
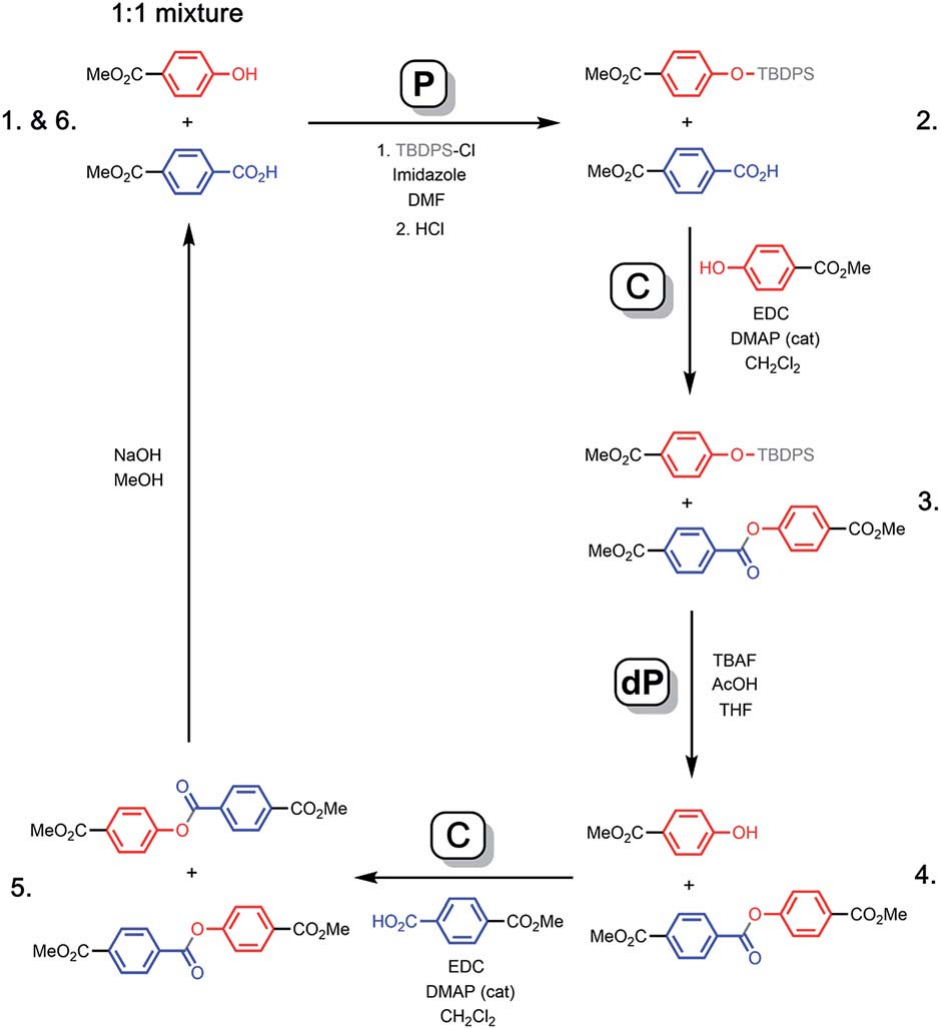
Formation of an ester between a phenol (red) and benzoic acid (blue) gives a covalent base-pair. Starting from a 1 : 1 mixture of two template bases, sequential formation of two base-pairs *via* the **P**–**C**–**dP**–**C** sequence is illustrated. Cleavage by hydrolysis gives the two template bases and two product bases, which are identical to the original template bases. The 1–6 numbering corresponds to the ^1^H NMR spectra shown in Fig. 4.

Fig. 3 shows how a covalent base-pairing system can be implemented based on formation of an ester between a phenol (red base) and a benzoic acid (blue base). The base-pair attachment sequence shown in Fig. 2 can be achieved by selective protection of the phenol bases (**P**), ester coupling of the benzoic acid bases with phenol monomers (**C**), deprotection of the phenol bases (**dP**) and coupling of the phenol bases with benzoic acid monomers (**C**). Hydrolysis of the esters recovers the starting template and a complementary copy.

**Fig. 4:**
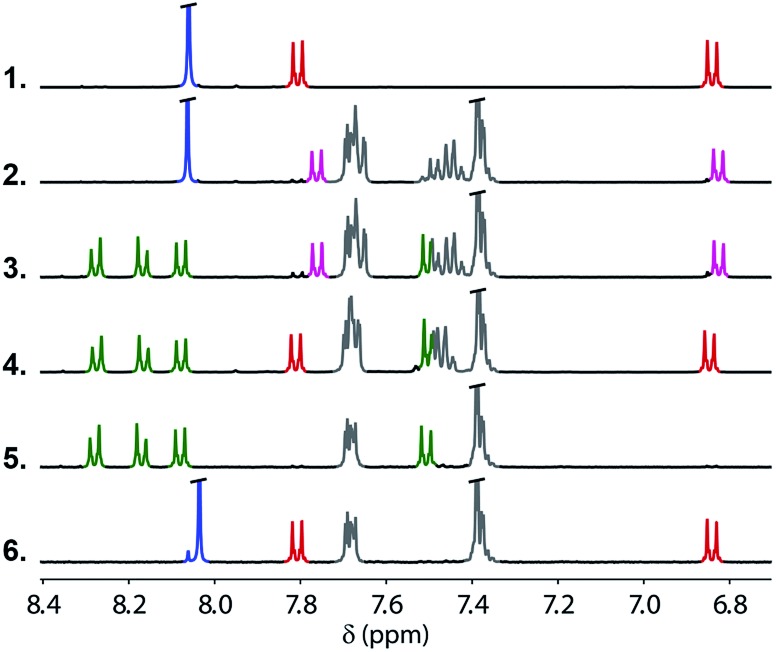
Partial 400 MHz ^1^H NMR spectra in DMSO-*d*_6_ at 298 K of the crude reaction mixtures for the ester base-pair attachment and cleavage cycle shown in Fig. 1. Spectrum 1: the starting template phenol (red) and benzoic acid (blue). Spectrum 2: selective protection of the phenol (pink) in the presence of the benzoic acid (blue). Spectrum 3: formation of the first base-pair (green) in the presence of the protected phenol (pink). Spectrum 4: deprotection of the phenol (red) in the presence of the first base-pair (green). Spectrum 5: formation of the second base-pair (green). Spectrum 6: products of the cleavage reaction, the phenol (red) and benzoic acid (blue). There is a small blue signal due to partial hydrolysis of the spectator methyl esters, and the grey signals are TBDPS residues not removed by aqueous work-up.

In order to demonstrate the viability of this covalent base-pair attachment and cleavage chemistry, we carried out the complete reaction cycle on the model system shown in Fig. 3. In this case, the two different bases that represent the template (a phenol and a benzoic acid) are present as a mixture of two separate molecules rather than connected as part of an oligomer. Fig. 4 shows ^1^H NMR spectra of the crude reaction mixtures obtained from the sequence of reactions in Fig. 3. Selective formation and then cleavage of the esters of both bases were achieved quantitatively and the only purification required between steps was an aqueous work-up. Ester base-pairing chemistry therefore appears to be an ideal candidate for the development of robust quantitative covalent template-directed synthesis methodologies.

### Backbone chemistry

**Fig. 5:**
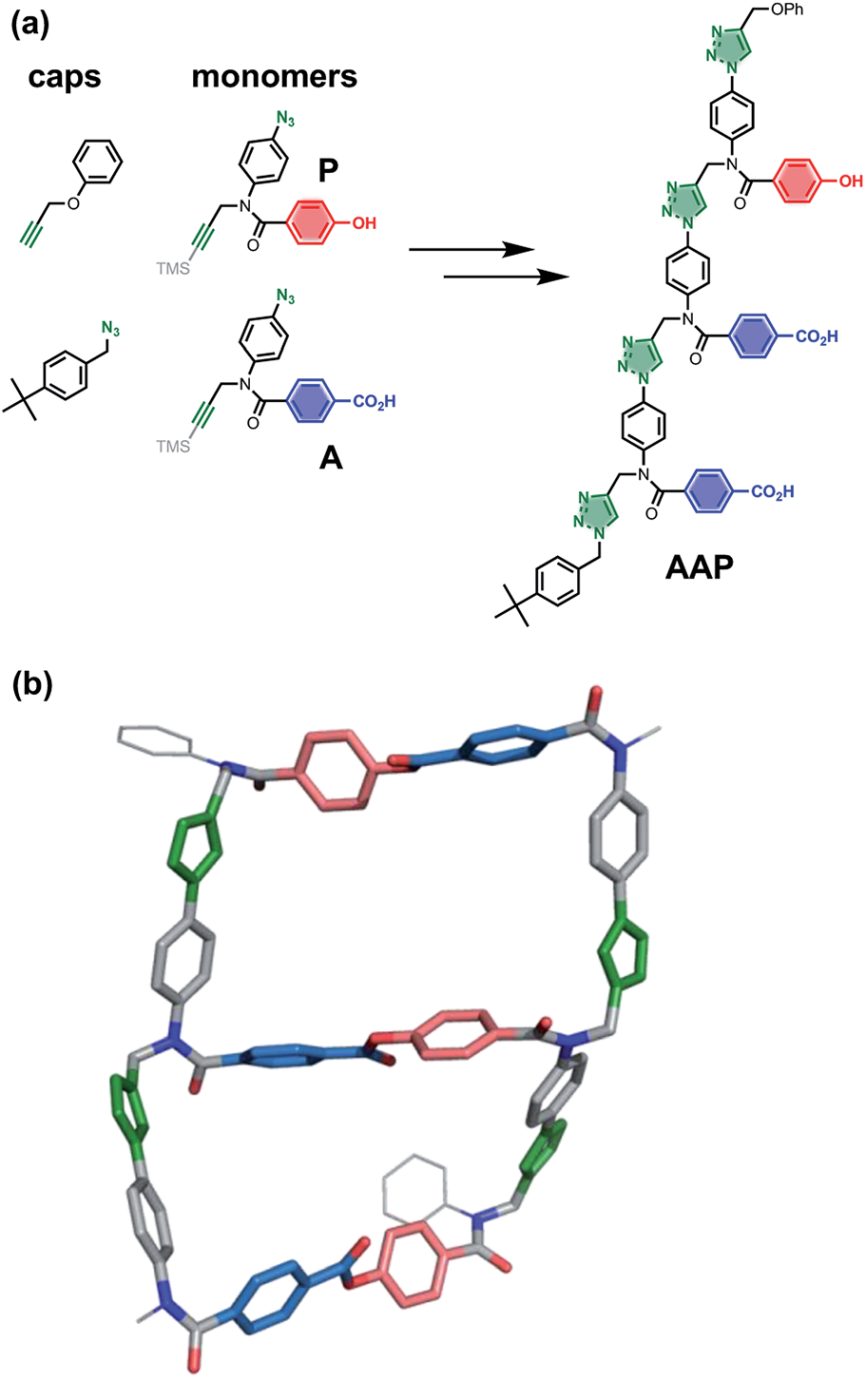
(a) Monomer building blocks (**A** and **P**) and monofunctional caps for assembly of mixed sequence oligomers by sequential CuAAC coupling and TMS deprotection reactions: the **AAP** trimer is shown. (b) Molecular mechanics structure of the antiparallel **AAP·APP** duplex (lowest energy structure from conformational searches).

The copper catalysed azide–alkyne cycloaddition (CuAAC) reaction is high yielding and compatible with a wide range of chemical functionality,^47^,^48^ so this reaction was selected for the **ZIP** step in Fig. 2. Fig. 5(a) shows the structures of monomer building blocks, equipped with an azide, an alkyne and either a phenol (**P**) or a benzoic acid (**A**), and a trimer template (**AAP**) that can be assembled from these monomers through sequential CuAAC and TMS deprotection reactions. The product of the **ZIP** step in Fig. 2 is a duplex, and it is therefore essential that the conformational properties of the backbone formed in CuAAC oligomerisation reactions are compatible with this polymacrocyclic architecture. The molecular mechanics model of the **AAP·APP** duplex shown in Fig. 5(b) indicates that the ring strain is low: 18–35 kJ mol^–1^ per macrocyclisation, which is comparable to cyclopentane (26 kJ mol^–1^).^49^,^50^ The CuAAC backbone shown in Fig. 5 is directional (we write the sequence in the alkyne to azide direction), so two isomeric duplexes can be formed in the **ZIP** step, parallel and antiparallel. Fig. 5(b) shows the antiparallel **AAP·APP** duplex, which was calculated to be 5 kJ mol^–1^ more stable than the corresponding parallel duplex, so selectivity in the **ZIP** step can be anticipated (see ESI† for details).

### Synthesis of the monomers

**Scheme 1:**
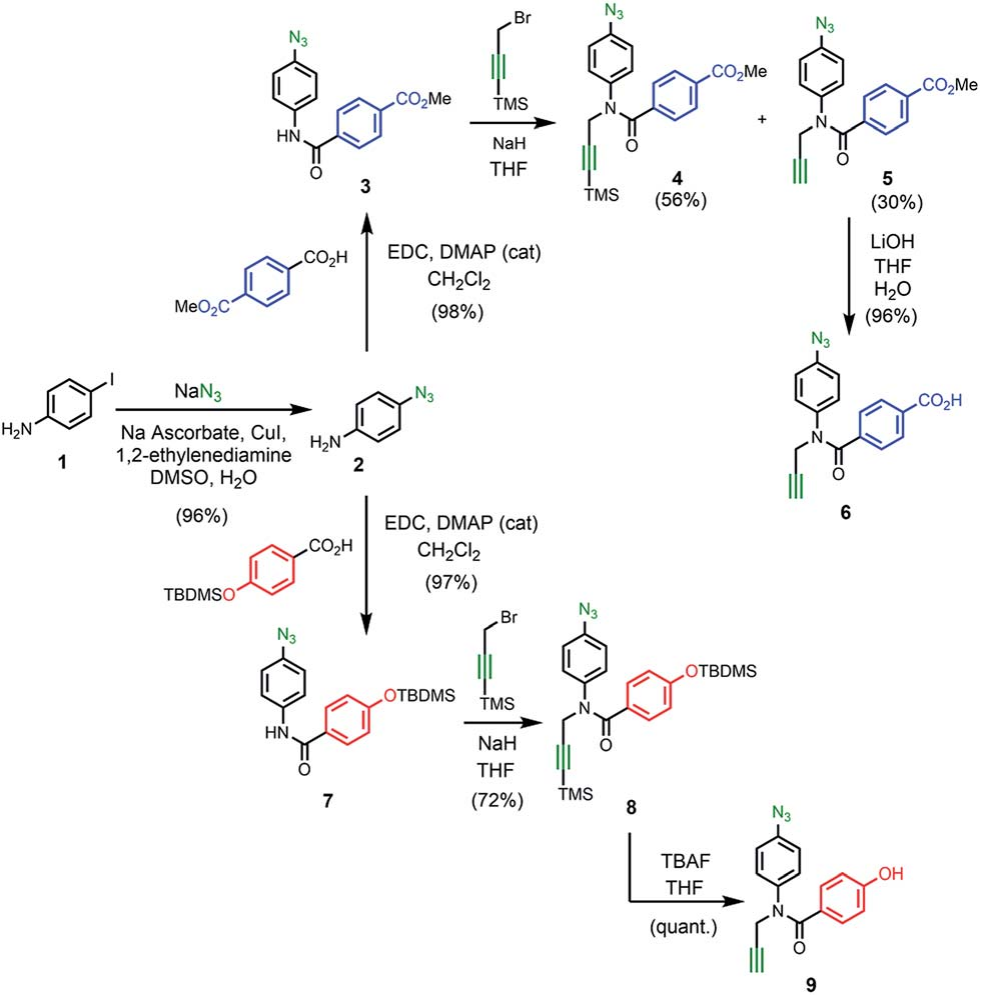


Scheme 1 shows the synthesis of the two monomer building blocks. *p*-Azidoaniline **2** was prepared according to a procedure described in the literature,^51^ and subsequent amide coupling with mono-methyl terephthalate gave **3** in excellent yield. Alkylation of **3** using TMS-protected propargyl bromide and sodium hydride gave a mixture of **4** and the TMS-deprotected alkyne **5**. TBAF deprotection of **4** and hydrolysis of **5** using lithium hydroxide afforded the benzoic acid monomer **6** in excellent yield. For the phenol monomer, aniline **2** was coupled with TBDMS-protected 4-hydroxybenzoic acid to give the amide **7** in excellent yield. Subsequent alkylation with TMS-protected propargyl bromide provided **8**, and TBAF-mediated deprotection of both silyl protecting groups gave phenol monomer **9** in excellent yield.

### Synthesis of the template

**Scheme 2:**
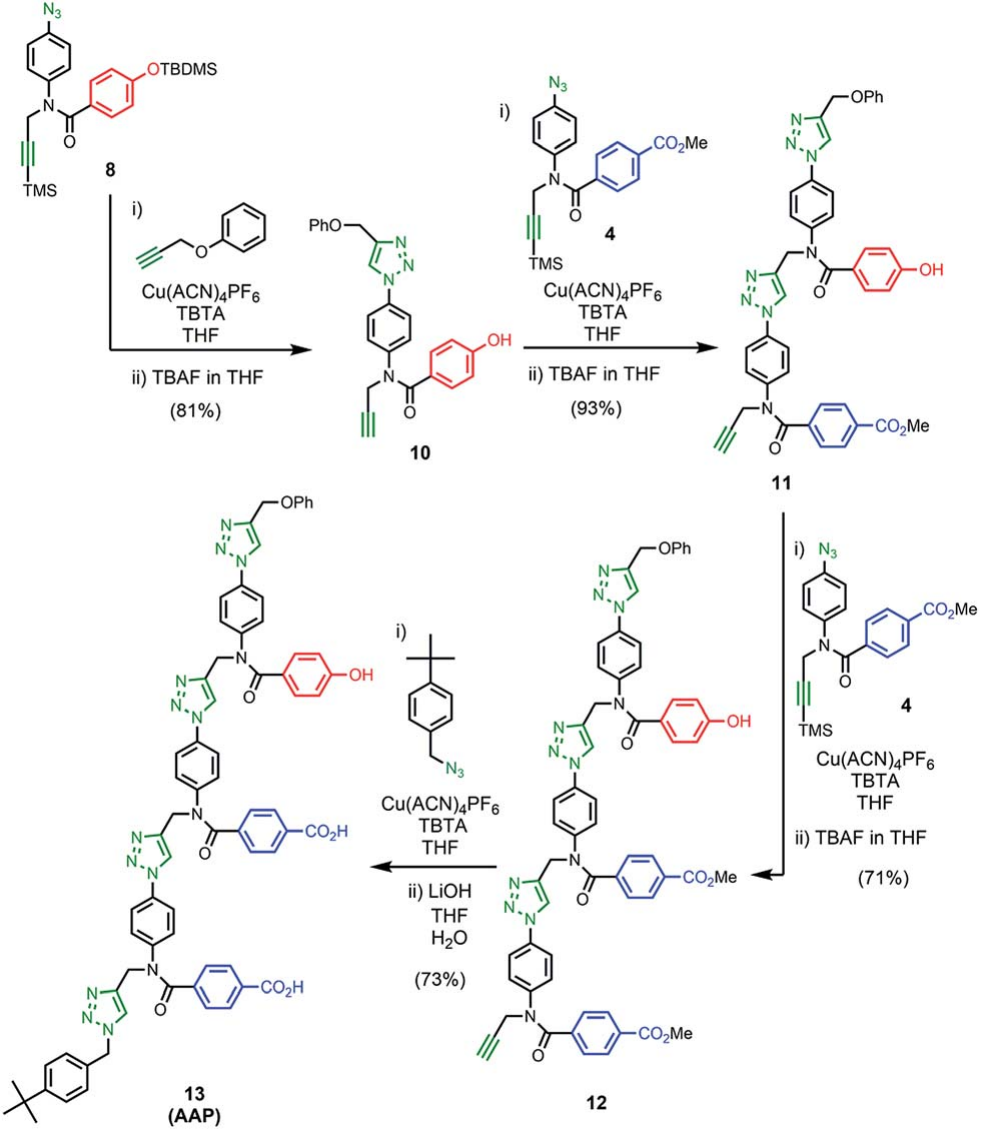


The two monomer building blocks were used to prepare a template oligomer through sequential CuAAC and TMS deprotection reactions. Scheme 2 shows the synthesis of a mixed sequence trimer. In order to prevent reaction with the ends of the template chain in the **ZIP** step, monofunctional chain terminating groups (phenyl propargyl ether and *p-tert*-butylbenzylazide) were used to cap the terminal azide and alkyne. The protected phenol monomer **8** was first coupled with phenyl propargyl ether using a CuAAC reaction. TBAF-mediated deprotection of the product gave the capped phenol monomer **10** in good yield. CuAAC coupling of **10** with **4** gave access to mixed 2-mer **11** in excellent yield. CuAAC coupling of **11** with **4** afforded 3-mer **12**. CuAAC coupling of **12** with *p-tert*-butylbenzylazide followed by LiOH-mediated basic hydrolysis yielded template **13** in good yield. We will use the shorthand **AAP** for this acid–acid-phenol template, writing the sequence in the alkyne to azide direction starting at the *t*-butyl benzyl terminus.

### Oligomer synthesis on a trimer template

**Fig. 6:**
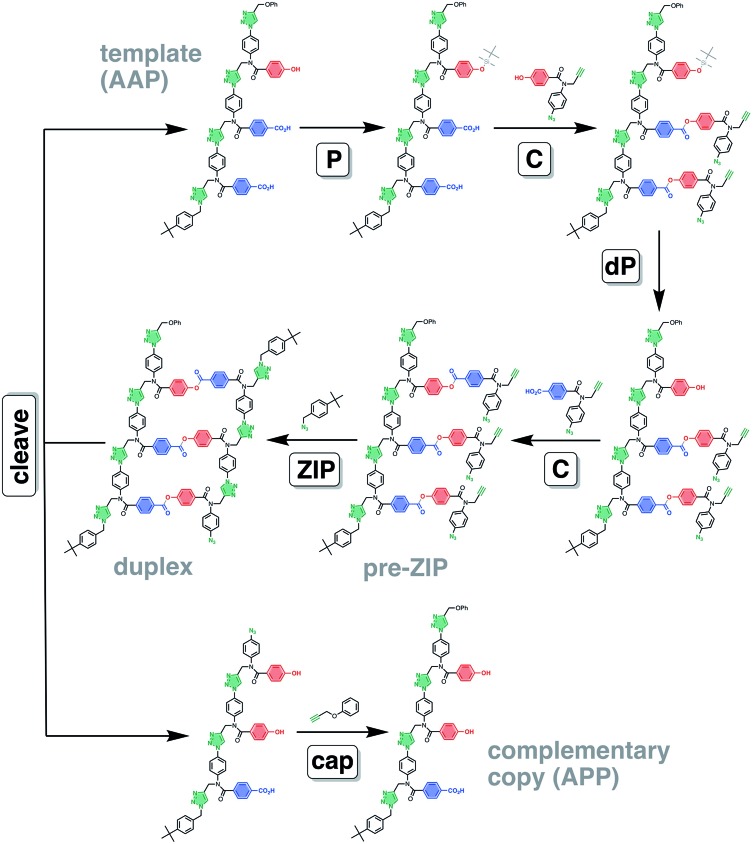
Covalent template-directed synthesis of a mixed sequence trimer. Ester base-pair chemistry was used to load two different monomer units onto the **AAP** template with the **P**–**C**–**dP**–**C** reaction sequence. The resulting pre-ZIP intermediate was subjected to a CuAAC reaction to give the duplex. Hydrolysis of the ester base-pairs in the **cleave** step regenerated the template, and capping of the terminal azide of the other product provided the complementary copy **APP**. The antiparallel duplex is shown as the product of the **ZIP** step, but the parallel duplex leading to **PPA** as the complementary copy is also possible. *Conditions*: **P** TBDMS-Cl, imidazole, then HCl; **C** EDC, DMAP; **dP** TBAF; **ZIP** Cu(CH_3_CN)_4_PF_6_, TBTA; **cleave** LiOH; **cap** Cu(CH_3_CN)_4_PF_6_, TBTA.

The **AAP** trimer was used as a template for the synthesis of a sequence-complementary copy using the ester base-pairing chemistry shown in Fig. 3 and CuAAC backbone chemistry for the **ZIP** step. The two different monomer units were loaded onto the template using the protection-coupling-deprotection-coupling reaction sequence shown in Fig. 6.

**Fig. 7:**
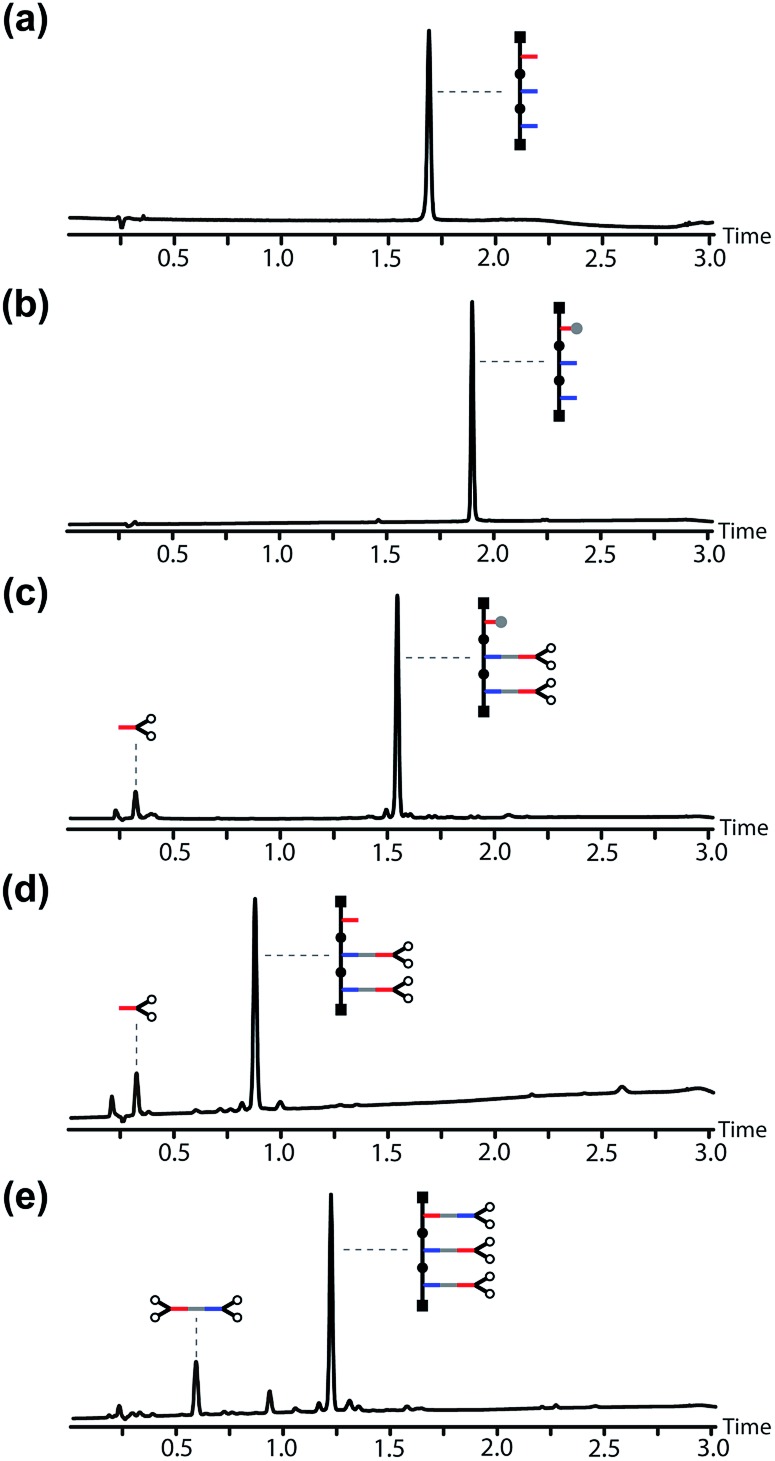
UPLC traces of crude reaction mixtures obtained after aqueous work-up in the base-pair attachment sequence using the **AAP** template: (a) the starting template, and the crude products obtained after (b) TBDMS protection, **P**, (c) EDC coupling with the phenol monomer, **C**, (d) TBAF deprotection, **dP**, (e) EDC coupling with the benzoic monomer, **C**. *Conditions*: C18 column at 40 °C using a gradient of water/formic acid (0.1%) and CH_3_CN/formic acid (0.1%). The gradients used in (a) and (b) were different from those used in (c–e).

**Fig. 8:**
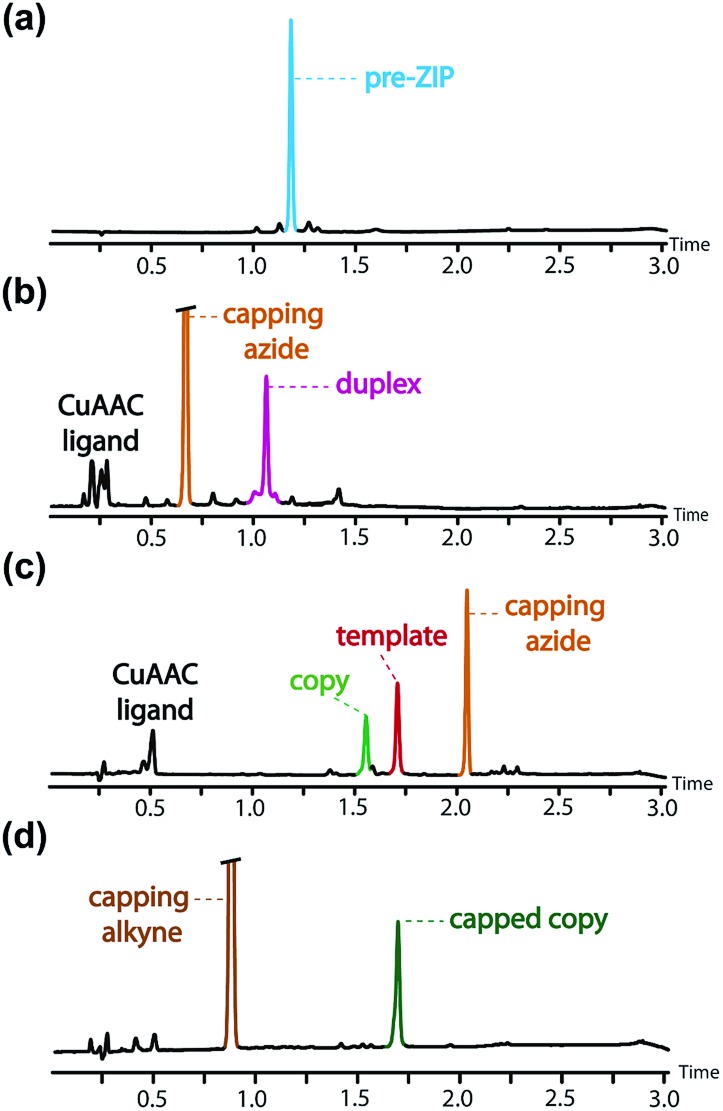
UPLC traces of crude reaction mixtures obtained after aqueous work-up in conversion of the pre-ZIP intermediate to the templated product: (a) the starting pre-ZIP intermediate and the crude products obtained after (b) the CuAAC **ZIP** step, (c) LiOH base-pair cleavage, (d) CuAAC capping of the copy strand. *Conditions*: C18 column at 40 °C using a gradient of water/formic acid (0.1%) and CH_3_CN/formic acid (0.1%). The capping azide appears at different retention times in (b) and (c), because the gradient used in (a) and (b) was different from that used in (c) and (d).

Fig. 7 shows the corresponding UPLC traces of the crude reaction mixtures obtained in each step of the base-pair attachment sequence. In each case, quantitative conversion of starting material to product was achieved with aqueous work-up as the only purification required. Fig. 7(a) and (b) show that the TBDMS protection step cleanly converted the template into the protected phenol. Fig. 7(c) shows the crude reaction mixture from the EDC coupling of the exposed benzoic acids on the template with an excess of the phenol monomer. Base-pair formation proceeded quantitatively, and the only other species present was the excess of the phenol monomer. The phenol group on the template was deprotected cleanly using TBAF, and the excess of phenol monomer used in the coupling step was the only impurity carried through in this step (Fig. 7(d)). The second base-pair coupling reaction to load the benzoic acid monomer onto the template gave quantitative conversion to the pre-ZIP intermediate. The only other species present in the crude reaction mixture shown in Fig. 7(e) is the ester formed between benzoic acid monomer and the excess of the phenol monomer carried through from the previous steps. This side product was easily removed by chromatography to obtain the pre-ZIP intermediate in high purity and 63% isolated yield (Fig. 8(a)).

The **ZIP** step to oligomerise the monomers on the template was carried out using copper(i) catalysis under dilute conditions to minimize competing intermolecular reactions. An azide chain terminator was also added to the reaction mixture to prevent oligomerisation of the product duplex through the unreacted terminal alkyne and azide groups. Fig. 8(b) shows the UPLC trace of the crude reaction mixture obtained after aqueous work-up, which apart from the reagents, contained a single major species with a molecular weight that corresponds to the capped **AAP·APP** duplex shown in Fig. 6. The crude reaction mixture was then subject to lithium hydroxide hydrolysis to **cleave** the ester base-pairs, which cleanly converted the duplex into the template and copy strands (Fig. 8(c)). The two products were separated by flash chromatography (see ESI†), and the terminal azide group in the copy strand was then capped with phenyl propargyl ether in a quantitative CuAAC reaction to give the final templated product (Fig. 8(d) shows the crude reaction mixture obtained from the final capping step). In summary, the complete reaction sequence shown in Fig. 6 can be achieved with very high efficiency and almost quantitative conversion in each of the seven steps.

### Sequencing the products of templated oligomerisation

**Fig. 9:**
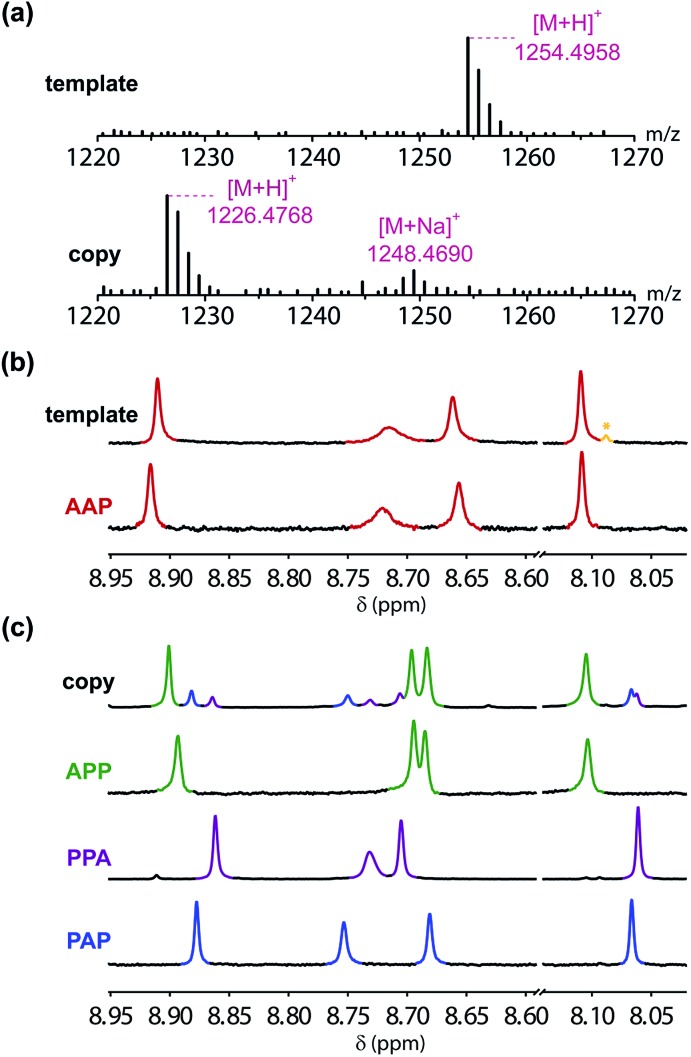
Sequencing of the products. (a) HRMS of the recovered template and the templated copy. (b) Triazole region of 500 MHz ^1^H NMR spectra in DMSO-*d*_6_ at 298 K of the recovered template (traces of the CuAAC ligand are marked in yellow with an asterisk) and the starting **AAP** template. (c) Triazole region of 500 MHz ^1^H NMR spectra in DMSO-*d*_6_ at 298 K of the templated copy and samples of all possible sequences containing one **A** and two **P** units, which were each synthesized independently.

In order to demonstrate successful sequence information transfer in the covalent template-directed synthesis cycle shown in Fig. 6, a method for sequencing the products is required. The MS–MS methods used in peptide sequencing failed for these triazole oligomers, which did not fragment cleanly.^52^,^53^ Sequencing was achieved using a combination of mass spectrometry, NMR spectroscopy and synthesis. The mass spectra shown in Fig. 9(a) are consistent with recovery of the **AAP** template strand and the sequence-complementary copy **APP** shown in Fig. 6 as the only products. The two bases have different molecular weights, so the presence of sequences containing different numbers of **A** and **P** units can be ruled out. The ^1^H NMR spectrum of the recovered template is identical to the spectrum of the starting material, apart from traces of the CuAAC ligand, which were not removed in the purification step (Fig. 9(b)). The template strand is therefore stable to the conditions of the templating cycle and can be recovered to be re-used in subsequent cycles.

**Scheme 3:**
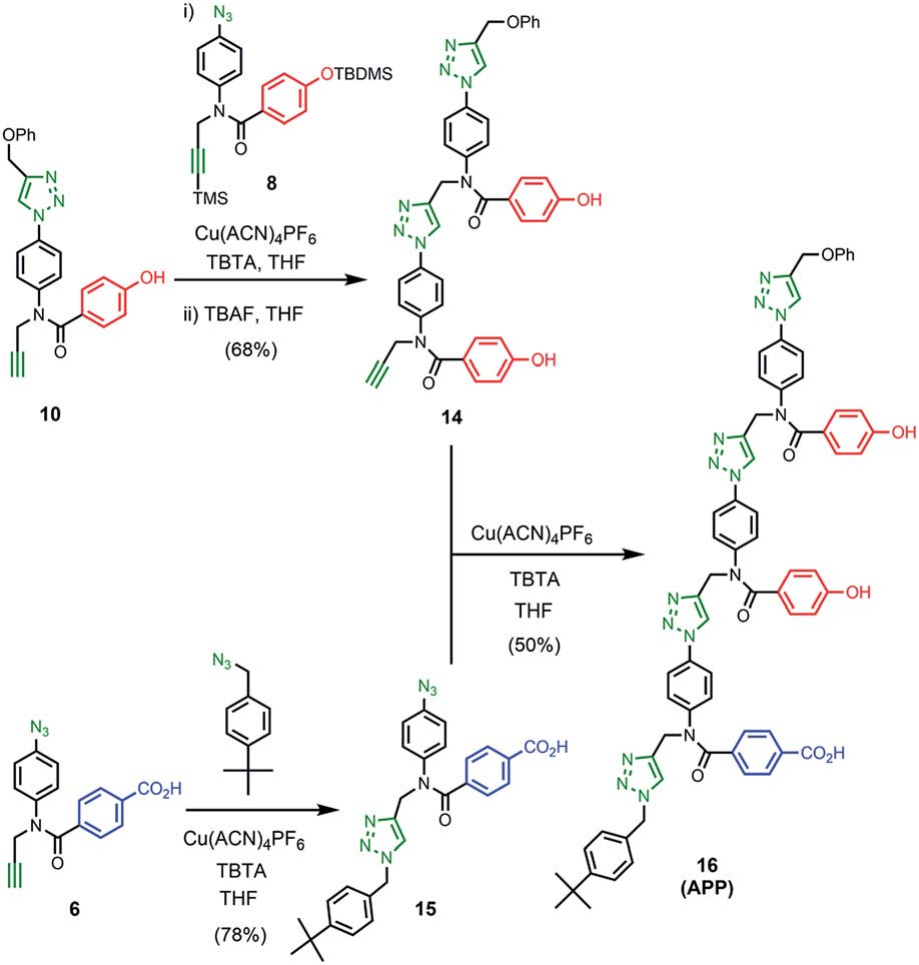


**Scheme 4:**
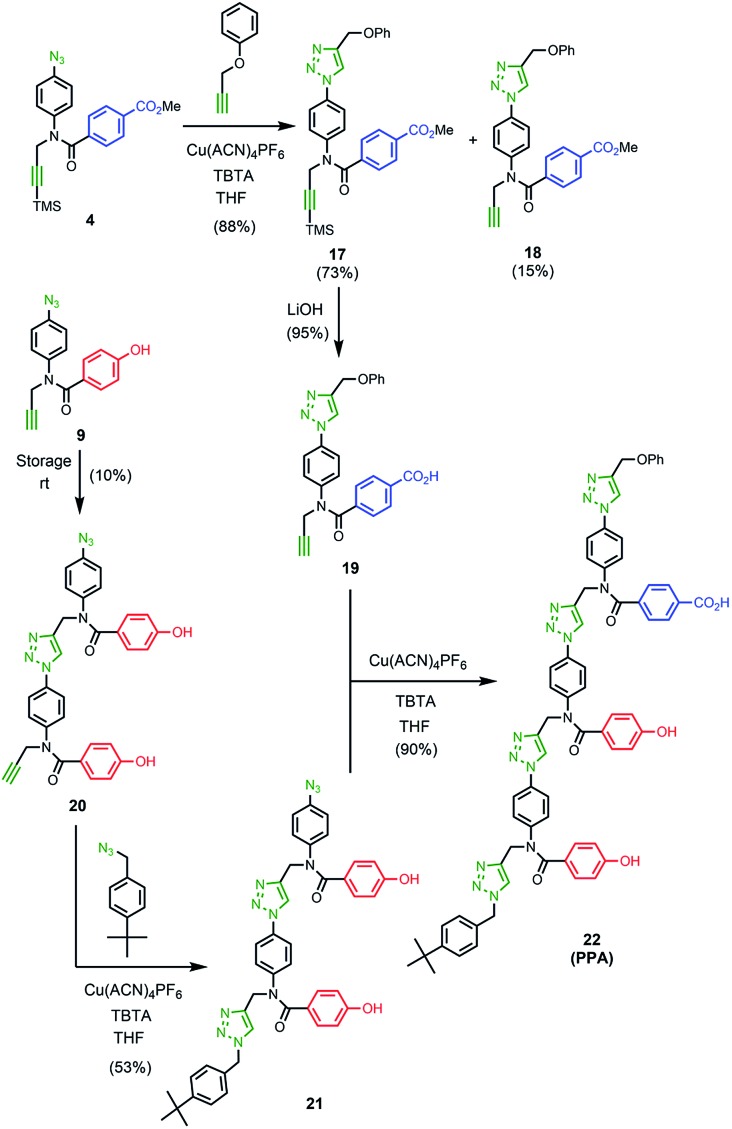


**Scheme 5:**
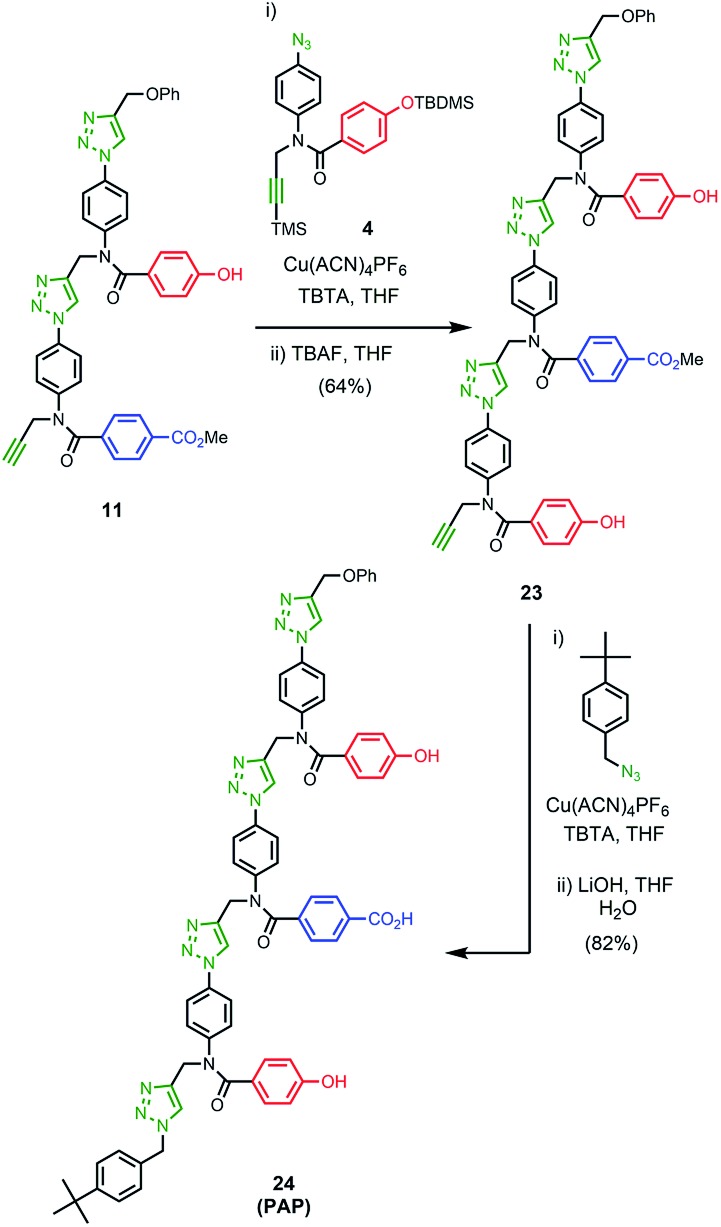


The ^1^H NMR spectrum of the copy strand is more complicated (Fig. 9(c)). Although there is one major product (green signals), there are traces of two other species (blue and purple signals). The mass spectrum of this sample indicates that all three species contain one **A** and two **P** units, so the products must be the three isomeric sequences: **APP**, **PPA** and **PAP**. Pure samples of these three oligomers were obtained by direct synthesis using a similar sequence of CuAAC and TMS deprotection reactions used to prepare the template oligomer **AAP**. The synthesis of **APP** and **PAP** exploited intermediates **10** and **11** used in the synthesis of the original template (Schemes 3 and 5). The synthesis of **PPA** used the phenol dimer **20**, which was fortuitously obtained from spontaneous azide–alkyne cycloaddition of the monomer building block **9** upon storage (Scheme 4).

Fig. 9(c) compares the ^1^H NMR spectra of the independently synthesised samples of **APP**, **PPA** and **PAP** with the ^1^H NMR spectrum of the copy sample obtained from the template-directed synthesis reaction. The major product (72%) from the templating reaction is **APP**, the sequence-complementary copy of the **AAP** template resulting from the antiparallel duplex shown in Fig. 6. One of the minor products (11%) is **PPA**, the sequence-complementary copy of the **AAP** template that would result from formation of the parallel duplex in the **ZIP** step. The other minor species (17%) is the scrambled sequence **PAP**, which must result from intramolecular coupling between the two terminal monomer units on the template. We conclude that the templating cycle proceeds through the antiparallel duplex to give a single major product, which is the sequence complement of the template. The fidelity of the information transfer process is degraded somewhat by a side-reaction that involves long range coupling between monomer units that are not attached at adjacent sites on the template.

## Conclusions

Biological synthesis of oligomers of defined sequence is based on non-covalent template-directed synthesis. We have developed an alternative chemical method based on covalent template-directed synthesis. The covalent base-pairing methodology described here provides a mechanism for quantitative attachment of monomers to a template without the competing equilibria that occur with reversible base-pairing interactions.^54^ The result is a robust method for the transfer of sequence information between parent and daughter strands. A mixed sequence trimer was successfully used to template the synthesis of a sequence-complementary copy. Minor quantities of a scrambled sequence were also observed, but rather than compromising the fidelity of the information transfer process, scrambled sequences could serve as a useful source of mutation, if these systems are developed for evolutionary searching of chemical structure space.

## Conflicts of interest

There are no conflicts to declare.

## Supplementary Material

Supplementary information
