# Supplementary material for: Sequence information transfer using covalent template-directed synthesis
Source: Chem Sci. 2019 Apr 26;10(20):5258–66. doi: 10.1039/c9sc01460h (PMC6540929; doi:10.1039/c9sc01460h)
Supplement: Supplementary file 1 [file SC-010-C9SC01460H-s001.pdf]

Electronic Supplementary Material (ESI) for Chemical Science.
This journal is © The Royal Society of Chemistry 2019

Supporting information

Sequence information transfer using covalent template-directed synthesis

Diego Núñez-Villanueva, Maria Ciaccia, Giulia Iadevaia, Elena Sanna, Christopher A. Hunter*

Department of Chemistry, University of Cambridge, Lensfield Road, Cambridge CB2 1EW, UK. E-mail: herchelsmith.orgchem@ch.cam.ac.uk

TABLE OF CONTENTS **Page**

General experimental details S2

Covalent base-pairing S3

Step 1: Phenol protection (P) S4

Step 2: Ester coupling (C1) S5

Step 3: TBAF-mediated phenol deprotection (dP) S6

Step 4: Ester coupling (C2) S7

Step 5: Cleavage (hydrolysis) S8

Synthesis of monomers S10

Synthesis of AAP template S35

Synthesis of APP 3-mer S52

Synthesis of PPA 3-mer S66

Synthesis of PAP 3-mer S86

Template-directed information transfer S97

Step 1: Phenol protection (P). S98

Step 2: Ester coupling (C1). S99

Step 3: TBAF-mediated phenol deprotection (dP). S101

Step 4: Ester coupling (C2). S103

Step 5: ZIP reaction S111

Step 6: Hydrolysis S113

Step 7: Capping reaction with phenyl propargyl ether S123

Molecular modelling calculations S132

References S136

General experimental details

All the reagents and materials used in the synthesis of the compounds described below were bought from commercial sources, without prior purification. Dry THF and CH_2Cl_2 were obtained from a solvent purification system (Pure Solv™, Innovative Technology, Inc.). Anhydrous DMF was purchased from Sigma-Aldrich. Thin layer chromatography was carried out using with silica gel 60F (Merck) on glass plates. Flash chromatography was carried out on an automated system (Combiflash Rf+ or Combiflash Rf Lumen) using prepacked cartridges of silica (25µ PuriFlash® columns). All NMR spectroscopy was carried out on a Bruker 400 MHz DPX400, 400 MHz AVIII400, 500 MHz DCH cryoprobe or 500 MHz TCI Cryoprobe spectrometer using the residual solvent as the internal standard. All chemical shifts (δ) are quoted in ppm and coupling constants given in Hz. Splitting patterns are given as follows: s (singlet), bs (broad singlet), d (doublet), t (triplet), q (quadruplet), m (multiplet). FT-IR spectra were measured on a PerkinElmer Spectrum One spectrometer equipped with an ATR cell. Melting points were measured in a Mettler Toledo MP50 Melting Point System. UPLC analysis of samples was performed using Waters Acquity H-class UPLC coupled with a single quadrupole Waters SQD2. ACQUITY UPLC CSH C18 Column, 130Å, 1.7 µm, 2.1 mm X 50 mm was used as the UPLC column. The conditions of the UPLC method are as follows: water +0.1% formic acid (solvent A); acetonitrile +0.1% formic acid (solvent B); gradient of 0-2 minutes 5% - 100%B + 1 minute 100%, 35% - 65%B + 1 minute 100% or 65% - 100%B + 1 minute 100% as specified in each case. Flow rate: 0.6 ml/min; Column temperature of 40°C; Injection volume of 2 µL. The signal was monitored at 254 nm. HRMS analysis was performed in a Waters LCT Premier equipped with a TOF mass analyser and W optics for enhanced resolution, using 50% aqueous acetonitrile with 0.25% formic acid as mobile phase.

Covalent base-pairing

The covalent base pairing is based on the cycle of reactions illustrated in Scheme S1 for a model system, where the two different types of base, phenol and carboxylic acid, are separate molecules. Starting from a mixture of two template bases, sequential formation of two base pairs is performed via a sequence of phenol protection (P)-ester coupling (C1)-phenol deprotection (dP)-ester coupling (C2). Cleavage of the base-pairs by hydrolysis regenerates the two template bases as well as two product bases that would correspond to the replicated product in an oligomer templated reaction.

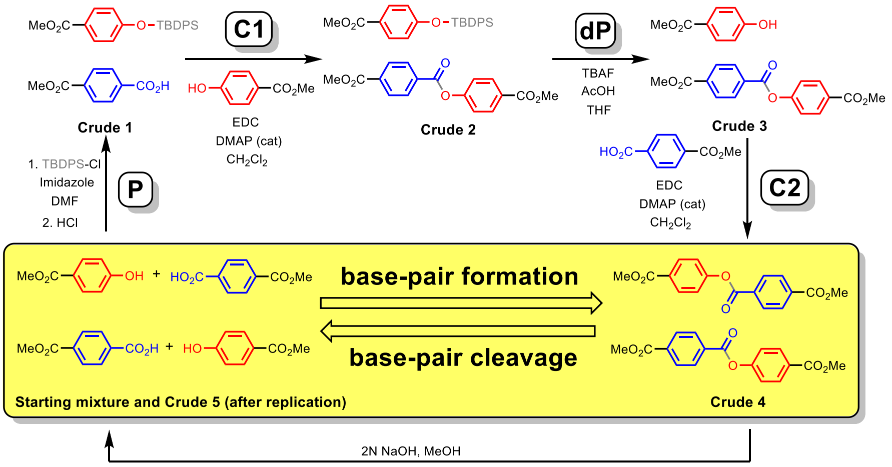

Scheme S1.

Figure S1 shows the corresponding ^{1}H NMR spectra of the crude reaction mixtures after aqueous work-up with no intermediate purification steps. The obtained spectra demonstrate that selective formation and cleavage of phenol-carboxylic acid esters can be achieved quantitatively and this covalent base-pair therefore constitutes an ideal starting point for exploring the potential of covalently templated chemistry. Top spectrum shows a starting 1:1 mixture of the template bases, phenol (red signals) and carboxylic acid (blue signal). Crude 2 was obtained after formation of the first base-pair (green signals), and crude 4 after formation of the second base-pair. Bottom spectrum shows the products of the hydrolysis reaction used to cleave the base-pairs, and is identical to top spectrum apart from the acid residue, which has been hydrolyzed to give terephthalic acid. Detail analysis of each step can be found below, including the experimental procedures.

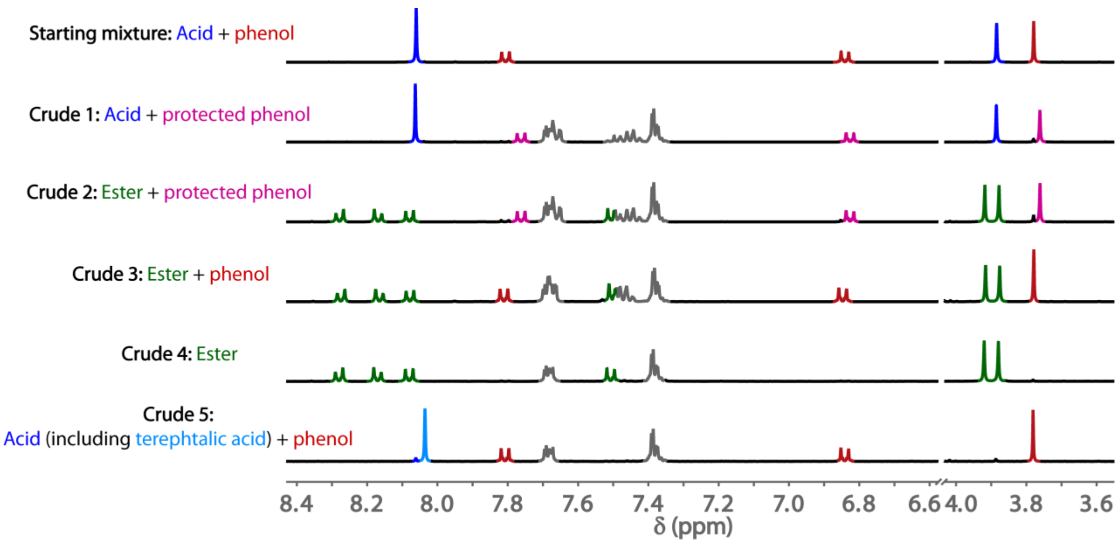

Figure S1. Partial 400 MHz ^{1}H NMR spectra in DMSO-d_6 of the **starting mixture** formed by mono-methyl terephthalate (blue) and methyl 4-hydroxybenzoate (red)**; crude 1** after phenol protection step (P) as a mixture of acid (blue) and protected phenol (pink); **crude 2** after formation of ester (C1) as a mixture of 4-(methoxycarbonyl)phenyl methyl terephthalate (green) and protected phenol (pink); **crude 3** after deprotection step (dP) as a mixture of 4-(methoxycarbonyl)phenyl methyl terephthalate (green) and phenol (red); **crude 4** after coupling step formed by 4-(methoxycarbonyl)phenyl methyl terephthalate (green); and **crude 5** after hydrolysis as mixture of mono-methyl terephthalate (blue), terephthalic acid (pale blue) and methyl 4-hydroxybenzoate (red). TBDPS residues are shown in grey.

Step 1: Phenol protection (P)

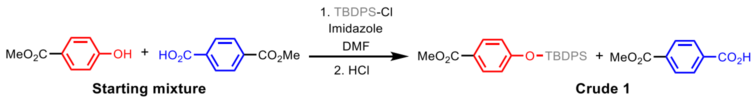

Scheme S2

A solution of methyl 4-hydroxybenzoate (0.150 g, 0.99 mmol) and mono-methyl terephthalate (0.178 g, 0.99 mmol) in DMF (5 mL) was treated with imidazole (0.269 g, 3.94 mmol) and TBDPS-Cl (0.564 mL, 2.17 mmol). After 15 h of stirring at room temperature, the reaction was quenched by addition of 0.1M HCl soln. until reaching pH= 3-4. The solution was stirred at room temperature for 45 min, then diluted with H_2O and extracted with EtOAc (3x). The combined organic layer was washed with 5% LiCl soln. (2x), H_2O (1x) and brine (1x), dried with anhydrous $MgSO_4$, filtered, and the solvent evaporated. The obtained residue (**crude 1**) was used in the next step without further purification. Figure S2 shows the ^{1}H NMR spectra in DMSO-d_6 of the **starting mixture** for this step and obtained **crude 1**. Based on the integrals, the starting acid:phenol ratio is 1.09:1 and the obtained acid:protected phenol ratio is 1.09:1.

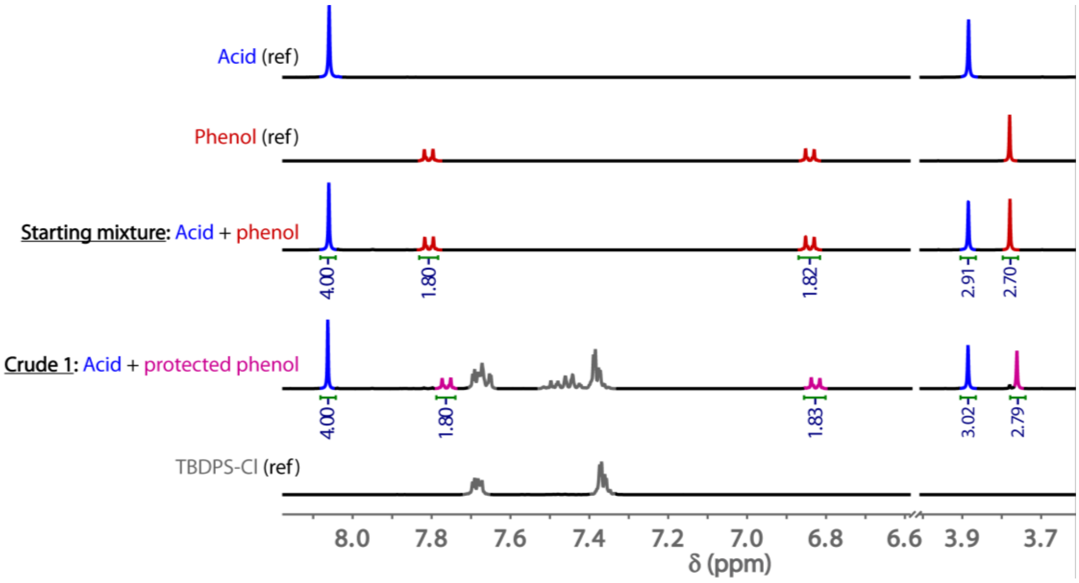

Figure S2. Partial 400 MHz ^{1}H NMR spectra in DMSO-d_6 of reference mono-methyl terephthalate (blue); reference methyl 4-hydroxybenzoate (red); **starting mixture** for step 1; **crude 1** as a mixture of acid (blue) and protected phenol (pink); and reference TBDPS-Cl (grey).

Step 2: Ester coupling (C1)

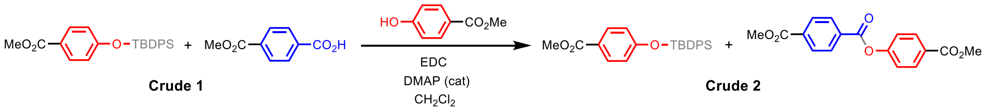

Scheme S3

Crude 1 (0.99 mmol respect to starting mono-methyl terephthalate), methyl 4-hydroxybenzoate (0.158 g, 1.04 mmol), EDC (0.227 g, 1.18 mmol) and DMAP (0.012 g, 0.10 mmol) were added to a flask. Under N_2 atmosphere, dry CH_2Cl_2 (5 mL) was added and reaction was stirred at room temperature for 1 h. Once finished, the reaction was diluted with EtOAc and washed with 0.1M HCl soln. (2x), H_2O (2x) and brine (1x). The solution was dried with anhydrous $MgSO_4$, filtered and the solvent evaporated. The obtained residue (**crude 2**) was used in the next step without further purification. Figure S3 shows the ^{1}H NMR spectra in DMSO-d_6 of the starting material for this step (**crude 1**) and **crude 2**. Based on the integrals, the starting acid:protected phenol ratio is 1.09:1 and the obtained ester:protected phenol ratio is 1.10:1.

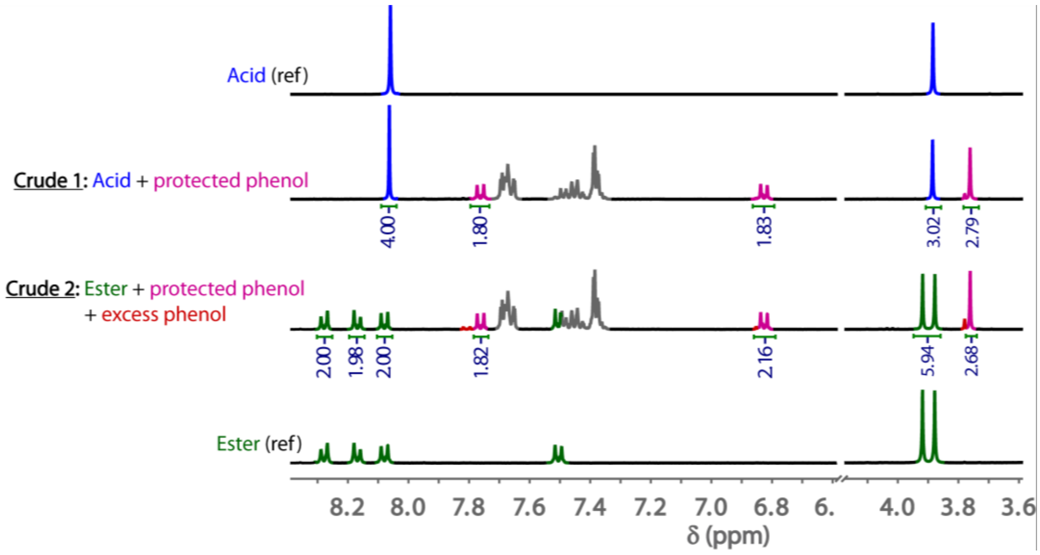

Figure S3. Partial 400 MHz ^{1}H NMR spectra in DMSO-d_6 of reference mono-methyl terephthalate (blue); starting material for step 2: **crude 1** as a mixture of acid (blue) and protected phenol (pink); **crude 2** as mixture of 4-(methoxycarbonyl)phenyl methyl terephthalate (green), protected phenol (pink) and excess phenol (red); and reference 4-(methoxycarbonyl)phenyl methyl terephthalate (green). TBDPS residues are shown in grey.

Synthesis of 4-(methoxycarbonyl)phenyl methyl terephthalate as a reference: mono-methyl terephthalate (0.500 g, 2.78 mmol), methyl 4-hydroxybenzoate (0.0.427 g, 2.80 mmol) DMAP (0.034 g, 0.28 mmol) and EDC (0.638 g, 3.33 mmol) were dissolved in dry CH_2Cl_2 (10 mL) and stirred at room temperature for 1 h. After completion, the reaction was quenched with 0.1M HCl soln. and extracted with EtOAc (3x). The combined organic phase was washed with brine and dried with anhydrous $MgSO_4$. 4-(Methoxycarbonyl)phenyl methyl terephthalate was obtained following this procedure as a white solid (0.871 g, quantitative).

^{1}H NMR (400 MHz, $CDCl_3$): δ_H = 8.27 (d, 2H, *J* = 8.5 Hz, H_{arom} tereph.), 8.18 (d, 2H, *J* = 8.5 Hz, H_{arom} tereph.), 8.14 (d, 2H, *J* = 9.0 Hz, 3-H), 7.32 (d, 2H, *J* = 9.0 Hz, 2-H), 3.98 (s.3H, OCH_3), 3.94 (s.3H, OCH_3).

^{13}C NMR (100.6 MHz, $CDCl_3$): δ_C =170.3, 170.4 and 170.4 (CO), 151.3 (1-C), 137.0, 133.0, 131.5, 131.0, 130.4 and 130.0 (C_{arom}), 121.8 (2-C), 52.4 and 52.7 (OCH_3).

Step 3: TBAF-mediated phenol deprotection (dP)

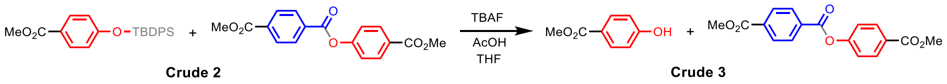

Scheme S4

Crude 2 (0.99 mmol respect to starting mono-methyl terephthalate) was dissolved in dry THF (10 mL) under N_2 atmosphere. After cooling down the solution to -30 °C, TBAF (1M in THF, 0.99 mL, 0.99 mmol) was added dropwise. After 10 minutes of stirring at -30 °C, the reaction was quenched with 0.1M HCl soln. and diluted with EtOAc. The organic layer was separated and washed with 0.1M HCl soln. (2x), H_2O (1x) and brine (1x). The solution was dried with

anhydrous $MgSO_4$, filtered and the solvents evaporated. The obtained residue (**crude 3**) was used in the next step without further purification. Figure S4 shows the ^1H NMR spectra in DMSO-d_6 of the starting material for this step (**crude 2**) and **crude 3**. Based on the integrals, the starting ester:phenols (both protected and unprotected excess) ratio is 0.93:1 and the obtained ester:phenol ratio is 0.91:1.

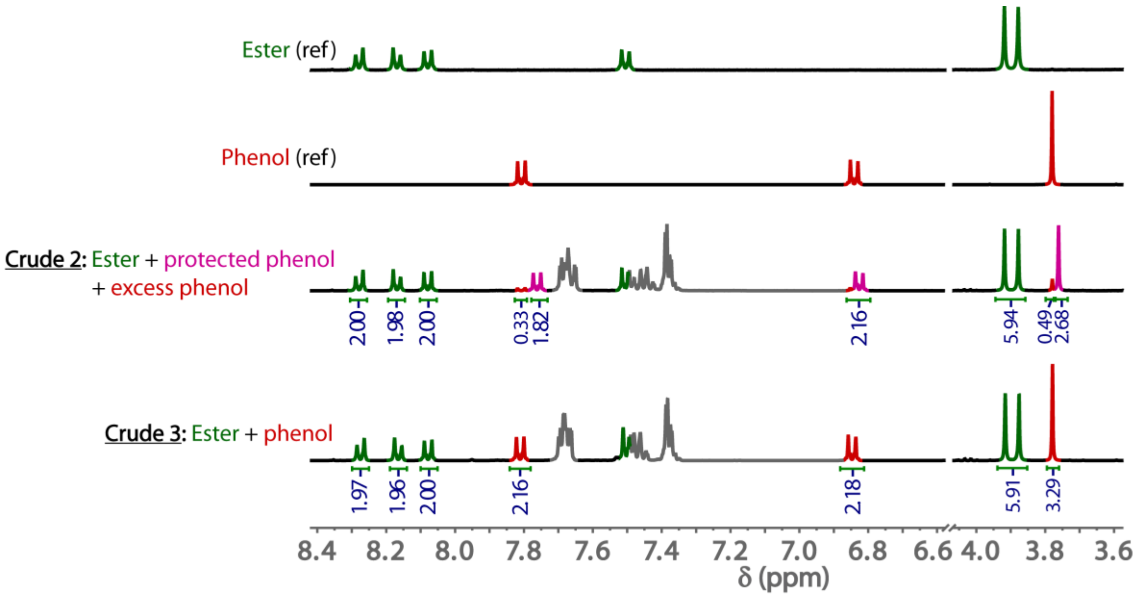

Figure S4. Partial 400 MHz ^1H NMR spectra in DMSO-d_6 of reference 4-(methoxycarbonyl)phenyl methyl terephthalate (green); reference methyl 4-hydroxybenzoate (red); starting material for step 3: **crude 2** as a mixture of ester (green), protected phenol (pink) and excess phenol (red); **crude 3** as mixture of ester (green) and phenol (red). TBDPS residues are shown in grey.

Step 4: Ester coupling (C2)

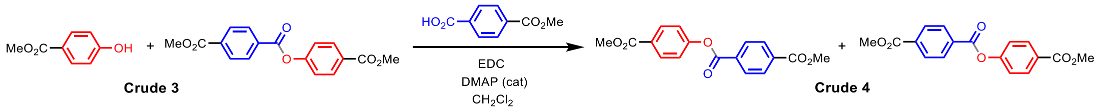

Scheme S5

Crude 3 (0.99 mmol respect to starting mono-methyl terephthalate), mono-methyl terephthalate (0.187 g, 1.04 mmol), EDC (0.227 g, 1.18 mmol) and DMAP (0.012 g, 0.10 mmol) were added to a flask. Under N_2 atmosphere, dry CH_2Cl_2 (10 mL) was added and reaction was stirred at room temperature for 1 h. Once finished, the reaction was diluted with EtOAc and washed with 0.1M HCl soln. (2x), H_2O (1x) and brine (1x). The solution was dried with anhydrous $MgSO_4$, filtered and the solvent evaporated. The obtained residue (**crude 4**) was used in the next step without further purification. Figure S5 shows the ^1H NMR spectra in DMSO-d_6 of the starting material for this step (**crude 3**) and **crude 4**.

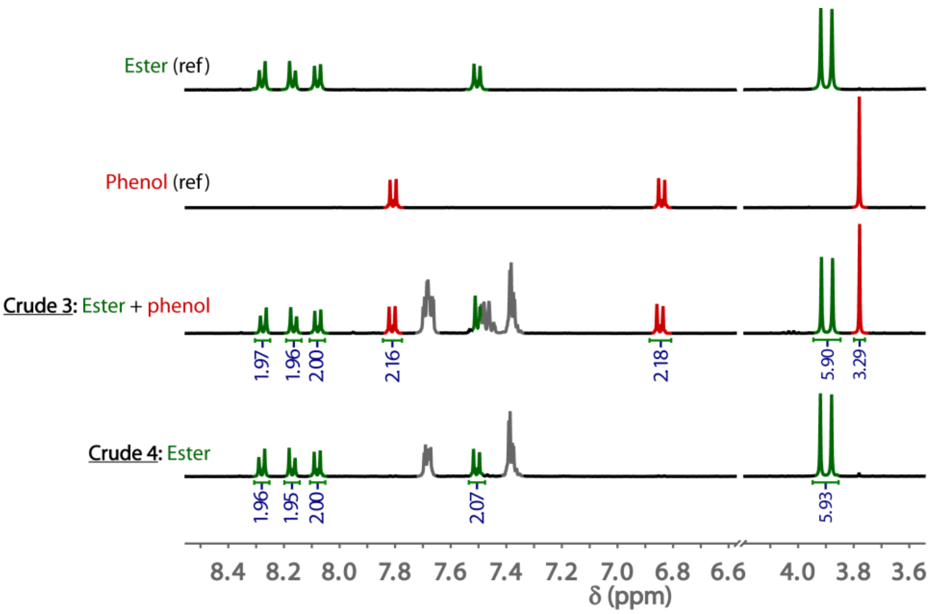

Figure S5. Partial 400 MHz ^{1}H NMR spectra in DMSO-d_6 of reference 4-(methoxycarbonyl)phenyl methyl terephthalate (green); reference methyl 4-hydroxybenzoate (red); starting material for step 4: **crude 3** as a mixture of ester (green) and phenol (red); and **crude 4** formed by 4-(methoxycarbonyl)phenyl methyl terephthalate (green). TBDPS residues are shown in grey.

Step 5: Cleavage (hydrolysis)

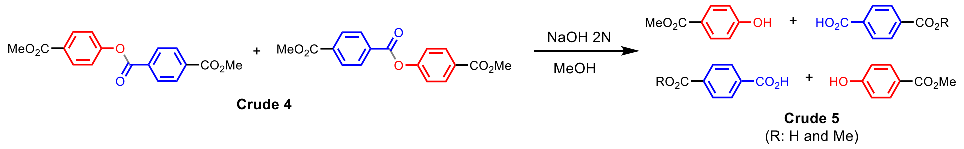

Scheme S6

Crude 4 (0.075 g, 0.24 mmol) was dissolved in MeOH (2 mL) and 2N NaOH soln. was added (1.20 mL, 2.39 mmol). After 1 h of stirring at room temperature, the reaction was quenched by addition of 0.1M HCl soln. until reaching pH= 3-4. The solution was diluted with H_2O and extracted with EtOAc (3x). The combined organic layer was washed with brine (1x), dried with anhydrous $MgSO_4$, filtered, and the solvent evaporated, yielding **crude 5**. Figure S6 shows the ^{1}H NMR spectra in DMSO-d_6 of the starting material for this step (**crude 4**) and **crude 5**, which is a mixture of acid residue (mostly hydrolyzed forming terephthalic acid) and phenol residue. Based on the integrals, the phenol: acid (both mono-methyl terephthalate and terephthalic acid) ratio is 1:1.

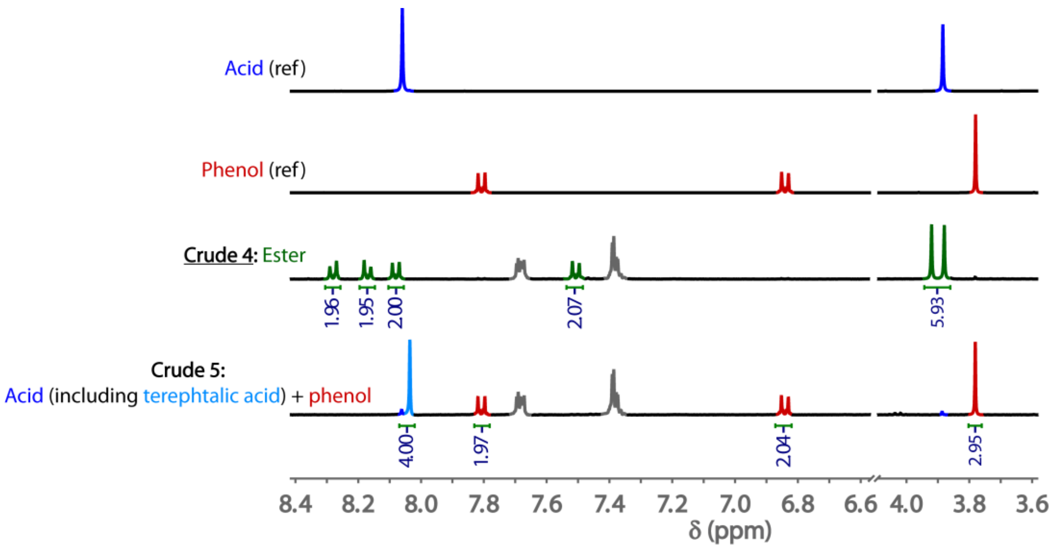

Figure S6. Partial 400 MHz ^{1}H NMR spectra in DMSO-d_6 of reference mono-methyl terephthalate (blue); reference methyl 4-hydroxybenzoate (red); starting material for step 5: **crude 4** formed by 4-(methoxycarbonyl)phenyl methyl terephthalate (green); and **crude 5** as mixture of mono-methyl terephthalate (blue), terephthalic acid (pale blue) and methyl 4-hydroxybenzoate (red). TBDPS residues are shown in grey.

Synthesis of monomers

The synthesis of *p*-azidoaniline-derived phenol (red) and carboxylic acid (blue) bases in shown in Scheme S7. It involves the preparation of *p*-azidoaniline **2**, according to a procedure described in the literature (Scheme 11).[S1] Amide coupling of **2** and mono-methyl terephthalate yielded **3** in excellent yield. Alkylation of **19** using TMS-protected propargyl bromide and sodium hydride gave **4**, together with TMS-deprotected alkyne **5** in good overall yield. Hydrolysis of **5** using lithium hydroxide afforded carboxylic acid monomer base **6** in excellent yield. Same procedure was used to synthesize the phenol monomer base. Amide coupling of **2** and TBDMS-protected 4-hydroxybenzoic acid gave access to **7** in excellent yield. Subsequent alkylation with TMS-protected propargyl bromide, providing **8** in good yield, followed by TBAF-mediated deprotection of silyl protecting groups gave phenol monomer base **9**.

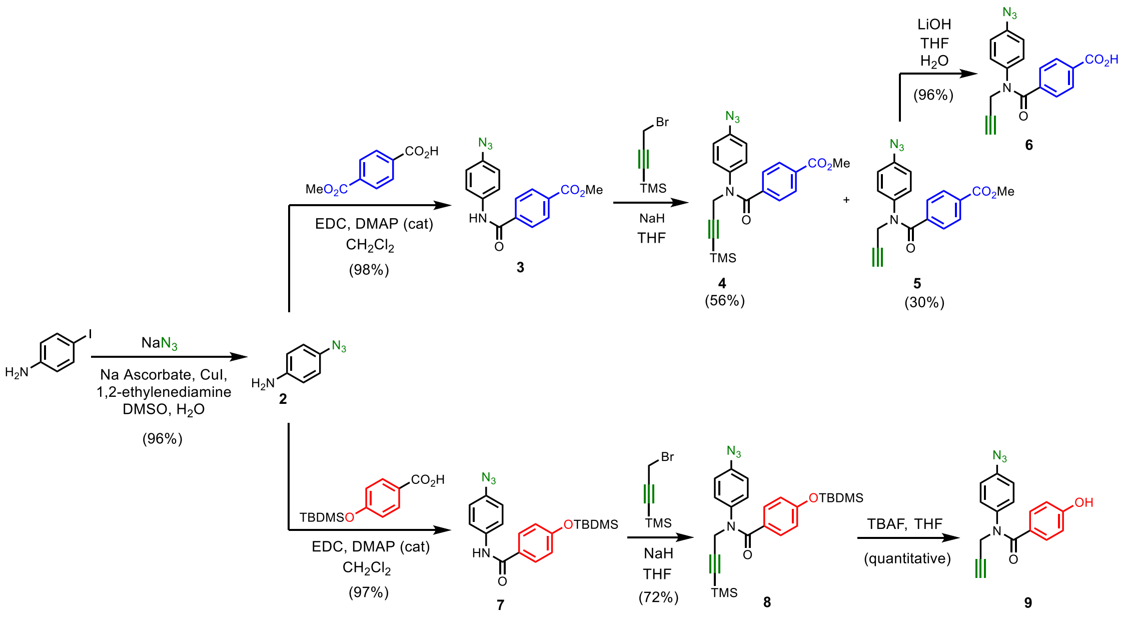

Scheme S7

p-Azidoaniline (2)

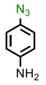

p-Azidoaniline (**2**) was synthesized according to a method described by Li *et al* (*Polym. Chem.* **2013**, *4*, 3057). CuI (0.087 g, 0.46 mmol) and 1,2-ethylenediamine (0.046 mL, 0.69 mmol) were added solution of *p*-iodoaniline (1.00 g, 4.57 mmol), NaN_3 (0.89 g, 13.70 mmol) and sodium ascorbate (0.045 g, 0.23 mmol) in DMSO:H_2O (5:1, 12 mL) under N_2 atmosphere. After overnight stirring, the reaction was diluted with EtOAc:brine (3:1), and the organic layer separated and washed with brine (5x). The organic layer was dried over MgSO4, filtered and evaporated to dryness. The crude was passed through a pad of silica using Pet. Ether:EtOAc 1:1, affording *p*-azidoaniline as a brown solid (0.587 g, 96%). The analytical and spectroscopic data match those previously reported in the literature.[S1]

Compound 3.

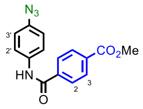

p-Azidoaniline **2** (0.250 g, 1.86 mmol), mono-methyl terephthalate (0.367 g, 2.04 mmol), EDC (0.435 g, 2.27 mmol) and DMAP (0.020 g, 0.25 mmol) were dissolved in dry CH_2Cl_2 (25 ml) and the reaction was left stirring under N_2 atmosphere at room temperature for 1h. The crude was diluted with EtOAc (50 mL) and washed with 5% aq. soln. HCl (2x), 2N NaOH (2x), H_2O (2x) and brine. The organic phase was dried with $MgSO_4$ and concentrated *in vacuo* to yield **3** as an orange solid (0.543 g, 98 %).

Melting point: Decomposition before melting.

^{1}H NMR (400 MHz, $CDCl_3$): δ_H = 8.16 (d, 2H, *J* = 8.0 Hz, 2-H), 7.93 (d, 2H, *J* = 8.0 Hz, 3-H), 7.81 (s, 1H, NH), 7.65 (d, 2H, *J* = 8.5 Hz, 2'-H), 7.05 (d, 2H, *J* = 8.5 Hz, 3'-H), 3.97 (s, 3H, OCH_3).

^{13}C NMR (100.6 MHz, $CDCl_3$): δ_C = 166.3 and 164.9 (CO), 138.7 (4'-C), 136.6 (1'-C), 134.7 (1-C), 133.3 (4-C), 130.2 (3-C), 127.2 (2-C), 122.0 (3'-C), 119.8 (2'-C), 52.7 (OCH_3).

HRMS (ES+): calcd for $C_{15}H_{13}N_4O_3$ 297.0988 $[M+H]^+$, found 297.0985 $[M+H]^+$.

FT-IR (ATR): ν_{max} 3347, 2114, 1726, 1649, 1526, 1284, 1112, 821 and 733 cm^{-1}.

^{1}H-NMR (400 MHz, $CDCl_3$) Compound 3.

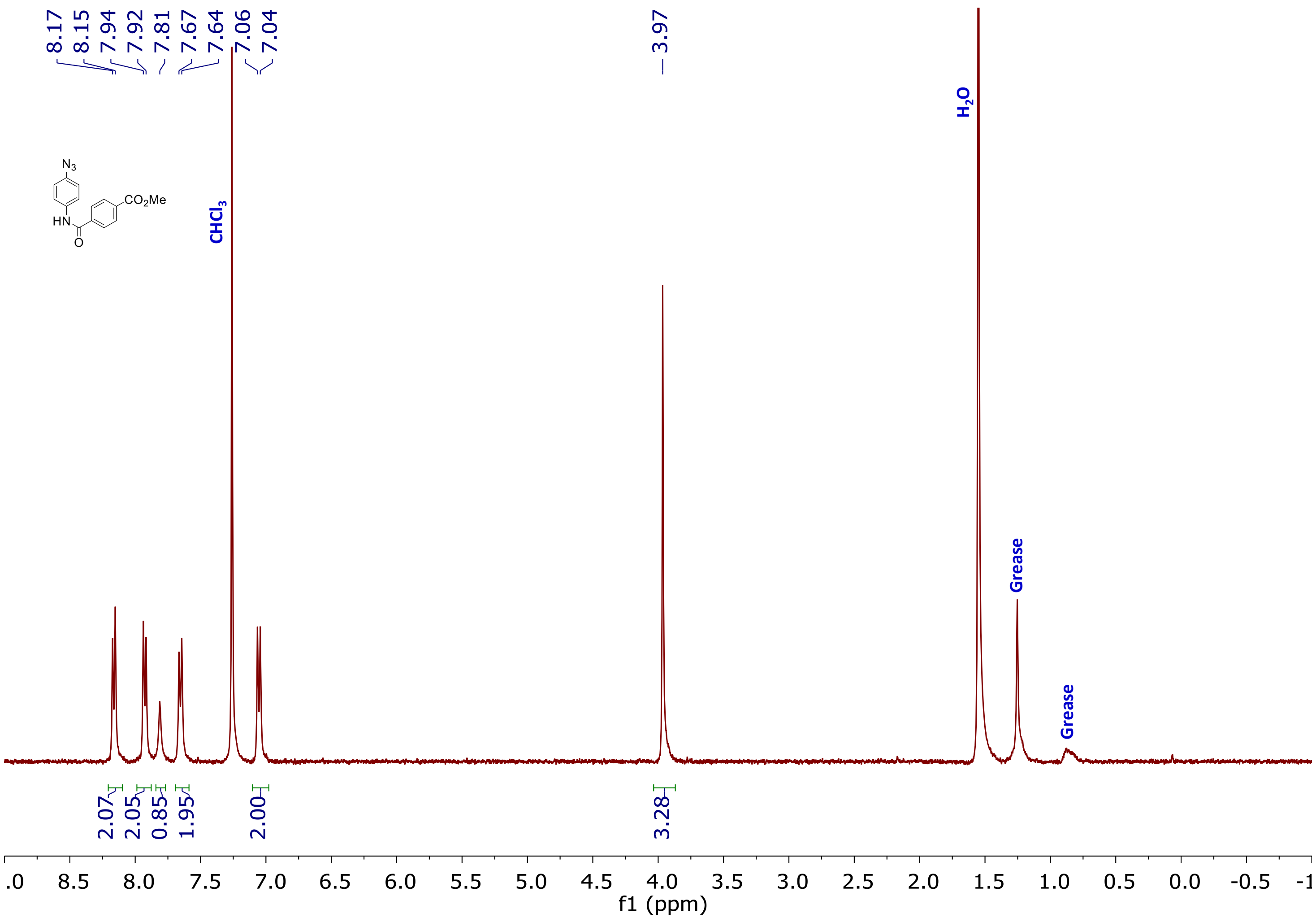

^{13}C-NMR (100.6 MHz, $CDCl_3$) Compound 3.

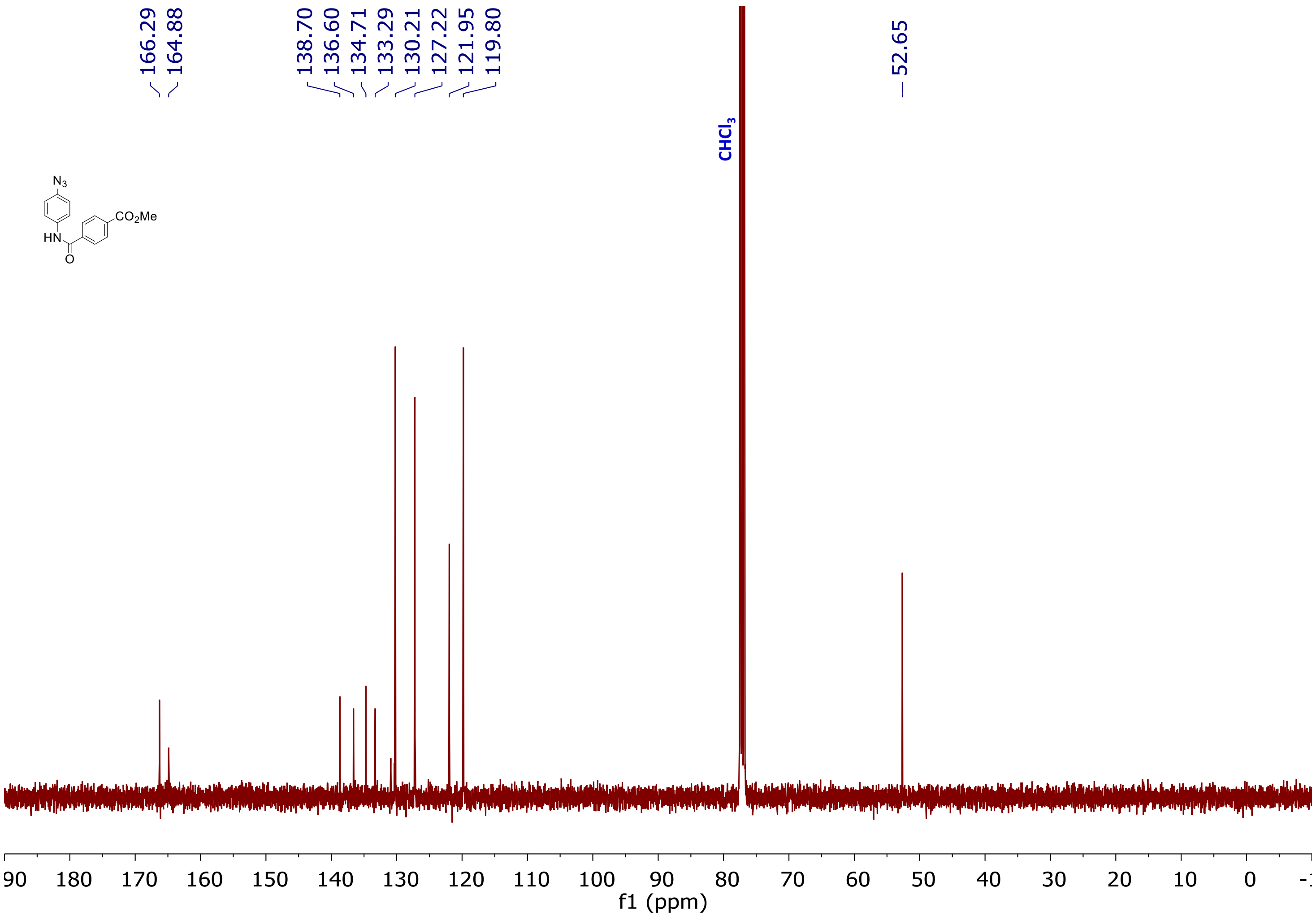

Compounds 4 and 5.

Compound **3** (0.178 g, 0.60 mmol) was dissolved in dry THF (3 mL) and added to a suspension of NaH (60% dispersion in mineral oil, 0.072 g, 1.80 mmol) in THF (4 mL) under inert atmosphere at 0 °C. The reaction was allowed to reach room temperature for 15 min. 3-Bromo-1-(trimethylsilyl)-1-propyne (0.294 mL, 1.80 mmol) was added dropwise and the reaction was vigorously stirred overnight. Satd. aq. NH_4Cl was carefully added at 0 °C and the reaction was extracted with EtOAc (3x). The combined organic phase was washed with brine (1x), dried with anhydrous $MgSO_4$, filtered, and the solvents evaporated. The obtained residue was purified by flash chromatography (from 0% to 25% of EtOAc in Pet. Ether) to afford **4** (0.136 g, 56%) as a yellow oil, and deprotected 1-mer **5** (0.061 g, 30%) as a brown oil.

Compound 4.

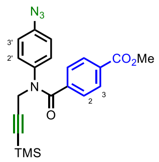

^{1}H NMR (400 MHz, CDCl$_3$): δ_H = 7.87 (d, 2H, *J* = 7.0 Hz, 3-H), 7.38 (d, 2H, *J* = 7.0 Hz, 2-H), 7.11 (d, 2H, *J* = 8.5 Hz, 2'-H), 6.89 (d, 2H, *J* = 8.5 Hz, 3'-H), 4.67 (s, 2H, N-CH_2), 3.88 (s, 3H, OCH_3), 0.13 (s, 9H, TMS).

^{13}C NMR (100.6 MHz, CDCl$_3$): δ_C = 169.2 and 166.4 (CO), 139.7 and 139.5 (1-C and 4'-C), 138.8 (1'-C), 131.3 (4-C), 129.7, 129.3 and 128.6 (2-C, 3-C and 2'-C), 119.8 (3'-C), 100.3 (C-TMS), 90.3 (C, alkyne), 52.4 (OCH_3), 40.4 (N-CH_2), -0.1 (TMS).

HRMS (ES+): calcd for $C_{21}H_{23}N_4O_3Si$ 407.1539 $[M+H]^+$, found 407.1524 $[M+H]^+$.

FT-IR (ATR): 2959, 2126, 2095, 1725, 1655, 1506, 1277, 1109 and 843 ν_{max}/cm^{-1}.

^{1}H-NMR (400 MHz, $CDCl_3$) Compound 4.

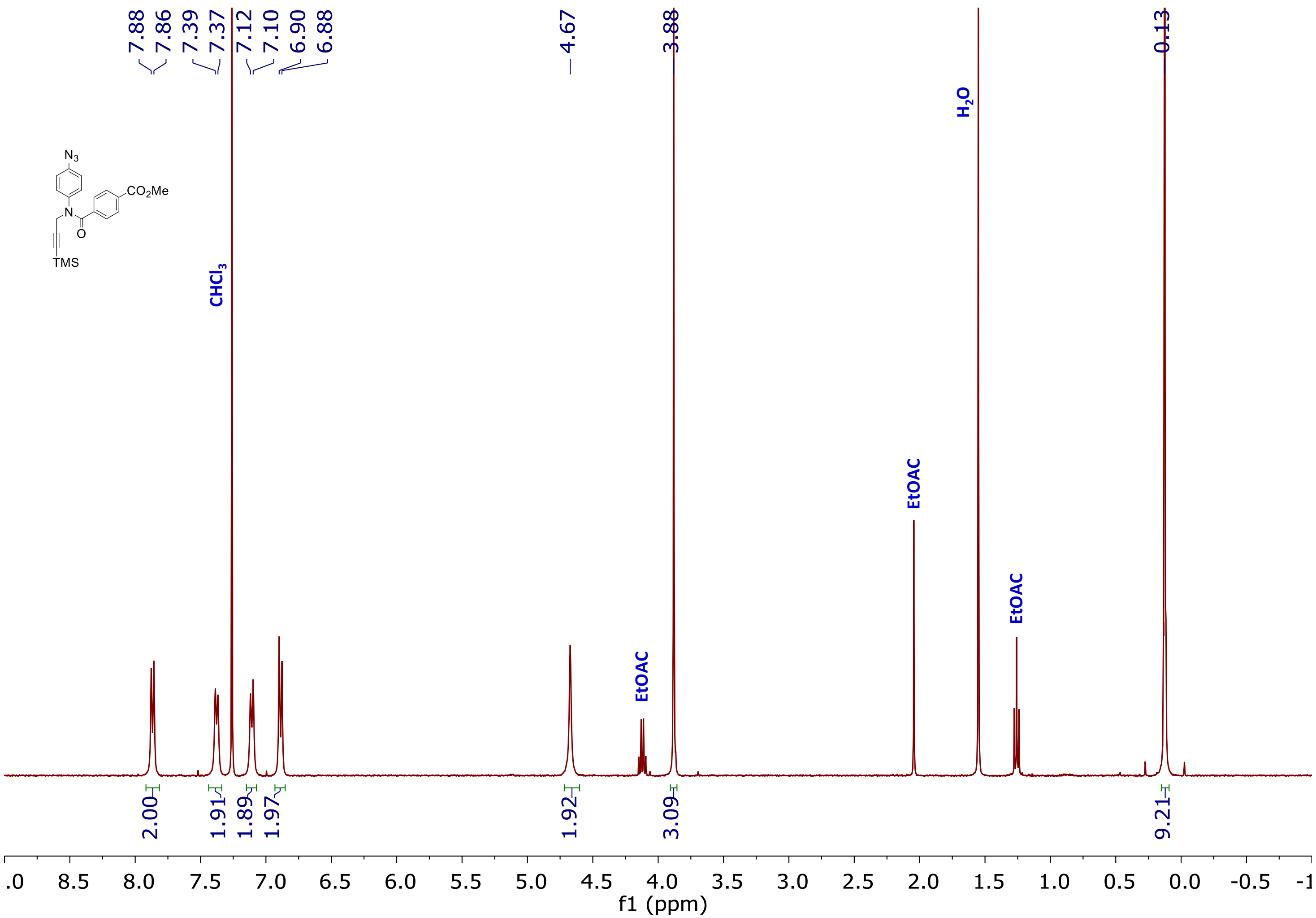

^{13}C-NMR (100.6 MHz, $CDCl_3$) Compound 4.

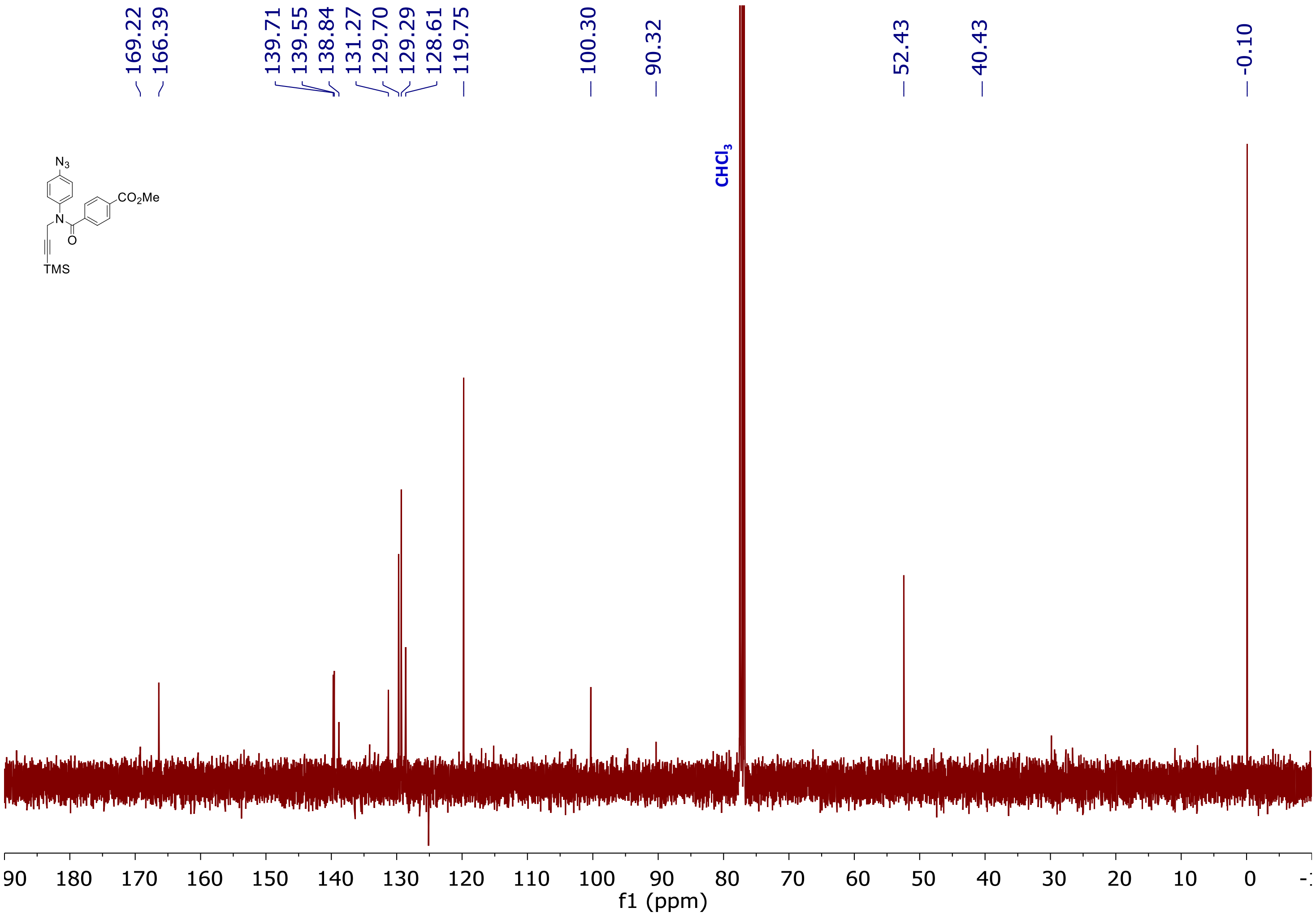

Compound 5.

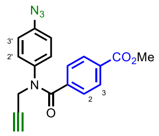

^{1}H NMR (400 MHz, CDCl$_3$): δ_H = 7.87 (d, 2H, J = 7.0 Hz, 3-H), 7.38 (d, 2H, J = 7.0 Hz, 2-H), 7.11 (d, 2H, J = 8.5 Hz, 2'-H), 6.90 (d, 2H, J = 8.5 Hz, 3'-H), 4.66 (m, 2H, N-CH$_2$), 3.88 (s, 3H, OCH$_3$), 2.27 (t, 1H, J = 2.4 Hz, CH, alkyne).

^{13}C NMR (100.6 MHz, CDCl$_3$): δ_C = 169.3 and 166.2 (CO), 139.5 and 139.5 (1-C and 4'-C), 138.8 (1'-C), 131.3 (4-C), 129.3, 129.2 and 128.6 (2-C, 3-C and 2'-C), 119.9 (3'-C), 78.6 (C, alkyne), 72.9 (C, alkyne), 52.4 (OCH$_3$), 39.7 (N-CH$_2$).

HRMS (ES+): calcd for $C_{18}H_{15}N_4O_3$ 335.1144 $[M+H]^+$, found 335.1132 $[M+H]^+$.

FT-IR (ATR): ν_{max} 3350, 2928, 2113, 1641, 1602, 1514, 1277, 918 and 836 cm^{-1}.

^{1}H-NMR (400 MHz, $CDCl_3$) Compound 5.

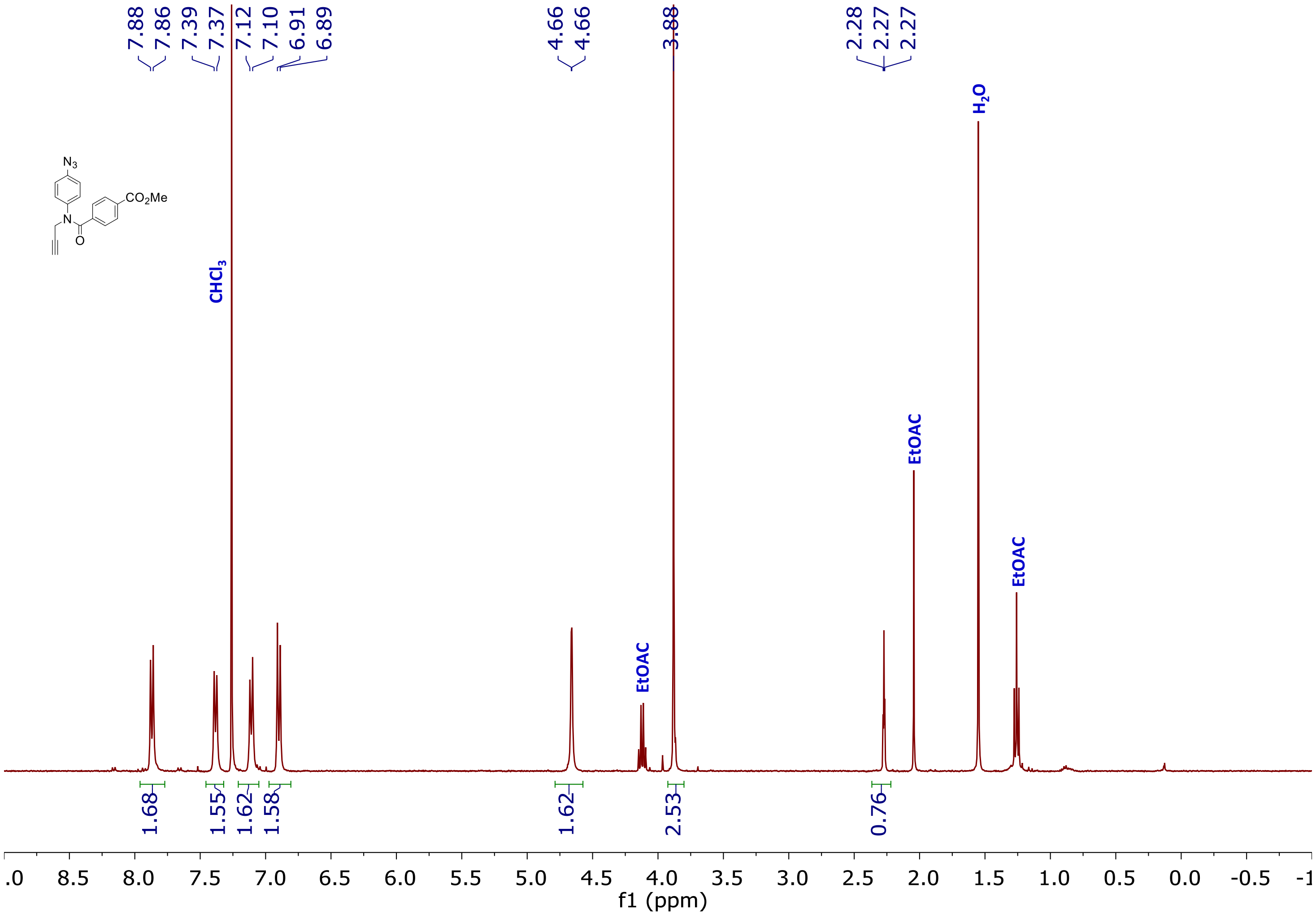

^{13}C-NMR (100.6 MHz, $CDCl_3$) Compound 5.

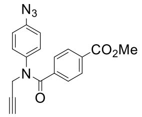

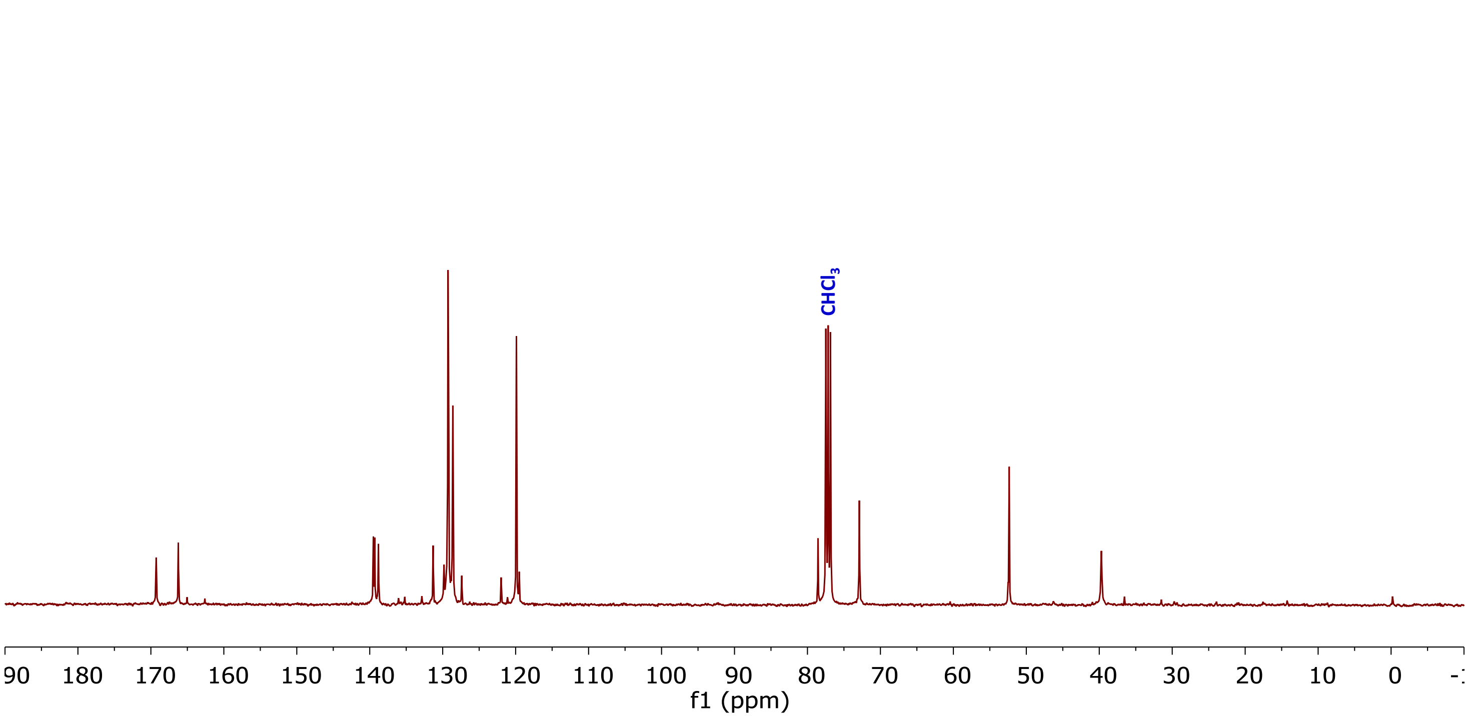

Compound 6.

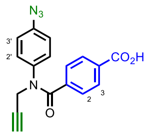

Compound **5** (0.099 g, 0.30 mmol) was dissolved in THF/ H_2O 3:1 (2 mL) and LiOH (0.049 g, 1.18 mmol) was added. After 6 h of stirring at room temperature, the crude was diluted with H_2O and acidified with 1M HCl soln. to pH 2-3. The aqueous phase was extracted with EtOAc (3x) and the combined organic phase was washed with brine, dried over $MgSO_4$ and evaporated to dryness. The obtained material was purified by flash column chromatography on silica gel (gradient from 0% to 40% of EtOAc in Pet. Ether) to afford **6** (0.040 g, 42%) as a yellow oil.

^{1}H NMR (400 MHz, $CDCl_3$): δ_H = 7.92 (d, 2H, *J* = 8.0 Hz, 3-H), 7.40 (d, 2H, *J* = 8.0 Hz, 2-H), 7.11 (d, 2H, *J* = 8.0 Hz, 2'-H), 6.90 (d, 2H, *J* = 8.0 Hz, 3'-H), 4.66 (bs, 2H, N-CH_2), 2.28 (bs, 1H,CH, alkyne).

^{13}C NMR (100.6 MHz, $CDCl_3$): δ_C = 170.8 and 169.3 (CO), 140.1 and 139.7 (1-C and 4'-C), 138.7 (1'-C), 130.6 (4-C), 129.9, 129.4 and 128.8 (2-C, 3-C and 2'-C), 120.0 (3'-C), 78.5 (C, alkyne), 73.0 (CH, alkyne), 39.8 (N-CH_2).

HRMS (ES+): calcd for $C_{17}H_{13}N_4O_3$ 321.0988 $[M+H]^+$, found 321.0920 $[M+H]^+$.

FT-IR (ATR): ν_{max} 3300, 2914, 2850, 2127, 2100, 1719, 1695, 1650, 1506, 1294, 1280, 1226, 759 and 732 cm^{-1}.

^{1}H-NMR (400 MHz, $CDCl_3$) Compound 6.

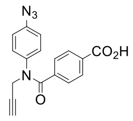

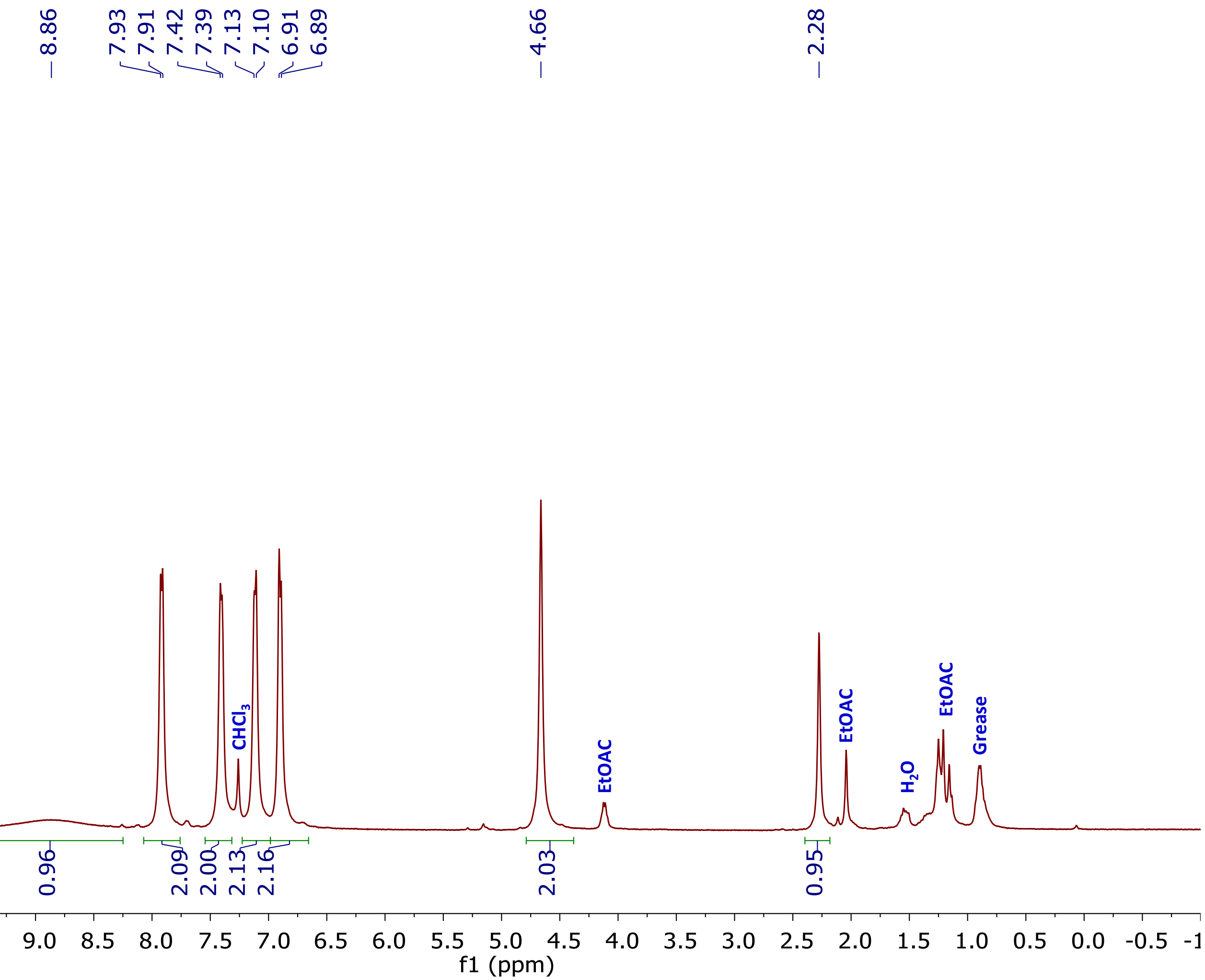

^{13}C-NMR (100.6 MHz, $CDCl_3$) Compound 6.

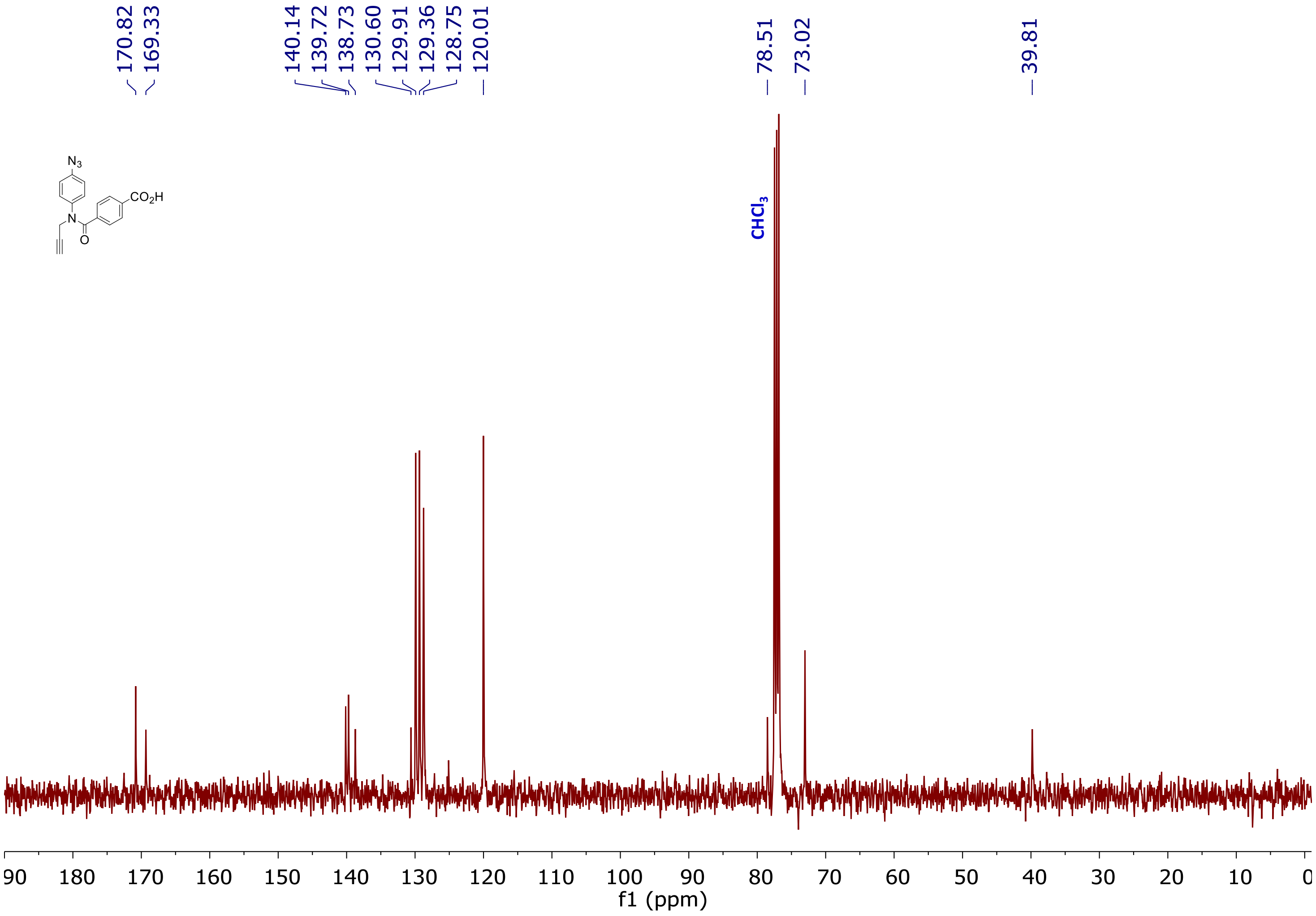

4-((*tert*-butyldimethylsilyl)oxy)benzoic acid.

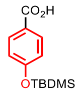

4-Hydroxybenzoic acid (2.00 g, 14.48 mmol) was dissolved in DMF (25 mL) and treated with imidazole (3.94 g, 57.92 mmol) and TBDMS-Cl (6.55 g, 43.44 mmol). The reaction was stirred at room temperature for 2 h. The reaction was quenched with 0.1 N HCl soln. to pH 4-5 and extracted with EtOAc (3x). The combined organic phase was washed with 5% aq. soln. LiCl (3x), H_2O (1x) and brine, dried ($MgSO_4$), and concentrated *in vacuo*. The crude was treated with acetic acid/H_2O/THF 3:1:1 (25 mL) for 2 h and extracted with EtOAc (3x), washed with H_2O (3x) and brine. The organic phase was evaporated to dryness and dried in a high vacuum pump for 12 h. Following this procedure, compound 4-((*tert*-butyldimethylsilyl)oxy)benzoic acid as obtained as a white solid (3.345 g, 92 %).

Melting point: 104-105 °C.

^{1}H NMR (400 MHz, $CDCl_3$): δ_H = 8.01 (d, 2H, *J* = 9.0 Hz, 2-H), 6.89 (d, 2H, *J* = 2.0 Hz, 3-H), 0.99 (s, 9H, ^{t}Bu, TBDMS), 0.24 (s, 6H, CH_3, TBDMS).

^{13}C NMR (100.6 MHz, $CDCl_3$): δ_C = 171.2 (CO), 161.0 (4-C), 132.4 (2-C), 122.3 (1-C), 120.1 (4-C), 25.7 (CH_3, ^{t}Bu, TBDMS), 18.4 (C, ^{t}Bu, TBDMS), -4.2 (CH_3, TBDMS).

HRMS (ES+): calcd for $C_{13}H_{21}O_3Si$ 253.1260 $[M+H]^+$, found 253.1274 $[M+H]^+$.

FT-IR (ATR): ν_{max} 2956, 2927, 2855, 1675, 1599, 1421, 1255, 1167, 904, 861, 835 and 777 cm^{-1}.

^{1}H-NMR (400 MHz, $CDCl_3$) 4-((*tert*-butyldimethylsilyl)oxy)benzoic acid.

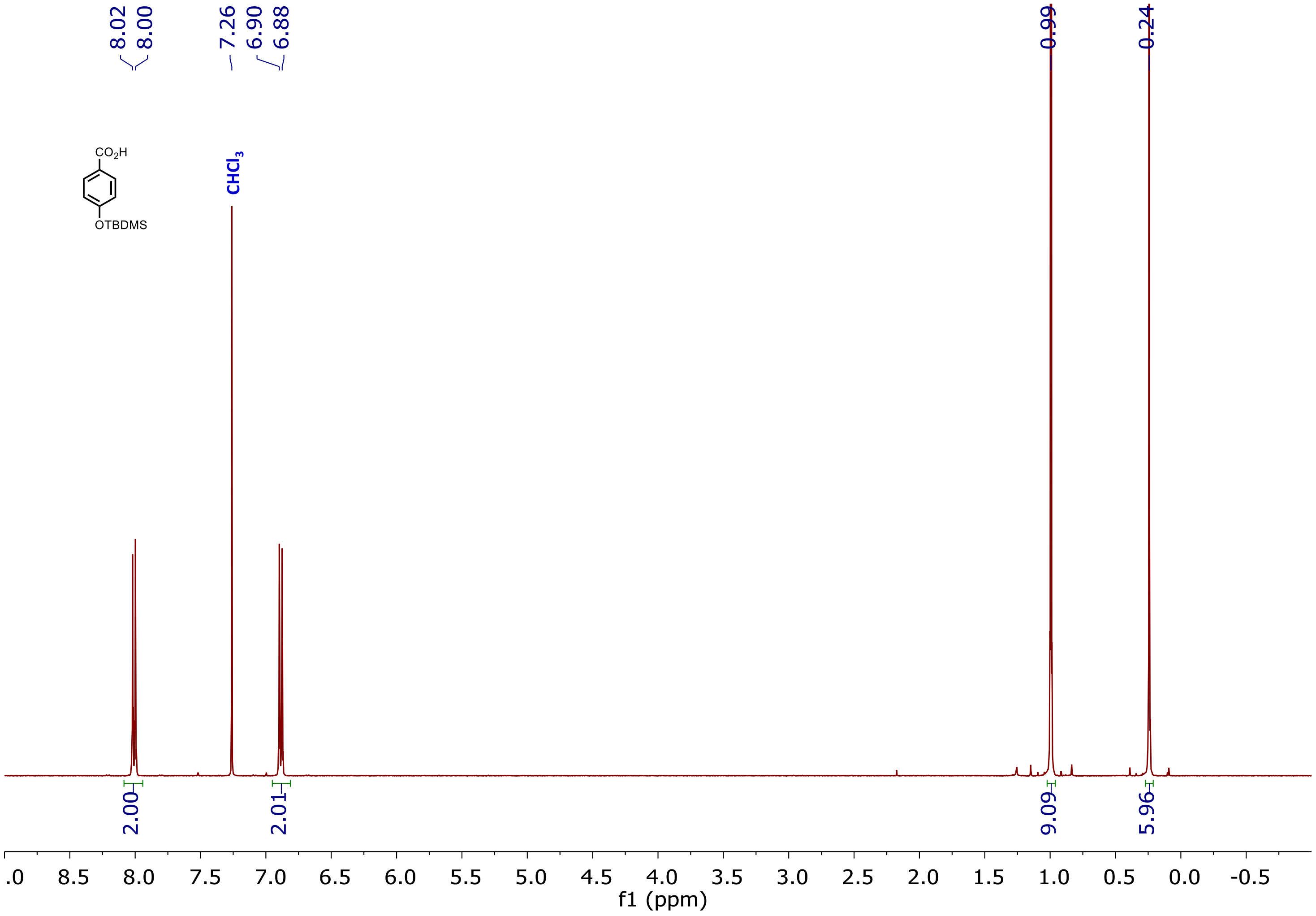

^{13}C-NMR (100.6 MHz, $CDCl_3$) 4-((*tert*-butyldimethylsilyl)oxy)benzoic acid.

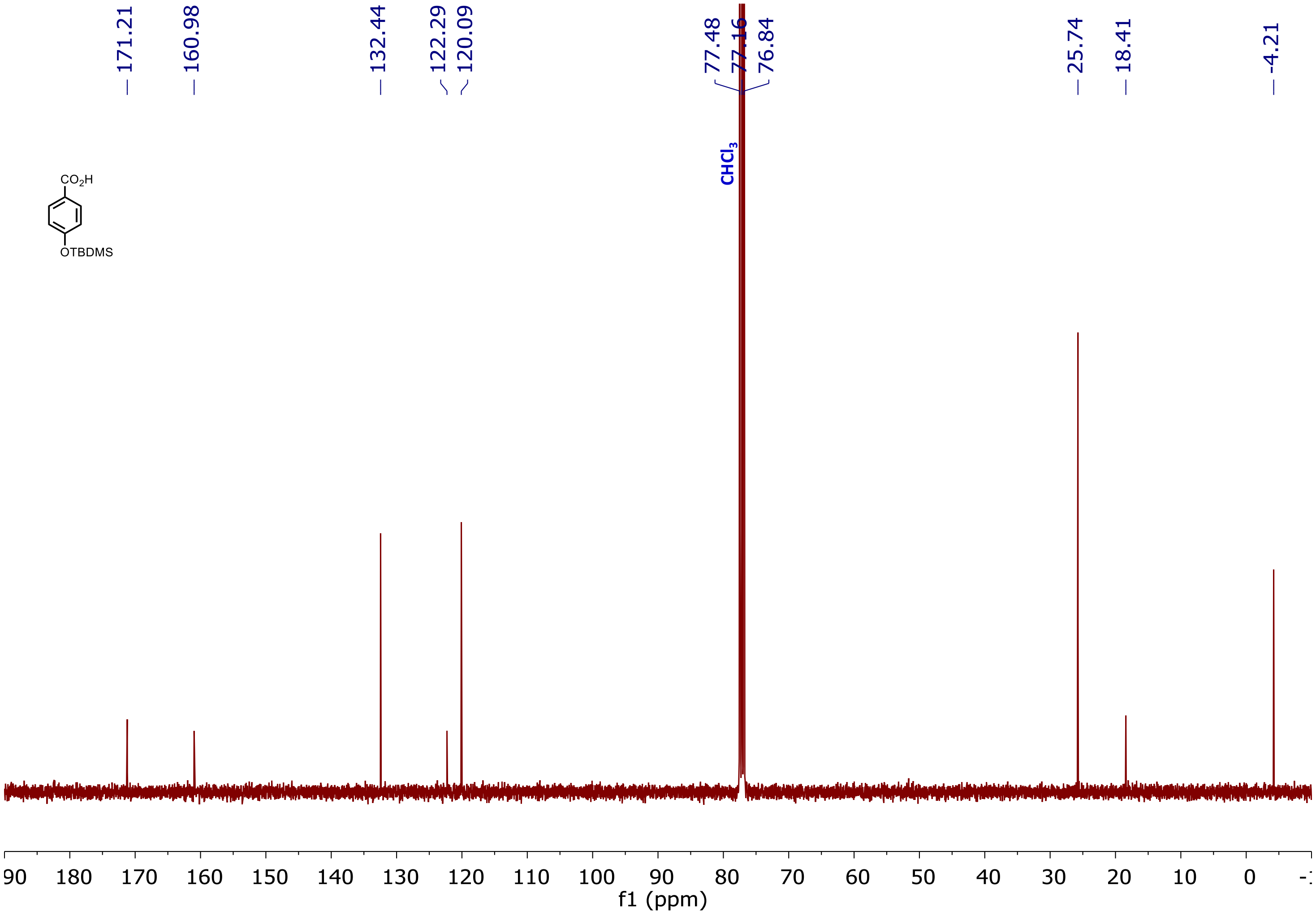

Compound 7.

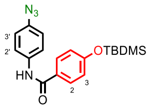

p-Azidoaniline **2** (0.200 g, 1.49 mmol), 4-((*tert*-butyldimethylsilyl)oxy)benzoic acid (0.376 g, 1.49 mmol), EDC (0.343 g, 1.79 mmol) and DMAP (0.036 g, 0.30 mmol) were dissolved in dry CH_2Cl_2 (5 ml) and the reaction was left stirring under N_2 atmosphere at room temperature for 1h. The crude was diluted with EtOAc (50 mL) and washed with 0.1M HCl soln. (2x), 2N NaOH (2x), H_2O (2x) and brine. The organic phase was dried with $MgSO_4$ and concentrated *in vacuo* to yield **7** as an orange solid (0.530 g, 97 %).

Melting point: Decomposition before melting.

^{1}H NMR (400 MHz, CDCl$_3$): δ_H = 7.77 (s partially overlapped, 1H, NH), 7.77 (d, 2H, *J* = 8.5 Hz, 2-H), 7.62 (d, 2H, *J* = 9.0 Hz, 2'-H), 7.01 (d, 2H, *J* = 9.0 Hz, 3'-H), 6.91 (d, 2H, *J* = 8.5 Hz, 3-H), 1.00 (s, 9H, ^{t}Bu, TBDMS), 0.23 (s, 6H, CH_3, TBDMS).

^{13}C NMR (100.6 MHz, CDCl$_3$): δ_C = 165.4 (CO), 159.4 (4-C), 145.4 (4'-C), 135.3 (1'-C), 129.0 (2-C), 127.7 (1-C), 121.8, 120.4 and 119.7 (3-C, 2'-C and 3'-C), 25.8 (^{t}Bu, TBDMS), 18.4 (C, TBDMS), -4.2 (CH_3, TBDMS).

HRMS (ES+): calcd $C_{19}H_{25}N_4O_2Si$ 369.1747 $[M+H]^+$, found 369.1771 $[M+H]^+$.

FT-IR (ATR): ν_{max} 3350, 2928, 2113, 1641, 1514, 1277, 918 and 836 cm^{-1}.

^{1}H-NMR (400 MHz, $CDCl_3$) Compound 7.

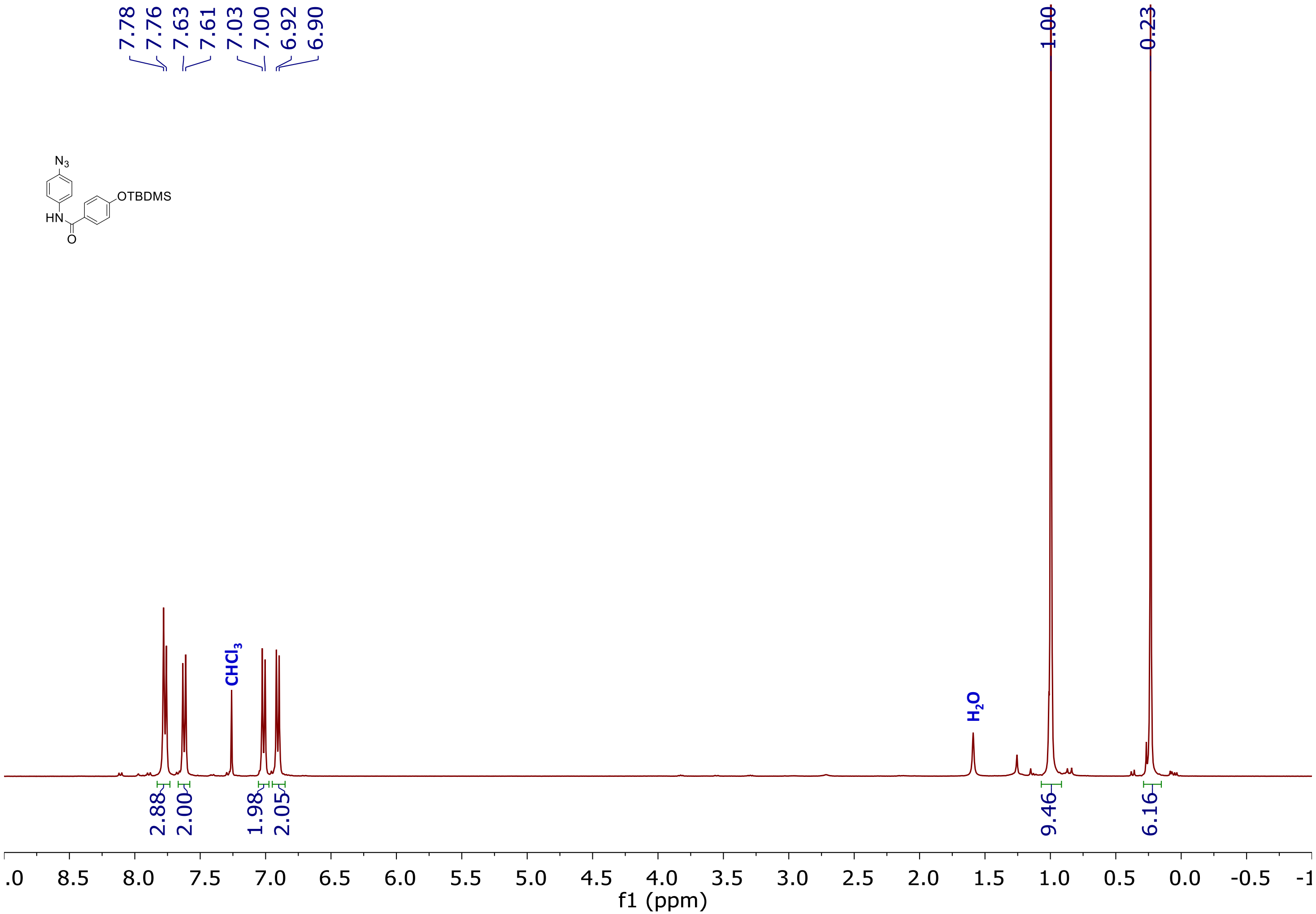

^{13}C-NMR (100.6 MHz, $CDCl_3$) Compound 7.

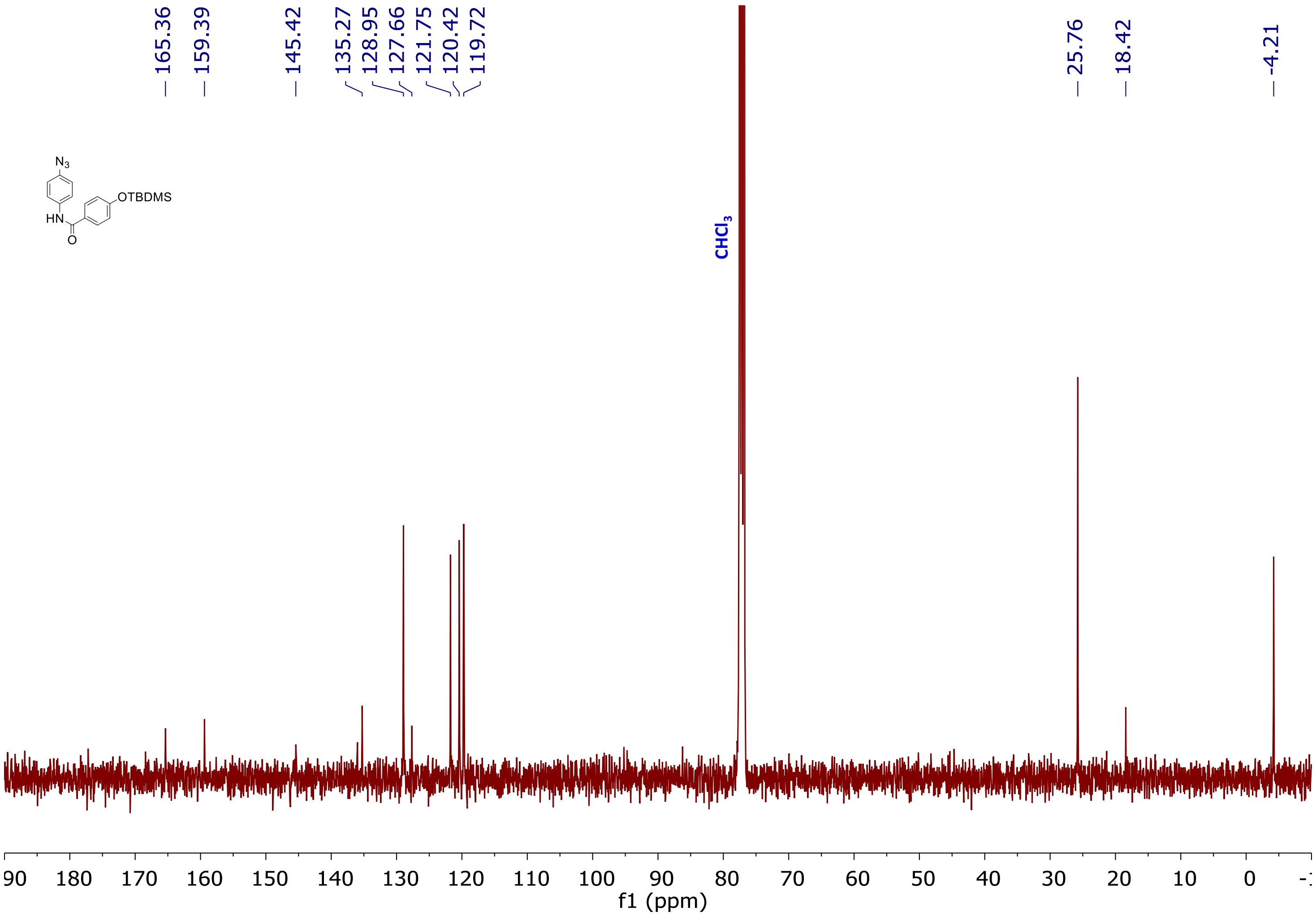

Compound 8.

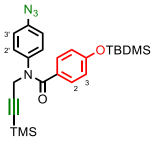

7 (0.252 g, 0.68 mmol) was dissolved in dry THF (3 mL) and added to a suspension of NaH (60% dispersion in mineral oil, 0.082 g, 2.05 mmol) in THF (2 mL) under inert atmosphere at 0 °C. The reaction was allowed to reach room temperature for 15 min. 3-Bromo-1-(trimethylsilyl)-1-propyne (0.335 mL, 2.05 mmol) was added dropwise and the reaction was vigorously stirred overnight. Satd. aq. NH_4Cl was carefully added at 0 °C and the reaction was extracted with EtOAc (3x). The combined organic phase was washed with brine (1x), dried with anhydrous $MgSO_4$, filtered, and the solvents evaporated. The obtained residue was purified by flash chromatography (from 0% to 25% of EtOAc in Pet. Ether) to afford **8** (0.235 g, 72%) as a yellow oil.

^{1}H NMR (400 MHz, $CDCl_3$): δ_H = 7.23 (d, 2H, *J* = 7.0 Hz, 2-H), 7.11 (d, 2H, *J* = 7.0 Hz, 2'-H), 6.90 (d, 2H, *J* = 8.5 Hz, 3'-H), 6.64 (d, 2H, *J* = 8.5 Hz, 3-H), 4.65 (s, 2H, N-CH_2), 0.93 (s, 9H, ^{t}Bu, TBDMS), 0.14 (s, 6H, CH_3, TBDMS), 0.12 (s, 9H, TMS).

^{13}C NMR (100.6 MHz, $CDCl_3$): δ_C = 169.9 (CO), 157.5 (4-C), 140.1 and 138.9 (1'-C and 4'-C), 130.9, 130.9, 129.5 and 128.1 (2-C, 2'-C and 3'-C), 119.6 (3-C), 101.0 (C-TMS), 89.8 (C, alkyne), 40.7 (N-CH_2), 25.7 (^{t}Bu, TBDMS), 18.4 (C, TBDMS), -0.1 (CH_3, TMS), -4.3 (CH_3, TBDMS).

HRMS (ES+): calcd for $C_{25}H_{35}N_4O_2Si_2$ 479.2299 $[M+H]^+$, found 479.2303 $[M+H]^+$.

FT-IR (ATR): ν_{max} 2957, 2930, 2858, 2124, 2094, 1651, 1506, 1265, 911 and 840 cm^{-1}.

^{1}H-NMR (400 MHz, $CDCl_3$) Compound 8.

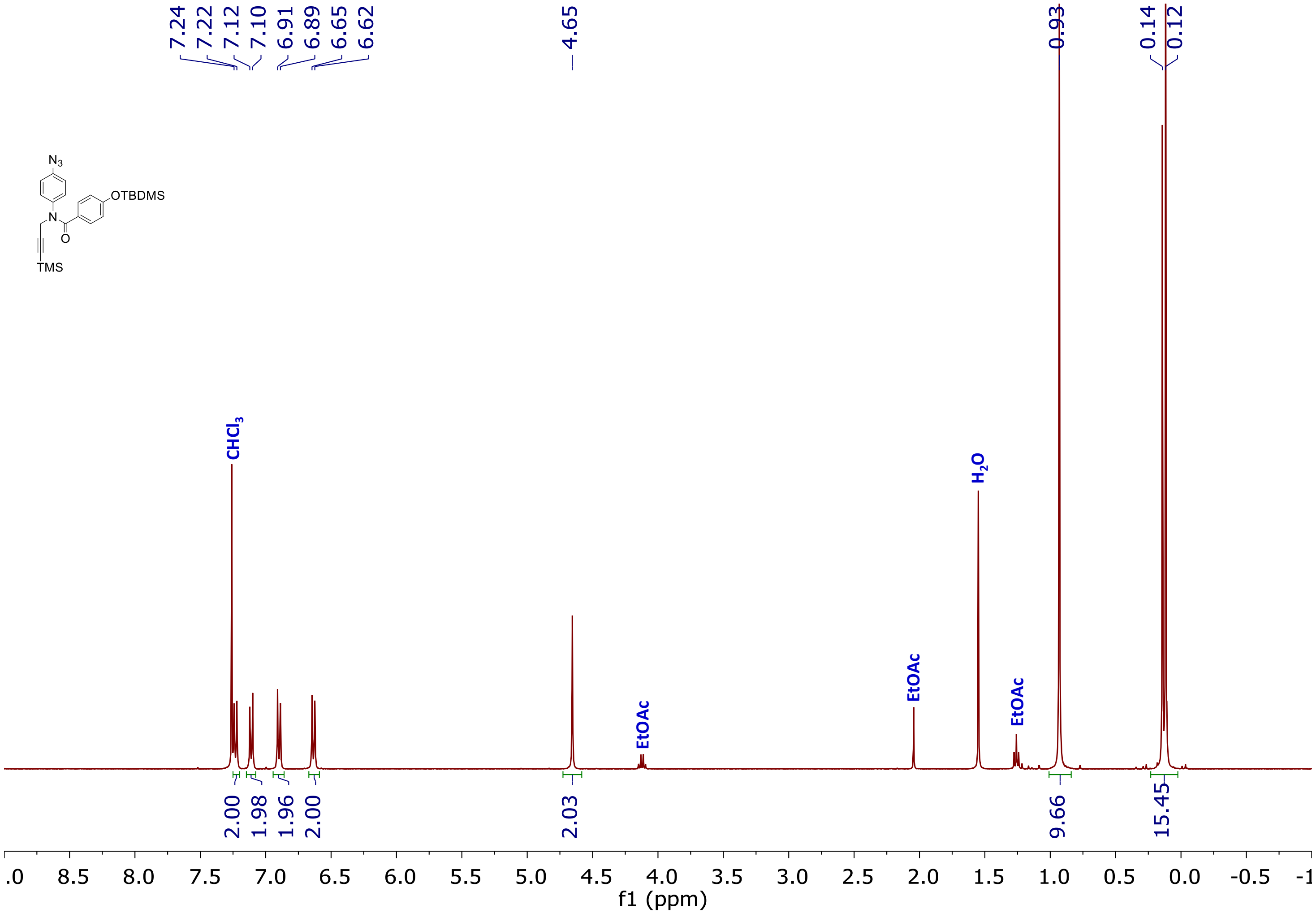

^{13}C-NMR (100.6 MHz, $CDCl_3$) Compound 8.

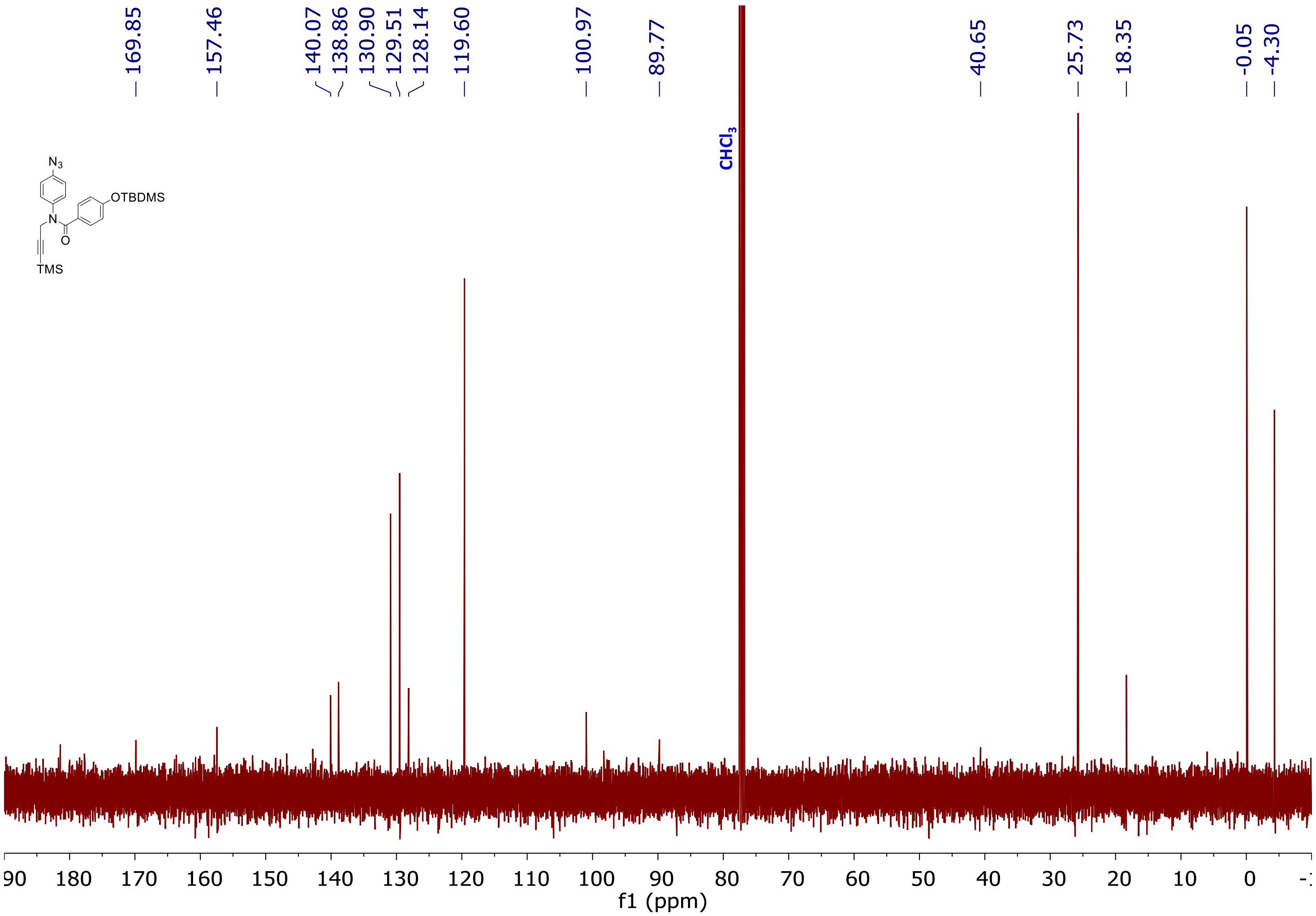

Compound 9

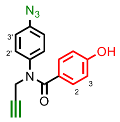

A solution of **8** (0.071 g, 0.15 mmol) in dry THF (2 mL) was treated with TBAF solution (1M in THF, 0.298 mL, 0.30 mmol). The reaction was stirred for 10 minutes at room temperature and quenched with 5% soln. HCl and extracted with EtOAc (3x) followed by washing with H_2O and brine The organic layer was dried over anhydrous $MgSO_4$ and concentrate under vacuum. The crude was repetitively dissolved in $CHCl_3$ and evaporated, and dried under high vacuum to remove traces of TBDMS-F. Compound **9** was obtained as a foam (0.044 g, quantitative).

^{1}H NMR (400 MHz, $CDCl_3$): δ_H = 7.26 (d, 2H, *J* = 8.5 Hz, 3-H), 7.12 (d, 2H, *J* = 8.5 Hz, 2-H), 6.93 (d, 2H, *J* = 8.5 Hz, 2'-H), 6.62 (d, 2H, *J* = 8.5 Hz, 3'-H), 5.24 (s, 1H, OH), 4.63 (d, 2H, *J* = 2.5 Hz, N-CH_2), 2.24 (t, 1H, *J* = 2.5 Hz, CH, alkyne).

^{13}C NMR (100.6 MHz, $CDCl_3$): δ_C = 170.7 (CO), 158.4 (4-C), 139.8 and 139.1 (1'-C and 4'-C), 131.2 and 129.1 (2-C and 2'-C), 126.1 (1-C), 119.9 (3'-C), 115.1 (3-C), 78.9 (C, alkyne), 72.7 (CH, alkyne), 40.3 (N-CH_2).

HRMS (ES+): calcd for $C_{16}H_{13}N_4O_2$ 293.1039 $[M+H]^+$, found 293.1056 $[M+H]^+$.

FT-IR (ATR): ν_{max} 3293, 2123, 2093, 1606, 1505, 1276, 1223, 1170 and 751 cm^{-1}.

^{1}H-NMR (400 MHz, $CDCl_3$) Compound 8.

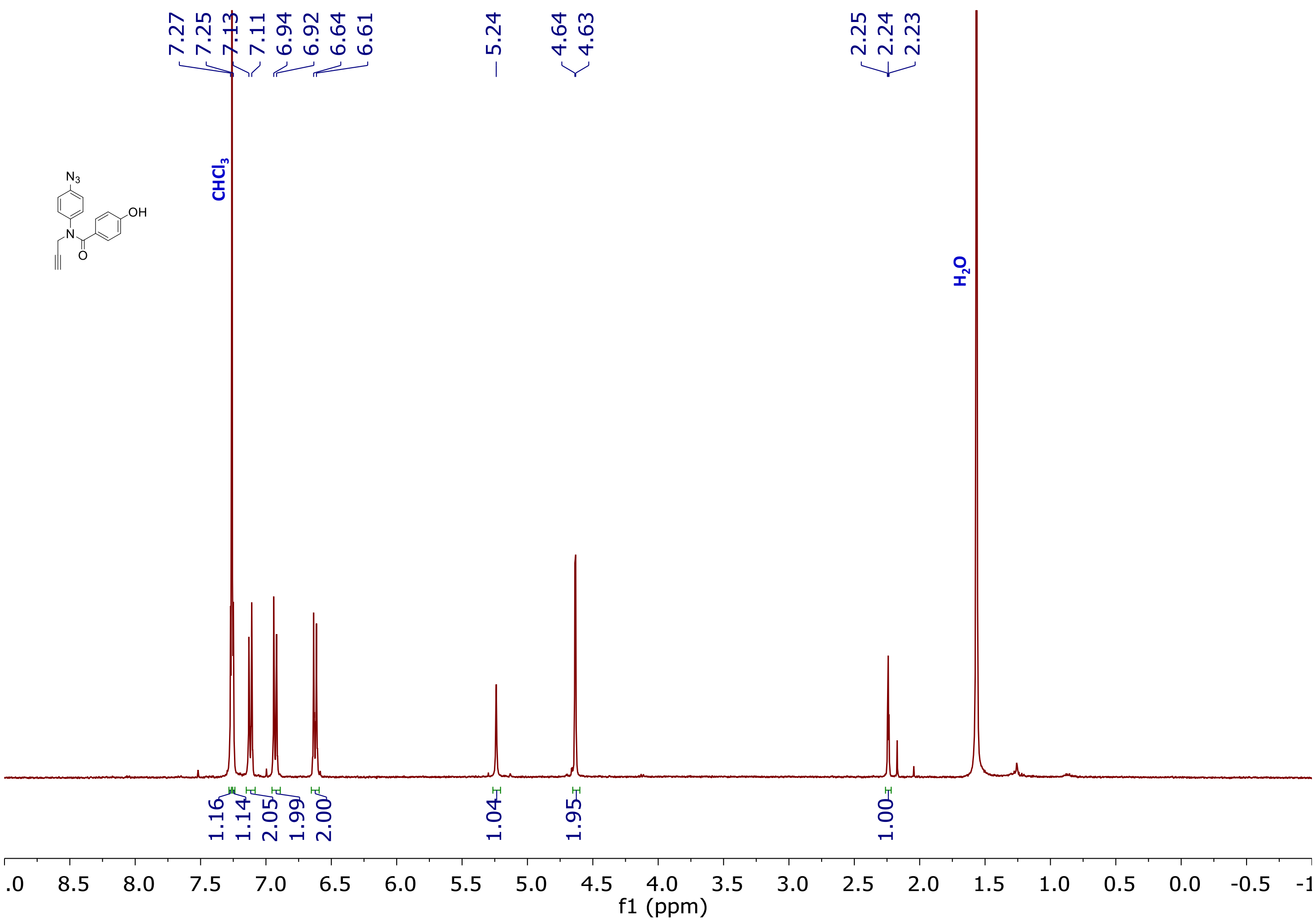

^{13}C-NMR (100.6 MHz, $CDCl_3$) Compound 8.

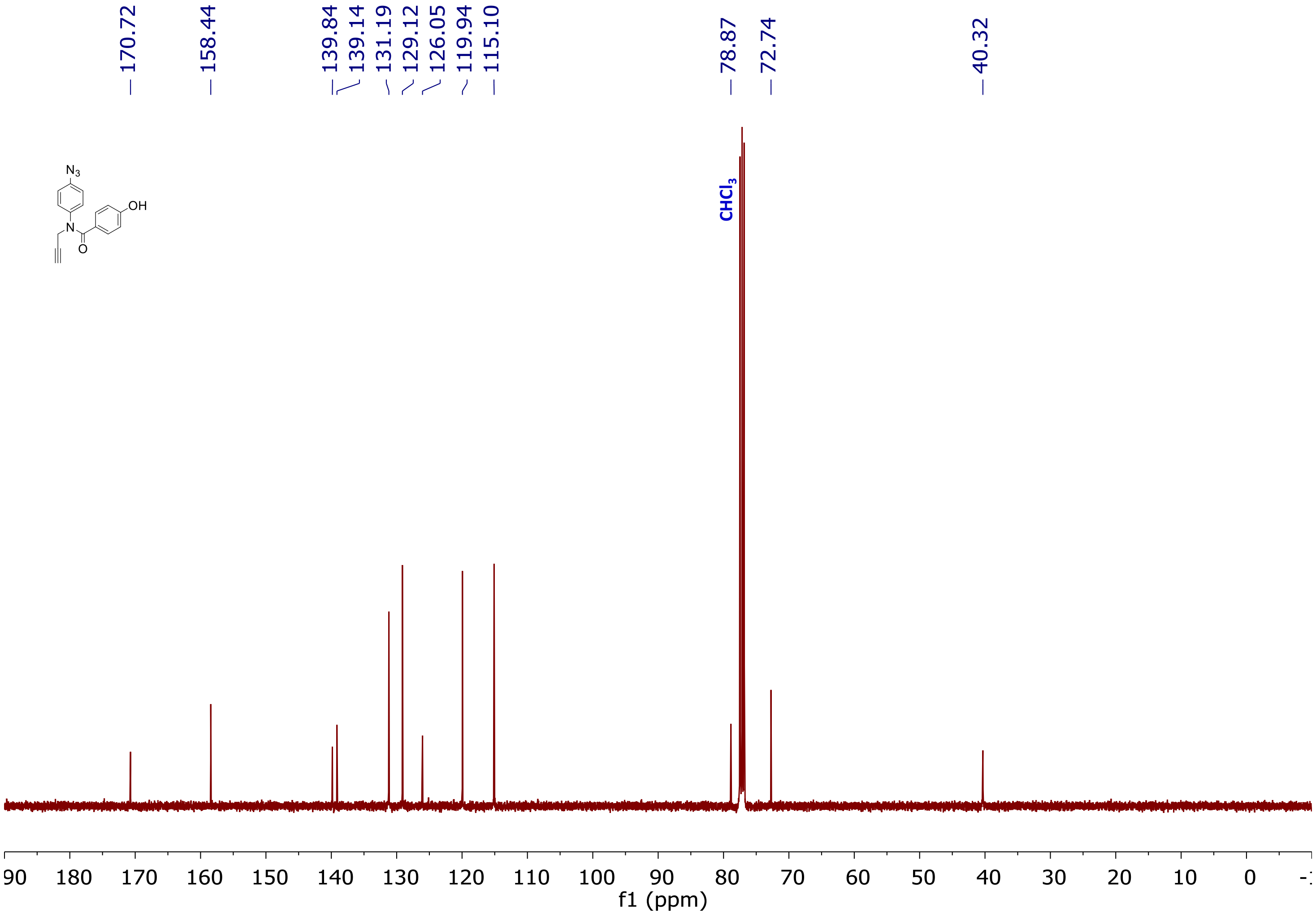

Synthesis of AAP template[S2]

The synthesis of mixed templates **13** (AAP) is highlighted in Scheme S8. CuAAC capping of **8** with phenyl propargyl ether followed by TBAF-mediated deprotection gave access to capped phenol monomer **10** in good yield. CuAAC coupling of **10** with **4** gave access to mixed 2-mer **11** in excellent yield. From **11**, subsequent CuAAC coupling with **4** afforded 3-mer **12** in moderate yield. Subsequent CuAAC capping of **12** with *p*-*tert*-butylbenzylazide and LiOH-mediated basic hydrolysis yielded AAP template **13** in good yield.

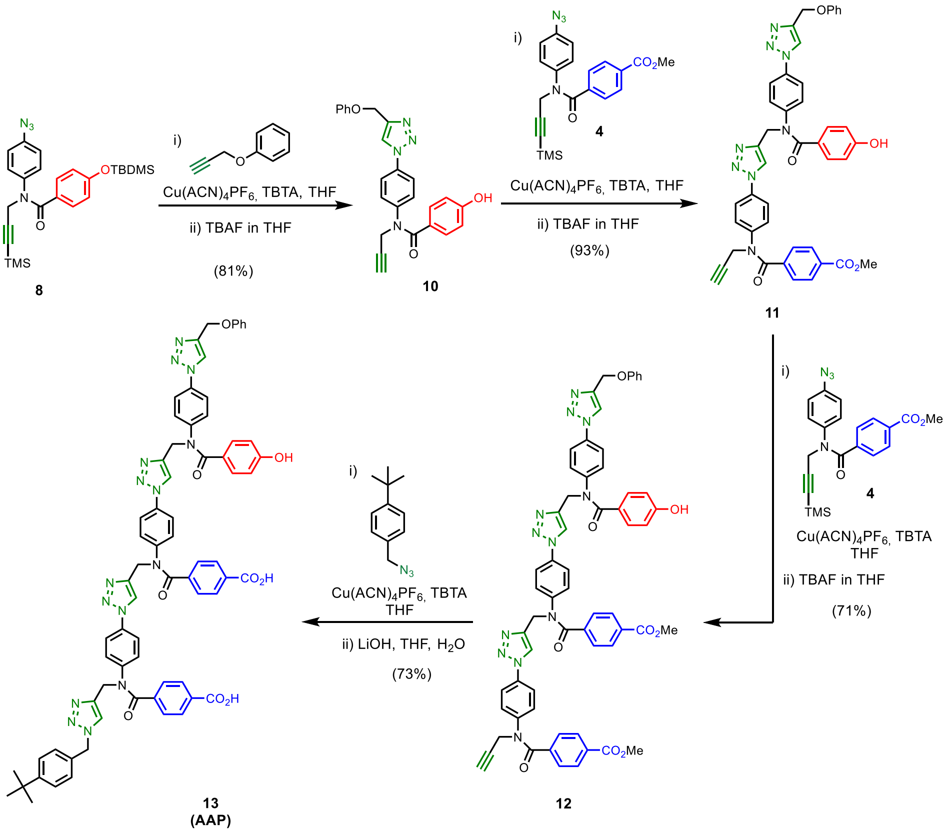

Scheme S8

Compound 10.

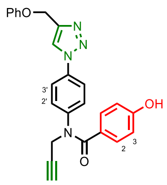

8 (0.320 g, 0.67 mmol), $Cu(CH_3CN)_4PF_6$ (0.025 g, 0.07 mmol) and TBTA (0.036 g, 0.07 mmol) were mixed in a round-bottom flask and, under N_2, THF (5 mL) was added. Phenyl propargyl ether (0.170 mL, 1.34 mmol) was added and the reaction was stirred overnight at room temperature. Once the reaction was completed, TBAF (1M in THF, 1.34 mL, 1.34 mmol) was added dropwise. The solution was stirred for 10 min at room temperature, quenched with 0.1M HCl soln. and extracted with EtOAc (3x) followed by washing with H_2O and brine. The organic layer was dried over anhydrous $MgSO_4$ and concentrate under vacuum. The crude was purified by flash column chromatography on silica gel (gradient from 0% to 50% of EtOAc in Pet. Ether) to afford **10** (0.229 g, 81%) as a foam.

^{1}H NMR (400 MHz, $CDCl_3$): δ_H = 8.46 (s, 1H, OH), 8.03 (s, 1H, CH_{triaz}), 7.62 (d, 2H, J = 9.0 Hz, 3'-H), 7.27 (m, 4H, 2'-H and 3''-H, PhO), 7.20 (d, 2H, J = 8.5 Hz, 2-H), 6.97 (t partially overlapped, J = 7.0 Hz, 4''-H, PhO), 6.96 (d, 2H, J = 8.5 Hz, 2''-H, PhO), 6.64 (d, 2H J = 8.5 Hz, 3-H), 5.23 (s, 2H, O-CH_2), 4.66 (d, 2H, J = 2.5 Hz, N-CH_2), 2.25 (t, 1H, J = 2.5 Hz, alkyne).

^{13}C NMR (100.6 MHz, $CDCl_3$): δ_C = 170.6 (CO), 159.2 (4-C), 158.1 (1''-C, PhO), 145.3 (C_{triaz}), 143.8 (1'-C), 135.1 (4'-C), 131.3 (2-C), 129.7 (3''-C, PhO), 128.9 (2'-C), 125.4 (1-C), 121.6 (4''-C, PhO), 121.4 (3'-C), 121.2 (CH_{triaz}), 115.3 (3-C), 114.8 (2''-C, PhO), 78.7 (C, alkyne), 73.0 (CH, alkyne), 61.7 (O-CH_2), 40.1 (N-CH_2).

HRMS (ES+): calcd for $C_{25}H_{21}N_4O_3$ 425.1614 $[M+H]^+$, found 425.1597 $[M+H]^+$.

FT-IR (ATR): ν_{max} 3286, 1634, 1604, 1518, 1280, 1234, 846 and 756 cm^{-1}.

^{1}H-NMR (400 MHz, $CDCl_3$) Compound 10.

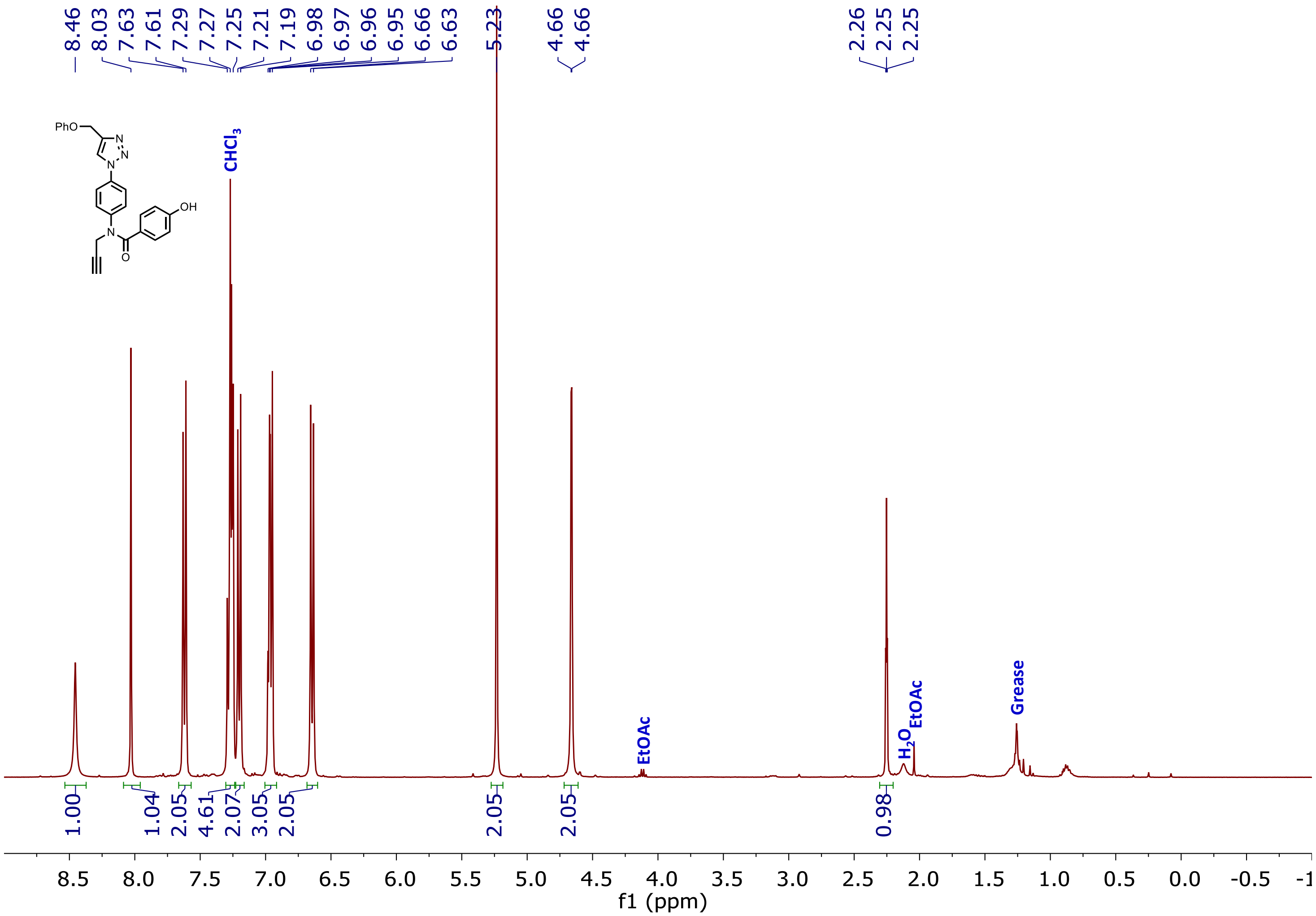

^{13}C-NMR (100.6 MHz, CDCl$_3$) Compound 10.

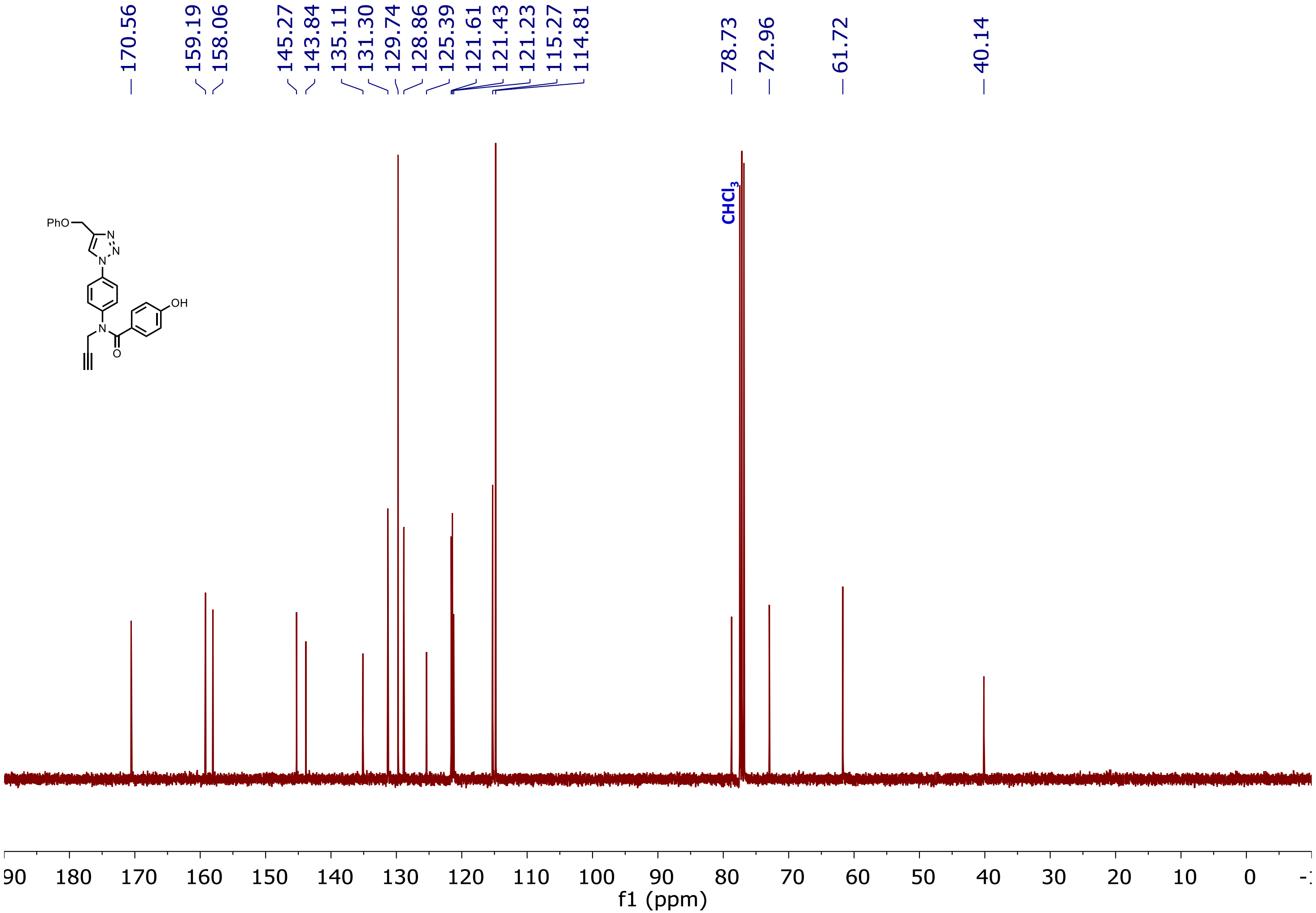

Compound 11.

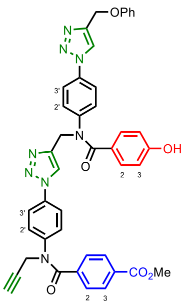

Compound **10** (0.100 g, 0.24 mmol), compound **4** (0.096 g, 0.24 mmol), $Cu(CH_3CN)_4PF_6$ (0.009 g, 0.02 mmol) and TBTA (0.013 g, 0.02 mmol) were mixed in a round-bottom flask and, under N_2, THF (4 mL) was added. The reaction was stirred overnight at room temperature. Once the reaction was completed, TBAF (1M in THF, 0.240 mL, 0.24 mmol) was added dropwise. The solution was stirred for 10 min at room temperature, quenched with 5% soln. HCl and extracted with EtOAc (3x) followed by washing with H_2O and brine. The organic layer was dried over anhydrous $MgSO_4$ and concentrate under vacuum. The crude was purified by flash column chromatography on silica gel (gradient from 0% to 80% of EtOAc in Pet. Ether) to afford **11** (0.166 g, 93%) as a foam.

^{1}H NMR (400 MHz, CDCl$_3$): δ_H = 8.22 (s, 1H, OH), 8.22 (s, 1H, CH_{triaz}), 7.99 (s, 1H, CH_{triaz}), 7.86 (d, 2H, *J* = 8.5 Hz, 3-H, ester), 7.62 (d, 2H, *J* = 9.0 Hz, 3'-H), 7.55 (d, 2H, *J* = 9.0 Hz, 3'-H), 7.40 (d, 2H, *J* = 8.5 Hz, 2-H, ester), 7.29-7.24 (m, 6H, 2'-H and 3''-H, PhO), 7.14 (d, 2H, *J* = 8.5 Hz, 2-H, phenol), 6.96 (m, 3H, 2''-H and 4''-H, PhO), 6.59 (d, 2H *J* = 8.5 Hz, 3-H, phenol), 5.23 (s, 2H, O-CH_2), 5.16 (s, 2H, N-CH_2, phenol), 4.69 (d, 2H, *J* = 2.5 Hz, N-CH_2, ester), 3.83 (s, 3H, O-CH_3), 2.29 (t, 1H, *J* = 2.5 Hz, alkyne).

^{13}C NMR (100.6 MHz, CDCl$_3$): δ_C = 170.4 (CO, amide phenol), 169.4 (CO, amide ester), 166.3 (CO, ester), 159.1 (4-C, phenol), 158.1 (1''-C, PhO), 145.3 (C_{triaz}), 144.9 (1'-C), 144.7 (C_{triaz}), 142.5 (1'-C), 139.0 (1-C, ester), 135.7 (4'-C), 134.8 (4'-C), 131.6 (4-C, ester), 131.4 (2-C, phenol), 129.7, 129.5, 129.1, 128.7 and 128.5 (2-C and 3-C, ester; 2'-C; 3''-C, PhO), 125.8 (1-C, phenol), 122.2 (CH_{triaz}), 121.6 (4''-C, PhO), 121.5 (3'-C), 121.2 (3'-C), 121.2 (CH_{triaz}), 115.2 (3-C, phenol), 114.8 (2''-C, PhO), 78.3 (C, alkyne), 73.3 (CH, alkyne), 61.8 (O-CH_2), 52.5 (O-CH_3), 46.5 (N-CH_2, phenol), 39.8 (N-CH_2, ester).

HRMS (ES+): calcd for $C_{43}H_{35}N_8O_6$ 759.2680 $[M+H]^+$, found 759.2689 $[M+H]^+$.

FT-IR (ATR): ν_{max} 2954, 2856, 1271, 1642, 1604, 1518, 1279, 1235, 845 and 753 cm^{-1}.

^{1}H-NMR (400 MHz, $CDCl_3$) Compound 11.

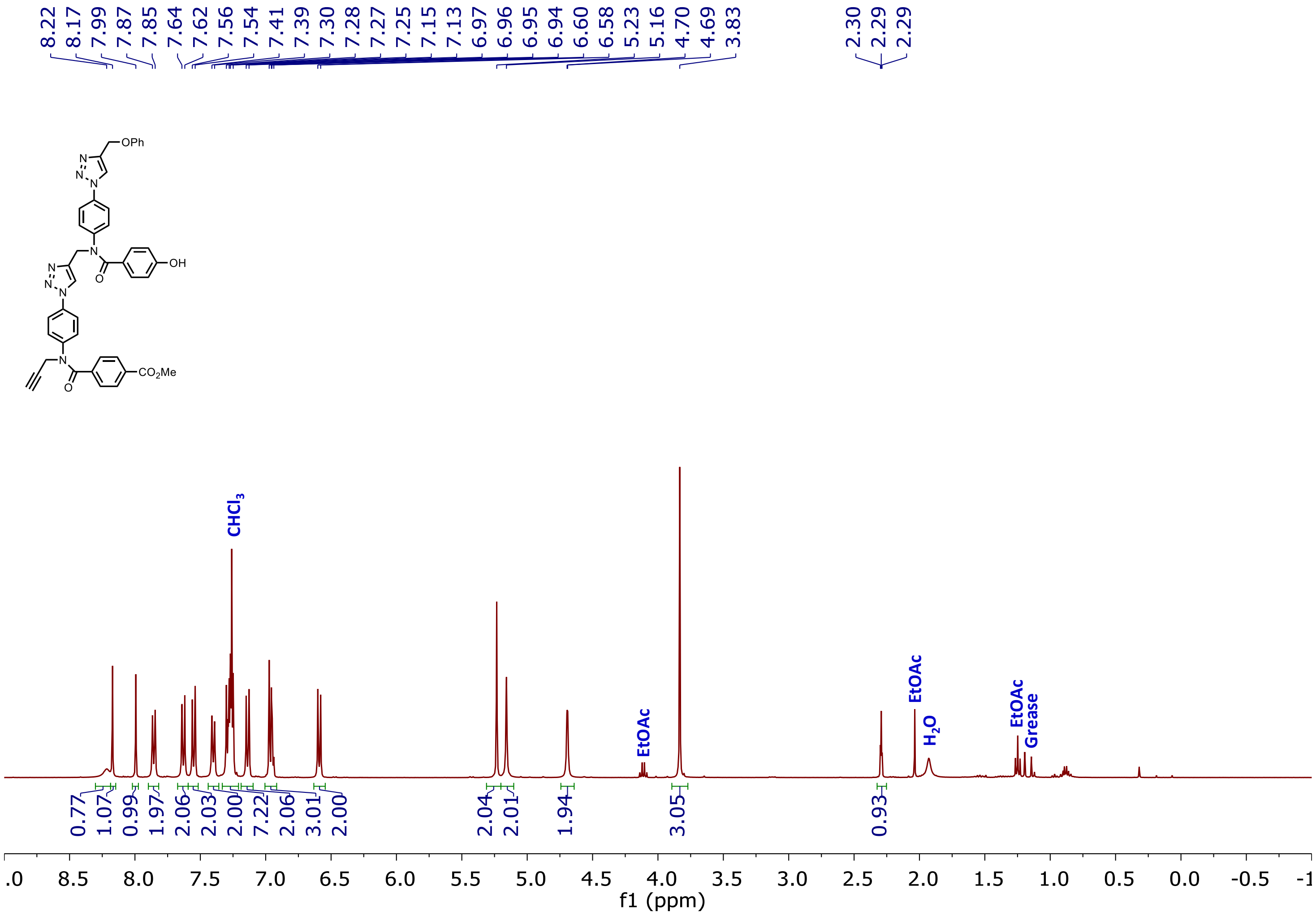

^{13}C-NMR (100.6 MHz, $CDCl_3$) Compound 11.

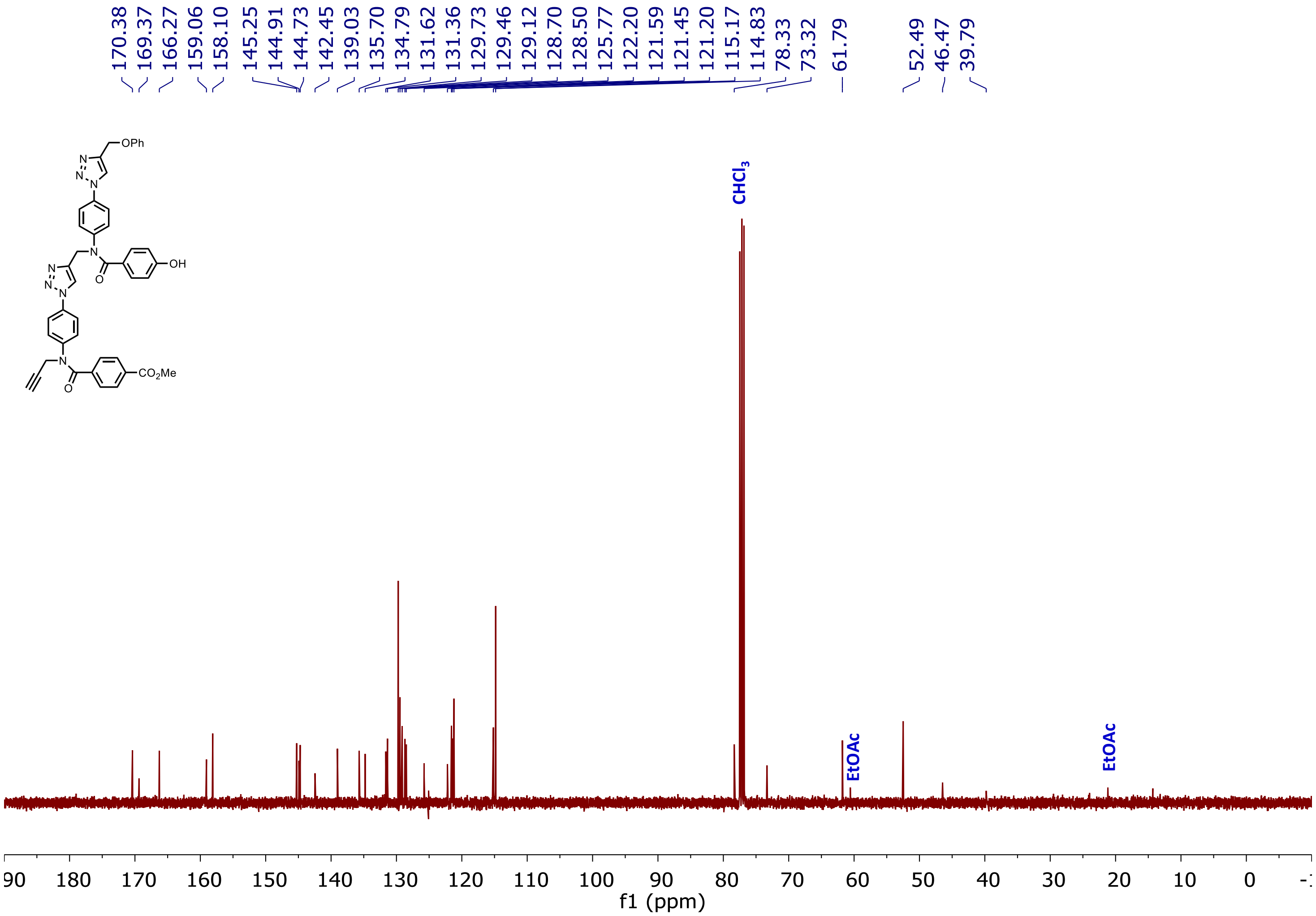

Compound 12.

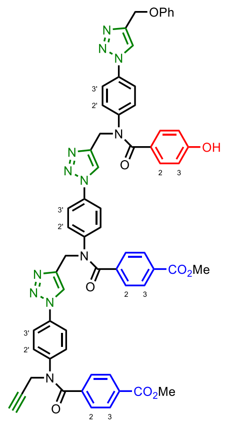

11 (0.082 g, 0.11 mmol), **4** (0.043 g, 0.11 mmol), $Cu(CH_3CN)_4PF_6$ (0.004 g, 0.01 mmol) and TBTA (0.006 g, 0.01 mmol) were mixed in a round-bottom flask and, under N_2, THF (3 mL) was added. The reaction was stirred overnight at room temperature. Once the reaction was completed, TBAF (1M in THF, 0.110 mL, 0.11 mmol) was added dropwise. The solution was stirred for 10 min at room temperature, quenched with 0.1M HCl soln. and extracted with EtOAc (3x) followed by washing with H_2O and brine. The organic layer was dried over anhydrous $MgSO_4$ and concentrate under vacuum. The crude was purified by flash column chromatography on silica gel (gradient from 20% to 100% of EtOAc in Pet. Ether) to afford **12** (0.082 g, 71%) as a foam.

^{1}H NMR (500 MHz, $CDCl_3$): δ_H = 8.18 (s, 1H, $CH_{triaz,}$), 8.16 (s, 1H, CH_{triaz}), 8.00 (s, 1H, CH_{triaz}), 7.88 (d, 2H, *J* = 8.5 Hz, 3-H, ester), 7.84 (d, 2H, *J* = 8.5 Hz, 3-H, ester), 7.67 (d, 2H, *J* = 9.0 Hz, 3'-H), 7.61 (d, 2H, *J* = 9.0 Hz, 3'-H), 7.59 (d, 2H, *J* = 9.0 Hz, 3'-H), 7.42 (d, 2H, *J* = 8.5 Hz, 2-H, ester), 7.37-7.27 (m, 10H, 2-H, ester, 2'-H and 3''-H, PhO), 7.18 (d, 2H, *J* = 9.0 Hz, 2-H, phenol), 6.98 (m, 3H, 2''-H and 4''-H, PhO), 6.86 (s, 1H, OH), 6.60 (d, 2H, *J* = 9.0 Hz, 3-H, phenol), 5.27 (s, 2H, O-CH_2), 5.20 (s, 2H, N-CH_2), 5.18 (s, 2H, N-CH_2), 4.72 (d, 2H, *J* = 2.5 Hz, N-CH_2, alkyne), 3.86 (s, 3H, O-CH_3), 3.84 (s, 3H, O-CH_3), 2.30 (t, 1H, *J* = 2.5 Hz, alkyne).

^{13}C NMR (125 MHz, $CDCl_3$): δ_C = 170.2 (CO, amide phenol), 169.6 and 169.4 (CO, amide ester), 166.3 and 166.2 (CO, ester), 158.5 (4-C, phenol), 158.2 (1''-C, PhO), 145.4 and 145.0 (C_{triaz}), 144.9 (1'-C), 144.4 (C_{triaz}), 143.6 and 142.7 (1'-C), 139.2 and 139.1 (1-C, ester), 135.7 and 135.5 (4'-C, internal), 134.9 (4'-C), 131.8 and 131.7 (4-C, ester), 131.4 (2-C, phenol), 129.8, 129.5, 129.2, 128.9, 128.8, 128.5 and 126.5 (2-C and 3-C, ester; 2'-C; 3''-C, PhO), 126.5 (1-C, phenol), 122.2 and 122.2 (CH_{triaz}, internal), 121.6 (4''-C, PhO), 121.5 (3'-C), 121.3 and 121.3 (3'-C, internal), 121.1 (CH_{triaz}), 115.2 (3-C, phenol), 114.9 (2''-C, PhO), 78.4 (C, alkyne), 73.3 (CH, alkyne), 62.0 (O-CH_2), 52.5 (O-CH_3), 46.5 (N-CH_2), 46.3 (N-CH_2), 39.8 (N-CH_2, alkyne).

HRMS (ES+): calcd for $C_{61}H_{48}N_{12}O_9Na$ 1116.3598 $[M+H]^+$, found 1116.0592 $[M+H]^+$.

FT-IR (ATR): ν_{max} 3140, 1722, 1646, 1518, 1279, 1238, 846 and 755 cm^{-1}.

^{1}H-NMR (500 MHz, $CDCl_3$) Compound 12.

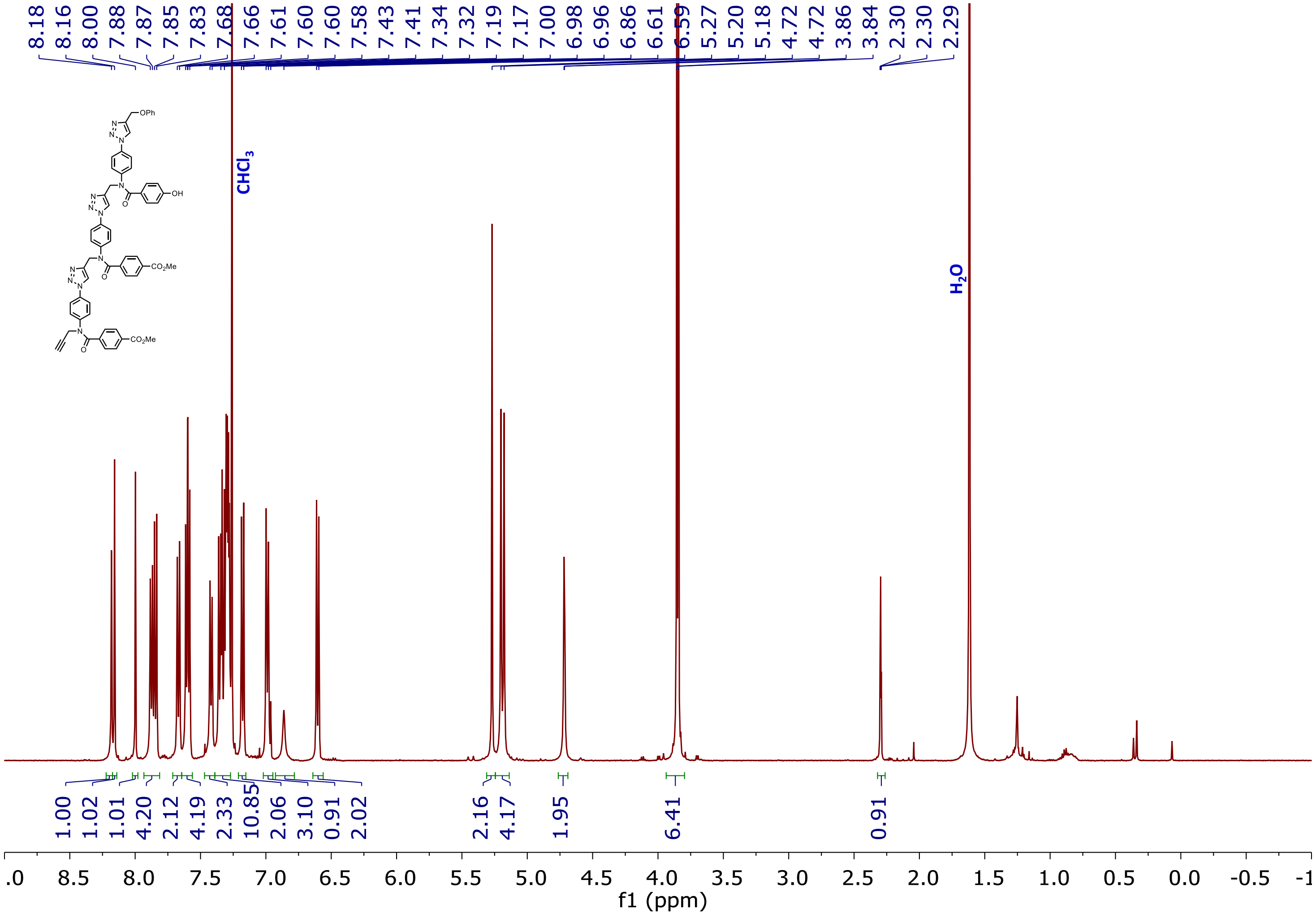

^{13}C-NMR (125MHz, $CDCl_3$) Compound 12.

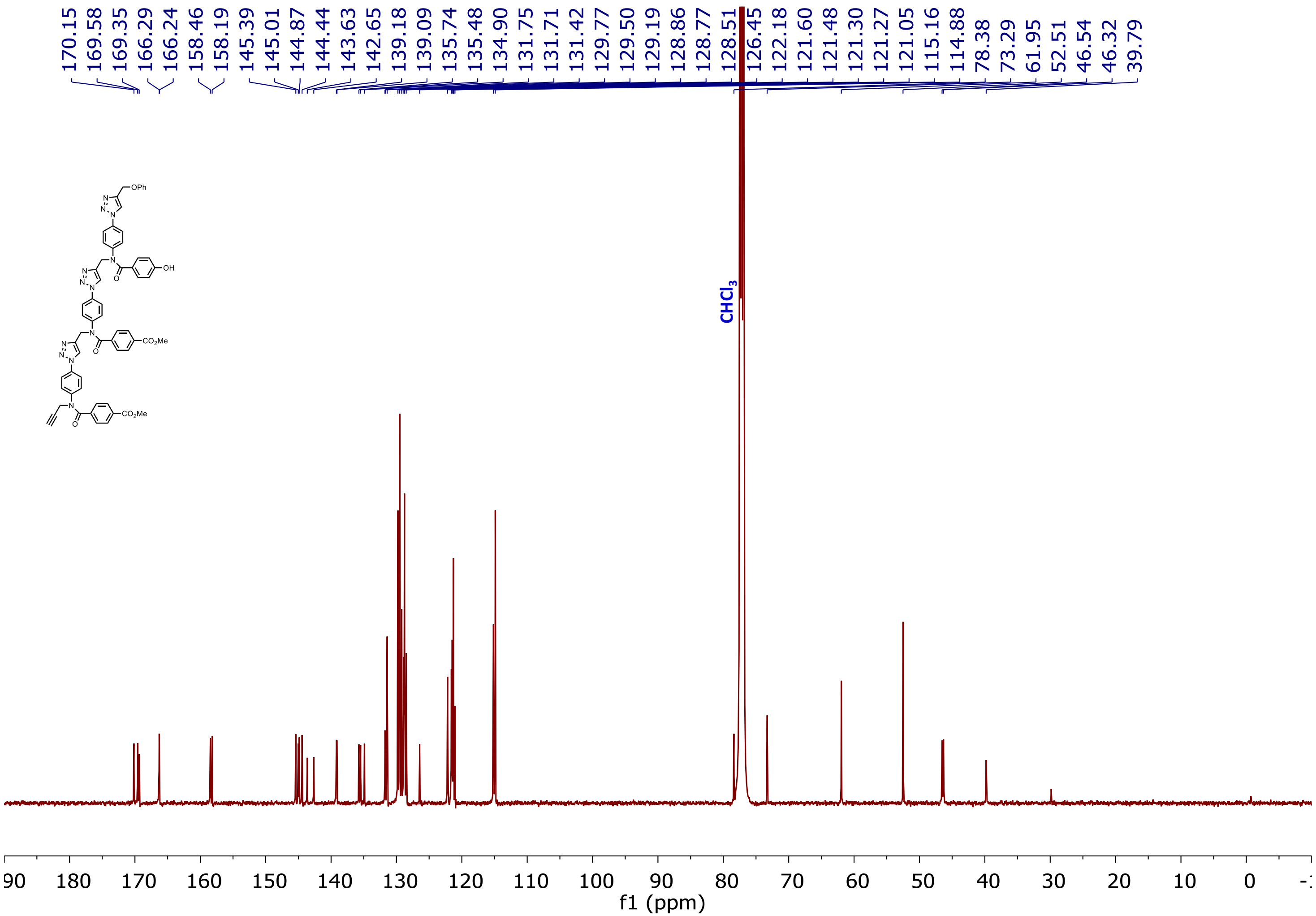

Compound 13 (AAP template).

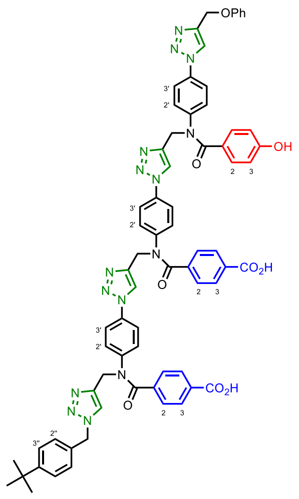

12 (0.076 g, 0.07 mmol), 1-(azidomethyl)-4-*tert*-butylbenzene (0.026 g, 0.14 mmol), $Cu(CH_3CN)_4PF_6$ (0.005 g, 0.01 mmol) and TBTA (0.007 g, 0.01 mmol) were mixed in a round-bottom flask and, under N_2, THF (2 mL) was added. The reaction was stirred overnight at room temperature. The reaction was then diluted with EtOAc and washed with 0.02M EDTA soln. (2x), H_2O (1x) and brine. The organic layer was dried over $MgSO_4$ and concentrate under vacuum. The crude was dissolved in THF:H_2O 3:1 (2 mL) and LiOH (0.017 g, 0.41 mmol) was added. The reaction was stirred at room temperature for 2h. Then, the crude was diluted with H_2O and acidified with 1M HCl soln. to pH 2-3. The aqueous phase was extracted with EtOAc (3x) and the combined organic phase was washed with brine, dried over $MgSO_4$ and evaporated to dryness. The residue was purified by flash chromatography on silica gel (gradient from 0% to 10% of MeOH in CH_2Cl_2) to afford **13** (0.063 g, 73%) as a white solid.

mp: 182-184 °C.

^{1}H NMR (500 MHz, DMSO-*d*$_6$): δ_H = 8.86 (s, 1H, CH_{triaz}), 8.72 (s, 1H, CH_{triaz}), 8.65 (s, 1H, CH_{triaz}), 8.09 (s, 1H, CH_{triaz}), 7.76 (m, 10H, 3-H, acid; 3'-H), 7.42 (m, 6H, 2-H and 2'-H), 7.38 (d, 2H, *J* = 9.0 Hz, 2'-H), 7.33 (d, 2H, *J* = 8.5 Hz, 2-H, acid), 7.29 (m, 4H, 3''-H, PhO; 3''-H, ^{t}BuPh), 7.19 (d, 2H, *J* = 9.0 Hz, 2-H, phenol), 7.07 (d, 2H, *J* = 8.5 Hz, 2''-H, ^{t}BuPh), 7.04 (m, 2H, 2''-H, PhO), 6.95 (t, 1H, *J* = 7.5 Hz, 4''-H, PhO), 6.59 (d, 2H, *J* = 8.5 Hz, 3-H, phenol), 5.48 (s, 2H, N-CH_2), 5.18 (m, 4H, N-CH_2), 5.15 (m, 4H, N-CH_2), 1.16 (s, 9H, ^{t}Bu).

^{13}C NMR (125 MHz, DMSO-*d*$_6$): δ_C = 169.4, 168.9 and 168.9 (CO, amide), 167.1 (CO, acid), 159.3 (4-C, phenol), 158.0 (1''-C), 150.6 (4''-C, ^{t}BuPh), 144.8, 144.3 and 144.0 (C_{triaz}), 143.9 (1'-C), 143.0 (C_{triaz}), 142.5 and 142.4 (1'-C), 139.3 and 139.1 (1-C, acid), 134.8, 134.6 and 134.2 (4'-C), 133.2 (4-C, acid), 131.0 (2-C, phenol), 129.6, 129.3, 128.9, 128.8, 128.3 and 127.4 (C_{arom}), 125.6 (1-C, phenol), 125.4 (3''-C, ^{t}BPh), 124.2, 122.8, 121.6 and 121.6 (CH_{triaz}), 121.0 (4''-C, PhO), 120.6, 120.5 and 120.4 (3'-C), 114.8 (2''-C, PhO), 114.6 (3-C, phenol), 60.9 (O-CH_2), 52.5, 45.5, 45.2 and 44.9 (N-CH_2), 34. 2 (C, ^{t}Bu), 31.0 (CH_3, ^{t}Bu).

HRMS (ES+): calcd for $C_{70}H_{60}N_{15}O_9$ 1254.4698 $[M+H]^+$, found 1254.4014 $[M+H]^+$.

FT-IR (ATR): ν_{max} 2957, 2921, 2850, 1644, 1600, 1518, 1385, 1240, 846 and 756 cm^{-1}.

^{1}H-NMR (500 MHz, DMSO-d_6) Compound 13 (AAP template).

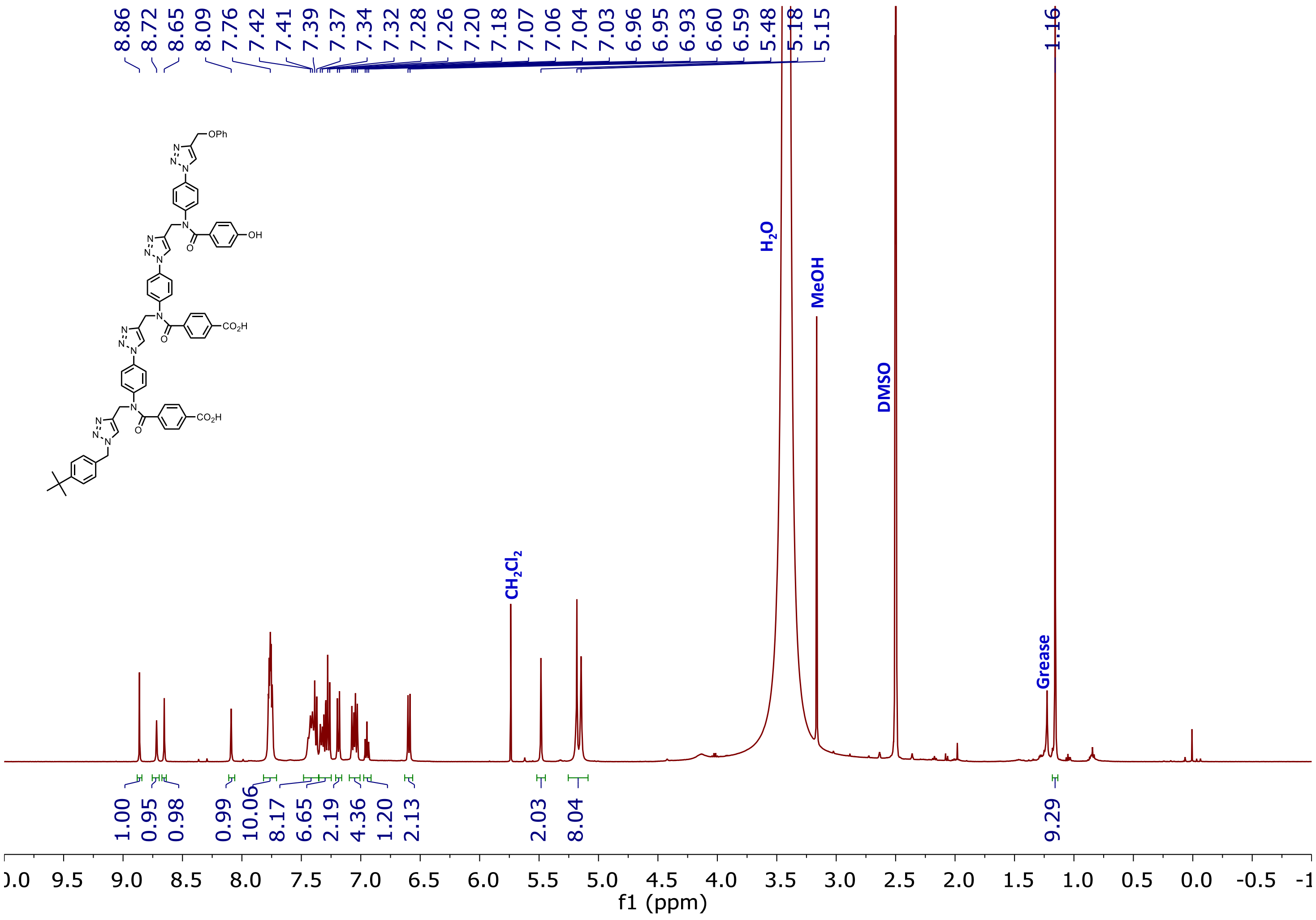

^{13}C-NMR (125MHz, DMSO-d_6) Compound 13 (AAP template).

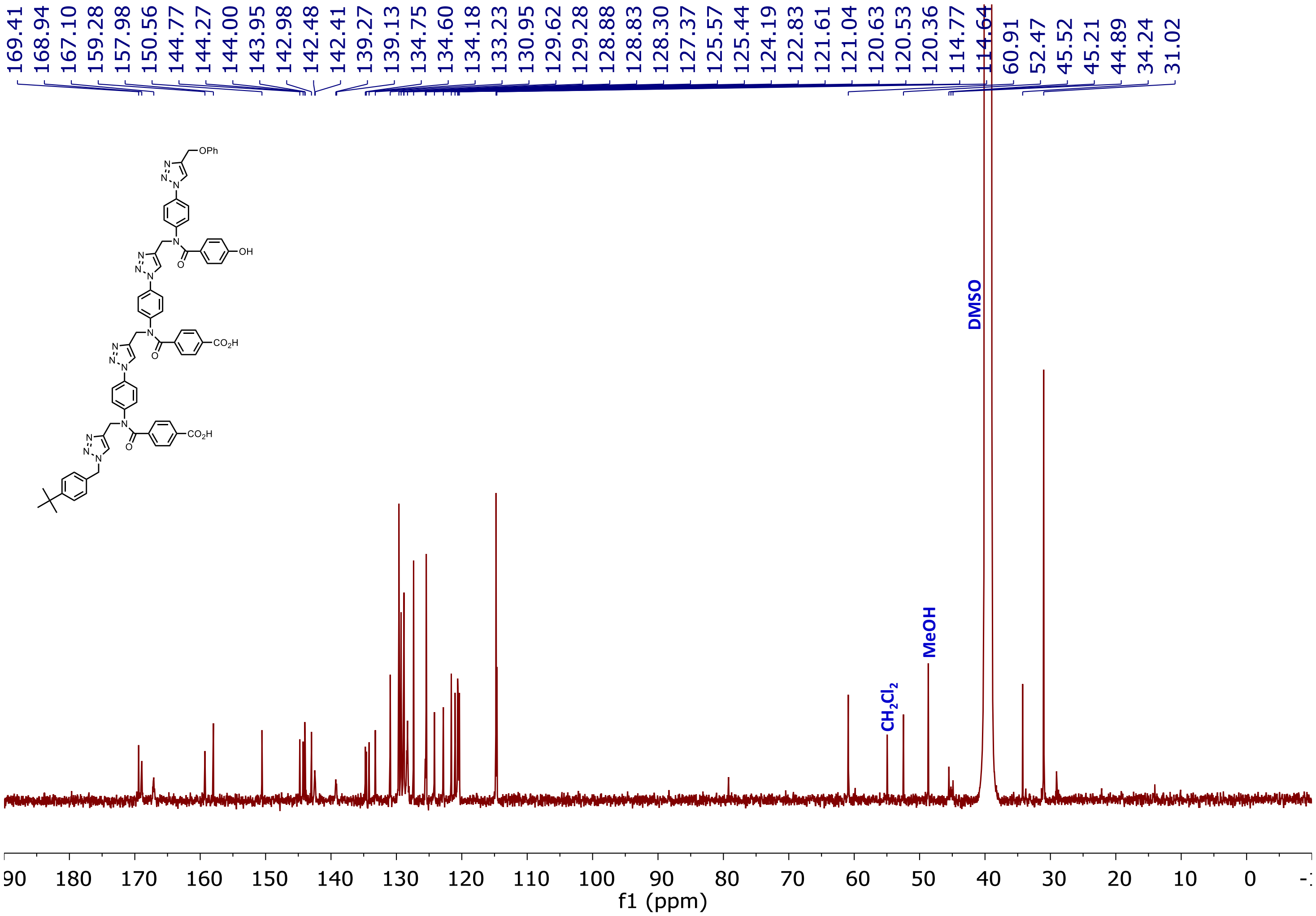

COSY (DMSO-d_6) Compound 13 (AAP template).

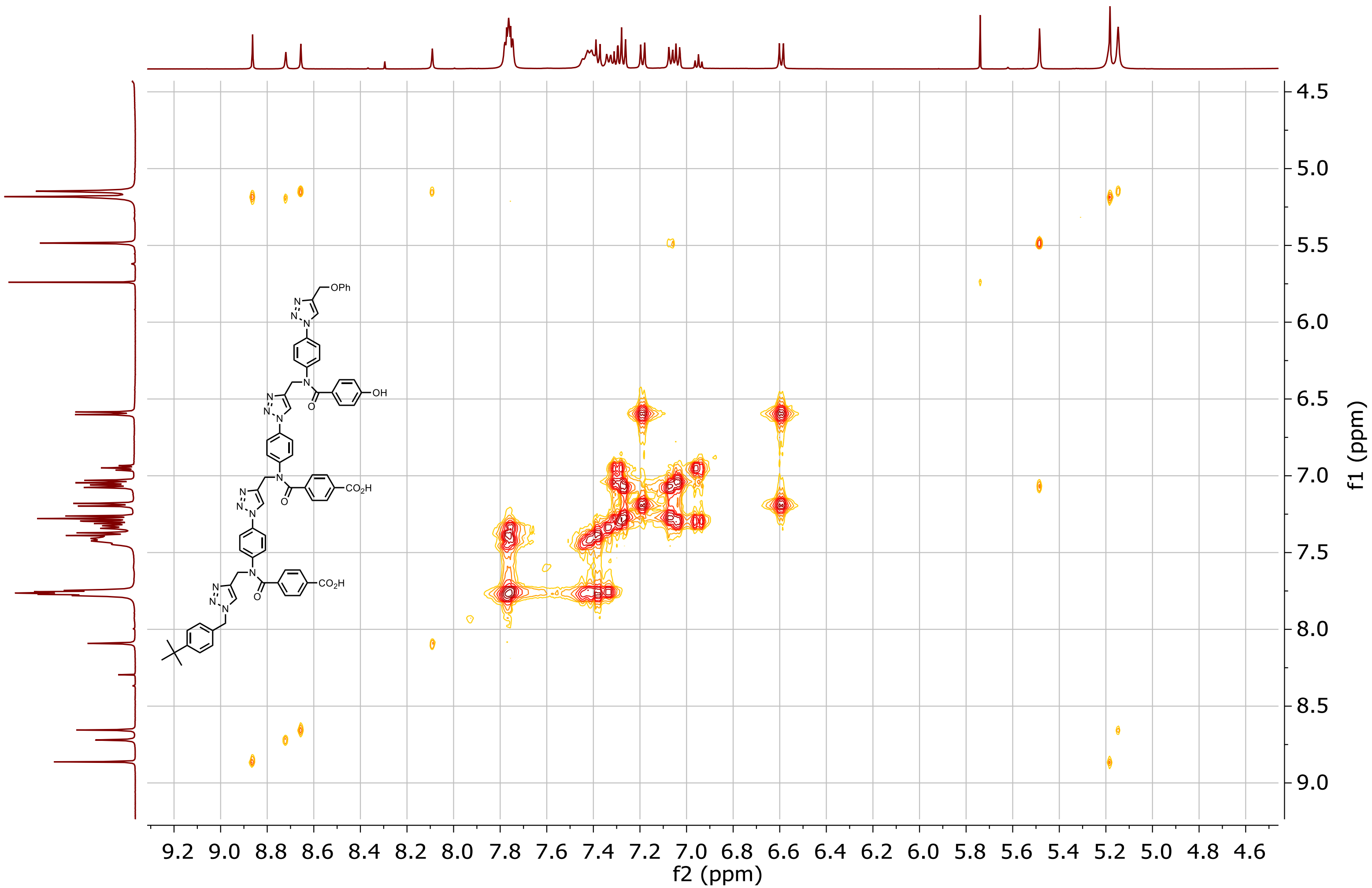

HSQC (DMSO-d_6) Compound 13 (AAP template).

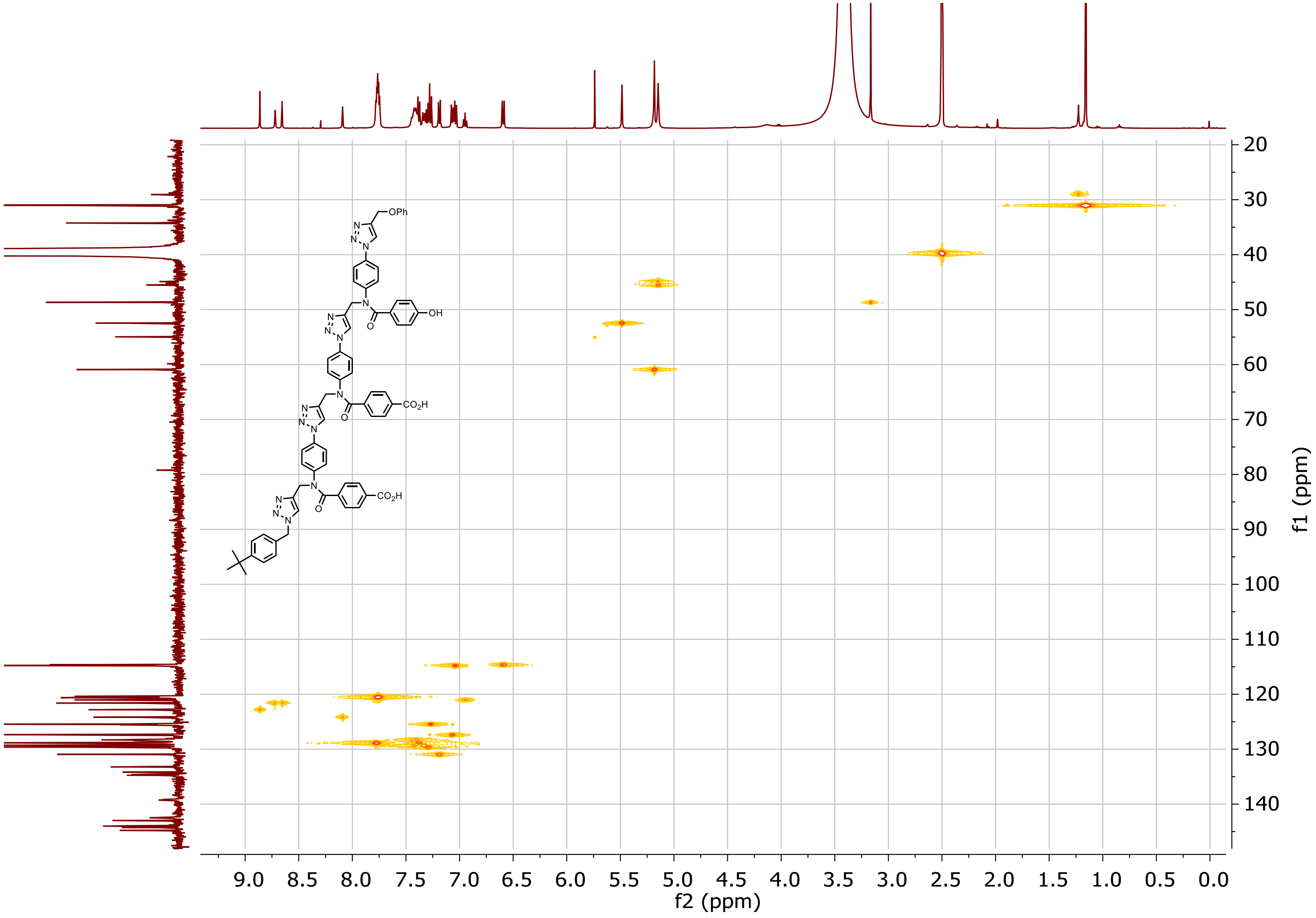

HMBC (DMSO-d_6) Compound 13 (AAP template).

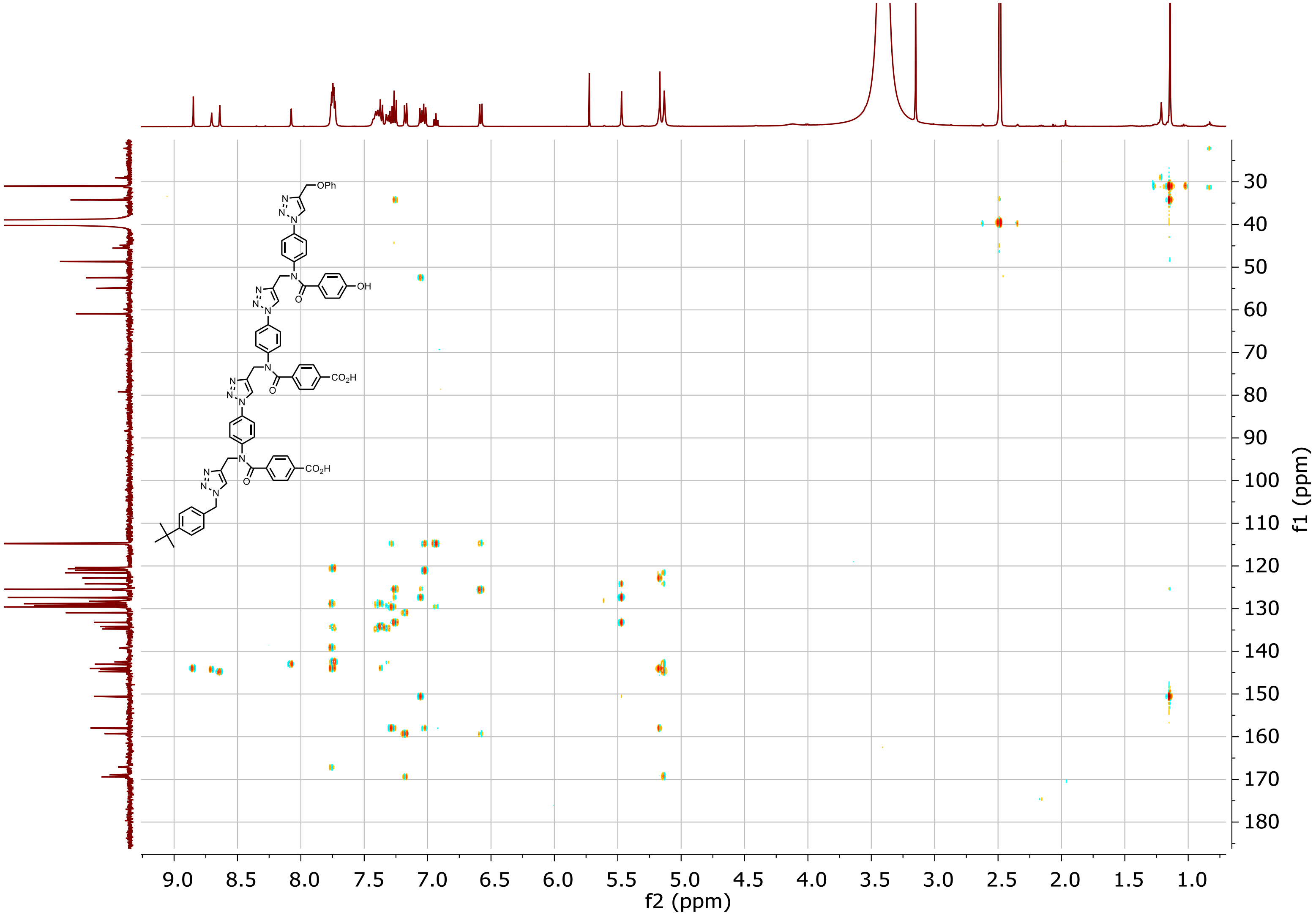

LCMS Compound 13 (AAP template).

Conditions: C18 column at 40 °C (254 nm) using water + 0.1% formic acid (A) and CH_3CN + 0.1% formic acid (B) with flow rate of 0.6 ml/min; Gradient of 0-2 minutes 5% -100%B + 1 minute 100% B.

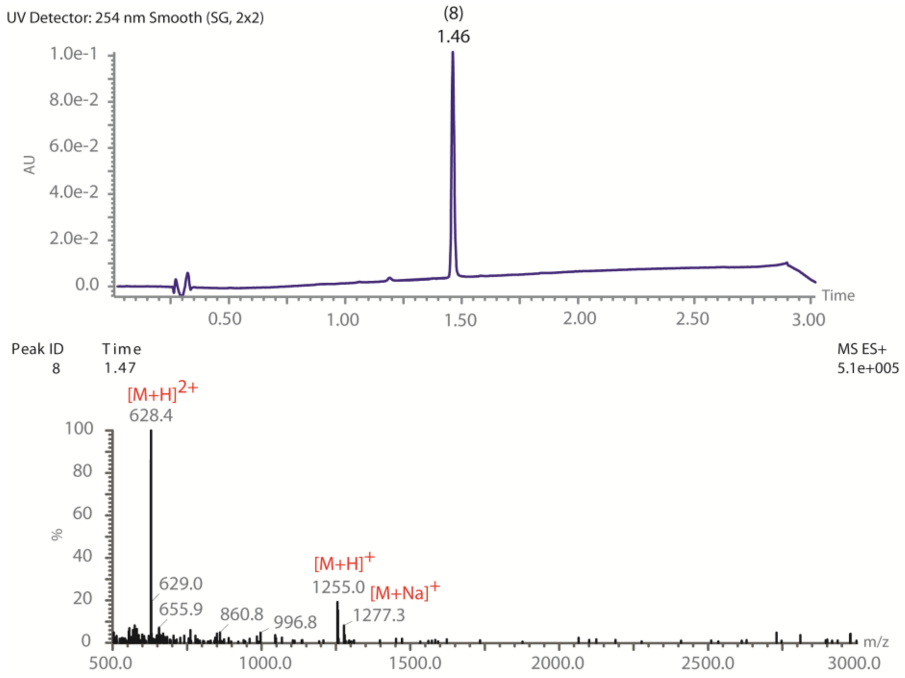

HRMS Compound 13 (AAP template).

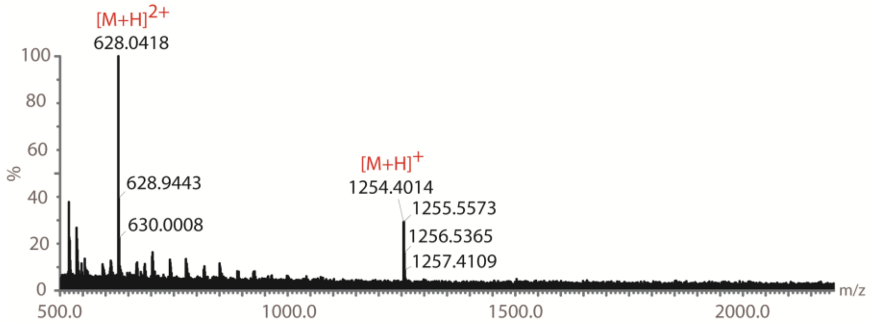

Synthesis of APP 3-mer[S2]

As shown in Scheme S9, the synthesis of APP 3-mer involve the preparation of phenol 2-mer **14**, synthesized by CuAAC coupling of **10** with protected monomer **8** in excellent yield. CuAAC coupling of **14** with **15**, obtained after capping monomer **6** with *p-tert*-butylbenzylazide, yielded 3-mer **16** in moderate yield.

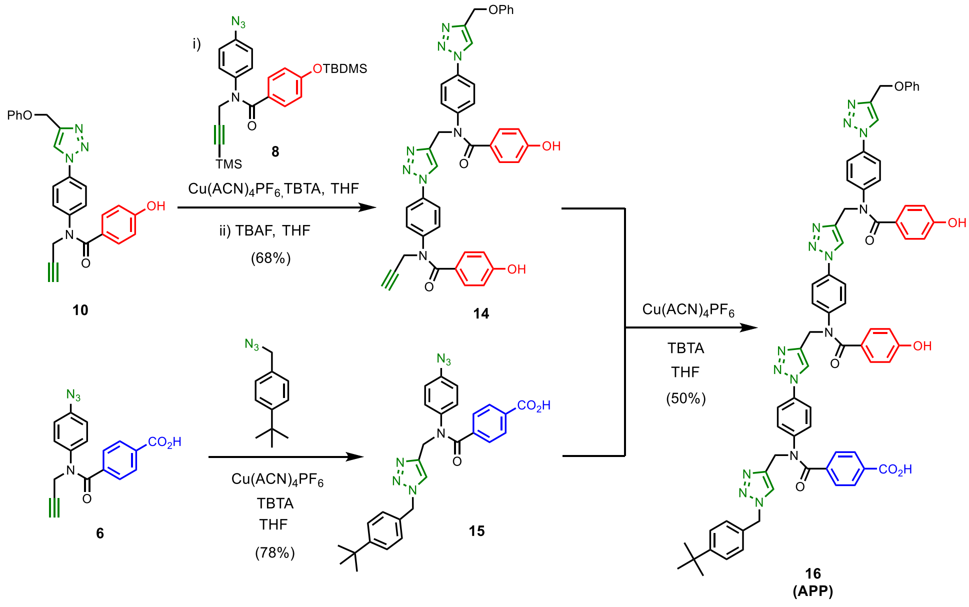

Scheme S9

Compound 14.

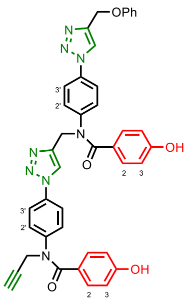

Compound **10** (0.020 g, 0.05 mmol), compound **8** (0.020 g, 0.04 mmol), $Cu(CH_3CN)_4PF_6$ (0.008 g, 0.02 mmol) and TBTA (0.011 g, 0.02 mmol) were mixed in a round-bottom flask and, under N_2, THF (2 mL) was added. The reaction was stirred overnight at room temperature. Once the reaction was completed, TBAF (1M in THF, 0.100 mL, 0.10 mmol) was added dropwise. The solution was stirred for 10 min at room temperature, quenched with 5% soln. HCl and extracted with EtOAc (3x) followed by washing with H_2O and brine. The organic layer was dried over anhydrous $MgSO_4$ and concentrate under vacuum. The crude was purified by flash column chromatography on silica gel (gradient from 10% to 90% of EtOAc in Pet. Ether) to afford **14** (0.022 g, 68%) as a foam.

^{1}H NMR (400 MHz, DMSO-*d*$_6$): δ_H = 9.88 (s, 2H, OH), 8.89 (s, 1H, CH_{triaz}), 8.74 (s, 1H, CH_{triaz}), 7.85 (d, 2H, *J* = 9.0 Hz, 3'-H), 7.80 (d, 2H, *J* = 9.0 Hz, 3'-H), 7.43 (d, 2H, *J* = 9.0 Hz, 2'-H), 7.34 (d, 2H, *J* = 9.0 Hz, 2'-H), 7.30 (m, 2H, 3''-H, PhO), 7.22 (d, 2H, *J* = 9.0 Hz, 2-H), 7.18 (d, 2H, *J* = 9.0 Hz, 2-H), 7.05 (d, 2H, *J* = 8.0 Hz, 2''-H, PhO), 7.34 (t, 2H, *J* = 7.5 Hz, 4''-H, PhO), 6.60 (d, 4H, *J* = 8.5 Hz, 3-H), 5.20 (s, 4H, O-CH_2 and N-CH_2), 4.67 (d, 2H, *J* = 2.5 Hz, N-CH_2), 3.17 (t, 1H, *J* = 2.5 Hz, alkyne).

^{13}C NMR (100.6 MHz, DMSO-*d*$_6$): δ_C = 169.3 and 169.0 (CO, amide), 159.2 and 159.0 (4-C), 157.9 (1''-C), 144.7 ($C_{triaz,}$, 144.0 (C_{triaz}), 143.9 (1'-C), 143.0 (1'-C), 134.5 (4'-C), 134.2 (4'-C), 131.0 and 130.9 (2-C), 129.5 (3''-C, PhO), 128.8 and 128.8, (2'-C), 125.7 and 125.3 (1-C), 122.8 and 121.6 (CH_{triaz}), 121.0 (4''-C, PhO), 120.6 and 120.5 (3'-C), 114.7 (2''-C, PhO), 114.6 and 114.5 (3-C), 79.7 (C, alkyne), 74.8 (CH, alkyne), 60.9 (O-CH_2), 45.5 (N-CH_2), 38.9 (N-CH_2).

HRMS (ES+): calcd for $C_{41}H_{33}N_8O_5$ 717.2574 $[M+H]^+$, found 717.2566 $[M+H]^+$.

FT-IR (ATR): ν_{max} 3133, 2921, 1633, 1587, 1517, 1279, 1237, 1170, 845 and 758 cm^{-1}.

^{1}H-NMR (400 MHz, DMSO-d_6) Compound 14.

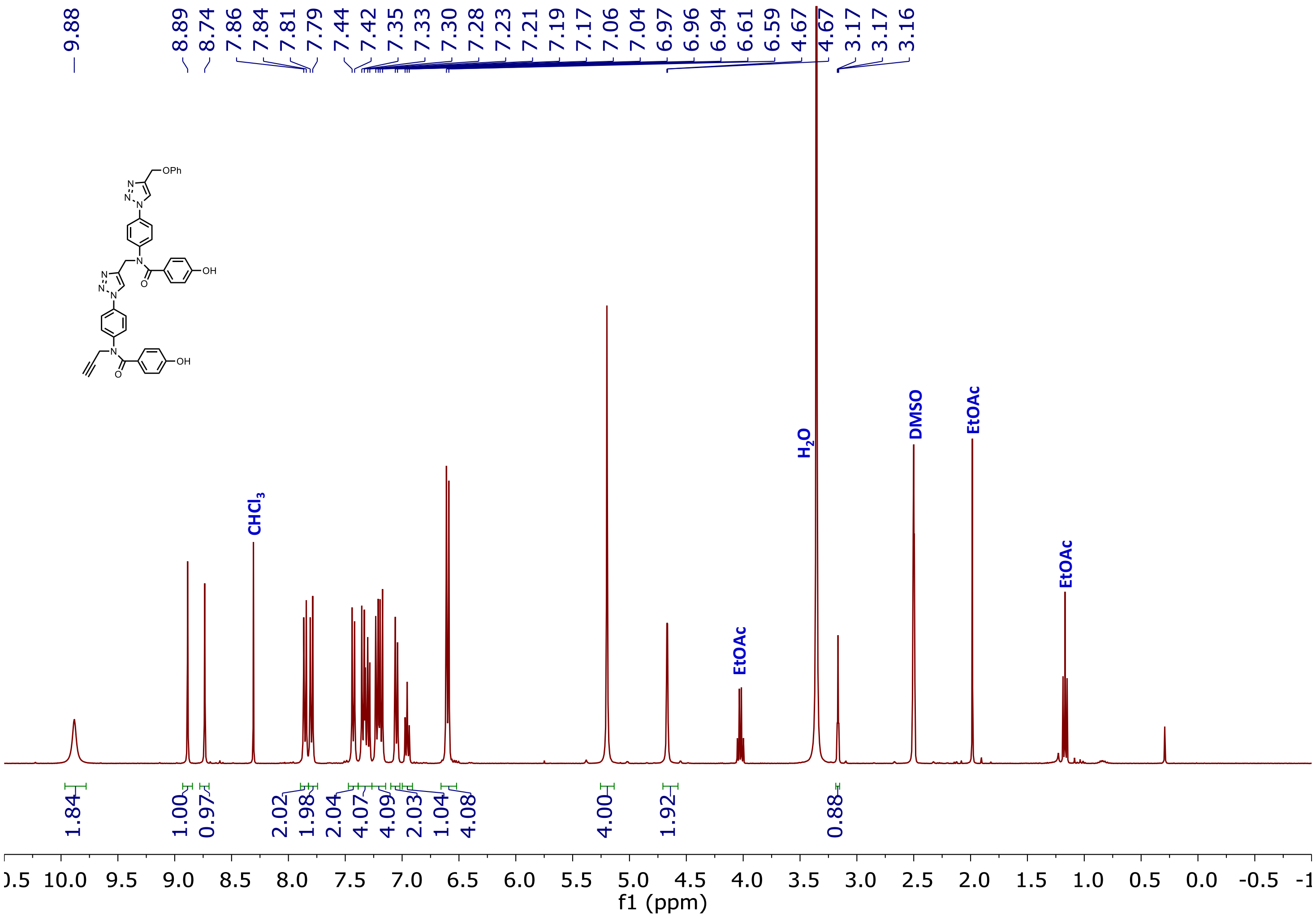

^{13}C-NMR (100.6 MHz, DMSO-d_6) Compound 14.

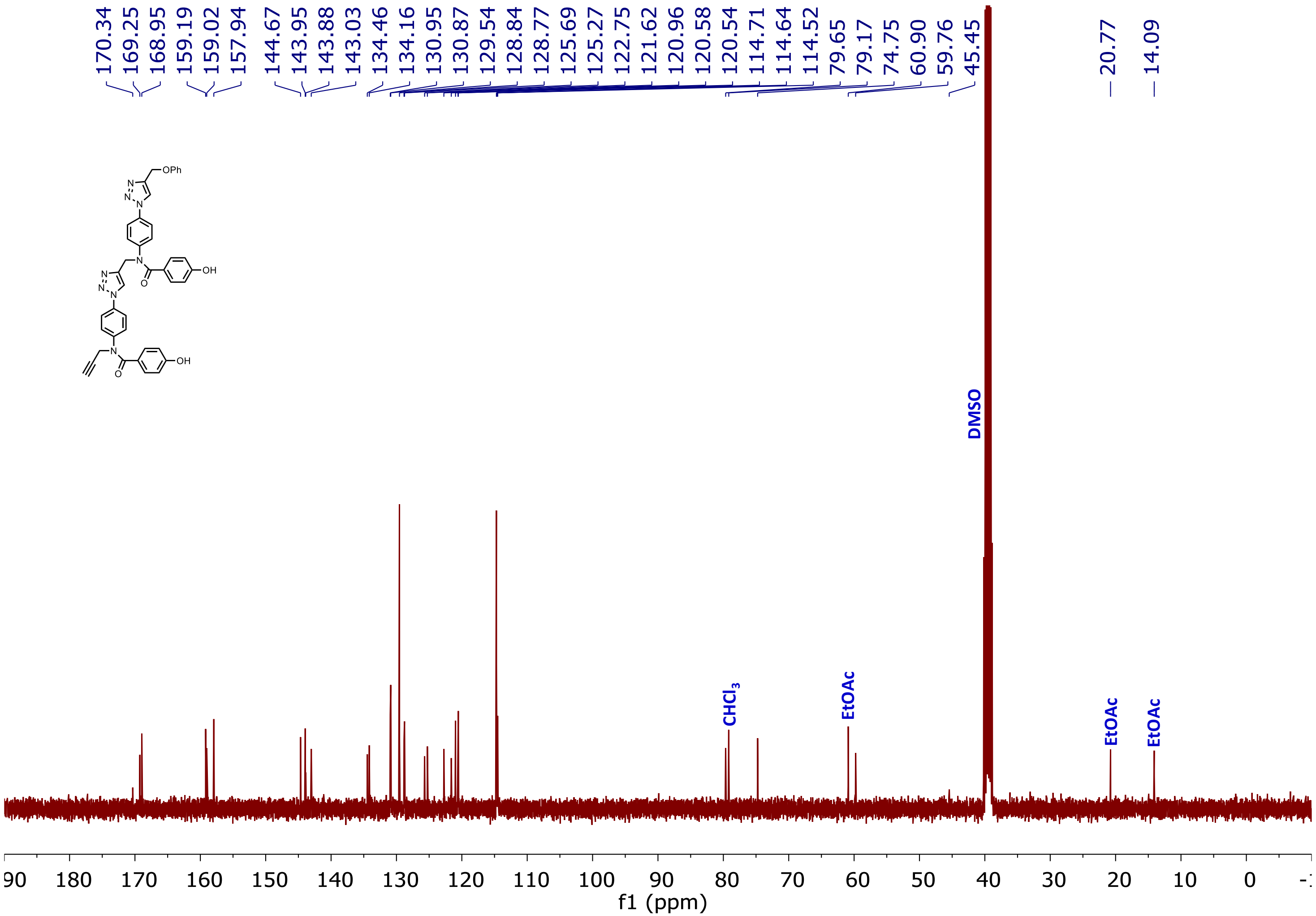

Compound 15.

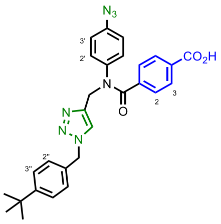

Compound **6** (0.025 g, 0.08 mmol), 1-(azidomethyl)-4-*tert*-butylbenzene (0.174 g, 0.92 mmol), $Cu(CH_3CN)_4PF_6$ (0.003 g, 0.01 mmol) and TBTA (0.004 g, 0.01 mmol) were mixed in a round-bottom flask and, under N_2, THF (100 mL) was added. The reaction was stirred overnight at room temperature. The solvent was evaporated to dryness and the residue dissolved in EtOAc and washed with 0.02M EDTA soln. (2x), H_2O (1x) and brine. The organic layer was dried over $MgSO_4$ and concentrate under vacuum. The residue was purified by flash chromatography on silica gel (gradient from 0% to 10% of MeOH with 0.1% acetic acid in EtOAc) to afford **15** (0.031 g, 78% containing TBTA as impurity) as a white solid.

^{1}H NMR (400 MHz, CDCl$_3$): δ_H = 7.85 (d, 2H, *J* = 8.5 Hz, 3-H), 7.66 (s, 1H, CH_{triaz}), 7.38 (d, 2H, *J* = 8.5 Hz, 3''-H), 7.31 (m, 2H, 2-H), 7.18 (d, 2H, *J* = 8.5 Hz, 2''-H), 7.06 (d, 2H, *J* = 8.5 Hz, 2'-H), 6.81 (d, 2H, *J* = 8.5 Hz, 3'-H), 5.47 (s, 2H, N-CH_2), 5.09 (s, 2H, N-CH_2), 1.30 (s, 9H, ^{t}Bu).

^{13}C NMR (100.6 MHz, CDCl$_3$): δ_C = 169.9 (CO, amide), 169.5 (CO, acid), 152.1(4''-C), 143.9 (C_{triaz}), 140.2 (1'-C), 139.9 (4'-C), 139.1 (1-C), 131.5 (1''-C), 131.0 (4-C), 129.8 (3-C), 129.1 (2'-C), 128.7 (2-C), 128.0 (2''-C), 126.2 (3''-C), 123.9 (CH_{triaz}), 120.0 (3'-C), 54.1 (N-CH_2), 46.2 (N-CH_2), 34.8 (C, ^{t}Bu), 31.4 (CH_3, ^{t}Bu).

HRMS (ES+): calcd for $C_{28}H_{28}N_7O_3$ 510.2254 $[M+H]^+$, found 510.2265 $[M+H]^+$.

FT-IR (ATR): ν_{max} 2961, 2925, 2856, 2126, 2097, 1636, 1505, 1294, 1277, 837 and 755 cm^{-1}.

^{1}H-NMR (400 MHz, $CDCl_3$) Compound 15.

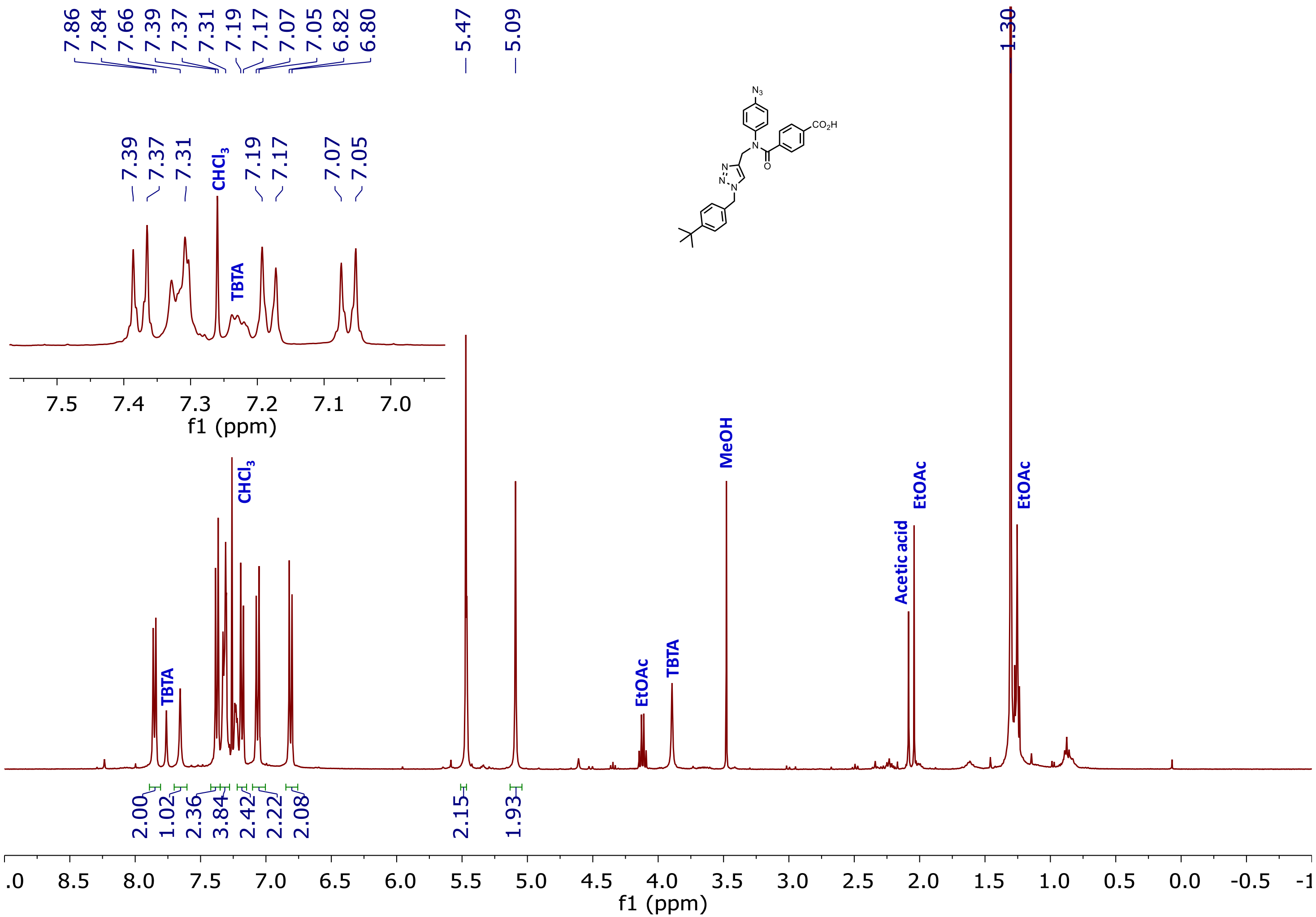

^{13}C-NMR (100.6 MHz, $CDCl_3$) Compound 15.

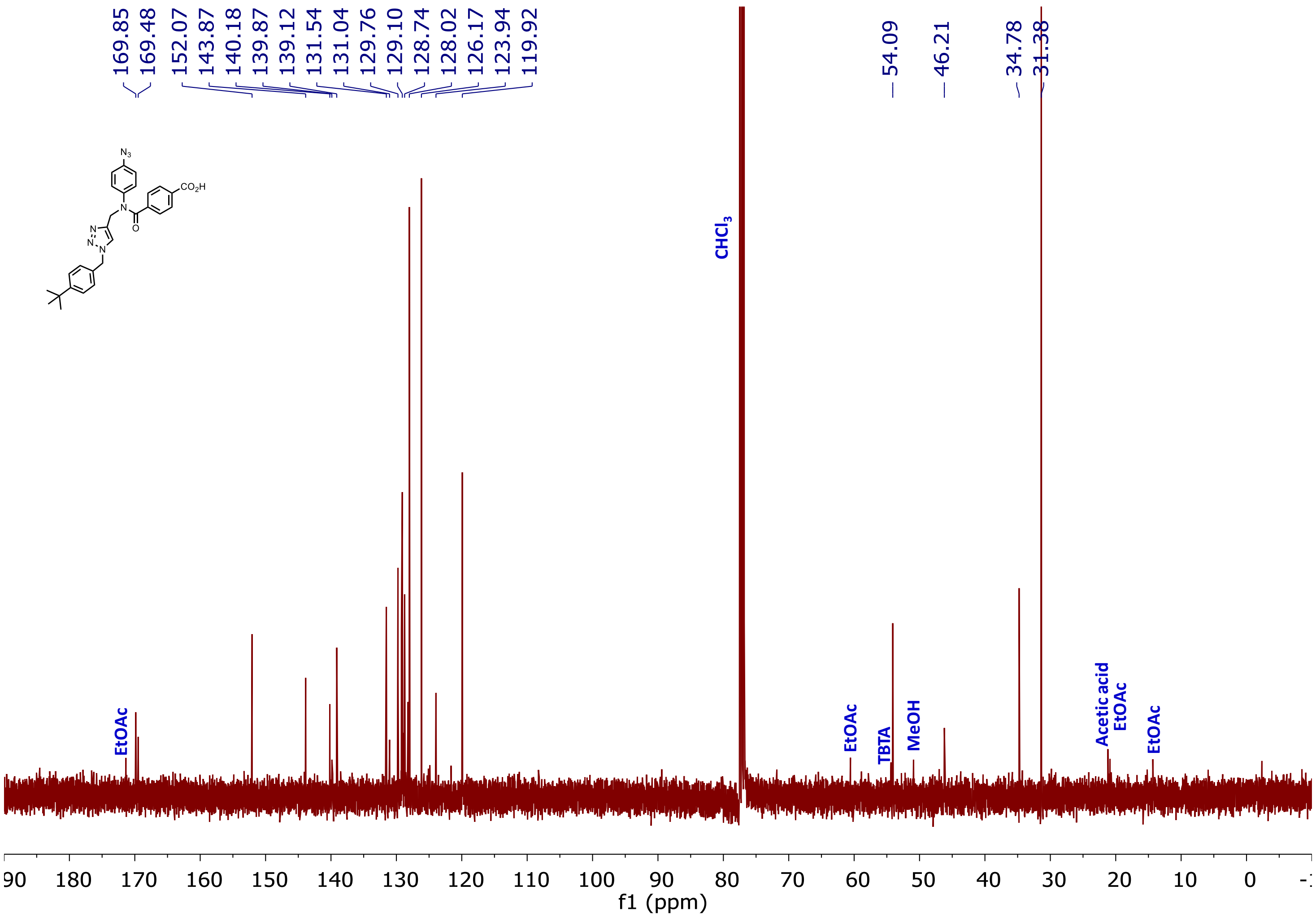

Compound 16 (APP 3-mer).

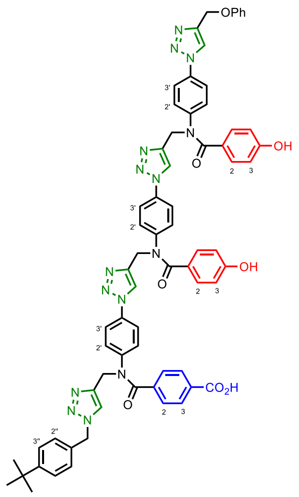

15 (0.018 g, 0.04 mmol), **14** (0.034 g, 0.05 mmol), $Cu(CH_3CN)_4PF_6$ (0.013 g, 0.04 mmol) and TBTA (0.02 g, 0.04 mmol) were mixed in a round-bottom flask and, under N_2, THF (2 mL) was added. The reaction was stirred overnight at room temperature. The reaction was then diluted with EtOAc and washed with 0.02M EDTA soln. (2x), H_2O (1x) and brine. The organic layer was dried over $MgSO_4$ and concentrate under vacuum. The residue was purified by flash column chromatography on silica gel (gradient from 0% to 20% of MeOH in CH_2Cl_2) to afford compound **16** (0.022 g, 50%) as a waxy solid.

^{1}H NMR (500 MHz, DMSO-*d*$_6$): δ_H = 9.87 (s, 2H, OH), 8.88 (s, 1H, CH_{triaz}), 8.69 (s, 1H, CH_{triaz}), 8.68 (s, 1H, CH_{triaz}), 8.09 (s, 1H, CH_{triaz}), 7.77 (m, 8H, 3-H, acid; 3'-H), 7.39 (m, 4H, 2'-H), 7.37 (d, 2H, *J* = 9.0 Hz, 2'-H), 7.30 (m, 6H, 2-H, acid; 3''-H, PhO; 3''-H, ^{t}BuPh), 7.20 (d, 2H, *J* = 8.5 Hz, 2-H, phenol), 7.20 (d, 2H, *J* = 8.5 Hz, 2-H, phenol), 7.08 (d, 2H, *J* = 8.5 Hz, 2''-H, ^{t}BuPh), 7.04 (d, 2H, *J* = 8.0 Hz, 2''-H, PhO), 6.95 (t, 1H, *J* = 7.5 Hz, 4''-H, PhO), 6.60 (d, 2H, *J* = 8.5 Hz, 3-H, phenol), 6.58 (d, 2H, *J* = 8.5 Hz, 3-H, phenol), 5.49 (s, 2H, N-CH_2), 5.19 (s, 2H, N-CH_2), 5.17 (s, 2H, N-CH_2), 5.15 (s, 4H, N-CH_2), 1.17 (s, 9H, ^{t}Bu).

^{13}C NMR (125 MHz, DMSO-*d*$_6$): δ_C = 169.3 and 169.2 (CO, amide phenol), 168.8 (CO, amide acid), 167.2 (CO, acid), 159.3 and 159.0 (4-C, phenol), 157.9 (1''-C, PhO), 150.5 (4''-C, ^{t}BuPh), 144.7, 144.6, and 143.9 (C_{triaz}), 143.9 and 143.7 (1'-C), 142.9 (C_{triaz}), 142.3 (1'-C), 139.0 (1-C, acid), 134.6, 134.2 and 134.1 (4'-C), 133.2 (4-C, acid), 130.9 and 130.8 (2-C, phenol), 129.6, 129.2, 128.8, 128.8, 128.2 and 127.3 (C_{arom}), 125.7 and 125.5 (1-C, phenol), 125.4 (3''-C, ^{t}BuPh), 124.1, 122.8, 121.5 and 121.5 (CH_{triaz}), 121.0 (4''-C, PhO), 120.6, 120.4 and 120.3 (3'-C), 114.7 (2''-C, PhO), 114.6 and 114.5 (3-C, phenol), 60.9 (O-CH_2), 52.4 (N-CH_2), 45.5, 45.4 and 44.9 (N-CH_2), 34.2 (C, ^{t}Bu), 31.0 (CH_3, ^{t}Bu).

HRMS (ES+): calcd for $C_{69}H_{60}N_{15}O_8$ 1226.4749 $[M+H]^+$, found 1226.4874$[M+H]^+$.

FT-IR (ATR): ν_{max} 2980, 1635, 1604, 1517, 1277, 1234, 1044, 846 and 756 cm^{-1}.

^{1}H-NMR (500 MHz, DMSO-d_6) Compound 16 (APP 3-mer).

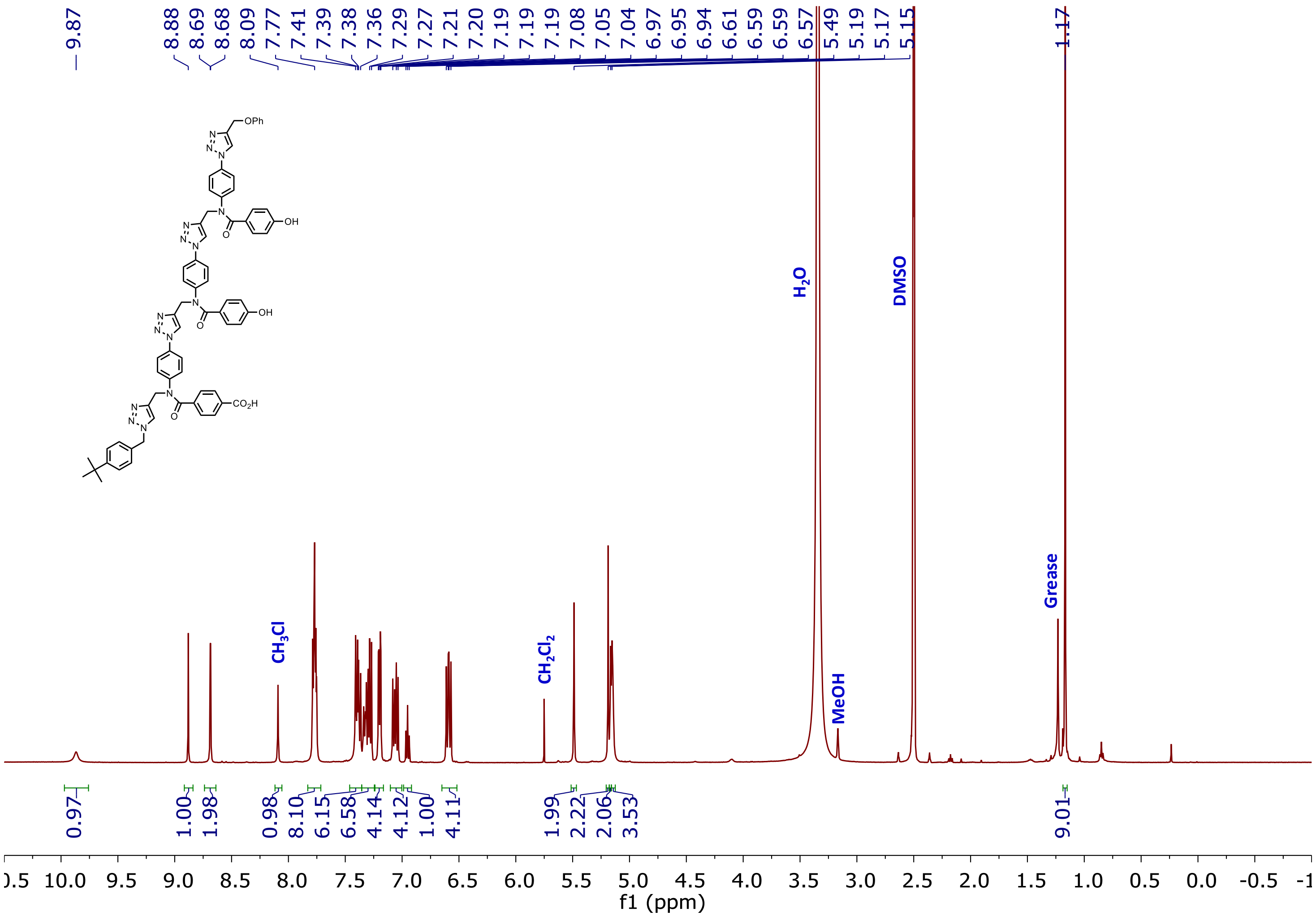

^{13}C-NMR (125MHz, DMSO-d_6) Compound 16 (APP 3-mer).

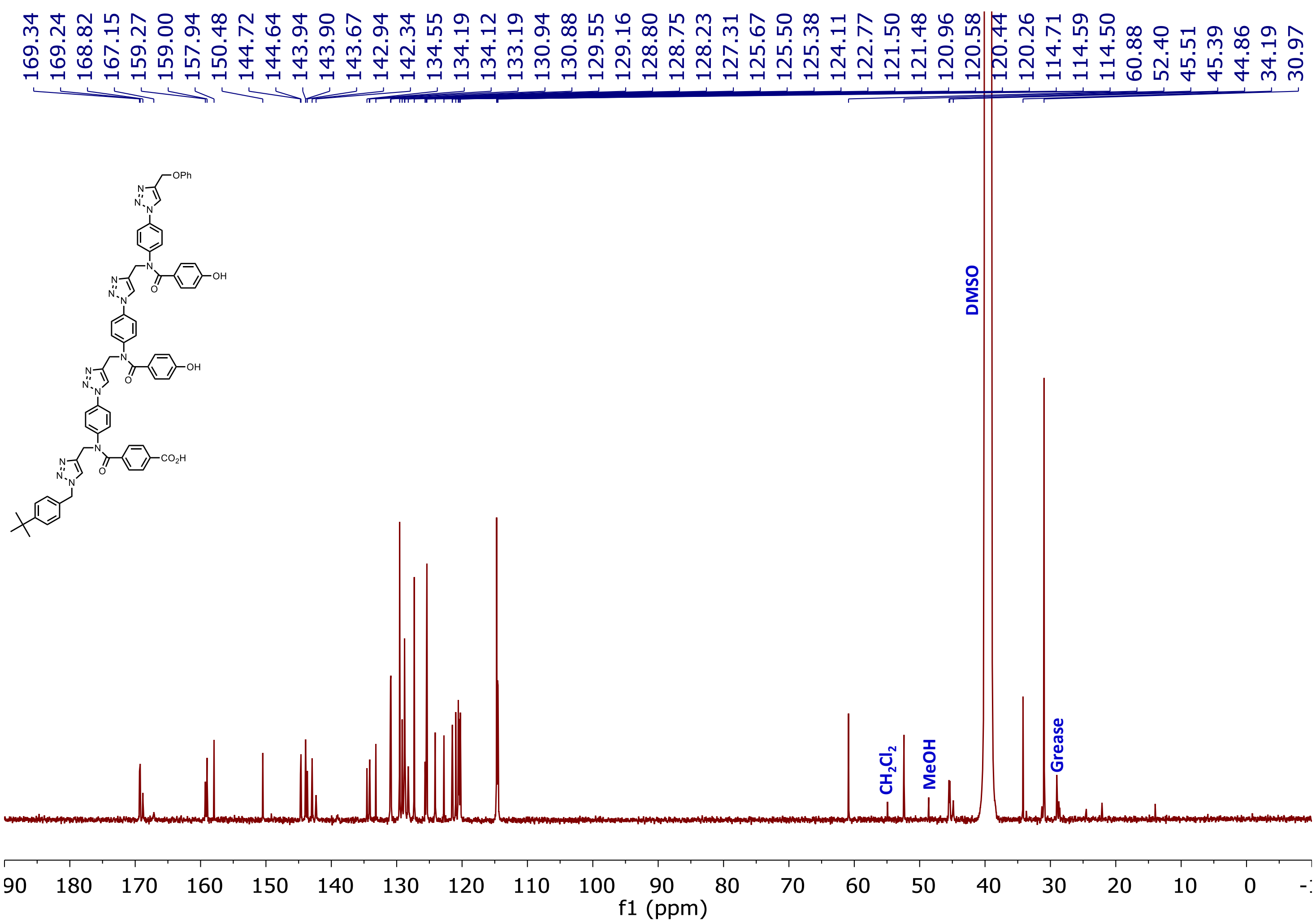

COSY (DMSO-d_6) Compound 16 (APP 3-mer).

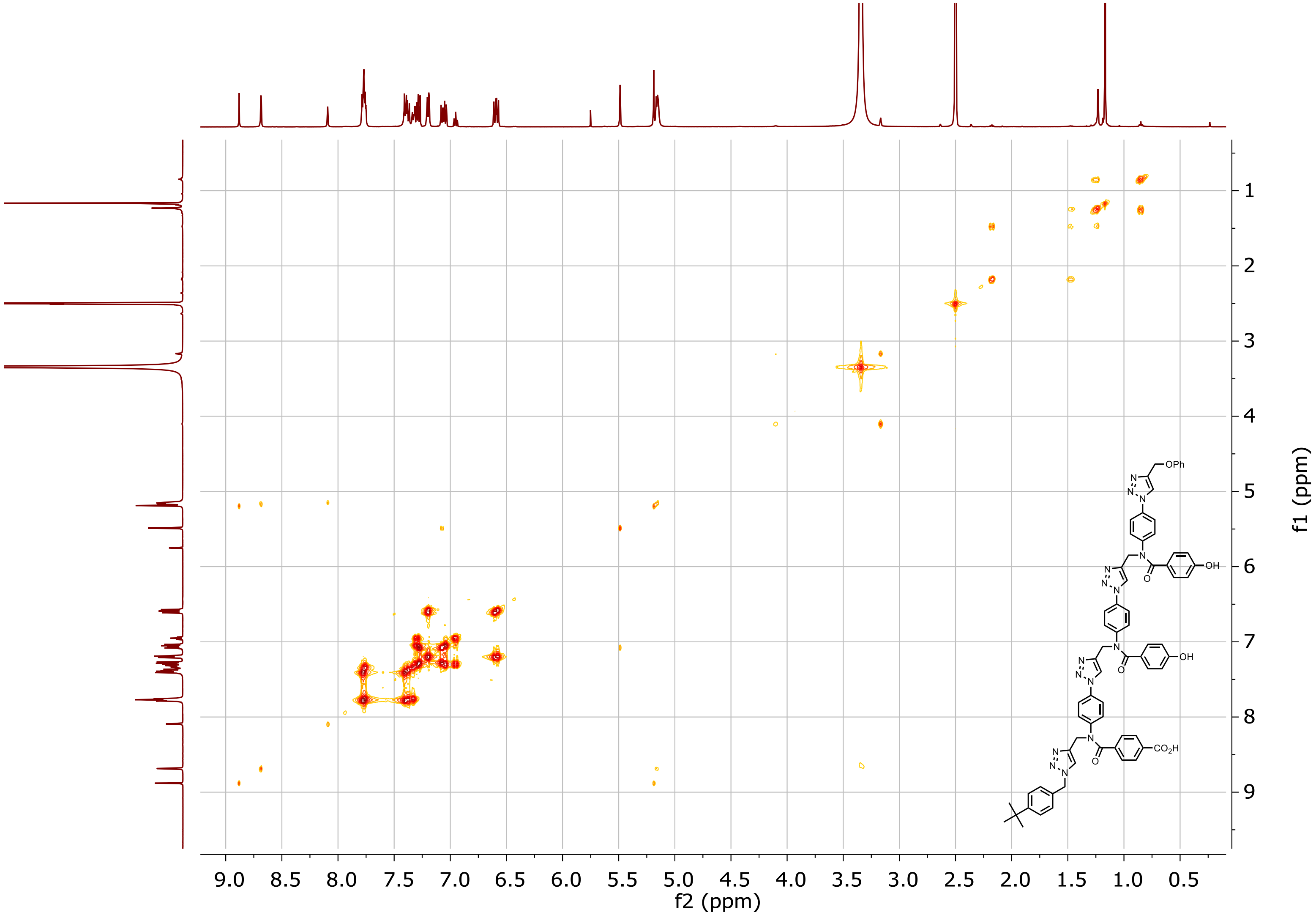

HSQC (DMSO-d_6) Compound 16 (APP 3-mer).

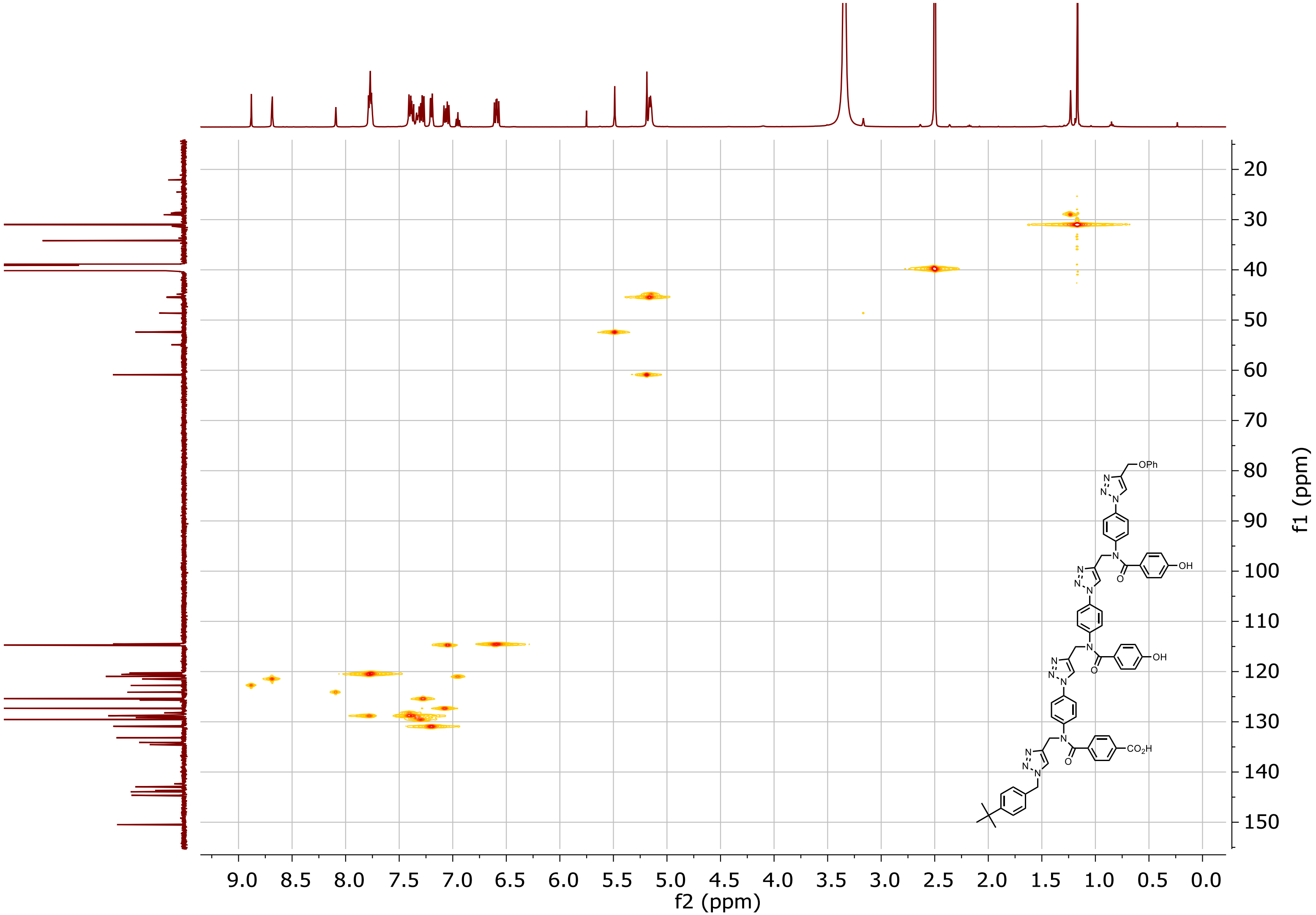

HMBC (DMSO-d_6) Compound 16 (APP 3-mer).

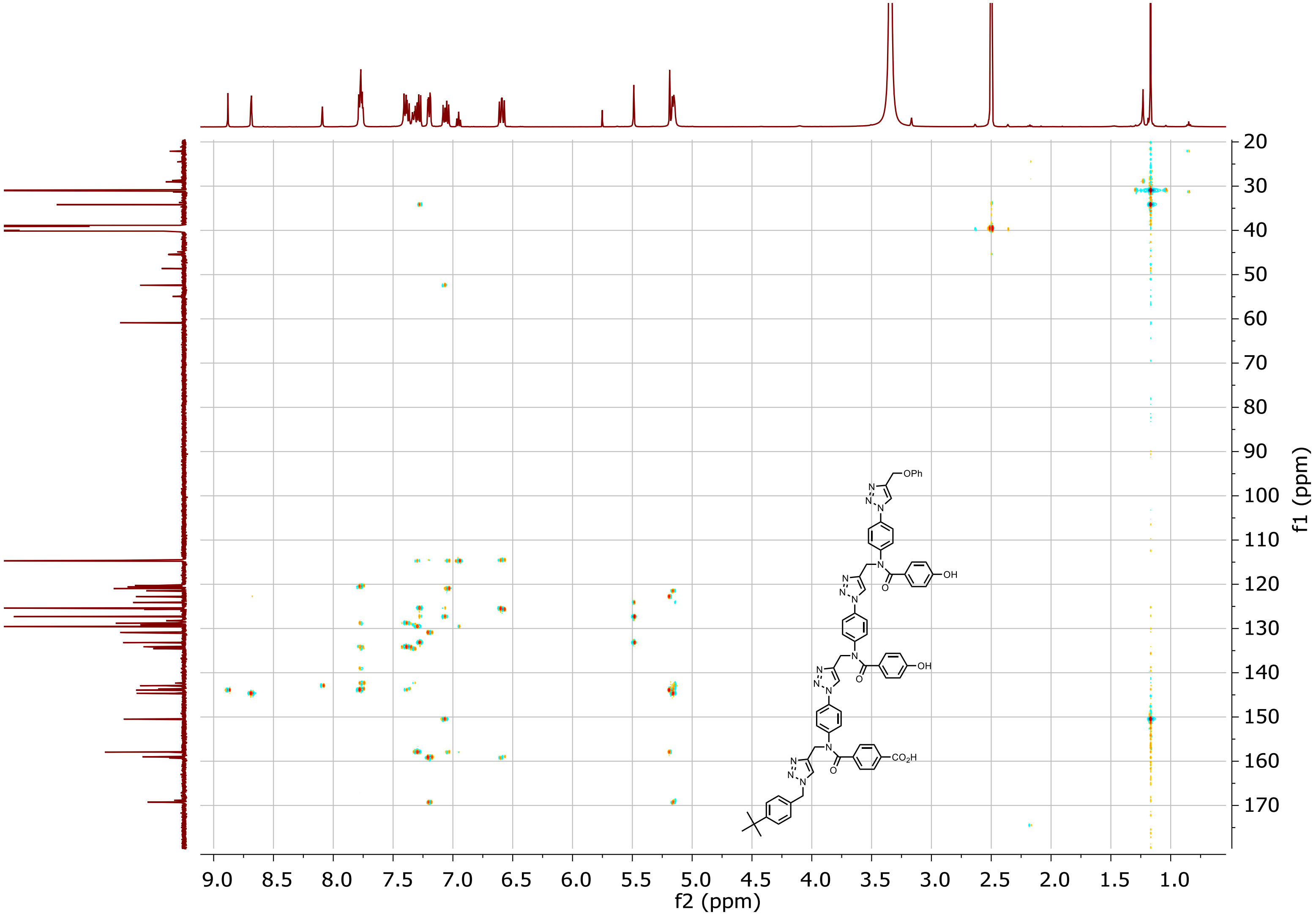

LCMS Compound 16 (APP 3-mer).

Conditions: C18 column at 40 °C (254 nm) using water + 0.1% formic acid (A) and CH_3CN + 0.1% formic acid (B) with flow rate of 0.6 ml/min; Gradient of 0-2 minutes 5% -100%B + 1 minute 100% B.

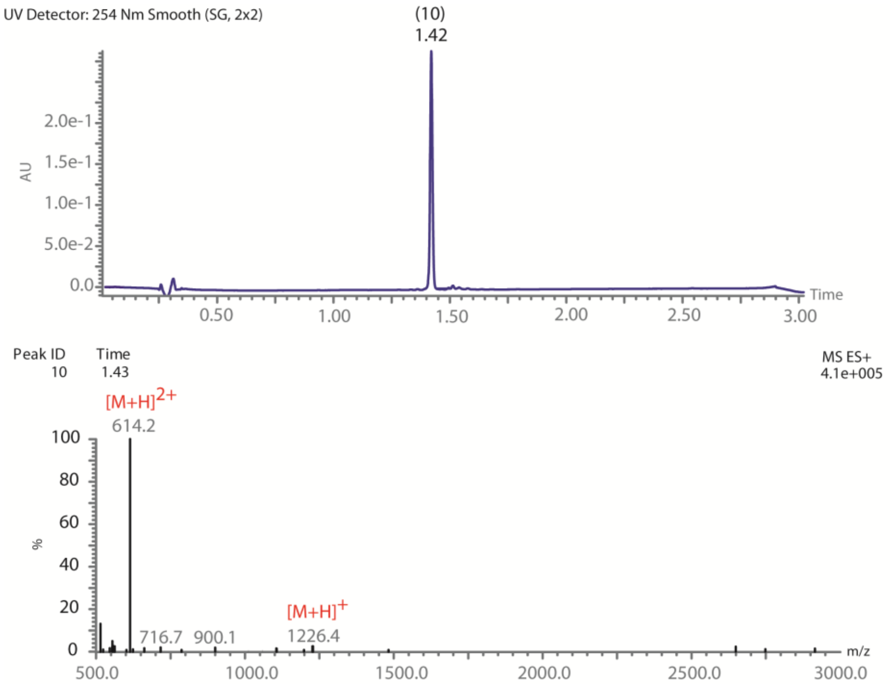

HRMS Compound 16 (APP 3-mer).

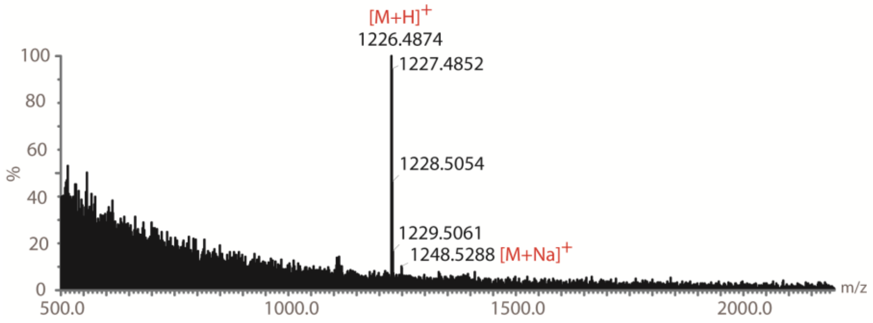

Synthesis of PPA 3-mer[S2]

As shown in Scheme S10, the synthesis of PPA 3-mer required the preparation of compounds **19** and **21**. Protected 1-mer **4** was capped with phenyl propargyl ether by copper(I)-catalyzed azide-alkyne cycloaddition (CuAAC). The reaction yielded TMS-protected 1-mer **17** as the main product, although some alkyne-deprotected product (**18**) was isolated. A mixture of **4** and **5** was subjected to basic hydrolysis with LiOH affording carboxylic acid **19** in good yield. Phenol 2-mer **20**, obtained from spontaneous azide-alkyne cycloaddition of 1-mer **9** upon storage, was capped with 1-(azidomethyl)-4-*tert*-butylbenzene providing capped 2-mer **21**. CuAAC coupling of **19** and **21** gave access to 3-mer 22 in excellent yield.

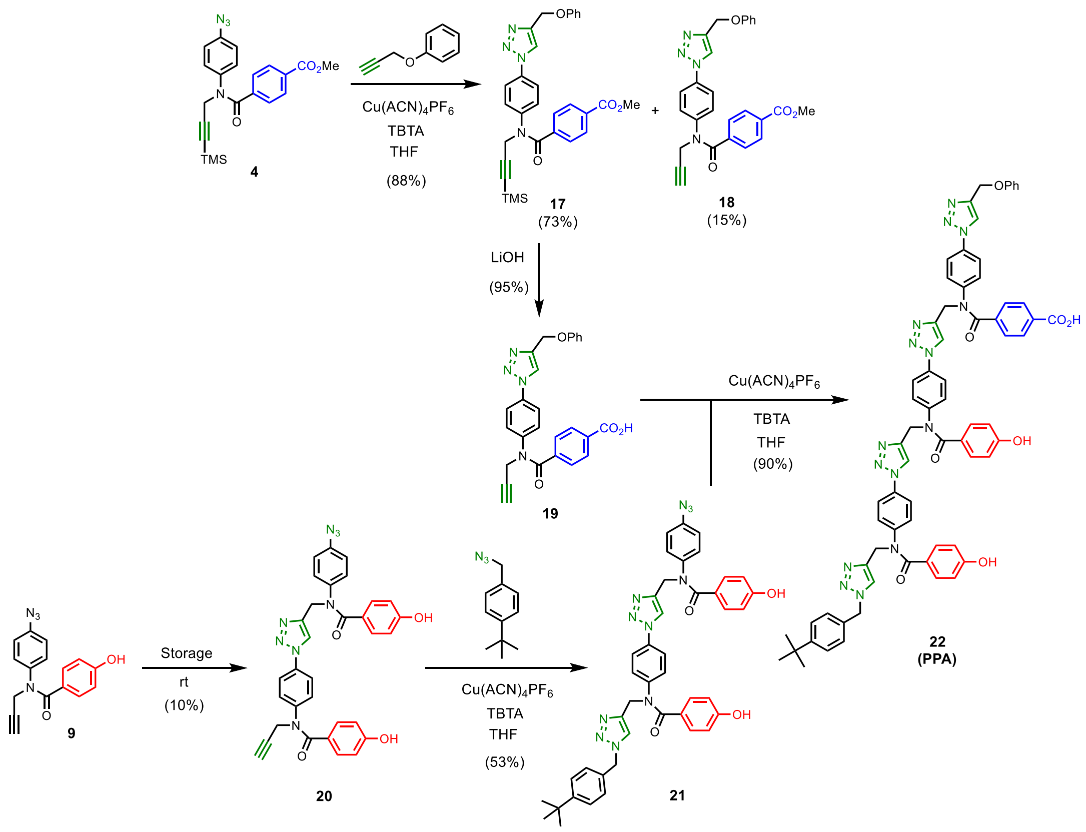

Scheme S10

Compounds 17 and 18.

Compound **4** (0.258 g, 0.64 mmol), $Cu(CH_3CN)_4PF_6$ (0.011 g, 0.03 mmol) and TBTA (0.015 g, 0.03 mmol) were mixed in a round-bottom flask and, under N_2, THF (5 mL) was added. Phenyl propargyl ether (0.160 mL, 1.27 mmol) was added and the reaction was stirred overnight at room temperature. Once the reaction was completed, the solvent was evaporated and the crude was purified by flash column chromatography on silica gel (gradient from 0% to 30% of EtOAc in Pet. Ether) to afford **17** (0.248 g, 73%) as a foam, together with the TMS-unprotected compound **18** (0.046 g, 15%; overall yield 88%) as a foam.

Compound 17.

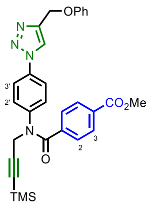

^{1}H NMR (400 MHz, CDCl$_3$): δ_H = 8.00 (s, 1H, CH$_{triaz}$), 7.88 (d, 2H, *J* = 8.0 Hz, 2-H), 7.65 (d, 2H, *J* = 9.0 Hz, 3'-H), 7.42 (d, 2H, *J* = 8.0 Hz, 3-H), 7.31 (m, 4H, 2'-H and 3''-H, PhO), 6.99 (m, 3H, 2''-H and 4''-H, PhO), 5.28 (s, 2H, O-CH$_2$), 4.73 (s, 2H, N-CH$_2$), 3.87 (s, 3H, O-CH$_3$), 0.13 (s, 9H, TMS).

^{13}C NMR (100.6 MHz, CDCl$_3$): δ_C = 168.9 (CO, amide), 166.0 (CO, ester), 158.0 (1''-C, PhO), 145.1 (C$_{triaz}$), 142.3 (1'-C), 139.2 (1-C), 135.6 (4'-C), 131.3 (4-C), 129.5, 129.3, 129.2 and 128.4 (2-C, 3-C, 2'-C and 3''-C, PhO), 121.3, 120.9 and 120.8 (CH$_{triaz}$, 3'-C and 4''-C, PhO), 114.6 (2''-C, PhO), 99.9 (C-TMS), 90.4 (C, alkyne), 61.7 (O-CH$_2$), 52.2 (O-CH$_3$), 40.3 (N-CH$_2$), -0.29 (TMS).

HRMS (ES+): calcd for $C_{30}H_{31}N_4O_4Si$ 539.2115 $[M+H]^+$, found 539.2151 $[M+H]^+$.

FT-IR (ATR): ν_{max} 2957, 1724, 1653, 1520, 1278, 1249, 845 and 756 cm^{-1}.

^{1}H-NMR (400 MHz, $CDCl_3$) Compound 17.

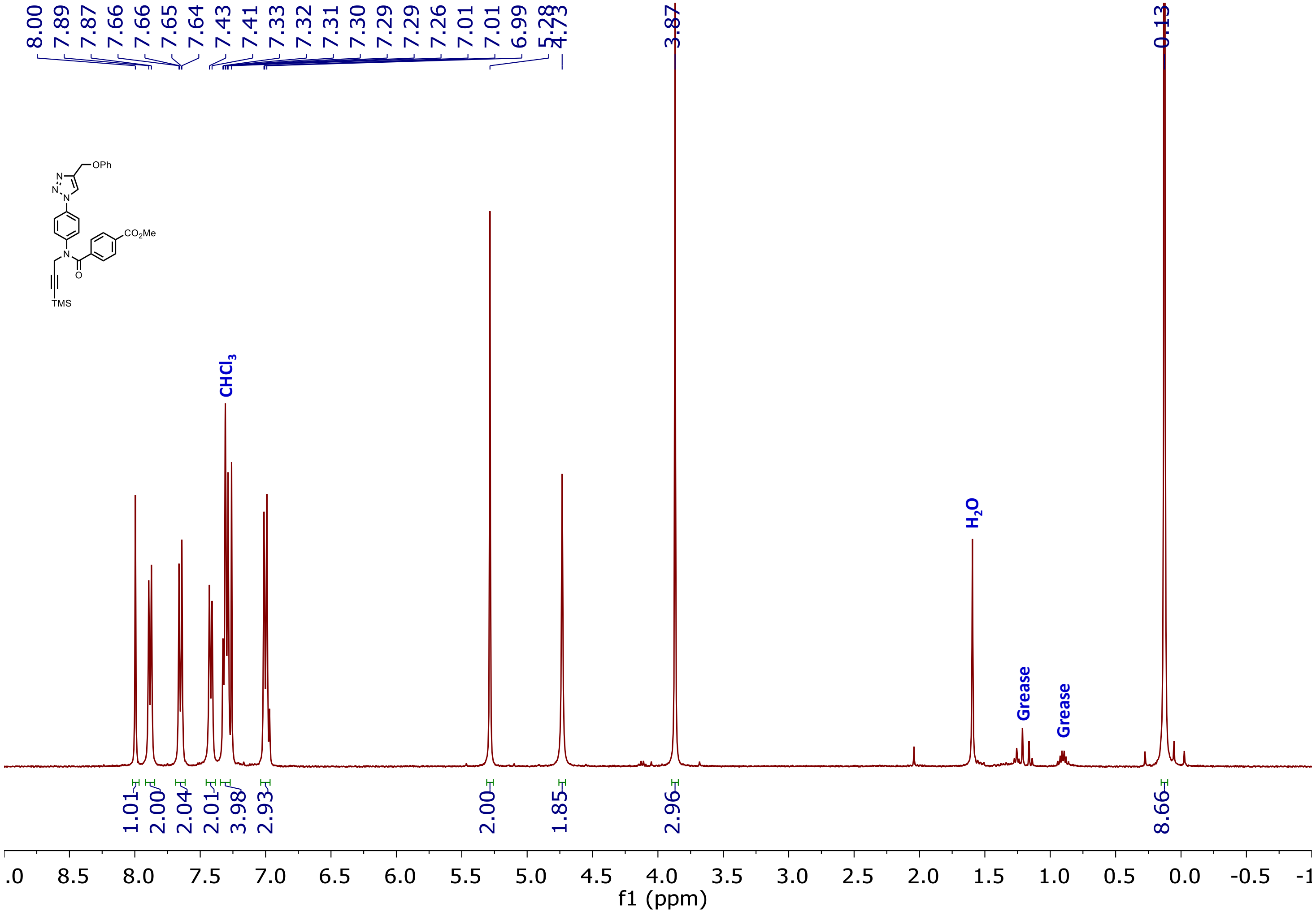

^{13}C-NMR (100.6 MHz, $CDCl_3$) Compound 17.

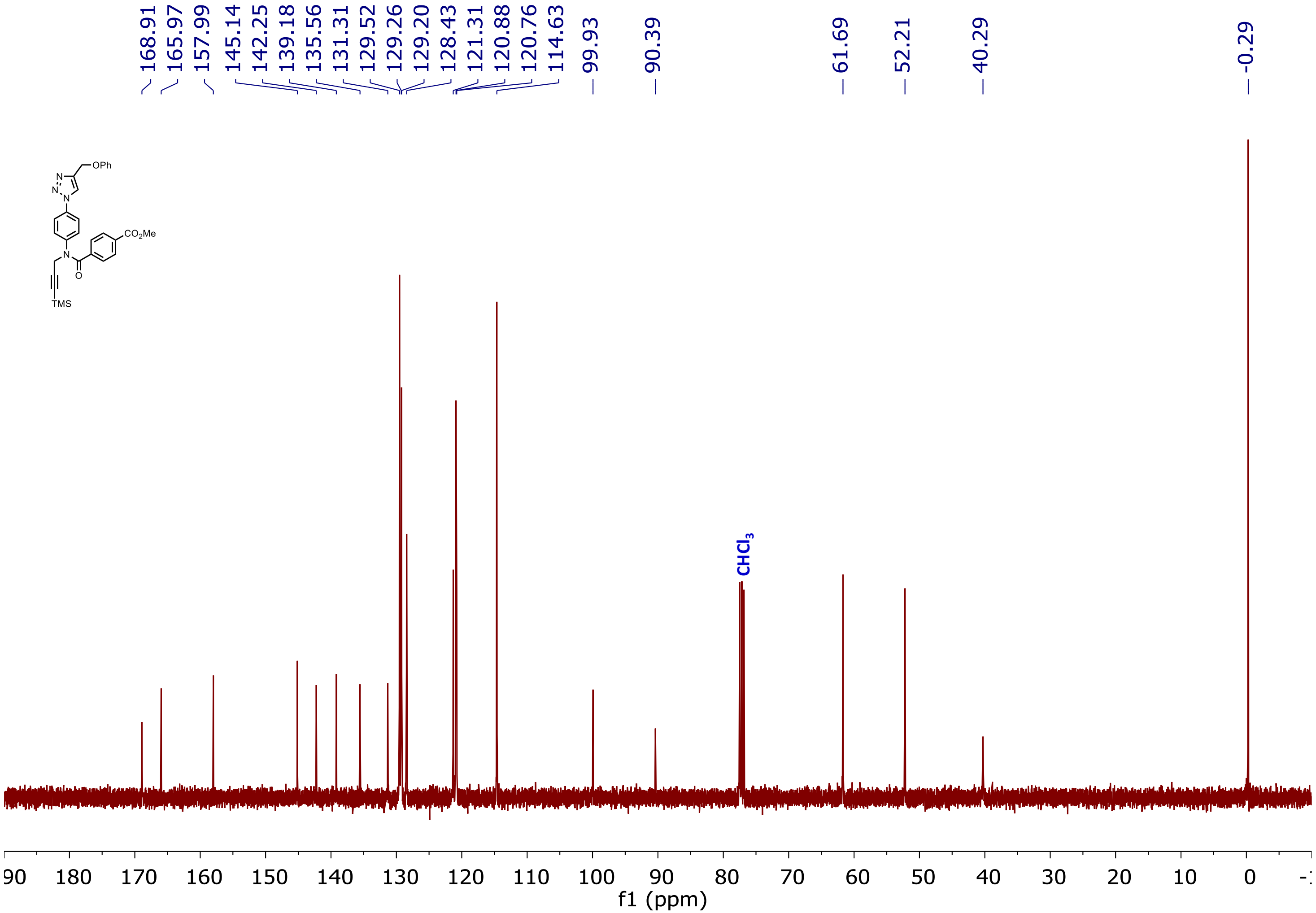

Compound 18.

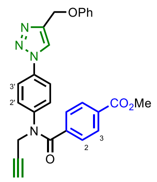

^{1}H NMR (400 MHz, CDCl$_3$): δ_H = 7.99 (s, 1H, CH$_{triaz}$), 7.88 (d, 2H, *J* = 8.0 Hz, 2-H), 7.65 (d, 2H, *J* = 9.0 Hz, 3'-H), 7.42 (d, 2H, *J* = 8.0 Hz, 3-H), 7.30 (m, 4H, 2'-H and 3''-H, PhO), 6.99 (m, 3H, 2''-H and 4''-H, PhO), 5.28 (s, 2H, O-CH$_2$), 4.72 (d, 2H, *J* = 2.5 Hz, N-CH$_2$), 3.87 (s, 3H, O-CH$_3$), 2.30 (t, 1H, *J* = 2.5 Hz, CH, alkyne).

^{13}C NMR (100.6 MHz, CDCl$_3$): δ_C = 169.3 (CO, amide), 166.2 (CO, ester), 158.2 (1''-C, PhO), 145.6 (C$_{triaz}$), 142.7 (1'-C), 139.1 (1-C), 135.8 (4'-C), 131.7 (4-C), 129.8, 129.5, 129.2 and 128.8 (2-C, 3-C, 2'-C and 3''-C, PhO), 121.6, 121.4 and 120.8 (CH$_{triaz}$, 3'-C and 4''-C, PhO), 114.9 (2''-C, PhO), 78.4 (C, alkyne), 73.3 (CH, alkyne), 62.0 (O-CH$_2$), 52.5 (O-CH$_3$), 39.8 (N-CH$_2$).

HRMS (ES+): calcd for $C_{27}H_{23}N_4O_4$ 467.1719 $[M+H]^+$, found 467.1719 $[M+H]^+$.

FT-IR (ATR): ν_{max} 3289, 1722, 1653, 1520, 1280, 1237 and 755 cm^{-1}.

^{1}H-NMR (400 MHz, $CDCl_3$) Compound 18.

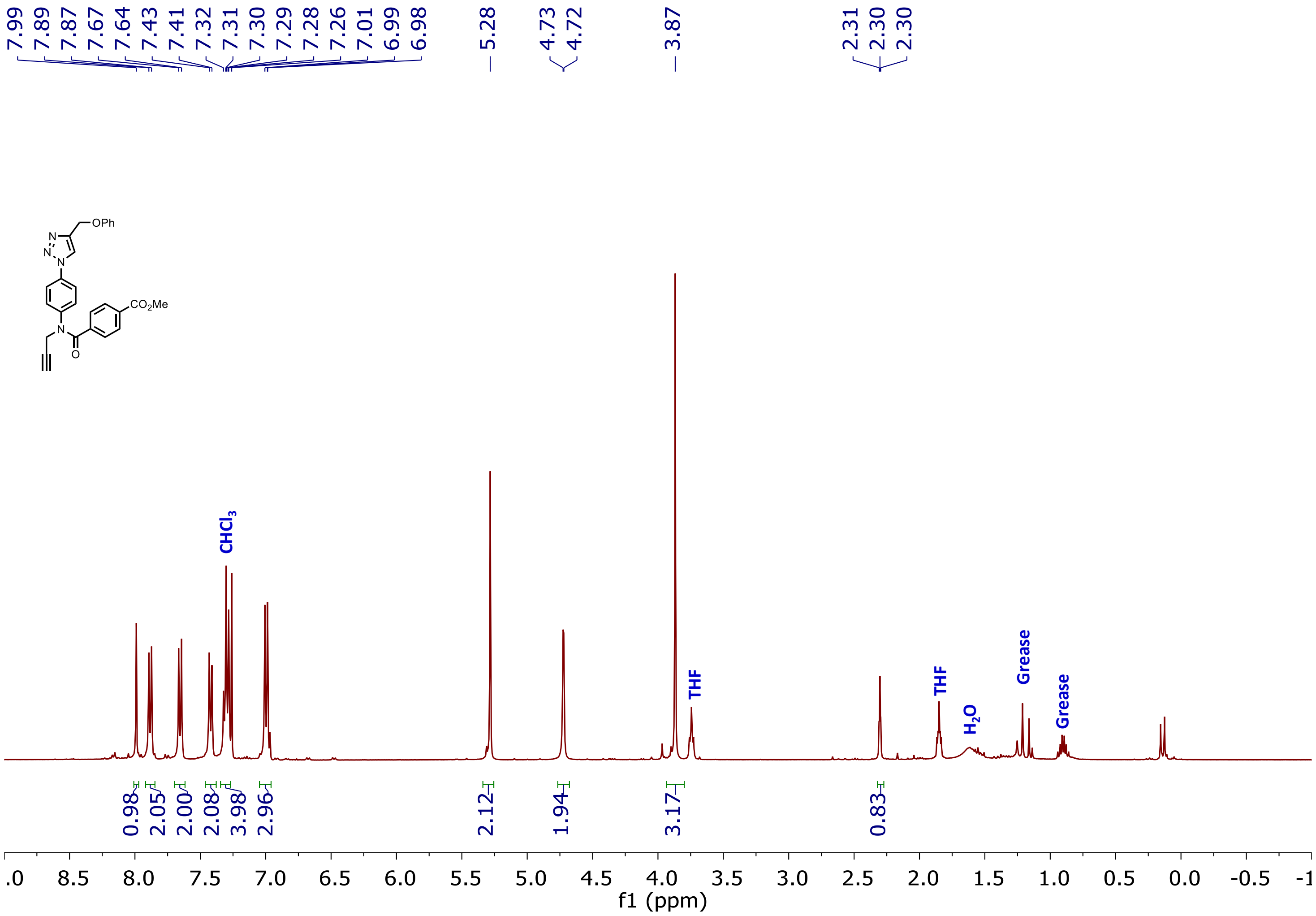

^{13}C-NMR (100.6 MHz, $CDCl_3$) Compound 18.

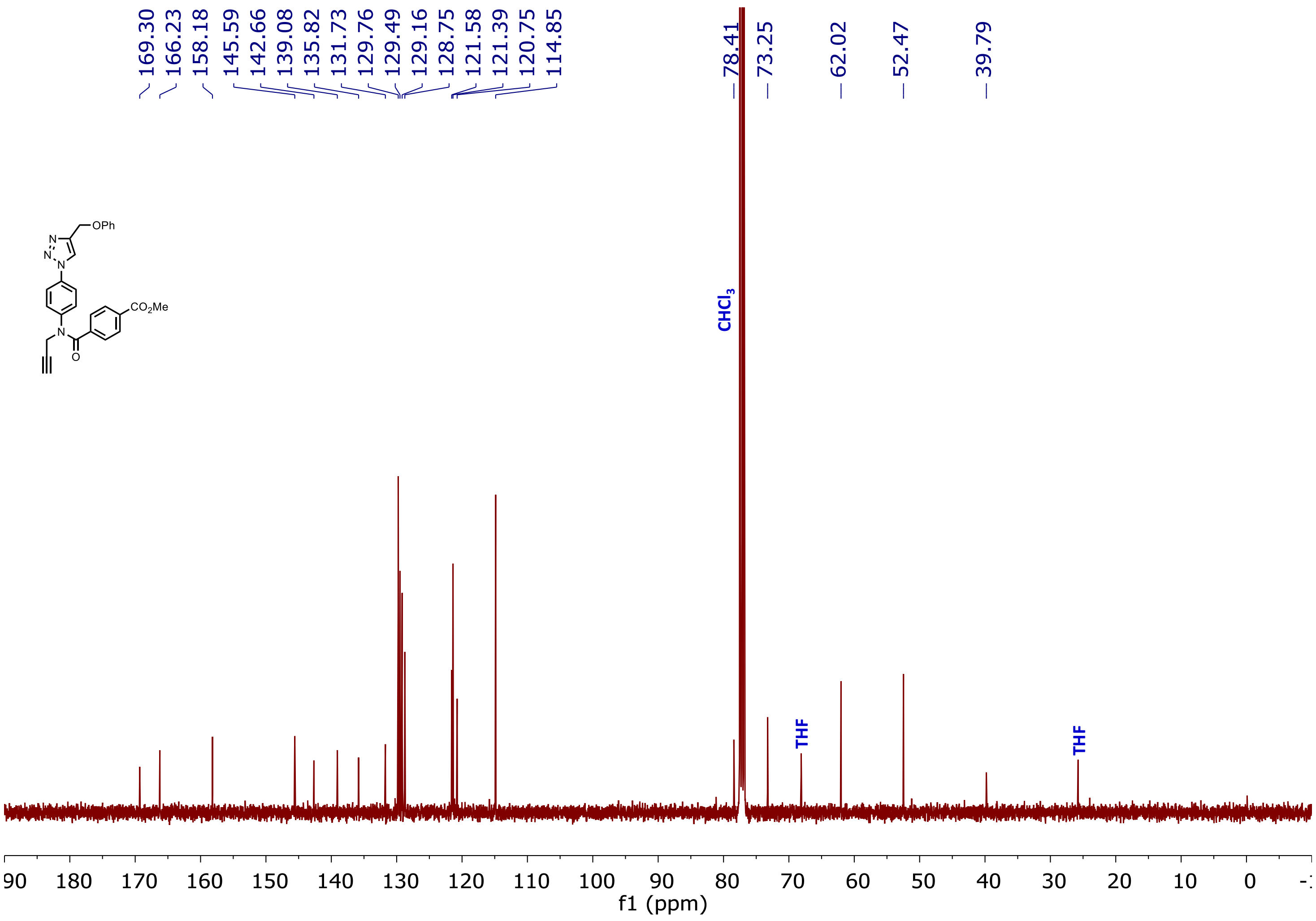

Compound 19.

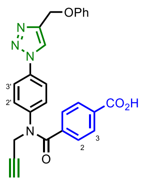

17 (0.145 g, 0.27 mmol) was dissolved in THF/ H_2O 3:1 (4 mL) and LiOH (0.046 g, 1.09 mmol) was added. After 3 h of stirring at room temperature, the crude was diluted with H_2O and acidified with 1M HCl soln. to pH 2-3. The aqueous phase was extracted with EtOAc (3x) and the combined organic phase was washed with brine, dried over $MgSO_4$ and evaporated to dryness. The obtained material was purified by flash column chromatography on silica gel (gradient from 0% to 60% of EtOAc in Pet. Ether) to afford **19** (0.117 g, 95%) as a colorless oil.

^{1}H NMR (400 MHz, $CDCl_3$): δ_H = 8.02 (s, 1H, CH_{triaz}), 7.96 (d, 2H, J = 8.0 Hz, 2-H), 7.68 (d, 2H, J = 9.0 Hz, 3'-H), 7.47 (d, 2H, J = 8.0 Hz, 3-H), 7.30 (m, 4H, 2'-H and 3''-H, PhO), 7.00 (m, 3H, 2''-H and 4''-H, PhO), 5.31 (s, 2H, O-CH_2), 4.75 (d, 2H, J = 2.0 Hz, N-CH_2), 3.87 (s, 3H, O-CH_3), 2.33 (t, 1H, J = 2.0 Hz, CH, alkyne).

^{13}C NMR (100.6 MHz, $CDCl_3$): δ_C = 170.2 and 169.2 (CO), 158.1 (1''-C, PhO), 145.6 (C_{triaz}), 142.5 (1'-C), 139.8 (1-C), 135.8 (4'-C), 131.0 (4-C), 130.1, 129.7, 129.2 and 128.8 (2-C, 3-C, 2'-C and 3''-C, PhO), 121.6, 121.4 and 120.8 (CH_{triaz}, 3'-C and 4''-C, PhO), 114.8 (2''-C, PhO), 78.3 (C, alkyne), 73.3 (CH, alkyne), 62.0 (O-CH_2), 39.8 (N-CH_2).

HRMS (ES+): calcd for $C_{26}H_{21}N_4O_4$ 453.1563 $[M+H]^+$, found 453.1508 $[M+H]^+$.

FT-IR (ATR): ν_{max} 3296, 2923, 1713, 1650, 1519, 1231, 753 and 738cm^{-1}.

^{1}H-NMR (400 MHz, $CDCl_3$) Compound 19.

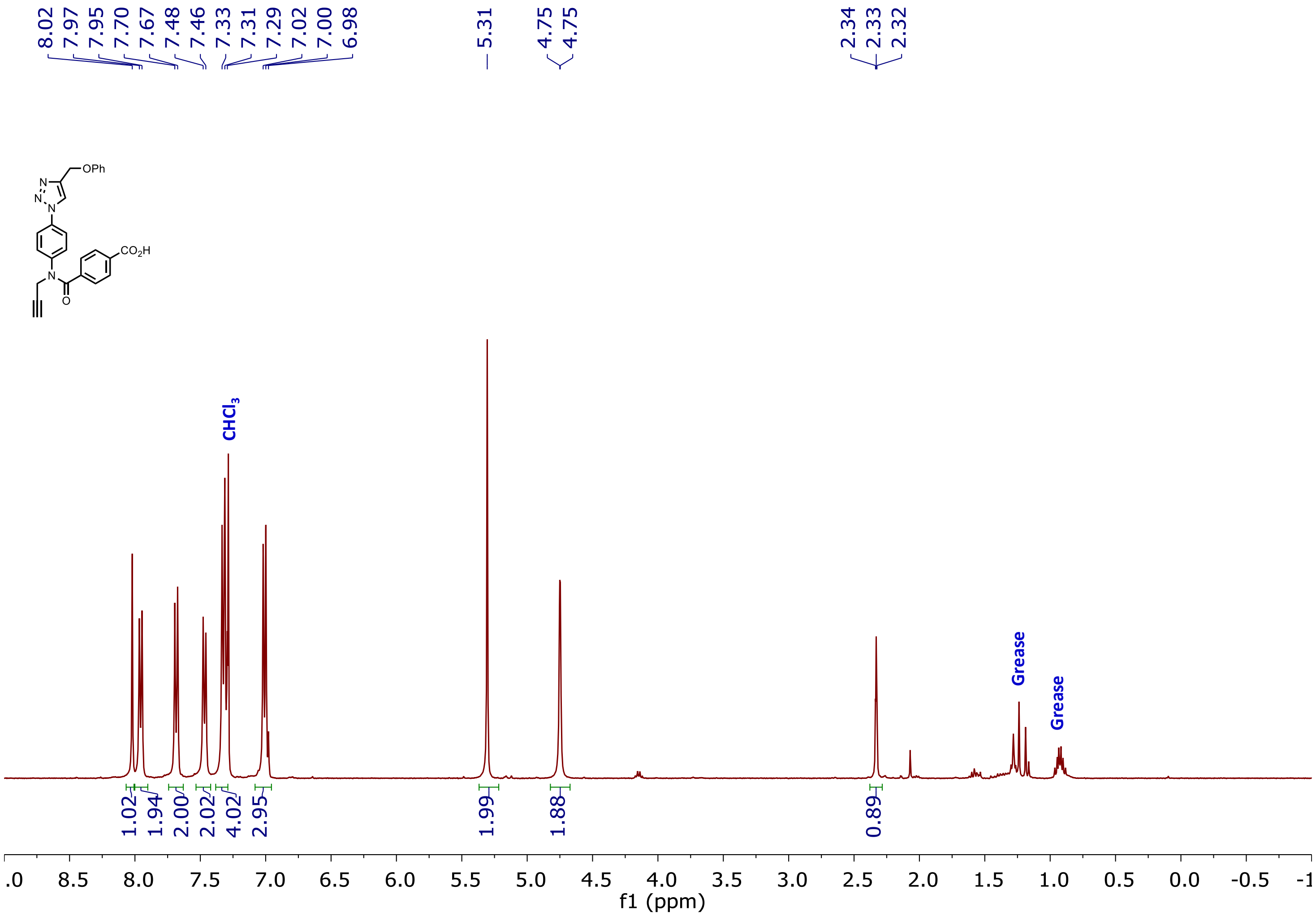

^{13}C-NMR (100.6 MHz, $CDCl_3$) Compound 19.

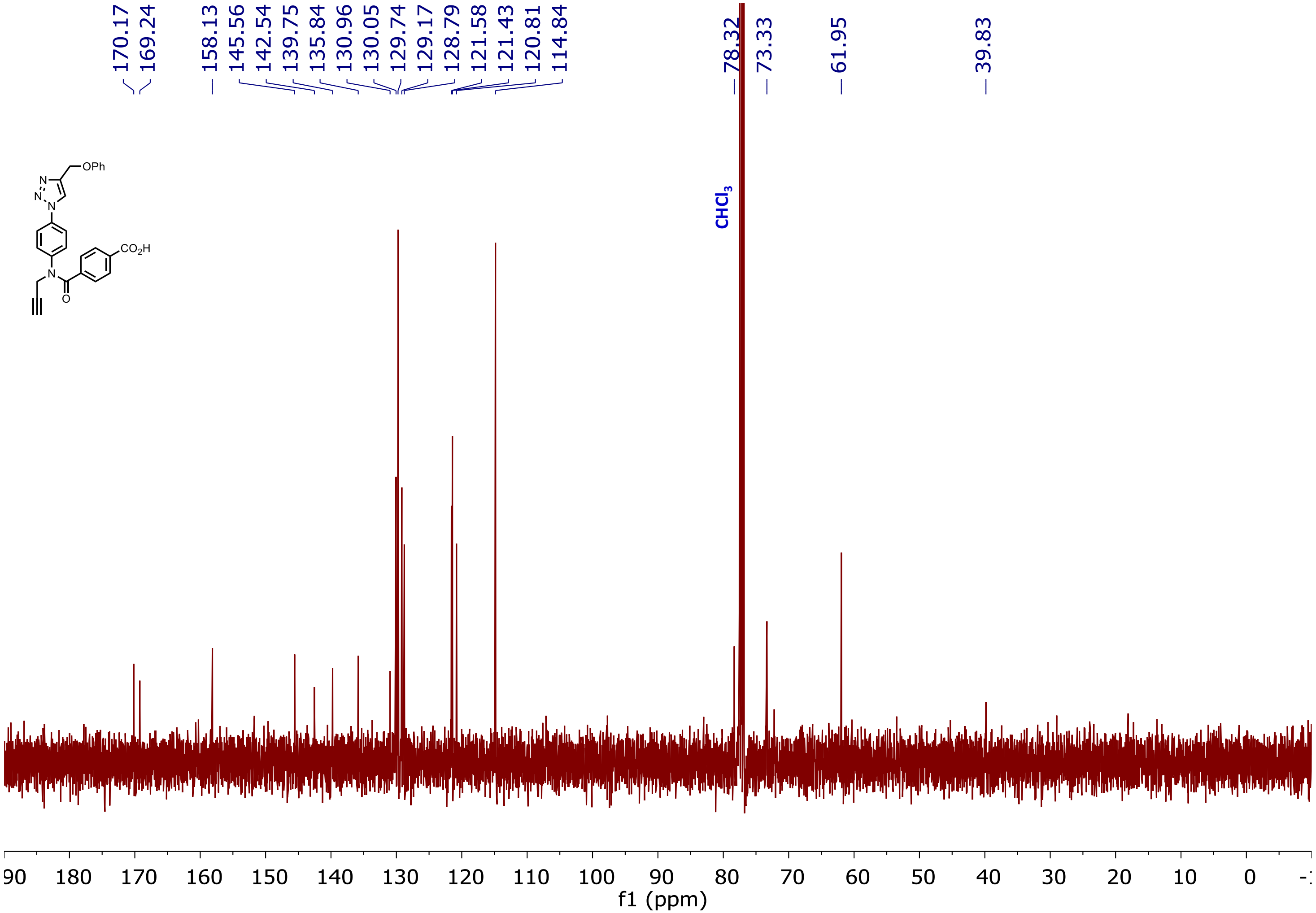

Compound 20.

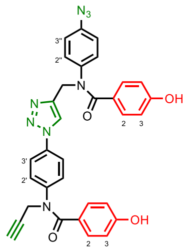

Storage at room temperature of **9** (0.063 g, 0.22 mmol) results in a mixture of oligomers, purified by flash column chromatography on silica gel (gradient from 10% to 100% of EtOAc in Pet. Ether) to afford **20** (0.013 g, 10 %) as a waxy white solid.

^{1}H NMR (400 MHz, DMSO-*d*$_6$): δ_H = 9.90 (s, 1H, OH), 9.84 (s, 1H, OH), 8.68 (s, 1H, CH_{triaz}), 7.84 (d, 2H, *J* = 9.0 Hz, 3'-H), 7.34 (d, 2H, *J* = 9.0 Hz, 2'-H), 7.23-7.15 (m, 6H, 2-H and 2''-H), 6.98 (d, 2H, *J* = 8.5 Hz, 3''-H), 6.59 (m, 4H, 3-H), 5.11 (s, 2H, N-CH_2), 4.66 (d, 2H, *J* = 2.5 Hz, N-CH_2), 3.16 (t, 1H, *J* = 2.5 Hz, alkyne).

^{13}C NMR (100.6 MHz, DMSO-*d*$_6$): δ_C = 169.2 and 169.0 (CO, amide), 159.2 and 158.8 (4-C), 144.7 (C_{triaz}), 143.0 (1'-C), 140.6 (1''-C), 137.8 (4''-C), 134.5 (4'-C), 130.9 and 130.8 (2-C), 129.2 (2'-C), 128.8 (2''-C), 125.9 and 125.3 (1-C), 121.6 (CH_{triaz}), 120.5 (3'-C), 119.6 (3'-C), 114.6 and 114.4 (3-C), 79.7 (C, alkyne), 74.8 (CH, alkyne), 45.5 (N-CH_2), 39.1 (N-CH_2, partially overlapped).

HRMS (ES+): calcd for $C_{32}H_{25}N_8O_4$ 585.1999 $[M+H]^+$, found 585.1991 $[M+H]^+$.

FT-IR (ATR): ν_{max} 3207, 2123, 2097, 1637, 1607, 1514, 1506, 1379, 1279, 1231 and 845 cm^{-1}.

^{1}H-NMR (400 MHz, DMSO-d_6) Compound 20.

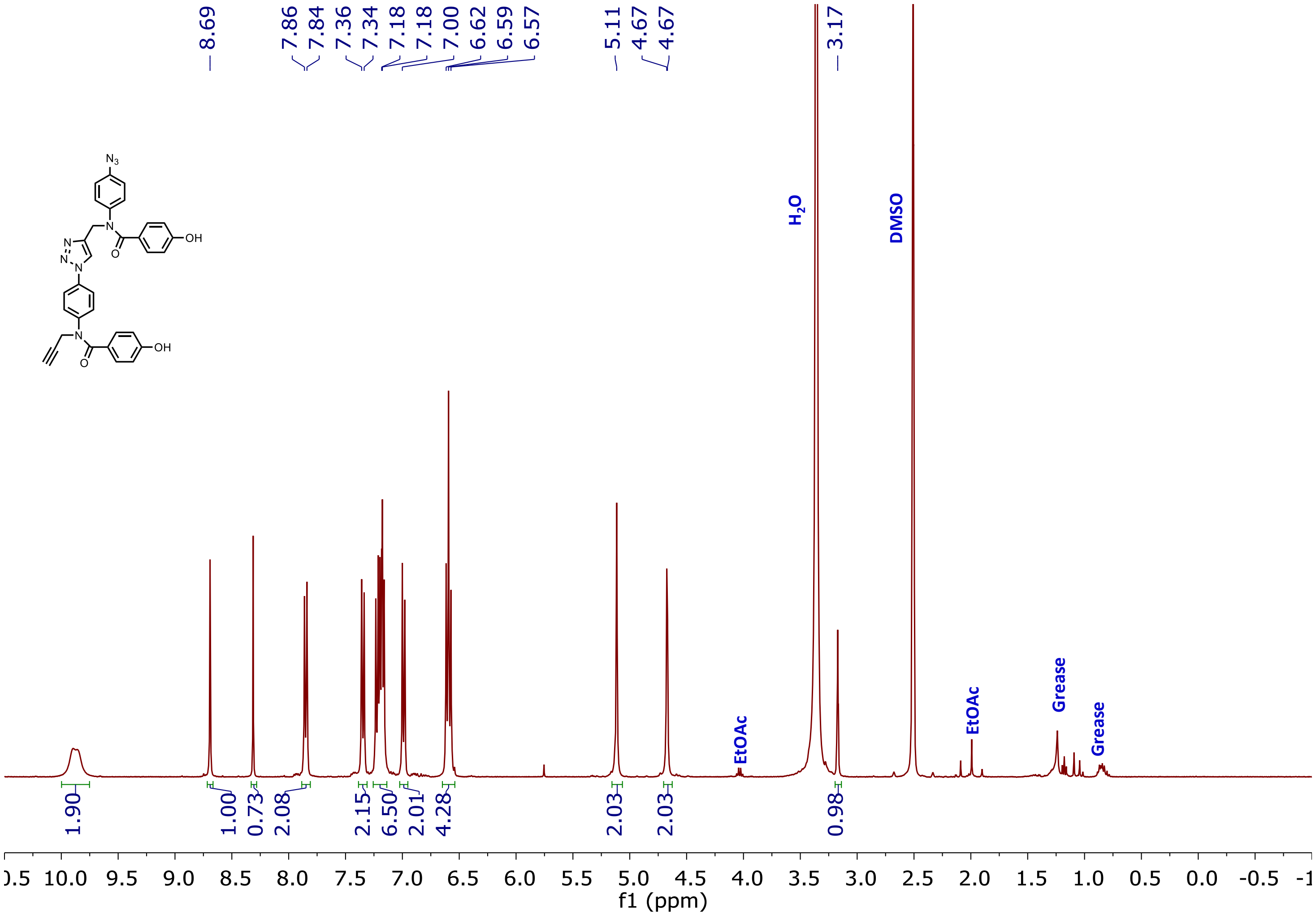

^{13}C-NMR (100.6 MHz, DMSO-d_6) Compound 20.

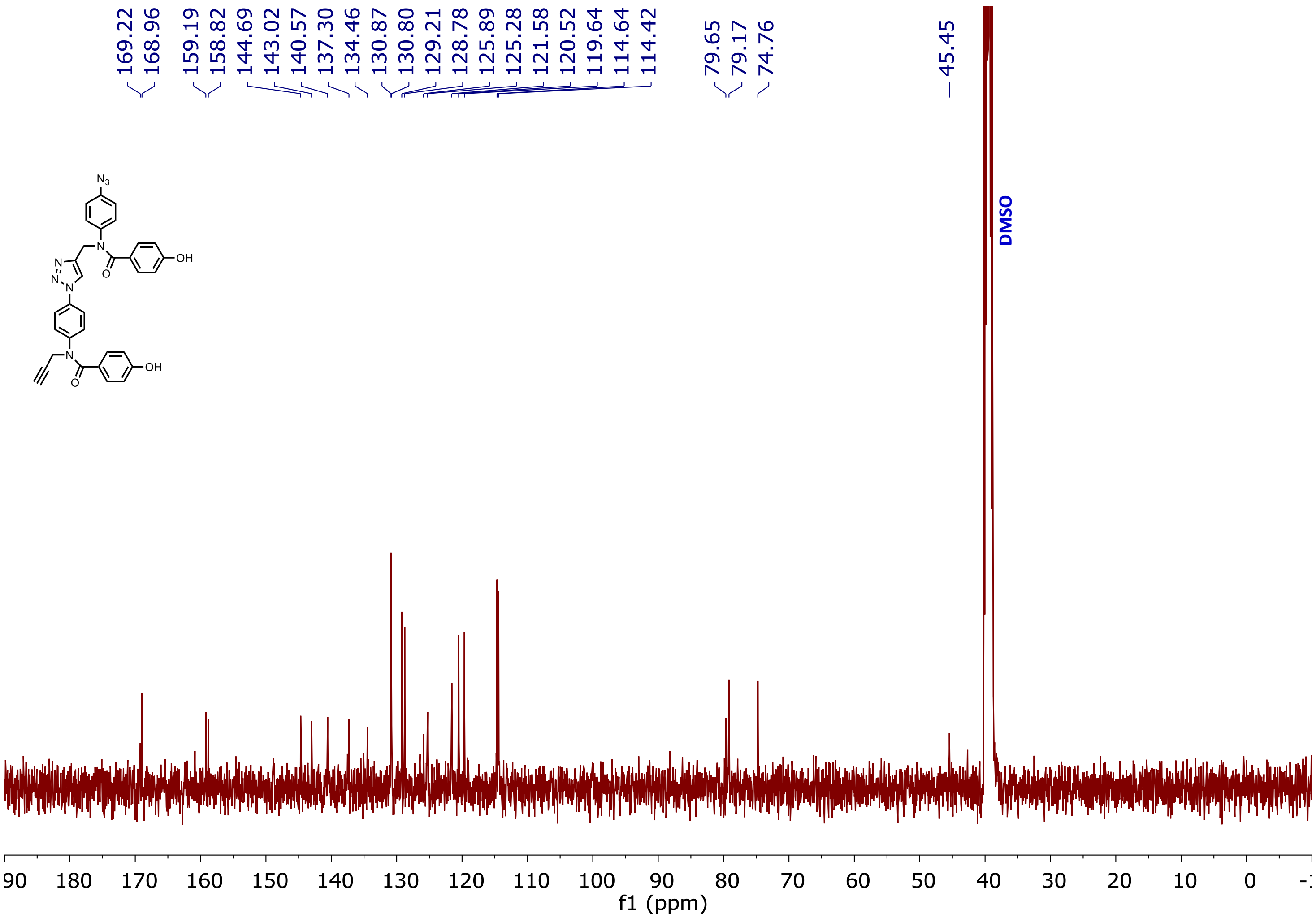

Compound 22 (PPA 3-mer).

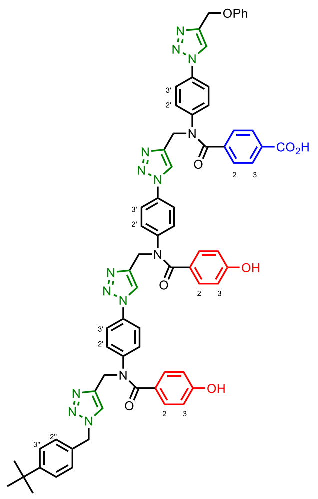

20 (0.005 g, 0.009 mmol), 1-(azidomethyl)-4-*tert*-butylbenzene (0.162 g, 0.865 mmol), $Cu(CH_3CN)_4PF_6$ (0.003 g, 0.009 mmol) and TBTA (0.004 g, 0.009 mmol) were mixed in a round-bottom flask and, under N_2, THF (50 mL) was added. The reaction was stirred overnight at room temperature. The solvent was evaporated to dryness and the residue purified by flash chromatography on silica gel (gradient from 0% to 100% of EtOAc in Pet. Ether) to afford the corresponding capped 2-mer **21** (0.004 g, 53%). The obtained 2-mer **21** (0.004 g, 0.005 mmol), **19** (0.002 g, 0.005 mmol), $Cu(CH_3CN)_4PF_6$ (0.002 g, 0.005 mmol) and TBTA (0.002 g, 0.005 mmol) were mixed in a round-bottom flask and, under N_2, THF (1 mL) was added. The reaction was stirred overnight at room temperature. The reaction was then diluted with EtOAc and washed with 0.02M EDTA soln. (2x), H_2O (1x) and brine. The organic layer was dried over $MgSO_4$ and concentrate under vacuum. The residue was purified by flash column chromatography on silica gel (gradient from 0% to 20% of MeOH in CH_2Cl_2) to afford compound **22** (0.005 g, 90%) as a waxy solid.

^{1}H NMR (500 MHz, DMSO-*d*$_6$): δ_H = 9.91 (s, 2H, OH), 8.86 (s, 1H, CH_{triaz}), 8.74 (s, 1H, CH_{triaz}), 8.70 (s, 1H, CH_{triaz}), 8.05 (s, 1H, CH_{triaz}), 7.78 (m, 8H, 3-H, acid; 3'-H), 7.45 (m, 4H, 2'-H), 7.40 (d, 2H, *J* = 9.0 Hz, 2'-H), 7.28 (m, 6H, 2-H, acid; 3''-H, PhO; 3''-H, ^{t}BuPh), 7.21 (d, 2H, *J* = 8.5 Hz, 2-H, phenol), 7.17 (d, 2H, *J* = 8.5 Hz, 2-H, phenol), 7.08 (d, 2H, *J* = 8.5 Hz, 2''-H, ^{t}BuPh), 7.03 (d, 2H, *J* = 8.0 Hz, 2''-H, PhO), 6.95 (t, 1H, *J* = 7.5 Hz, 4''-H, PhO), 6.59 (d, 2H, *J* = 8.5 Hz, 3-H, phenol), 6.58 (d, 2H, *J* = 8.5 Hz, 3-H, phenol), 5.48 (s, 2H, N-CH_2), 5.21 (s, 2H, N-CH_2), 5.17 (s, 4H, N-CH_2), 5.10 (s, 2H, N-CH_2, phenol), 1.17 (s, 9H, ^{t}Bu).

^{13}C NMR (125 MHz, DMSO-*d*$_6$): δ_C = 169.3 and 169.2 (CO, amide phenol), 168.9 (CO, amide acid), 166.9 (CO, acid), 159.1 and 159.0 (4-C, phenol), 157.9 (1''-C, PhO), 150.5 (4''-C, ^{t}BuPh), 144.6, 144.2, and 144.0 (C_{triaz}), 143.7 and 143.6 (1'-C), 143.4 (C_{triaz}), 142.6 (1'-C), 139.2 (1-C, acid), 134.7, 134.2 and 134.0 (4'-C), 133.2 (4-C, acid), 130.9 and 130.8 (2-C, phenol), 129.5, 129.2, 128.8, 128.8, 128.7, 128.4, 128.0 and 127.3 (C_{arom}), 125.7 and 125.7 (1-C, phenol), 125.4 (3''-C, ^{t}BuPh), 124.0, 122.8, 121.6 and 121.5 (CH_{triaz}), 121.0 (4''-C, PhO), 120.6, 120.5 and 120.3 (3'-C), 114.7 (2''-C, PhO), 114.6 and 114.5 (3-C, phenol), 60.9 (O-CH_2), 52.4 (N-CH_2), 45.4, 45.3 and 45.1 (N-CH_2), 34.2 (C, ^{t}Bu), 31.0 (CH_3, ^{t}Bu).

HRMS (ES+): calcd for $C_{69}H_{60}N_{15}O_8$ 1226.4749 $[M+H]^+$, found 1226.4794 $[M+H]^+$.

FT-IR (ATR): ν_{max} 3139, 2963, 2920, 1639, 1606, 1517, 1279, 1238, 1045 and 845 cm^{-1}.

^{1}H-NMR (500 MHz, DMSO-d_6) Compound 22 (PPA 3-mer).

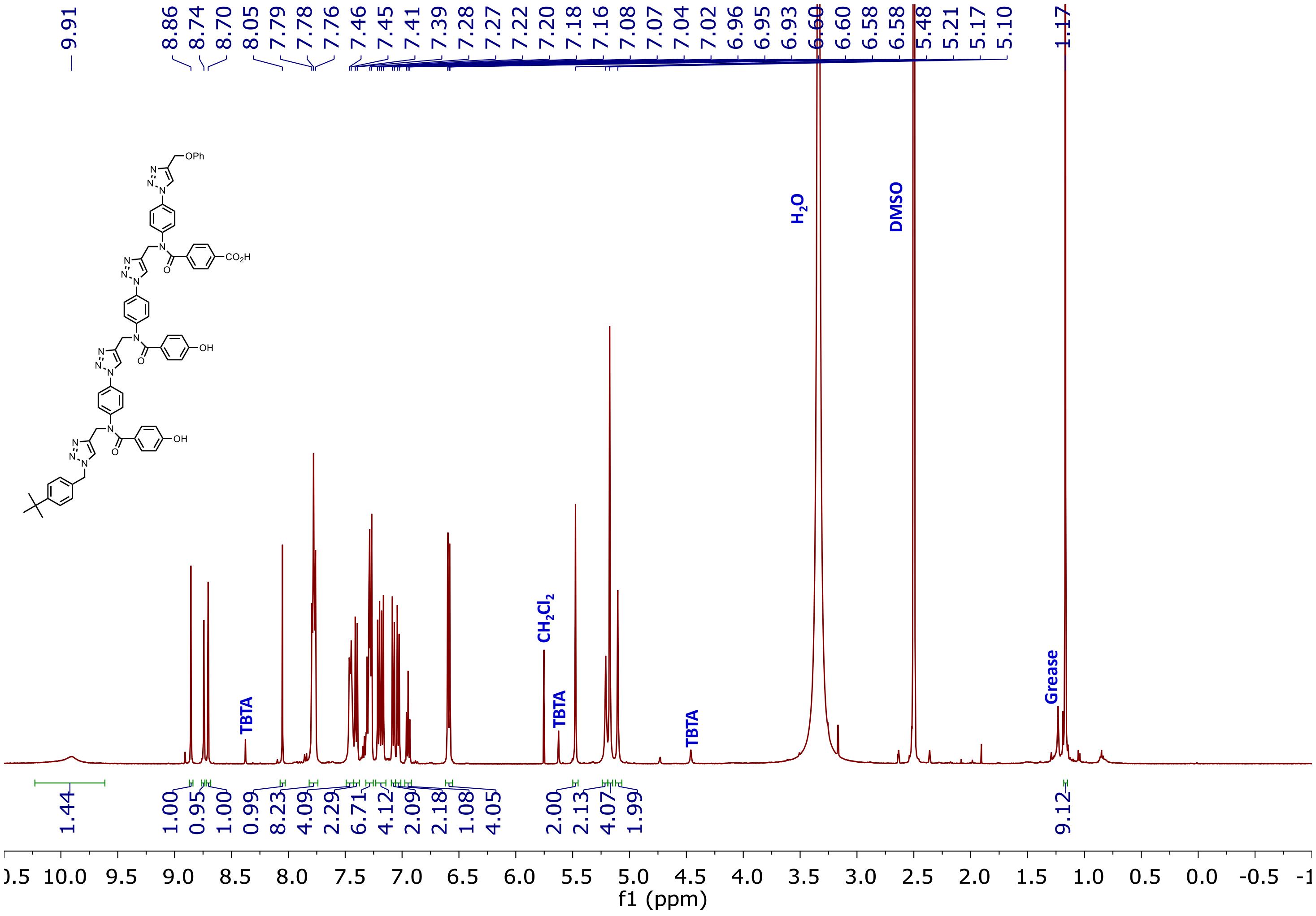

^{13}C-NMR (125MHz, DMSO-d_6) Compound 22 (PPA 3-mer).

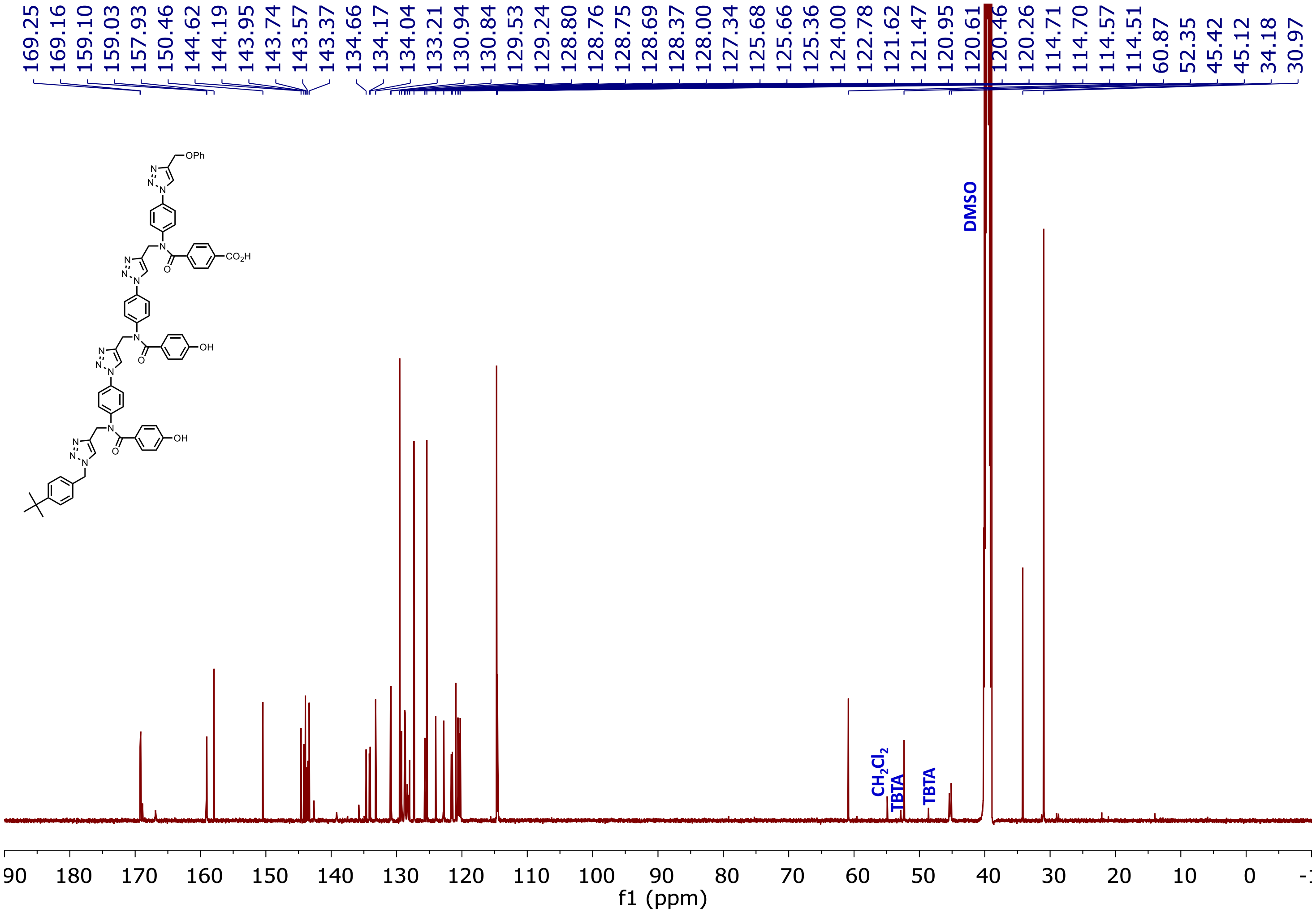

COSY (DMSO-d_6) Compound 22 (PPA 3-mer).

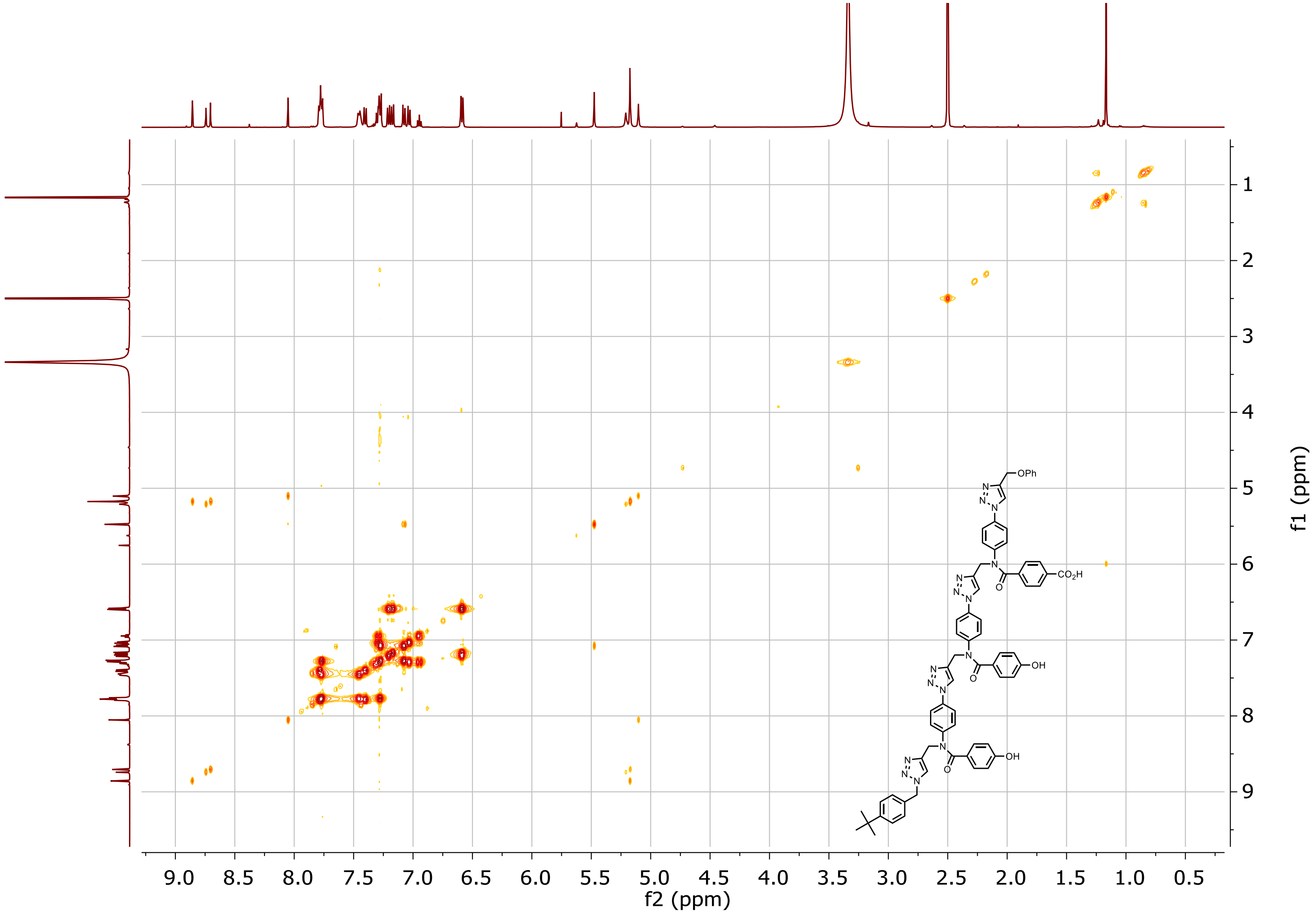

HSQC (DMSO-d_6) Compound 22 (PPA 3-mer).

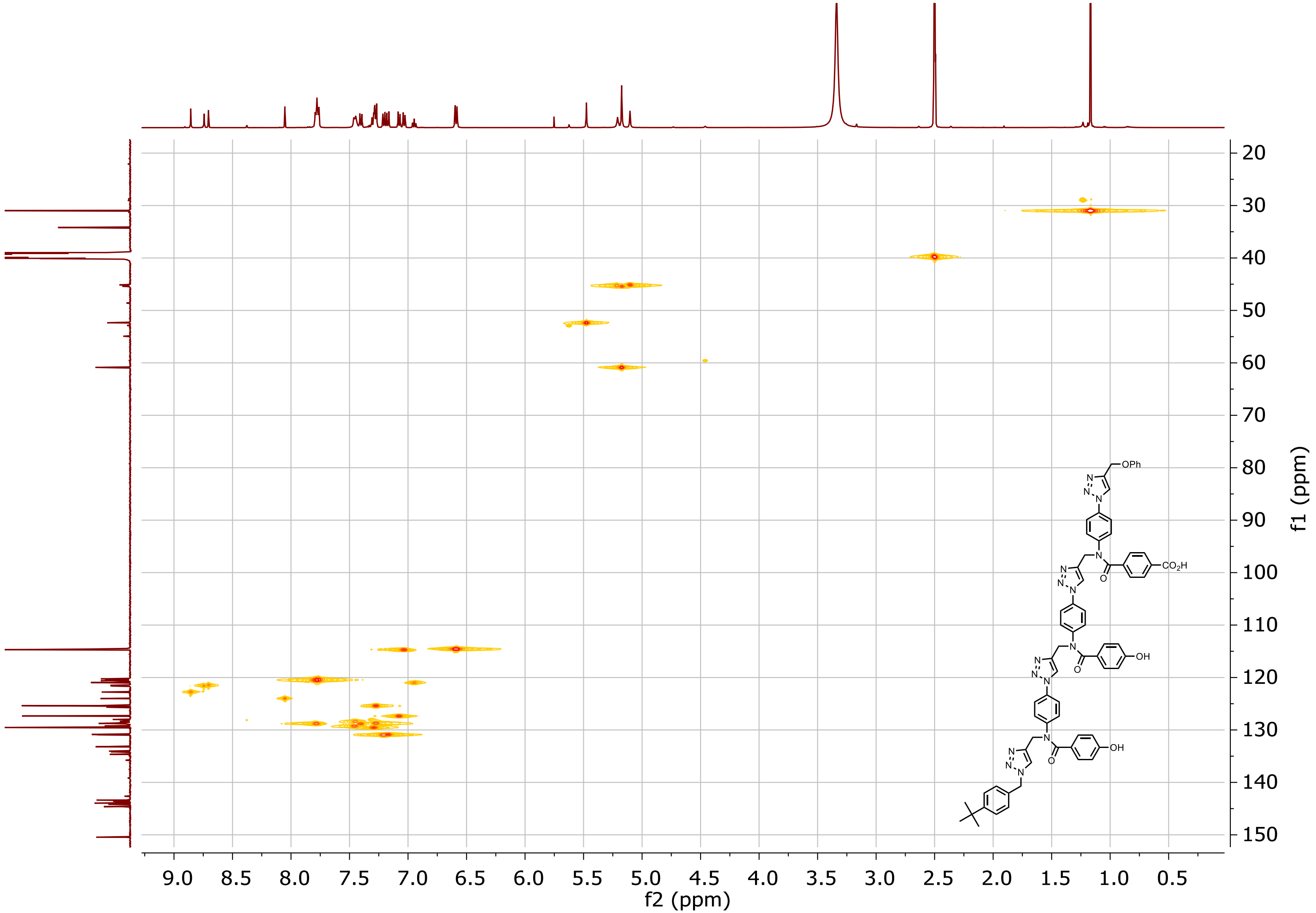

HMBC (DMSO-d_6) Compound 22 (PPA 3-mer).

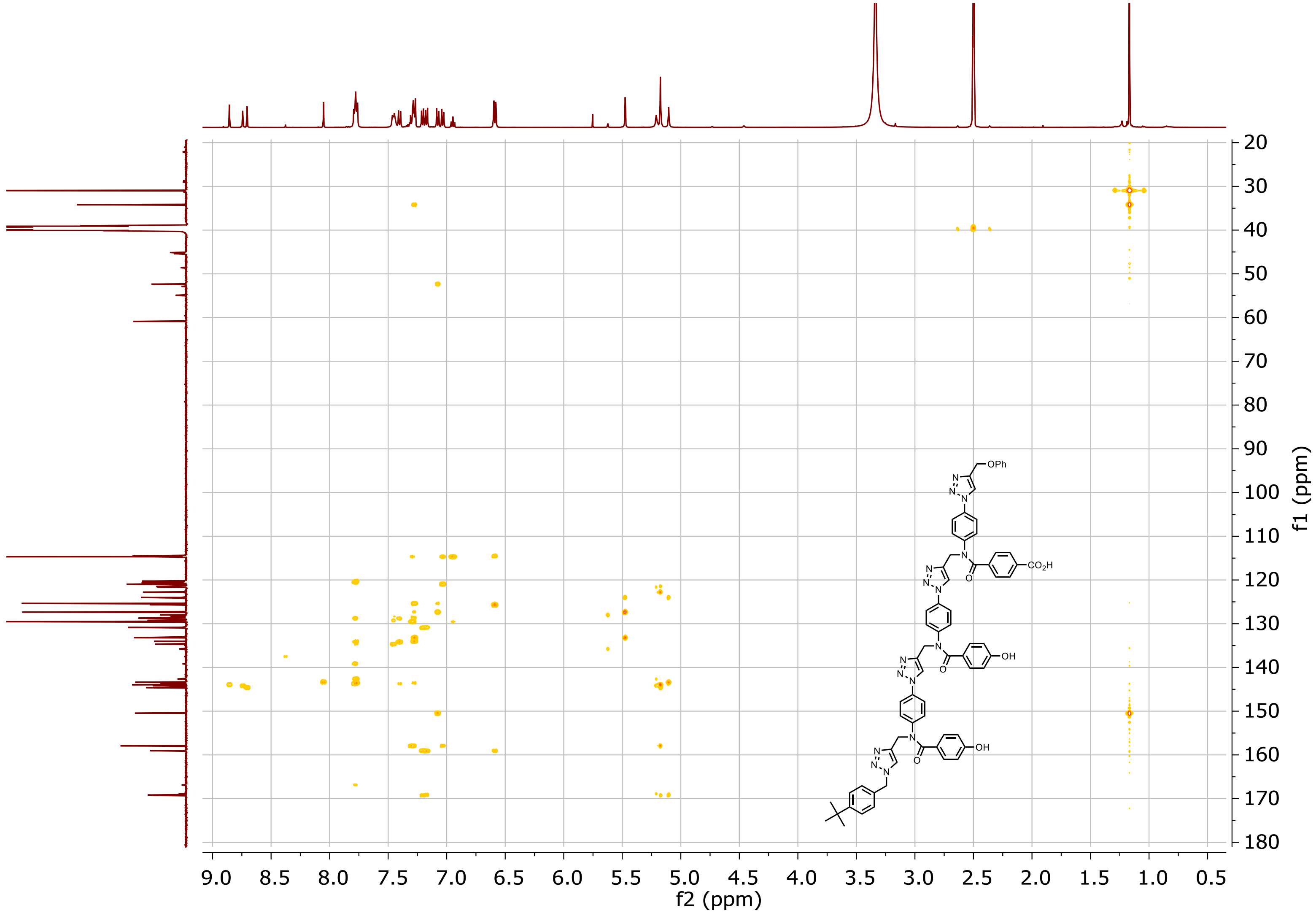

LCMS Compound 22 (PPA 3-mer).

Conditions: C18 column at 40 °C (254 nm) using water + 0.1% formic acid (A) and CH_3CN + 0.1% formic acid (B) with flow rate of 0.6 ml/min; Gradient of 0-2 minutes 5% -100%B + 1 minute 100% B.

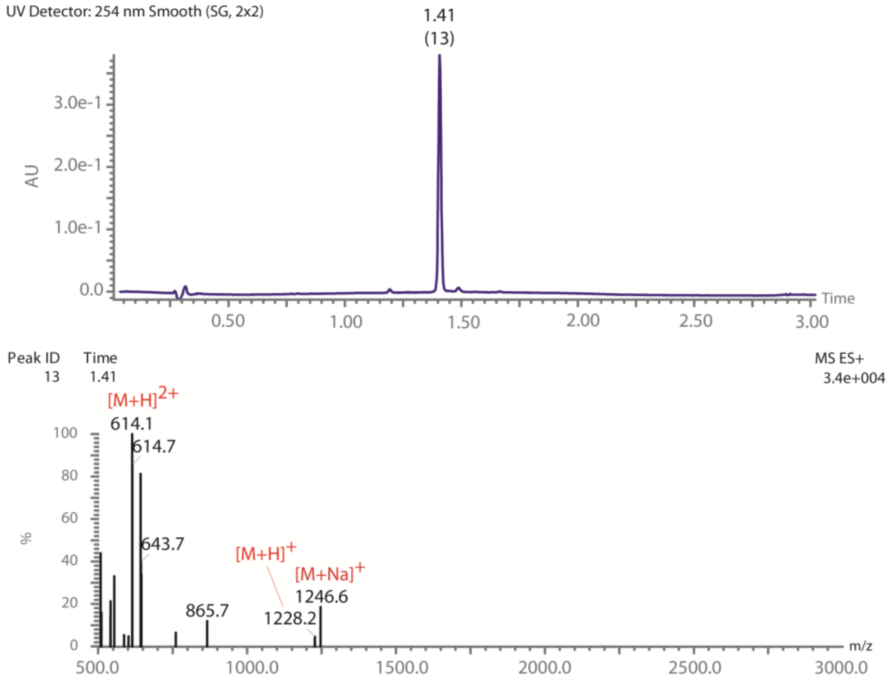

HRMS Compound 22 (PPA 3-mer).

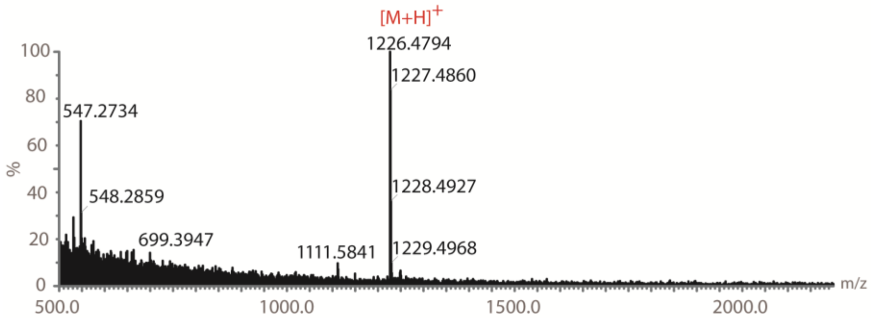

Synthesis of PAP 3-mer[S2]

As shown in Scheme S11, CuAAC coupling of previously described intermediate **11** with protected phenol 1-mer 4 afforded 3-mer **23** in moderate yield but with recovery on unreacted staring material. CuAAC capping of **23** with *p-tert*-butylbenzylazide and subsequent LiOH-mediated basic hydrolysis of the obtained capped product yielded template **24** in good yield.

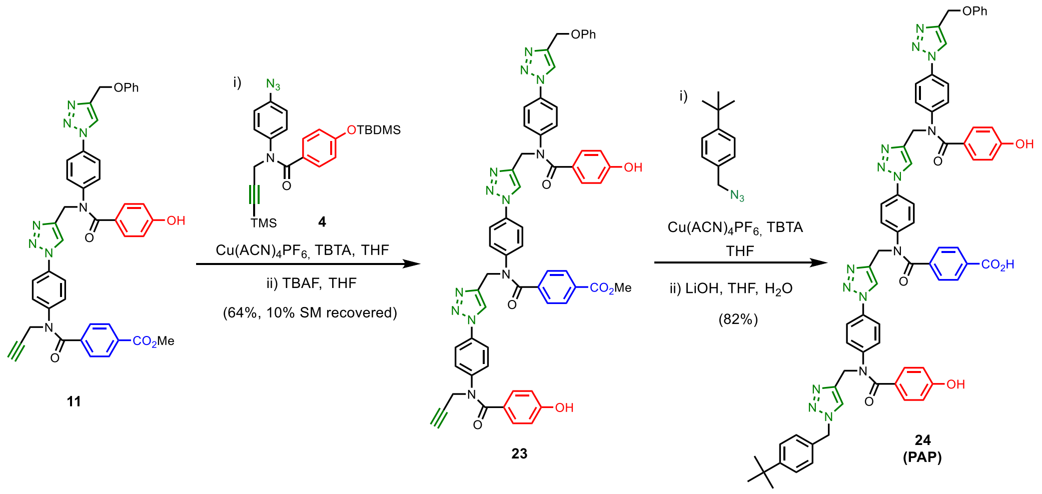

Scheme S11

Compound 23.

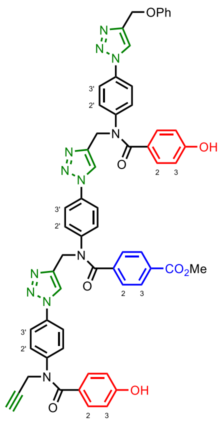

11 (0.085 g, 0.11 mmol), **4** (0.054 g, 0.11 mmol), $Cu(CH_3CN)_4PF_6$ (0.004 g, 0.01 mmol) and TBTA (0.006 g, 0.01 mmol) were mixed in a round-bottom flask and, under N_2, THF (3 mL) was added. The reaction was stirred overnight at room temperature. Once the reaction was completed, TBAF (1M in THF, 0.220 mL, 0.22 mmol) was added dropwise. The solution was stirred for 10 min at room temperature, quenched with 5% soln. HCl and extracted with EtOAc (3x) followed by washing with H_2O and brine. The organic layer was dried over anhydrous $MgSO_4$ and concentrate under vacuum. The crude was purified by flash column chromatography on silica gel (gradient from 0% to 100% of EtOAc in Pet. Ether) to afford compound **23** (0.075 g, 64%) as a foam, and recovered starting material **11** (0.009 g, 10%).

^{1}H NMR (500 MHz, $CDCl_3$): δ_H = 8.11 (s, 1H, CH_{triaz}), 8.07 (s, 1H, CH_{triaz}), 7.99 (s, 1H, CH_{triaz}), 7.85 (d, 2H, J = 8.0 Hz, 3-H, ester), 7.62 (d, 2H, J = 9.0 Hz, 3'-H), 7.57 (d, 2H, J = 9.0 Hz, 3'-H), 7.54 (d, 2H, J = 9.0 Hz, 3'-H), 7.39 (d, 2H, J = 8.0 Hz, 2-H, ester), 7.32-7.24 (m, 6H, 2'-H and 3''-H, PhO), 7.19 (m, 4H, 2-H, phenol and 2'-H), 7.14 (d, 2H, J = 8.5 Hz, 2-H, phenol), 6.97 (m, 3H, 2''-H and 4''-H, PhO), 6.57 (m, 4H, 3-H, phenol), 5.26 (m, 4H, O-CH_2 and N-CH_2), 5.16 (s, 2H, N-CH_2), 4.68 (d, 2H, J = 2.5 Hz, N-CH_2), 3.84 (s, 3H, O-CH_3), 2.25 (t, 1H, J = 2.5 Hz, alkyne).

^{13}C NMR (125 MHz, $CDCl_3$): δ_C = 170.6 and 170.3 (CO, amide phenol), 169.8 (CO, amide ester), 166.3 (CO, ester), 158.9 (4-C, phenol), 158.2 (1''-C), 145.4 (C_{triaz}), 144.8 (1'-C), 144.7 and 144.2 (C_{triaz}), 143.9 and 143.0 (1'-C), 139.2 (1-C, ester), 135.4 and 135.1 (4'-C), 134.9 (4'-C), 131.8 (4-C, ester), 131.4 (2-C, phenol), 129.8, 129.5, 129.0, 128.9, 128.8 and 128.5 (2-C and 3-C, ester; 2'-C; 3''-C, PhO), 125.9 and 125.9 (1-C, phenol), 122.2 and 121.9 (CH_{triaz}), 121.6 (4''-C, PhO), 121.5 (3'-C), 121.3 (3'-C), 121.2 (CH_{triaz}), 115.3 and 115.1 (3-C, phenol), 114.9 (2''-C, PhO), 78.9 (C, alkyne), 72.9 (CH, alkyne), 61.9 (O-CH_2), 52.5 (O-CH_3), 46.6 (N-CH_2), 45.8 (N-CH_2), 39.9 (N-CH_2).

HRMS (ES+): calcd for $C_{59}H_{46}N_{12}O_8Na$ 1073.3459 $[M+H]^+$, found 1073.3857 $[M+H]^+$.

FT-IR (ATR): ν_{max} 2955, 2925, 1720, 1638, 1604, 1517, 1278, 1239, 844 and 754cm^{-1}.

^{1}H-NMR (500 MHz, $CDCl_3$) Compound 23.

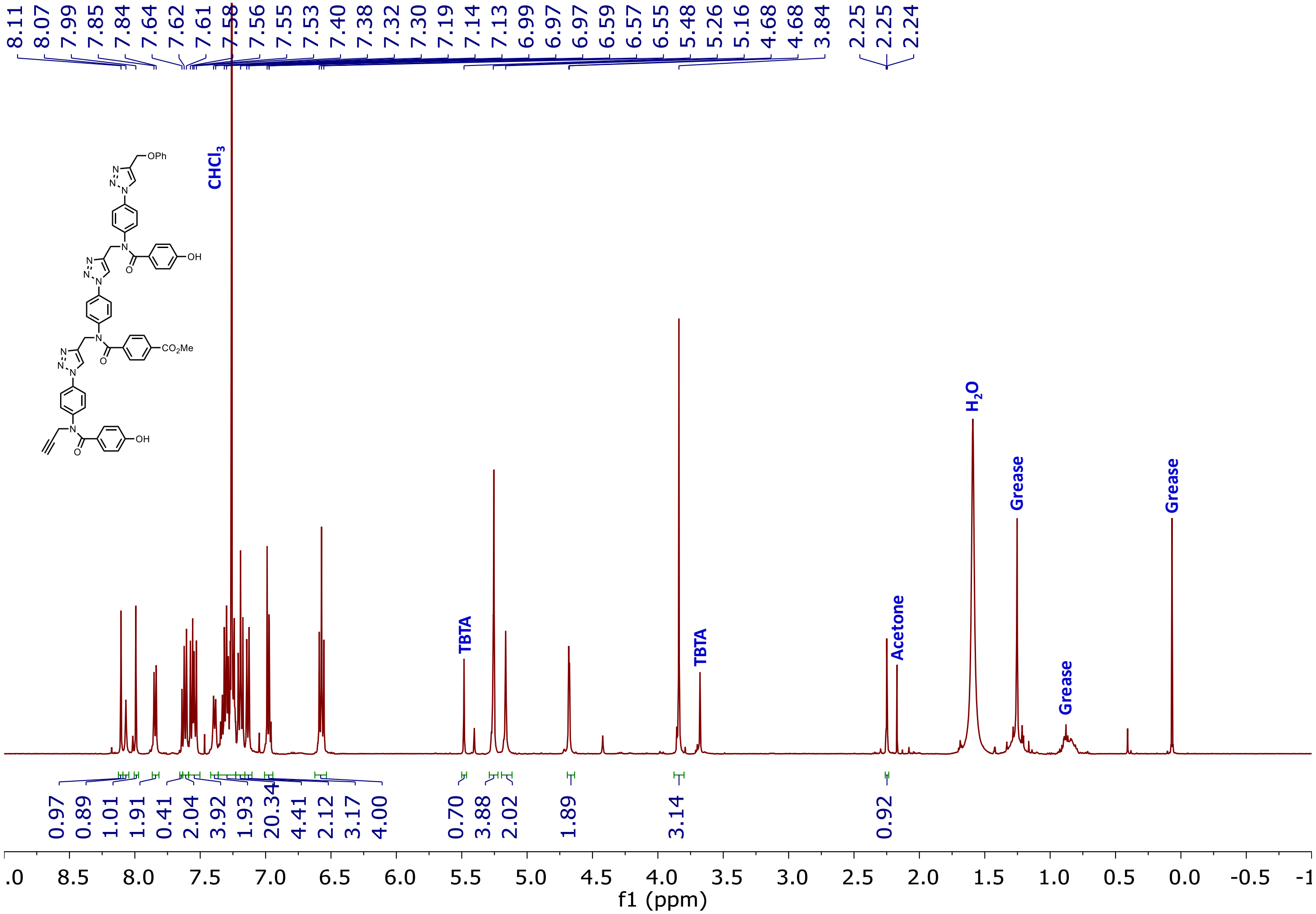

^{13}C-NMR (125MHz, $CDCl_3$) Compound 23.

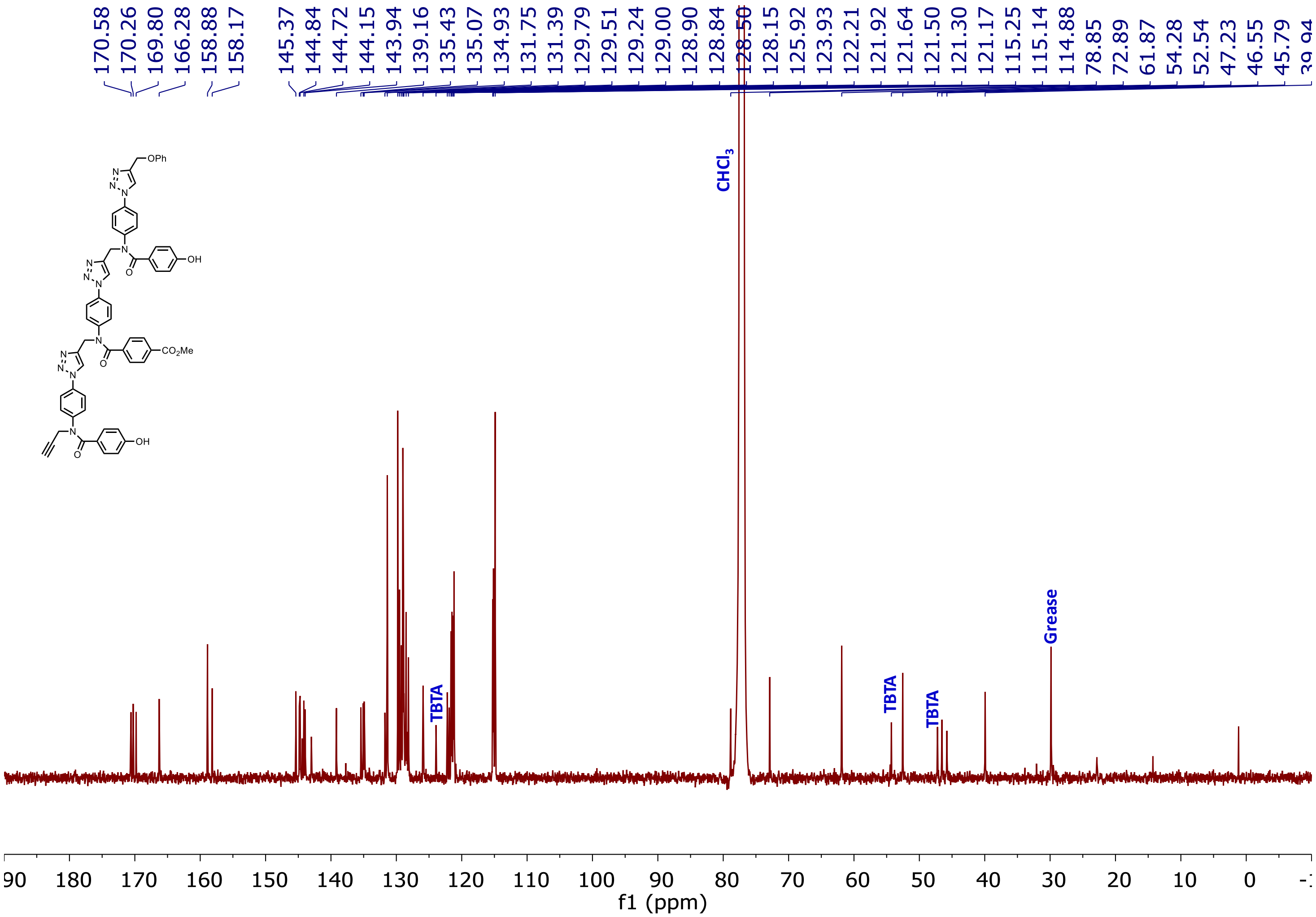

Compound 24 (PPA 3-mer).

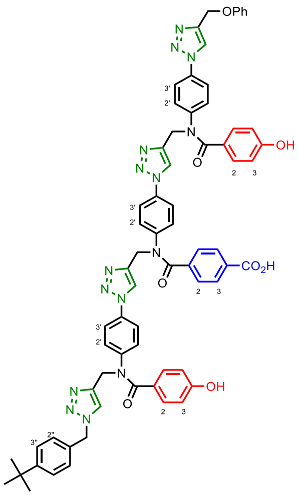

23 (0.071 g, 0.07 mmol), 1-(azidomethyl)-4-*tert*-butylbenzene (0.026 g, 0.14 mmol), $Cu(CH_3CN)_4PF_6$ (0.005 g, 0.01 mmol) and TBTA (0.007 g, 0.01 mmol) were mixed in a round-bottom flask and, under N_2, THF (2 mL) was added. The reaction was stirred overnight at room temperature. The reaction was then diluted with EtOAc and washed with 0.02M EDTA soln. (2x), H_2O (1x) and brine. The organic layer was dried over $MgSO_4$ and concentrate under vacuum. The crude was dissolved in THF:H_2O 3:1 (2 mL) and LiOH (0.014 g, 0.34 mmol) was added. The reaction was stirred at room temperature for 2h. Then, the crude was diluted with H_2O and acidified with 1M HCl soln. to pH 2-3. The aqueous phase was extracted with EtOAc (3x) and the combined organic phase was washed with brine, dried over $MgSO_4$ and evaporated to dryness. The residue was purified by flash column chromatography on silica gel (gradient from 0% to 10% of MeOH in CH_2Cl_2) to afford **24** (0.068 g, 82%) as a white solid.

mp: 188-190 °C.

^{1}H NMR (500 MHz, DMSO-*d*$_6$): δ_H = 9.91 (s, 2H, OH), 8.85 (s, 1H, CH_{triaz}), 8.74 (s, 1H, CH_{triaz}), 8.66 (s, 1H, CH_{triaz}), 8.05 (s, 1H, CH_{triaz}), 7.77 (m, 8H, 3-H, acid; 3'-H), 7.44 (m, 4H, 2'-H), 7.39 (d, 2H, *J* = 9.0 Hz, 2'-H), 7.29 (m, 6H, 2-H, acid; 3''-H, PhO; 3''-H, ^{t}BuPh), 7.19 (d, 2H, *J* = 8.5 Hz, 2-H, phenol), 7.18 (d, 2H, *J* = 8.5 Hz, 2-H, phenol), 7.07 (d, 2H, *J* = 8.5 Hz, 2''-H, ^{t}BuPh), 7.04 (d, 2H, *J* = 8.0 Hz, 2''-H, PhO), 6.95 (t, 1H, *J* = 7.5 Hz, 4''-H, PhO), 6.59 (d, 2H, *J* = 8.5 Hz, 3-H, phenol), 6.58 (d, 2H, *J* = 8.5 Hz, 3-H, phenol), 5.47 (s, 2H, N-CH_2), 5.20 (s, 2H, N-CH_2), 5.18 (s, 2H, N-CH_2), 5.15 (s, 2H, N-CH_2), 5.11 (s, 2H, N-CH_2), 1.16 (s, 9H, ^{t}Bu).

^{13}C NMR (125 MHz, DMSO-*d*$_6$): δ_C = 169.3 and 169.3 (CO, amide phenol), 168.9 (CO, amide acid), 166.9 (CO, acid), 159.1 (4-C, phenol), 158.0 (1''-C, PhO), 150.6 (4''-C, ^{t}BuPh), 144.7, 144.2, and 144.0 (C_{triaz}), 143.9 and 143.6 (1'-C), 143.4 (C_{triaz}), 142.5 (1'-C), 139.5 (1-C, acid), 134.8, 134.2 and 134.1 (4'-C), 133.3 (4-C, acid), 131.0 and 130.9 (2-C, phenol), 129.6, 129.3, 128.9, 128.8, 128.5 and 127.4 (C_{arom}), 125.8 and 125.7 (1-C, phenol), 125.4 (3''-C, ^{t}BuPh), 124.1, 122.8, 121.7 and 121.6 (CH_{triaz}), 121.1 (4''-C, PhO), 120.6, 120.5 and 120.4 (3'-C), 114.8 (2''-C, PhO), 114.6 and 114.6 (3-C, phenol), 60.9 (O-CH_2), 52.4 (N-CH_2), 45.4, 45.3 and 45.2 (N-CH_2), 34.4 (C, ^{t}Bu), 31.0 (CH_3, ^{t}Bu).

HRMS (ES+): calcd for $C_{69}H_{60}N_{15}O_8$ 1226.4749 $[M+H]^+$, found 1226.2474 $[M+H]^+$.

FT-IR (ATR): ν_{max} 3138, 2953, 2929, 1517, 1279, 1236 and 844 cm^{-1}.

^{1}H-NMR (500 MHz, DMSO-d_6) Compound 24 (PPA 3-mer).

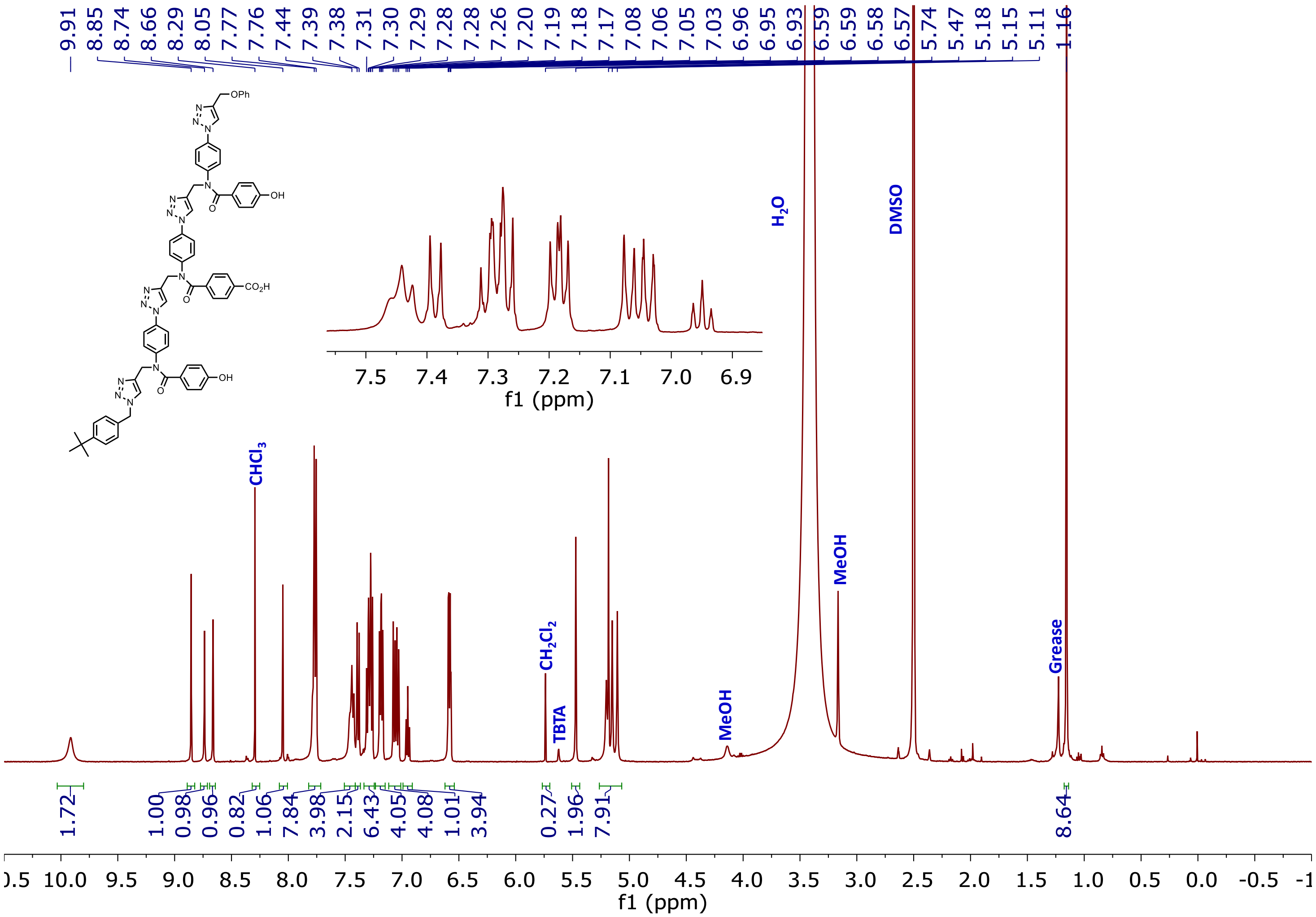

^{13}C-NMR (125MHz, DMSO-d_6) Compound 24 (PPA 3-mer).

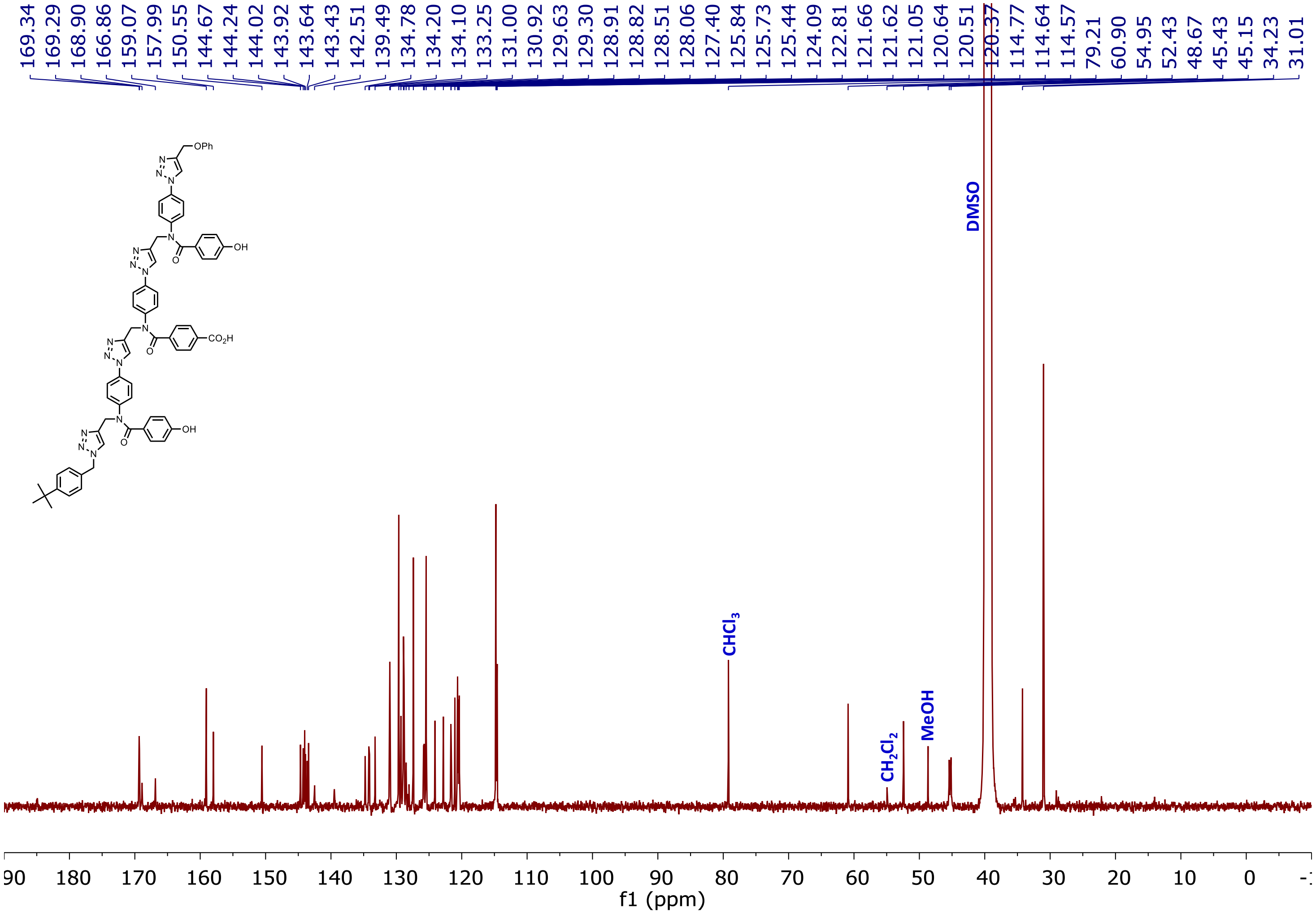

COSY (DMSO-d_6) Compound 24 (PPA 3-mer).

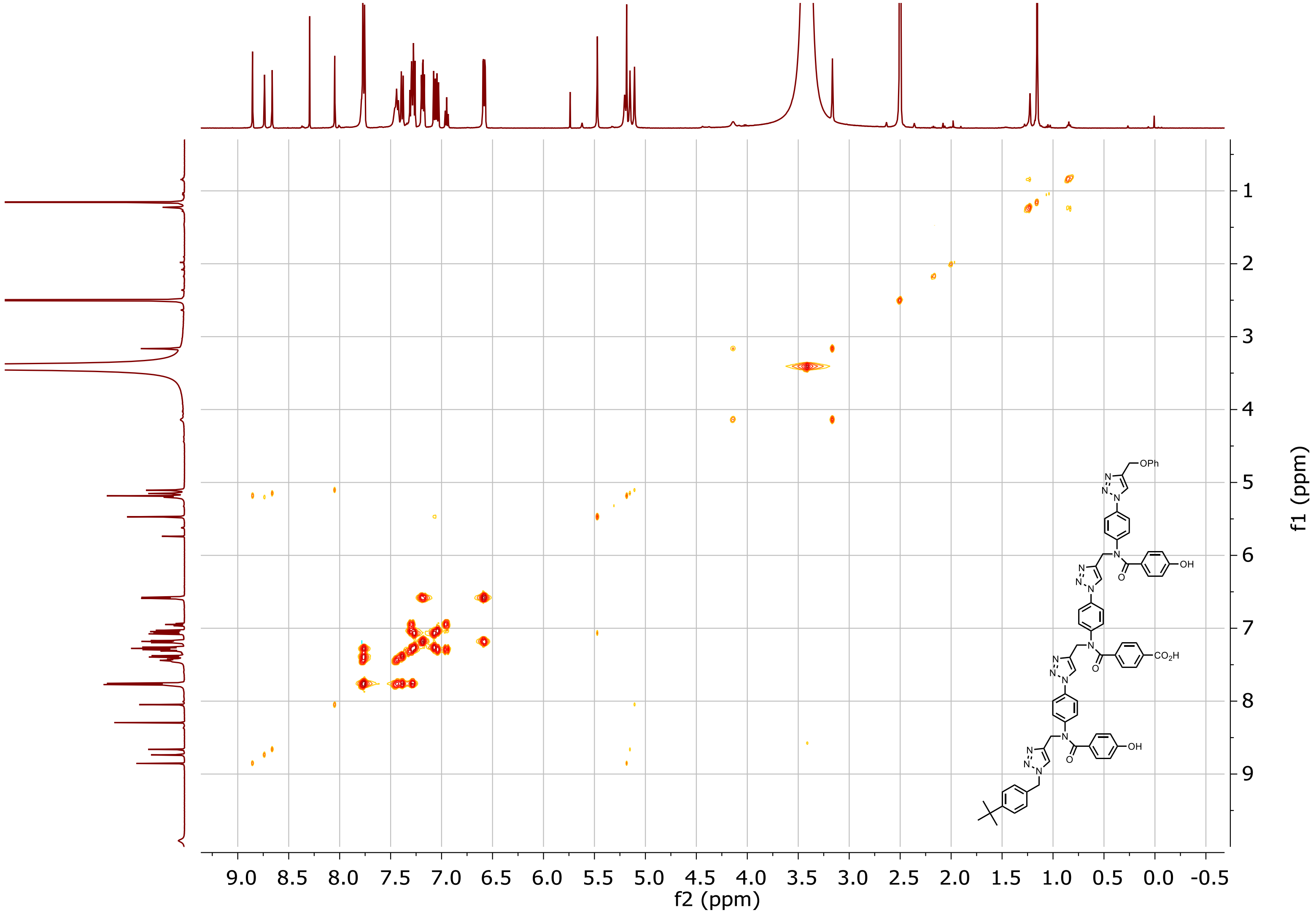

HSQC (DMSO-d_6) Compound 24 (PPA 3-mer).

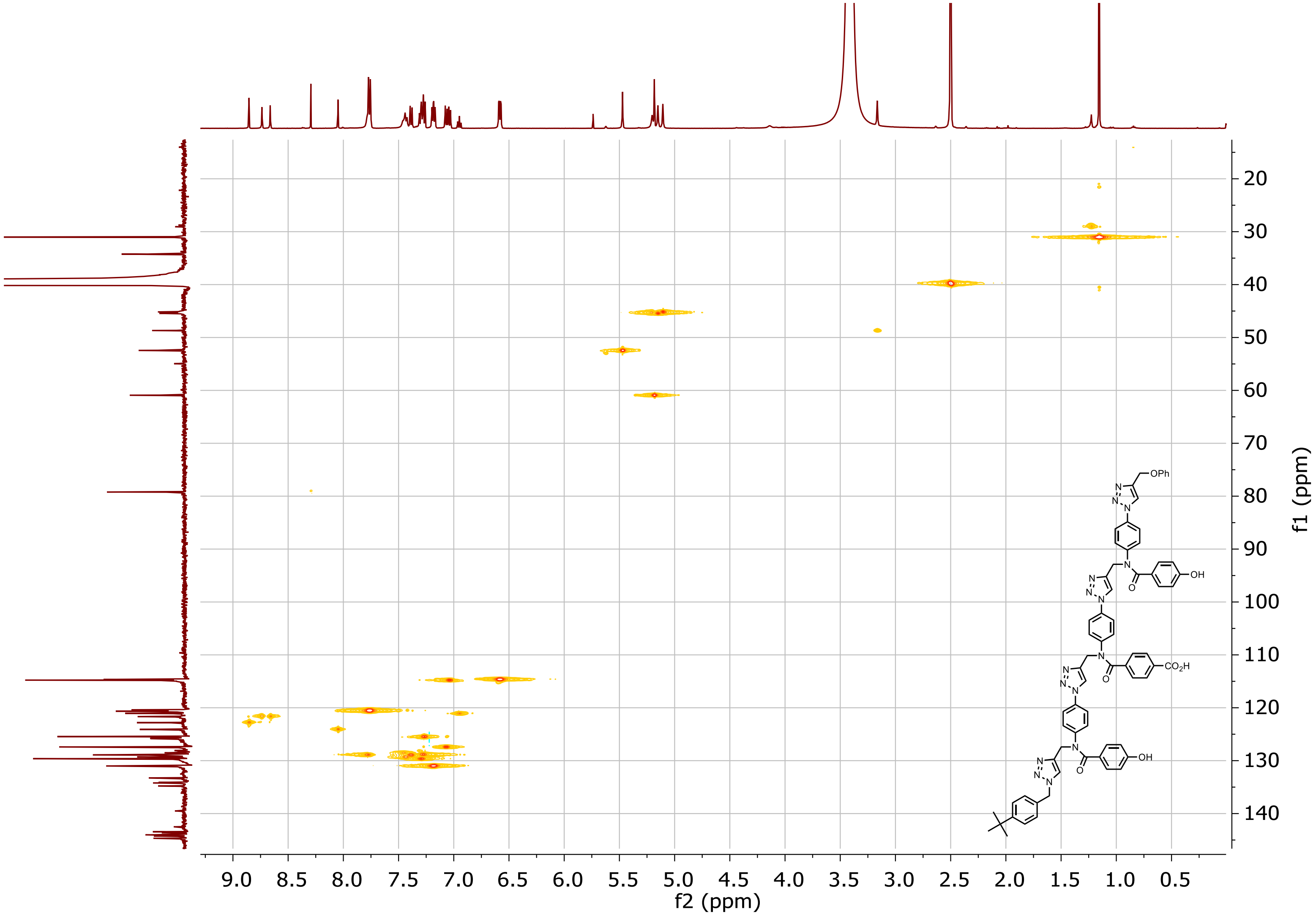

HMBC (DMSO-d_6) Compound 24 (PPA 3-mer).

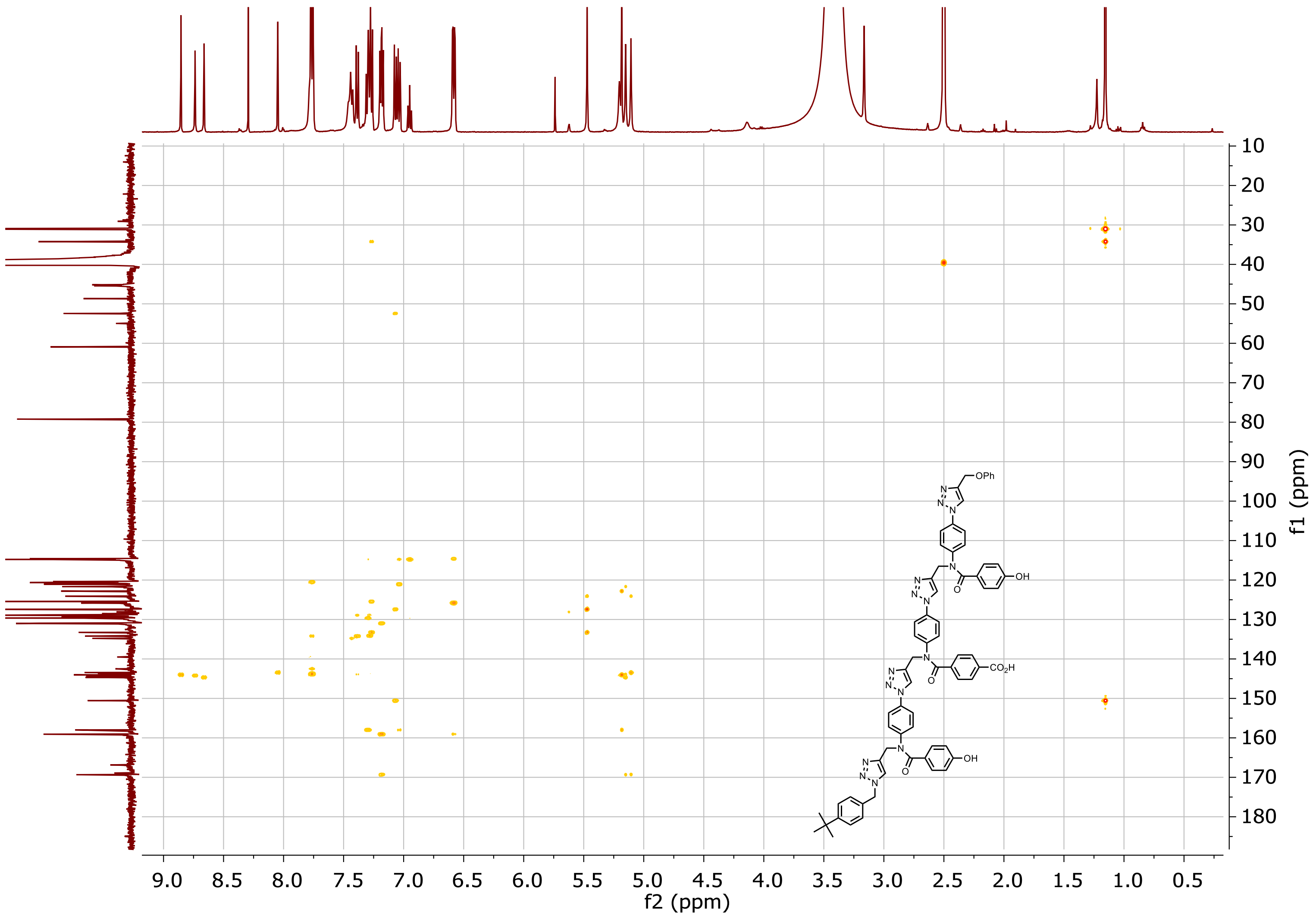

LCMS Compound 24 (PPA 3-mer).

Conditions: C18 column at 40 °C (254 nm) using water + 0.1% formic acid (A) and CH_3CN + 0.1% formic acid (B) with flow rate of 0.6 ml/min; Gradient of 0-2 minutes 5% -100%B + 1 minute 100% B.

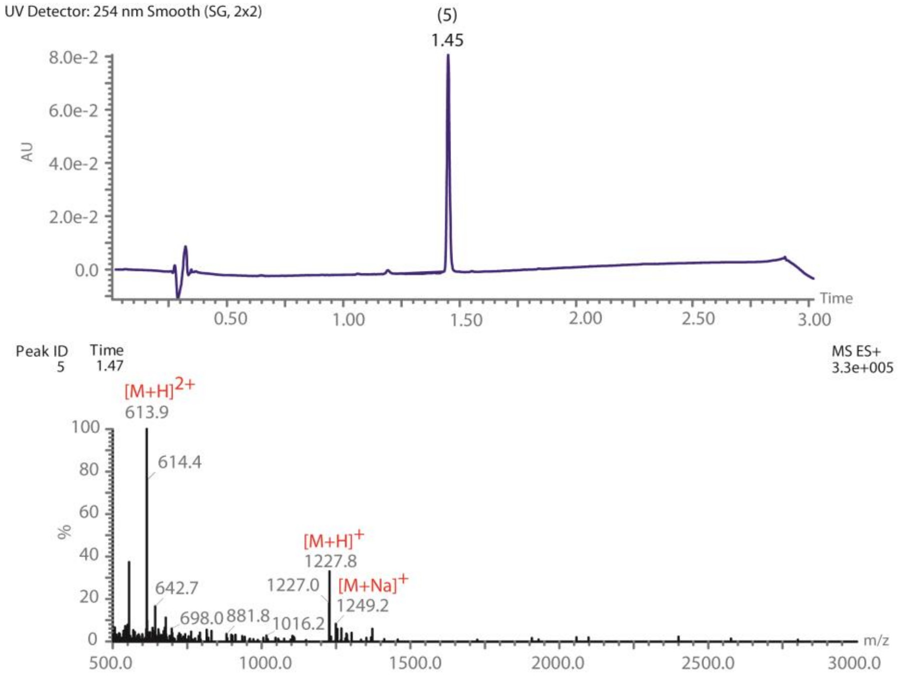

HRMS Compound 24 (PPA 3-mer).

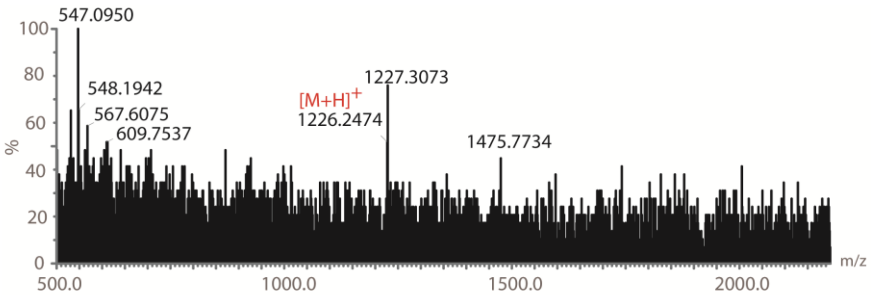

Template-directed information transfer

Mixed 3-mer template **13** was subjected to a replication cycle through selective formation and cleavage of phenol-carboxylic acid esters (Scheme S12). Following a protocol of phenol protection (P), ester coupling (C1), phenol deprotection (dP) and ester coupling (C2), the pre-ZIP complex **28** was isolated in 63% overall yield over 4 steps, with only one chromatography purification. The CuAAC ZIP reaction and *in situ* capping of **28** with *p-tert*-butylbenzylazide afforded duplex **29**. Hydrolysis of **19** using LiOH gave access to template reaction product **30**, and allowed the recovery of template **13**. Compound 30 was capped with phenyl propargyl ether to yield complementary copy **31**.

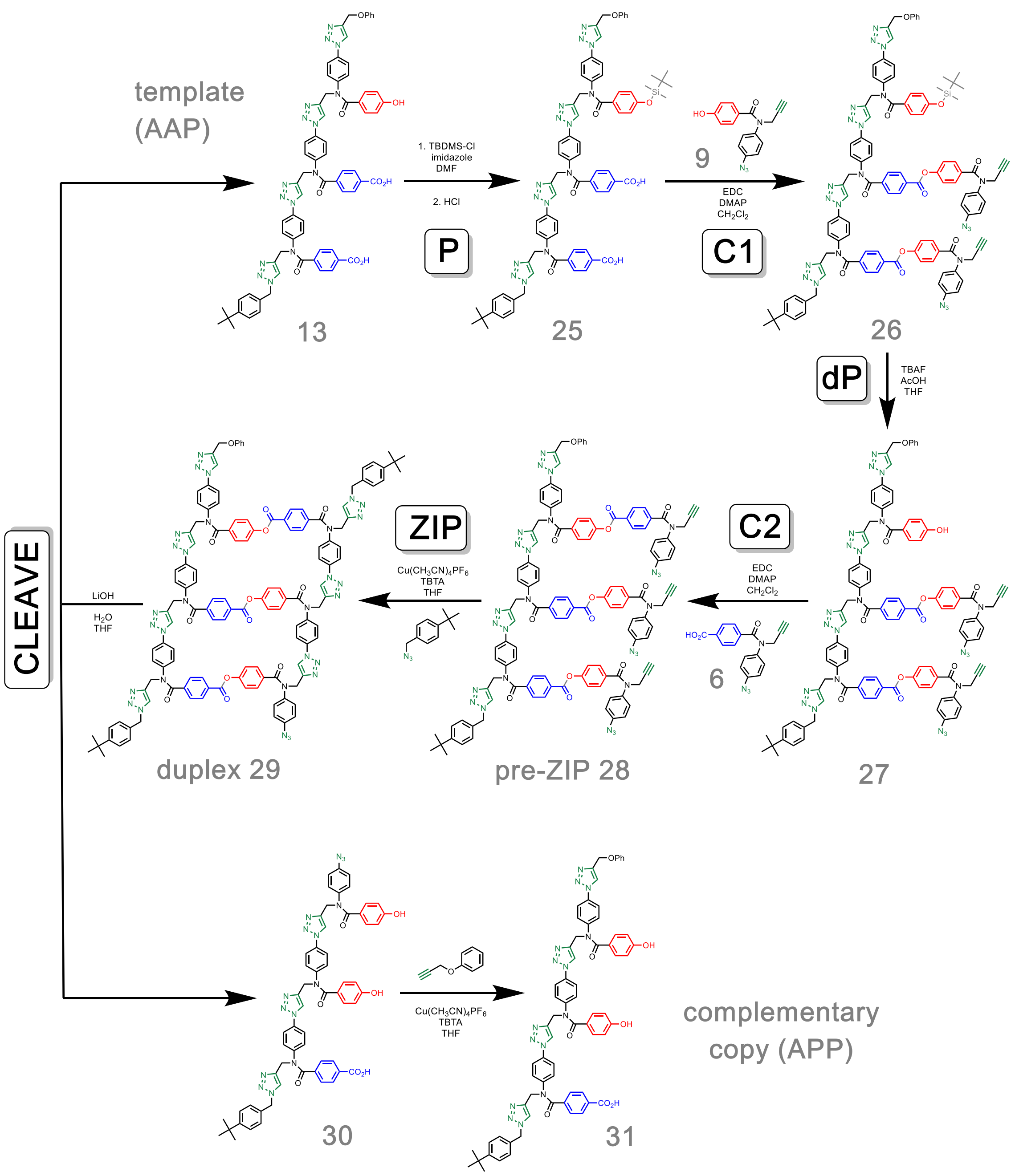

Scheme S12

Step 1: Phenol protection (P).

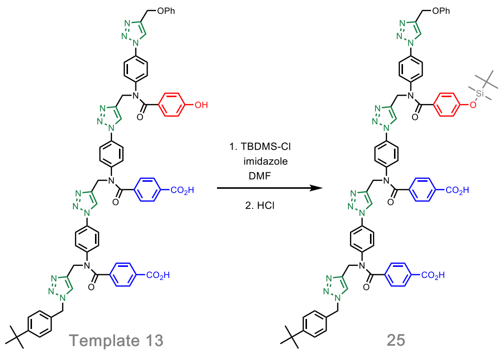

Scheme S13

A solution of template **13** (0.049 g, 0.04 mmol) in DMF (1 mL) was treated with imidazole (0.079 g, 1.16 mmol) and TBDMS-Cl (0.087 g, 0.58 mmol). After 5 h of stirring at room temperature, 0.1M HCl soln. was added until pH= 3-4 and the solution stirred at room temperature for 45 min. The solution was extracted with EtOAc (3x) and the combined organic layer was washed with 5% LiCl soln (3x), H_2O (1x) and brine (1x), dried with anhydrous $MgSO_4$, filtered, and the solvent evaporated. The crude (**25**) was used without further purification. Figure S7 shows the UPLC trace and mass spectrum of **25**.

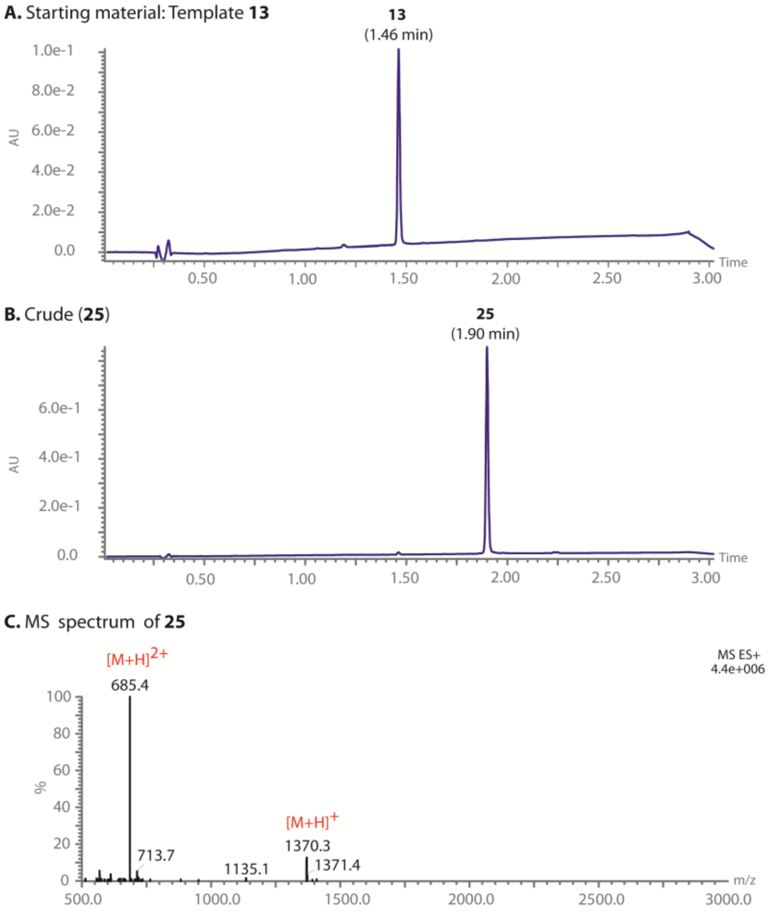

Figure S7. UPLC trace of the starting template **13** (**A**) and crude of step 1 (**B**). *Conditions:* C18 column at 40 °C (254 nm) using water + 0.1% formic acid (A) and CH_3CN + 0.1% formic acid (B) with flow rate of 0.6 ml/min; Gradient of 0-2 minutes 5% -100% B + 1 minute 100% B. **C.** MS spectrum of **25** (MW: 1368.6).

Step 2: Ester coupling (C1).

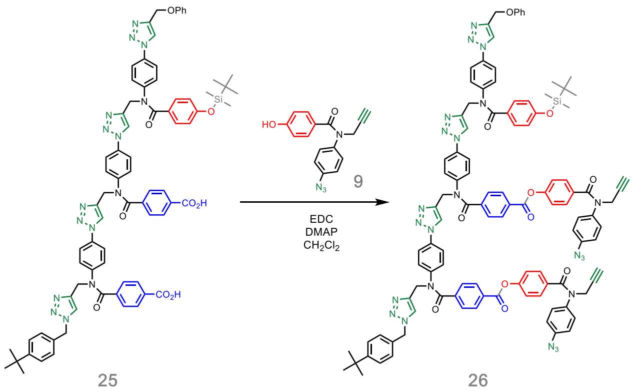

Scheme S14

Crude **25** (0.04 mmol), **9** (0.023 g, 0.08 mmol), EDC (0.018 g, 0.09 mmol) and DMAP (≈ 1 mg, $7{\cdot}10^{-3}$ mmol) were added to a flask. Under N_2 atmosphere, dry CH_2Cl_2 (1 mL) was added and reaction was stirred at room temperature for 2 h. Once finished, the reaction was diluted with EtOAc and washed with 0.1M HCl soln. (2x), H_2O (1x) and brine (1x). The solution was dried with anhydrous $MgSO_4$, filtered and the solvent evaporated. The obtained crude (**26**) was used in the next step without further purification. Figure S8 shows the UPLC trace and mass spectrum of **26**.

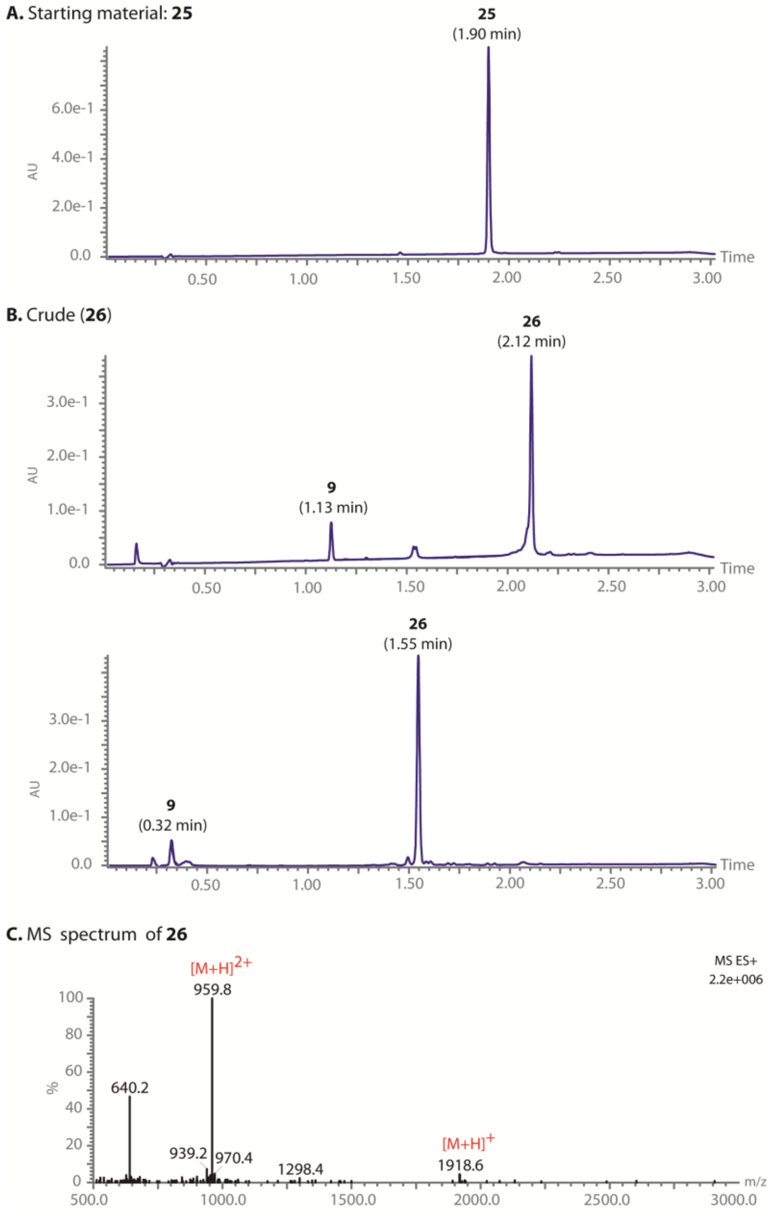

Figure S8. A. UPLC trace of the starting material **25**. *Conditions:* C18 column at 40 °C (254 nm) using water + 0.1% formic acid (A) and CH_3CN + 0.1% formic acid (B) with flow rate of 0.6 ml/min; Gradient of 0-2 minutes 5% -100% B + 1 minute 100% B. **B.** UPLC trace of crude of step 2. *Conditions:* C18 column at 40 °C (254 nm) using water + 0.1% formic acid (A) and CH_3CN + 0.1% formic acid (B) with flow rate of 0.6 ml/min; Gradient of 0-2 minutes 5% -100% B + 1 minute 100% B (top chromatogram), Gradient of 0-2 minutes 65% -100% B + 1 minute 100% B (bottom chromatogram). **C.** MS spectrum of **26** (MW: 1917.2).

Step 3: TBAF-mediated phenol deprotection (dP).

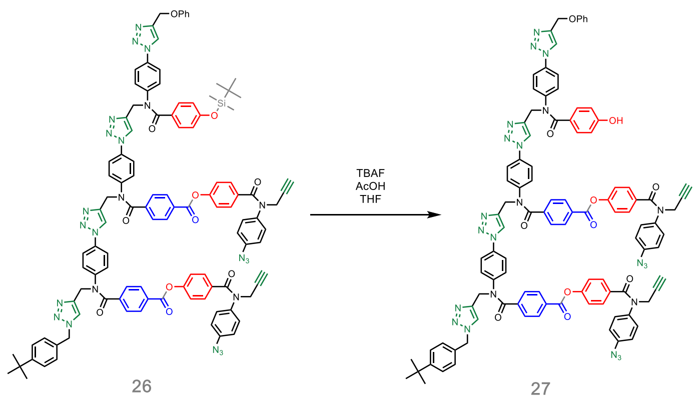

Scheme S15

Crude **26** (0.04 mmol) was dissolved in dry THF (1 mL) under N_2 atmosphere. Acetic acid (2 µL, 0.04 mmol) was added and, after cooling down the solution to 0 °C, TBAF (1M in THF, 0.04 mL, 0.04 mmol) was added dropwise. After 10 minutes of stirring at 0 °C, the reaction was quenched with 0.1M HCl soln. and diluted with EtOAc. The organic layer was separated and washed with 0.1M HCl soln. (2x), H_2O (1x) and brine (1x). The solution was dried with anhydrous $MgSO_4$, filtered and the solvents evaporated. The obtained crude (**27**) was used in the next step without further purification. Figure S9 shows the UPLC trace and mass spectrum of **27**.

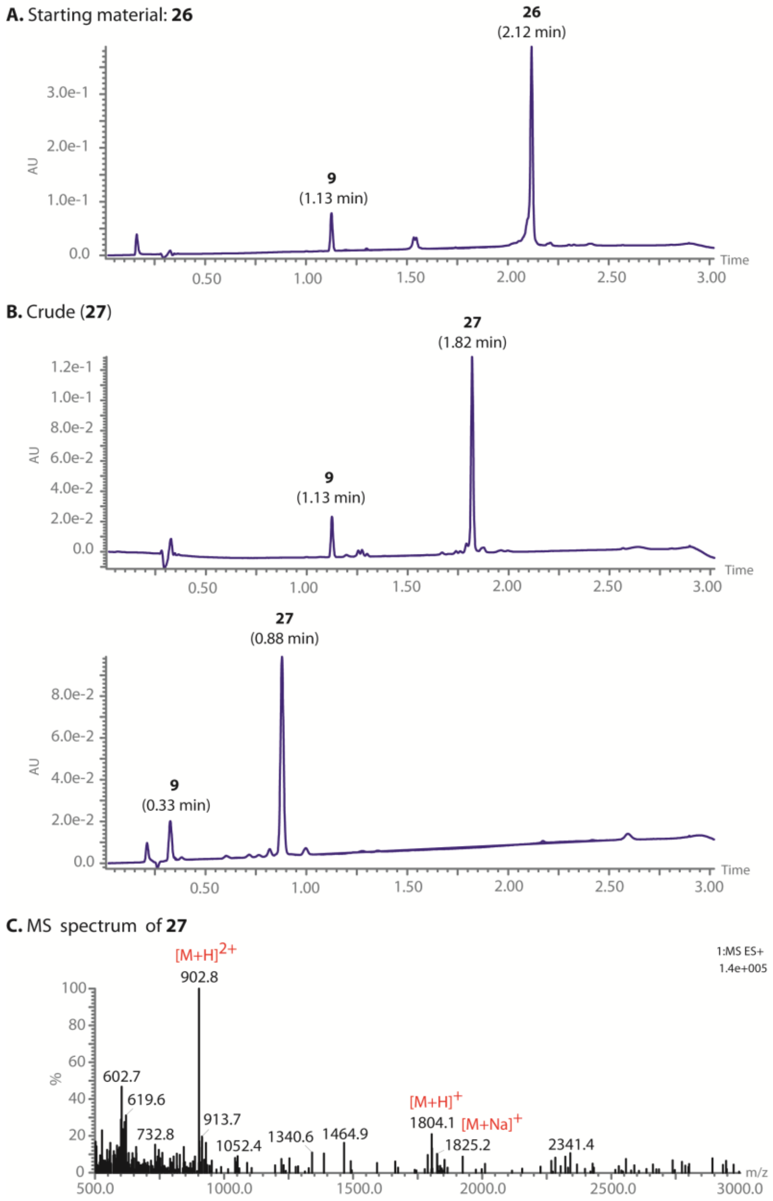

Figure S9. A. UPLC trace of the starting material **26**. *Conditions:* C18 column at 40 °C (254 nm) using water + 0.1% formic acid (A) and CH_3CN + 0.1% formic acid (B) with flow rate of 0.6 ml/min; Gradient of 0-2 minutes 5% -100% B + 1 minute 100% B. **B.** UPLC trace of crude of step 3. *Conditions:* C18 column at 40 °C (254 nm) using water + 0.1% formic acid (A) and CH_3CN + 0.1% formic acid (B) with flow rate of 0.6 ml/min; Gradient of 0-2 minutes 5% -100% B + 1 minute 100% B (top chromatogram), Gradient of 0-2 minutes 65% -100% B + 1 minute 100% B (bottom chromatogram). **C.** MS spectrum of **27** (MW: 1802.9).

Step 4: Ester coupling (C2).

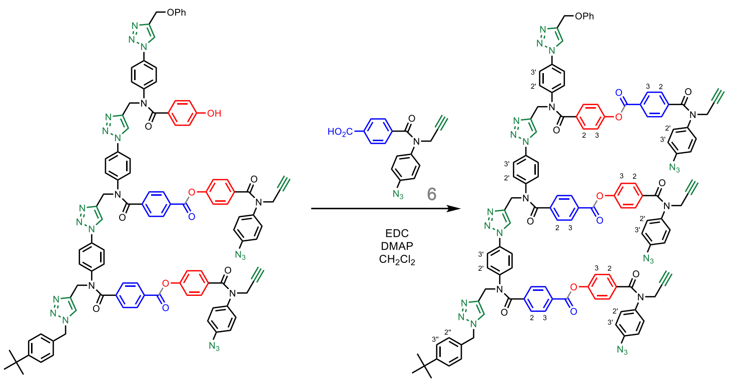

27

pre-ZIP 28

Scheme S16

Crude **27** (0.04 mmol), **6** (0.012 g, 0.04 mmol), EDC (0.009 g, 0.05 mmol) and DMAP (≈ 0.5 mg, $4{\cdot}10^{-3}$ mmol) were added to a flask. Under N_2 atmosphere, dry CH_2Cl_2 (1 mL) was added and reaction was stirred at room temperature for 2 h. Once finished, the reaction was diluted with EtOAc and washed with 0.1M HCl soln. (2x), H_2O (1x) and brine (1x). The solution was dried with anhydrous $MgSO_4$, filtered and the solvent evaporated. The obtained residue was purified by flash column chromatography on silica gel (gradient from 0% to 6% of MeOH in CH_2Cl_2) to afford pre-ZIP **28** (0.052 g, 63% over 4 steps: phenol protection-P, ester coupling-C1, phenol deprotection-dP and ester coupling-C2) as a yellow foam. Figure S10 shows the UPLC traces of the reaction crude and pure **28**, together with the mass spectrum of **28**.

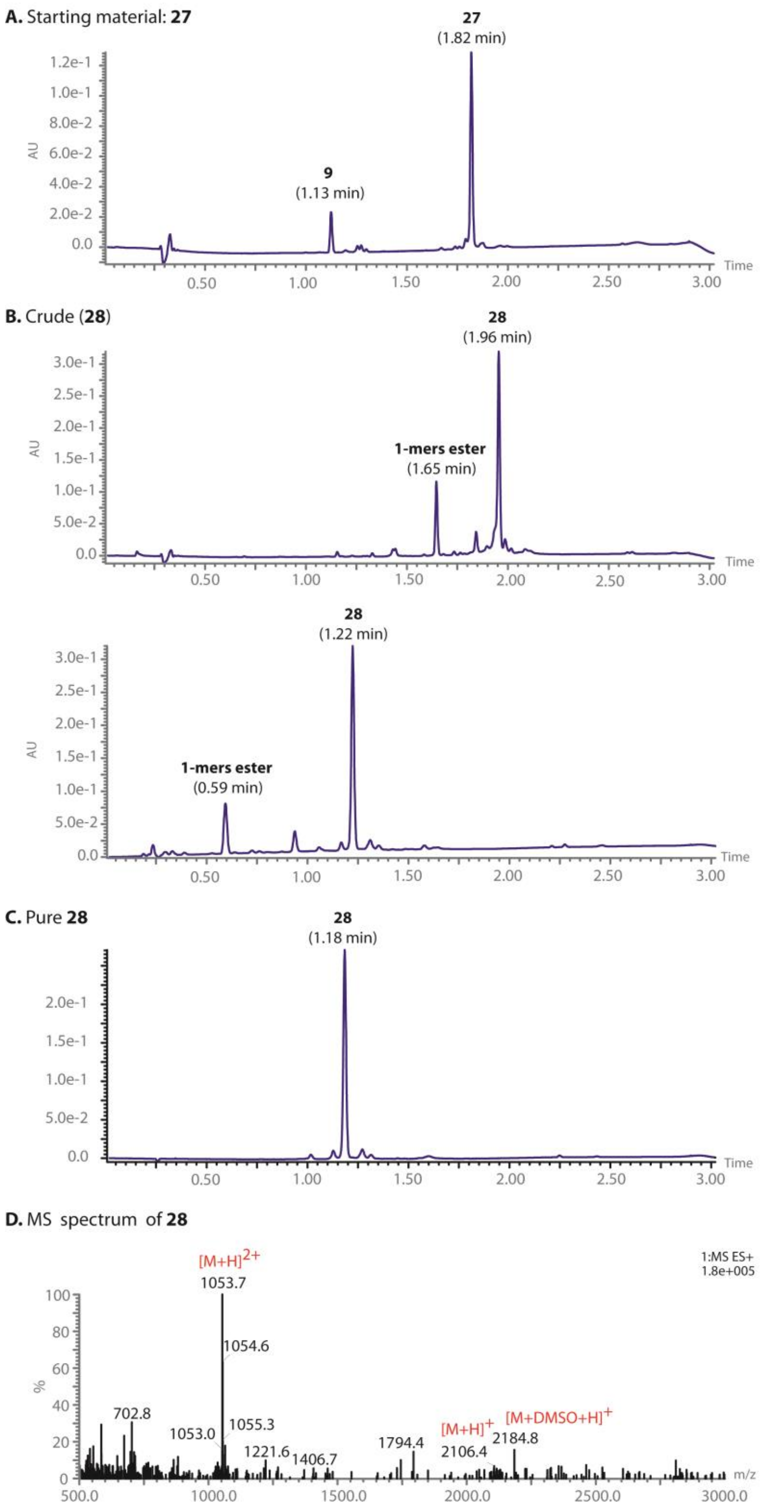

Figure S10. A. UPLC trace of the starting material **27**. *Conditions:* C18 column at 40 °C (254 nm) using water + 0.1% formic acid (A) and CH_3CN + 0.1% formic acid (B) with flow rate of 0.6 ml/min; Gradient of 0-2 minutes 5% -100% B + 1 minute 100% B. **B.** UPLC trace of crude of step 4. *Conditions:* C18 column at 40 °C (254 nm) using water + 0.1% formic acid (A) and CH_3CN + 0.1% formic acid (B) with flow rate of 0.6 ml/min; Gradient of 0-2 minutes 5% -100% B + 1 minute 100% B (top chromatogram), Gradient of 0-2 minutes 65% -100% B + 1 minute 100% B (bottom chromatogram). **C.** UPLC trace of pure **28**. *Conditions:* C18 column at 40 °C (254 nm) using water + 0.1% formic acid (A) and CH_3CN + 0.1% formic acid (B) with flow rate of 0.6 ml/min; Gradient of 0-2 minutes 65% -100% B + 1 minute 100% B. **D.** MS spectrum of **28** (MW: 2105.2).

Full characterization of pre-ZIP 28.

^{1}H NMR (400 MHz, CDCl$_3$): δ_H = 8.18 (s, 1H, CH$_{triaz}$, internal), 8.17 (s, 1H, CH$_{triaz}$, internal), 8.00 (s, 1H, CH$_{triaz}$, PhO cap), 7.67 (s, 1H, CH$_{triaz}$, ^{t}BuPh cap), 7.95 (m, 6H, J = 8.5 Hz, 3'-H, template), 7.42-7.29 (m, 22H, 2-H, phenol; 2-H, acid; 2'-H, linker; 3''-H, PhO; 3''-H, ^{t}BuPh), 7.21 (d, 2H, *J* = 8.5 Hz, 2''-H, ^{t}BuPh), 7.11 (d, 6H, *J* = 8.5 Hz, 2'-H, 1-mer), 7.03 (m, 6H, 3-H, phenol), 6.98 (m, 3H, 2''-H and 4''-H, PhO), 6.90 (d, 6H, 3'-H, 1-mer), 5.48 (s, 2H, N-CH$_2$, ^{t}BuPh cap), 5.28 (s, 2H, CH$_2$-O), 5.21 (s, 2H, N-CH$_2$, template), 5.20 (s, 2H, N-CH$_2$, template), 5.13 (s, 2H, N-CH$_2$, template), 4.66 (s, 2H, N-CH$_2$, alkyne, 1-mer), 4.63 (m, 4H, N-CH$_2$, alkyne, 1-mer), 2.28 (t, 1H, *J* = 2.5 Hz, CH, alkyne, 1-mer), 2.25 (t, 2H, *J* = 2.5 Hz, CH, alkyne, 1-mer), 1.31 (s, 9H, ^{t}Bu).

^{13}C NMR (100.6 MHz, CDCl$_3$): δ_C = 169.3, 169.3, 169.2, 169.2, 169.1 and 169.1 (CO, amide), 163.8, 163.7 and 163.7 (CO, ester), 158.2 (1''-C, PhO), 152.3, 152.1 and 152.0 (4-C, phenol), 145.5 (C$_{triaz}$, PhO), 144.7 (C$_{triaz}$), 144.3 and 144.3 (1'-C), 143.7 (C$_{triaz}$, ^{t}BuPh cap), 143.7 and 143.7 (4-C), 140.3, 140.3, 140.1, 139.7, 139.3 and 138.8 (1-C, acid; 1'-C, 1-mer; 4'-C , 1-mer), 135.6, 135.4, 135.3, 132.7, 132.7, 132.5, 131.5, 130.7, 130.6, 130.6, 130.5, 130.3, 130.0, 130.0, 129.9, 129.8, 129.4, 129.3, 129.3, 129.1, 129.0, 128.9, 128.8, 128.7, 128.7, 128.7, 128.1 and 126.2 (C$_{arom}$), 123.9 (CH$_{triaz}$, ^{t}Bu cap), 122.2 and 122.1 (CH$_{triaz}$, internal), 121.5, 121.5, 121.5, 121.4, 121.3, 121.2, 121.2 (3-C, phenol; 2'-C and 3'-C linker), 120.9 (CH$_{triaz}$, PhO), 120.0, 120.0 and 120.0 (3'-C, 1-mer), 114.9 (2''-C, PhO), 78.8 and 78.5 (C, alkyne, 1-mer), 73.0 and 72.8 (CH, alkyne, 1-mer), 62.0 (CH$_2$-O), 54.1 (CH$_2$, ^{t}BuPh cap), 46.6 and 46.4 (CH$_2$, internal), 40.1 (CH$_2$-alkyne, 1-mer), 34.8 (C, ^{t}Bu), 31.4 (CH$_3$, ^{t}Bu).

HRMS (ES+): calcd for $C_{119}H_{90}N_{27}O_{17}$ 1053.3662 $[M+H]^{2+}$, found 1053.2579 $[M+H]^{2+}$.

FT-IR (ATR): ν_{max} 2960, 2925, 2124, 2094, 1740, 1646, 1518, 1505, 1294, 1244, 1203, 1166, 1070 and 755 cm^{-1}.

^{1}H-NMR (400 MHz, $CDCl_3$) Pre-ZIP 28.

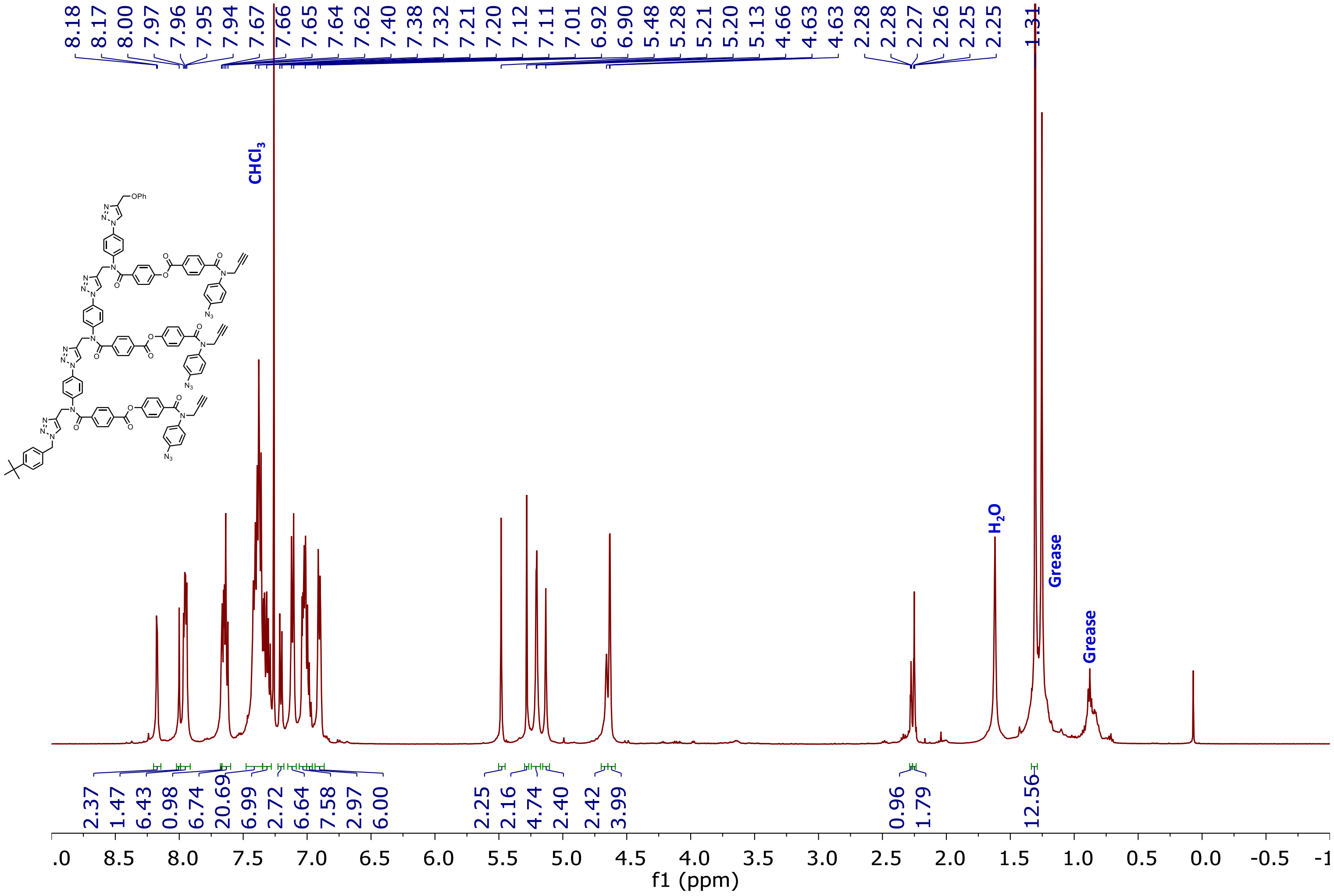

^{13}C-NMR (100.6 MHz, $CDCl_3$) Pre-ZIP 28.

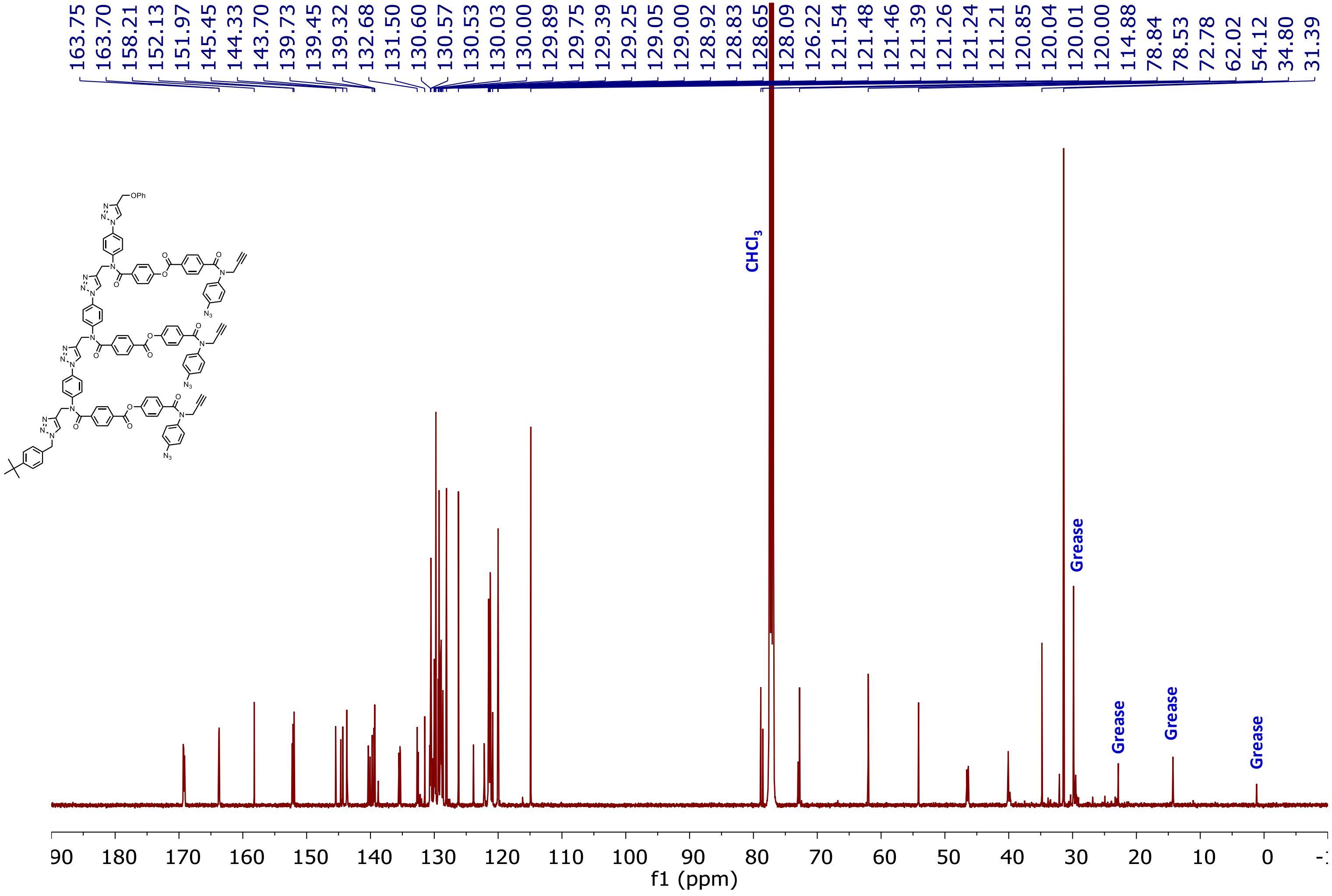

COSY ($CDCl_3$) Pre-ZIP 28.

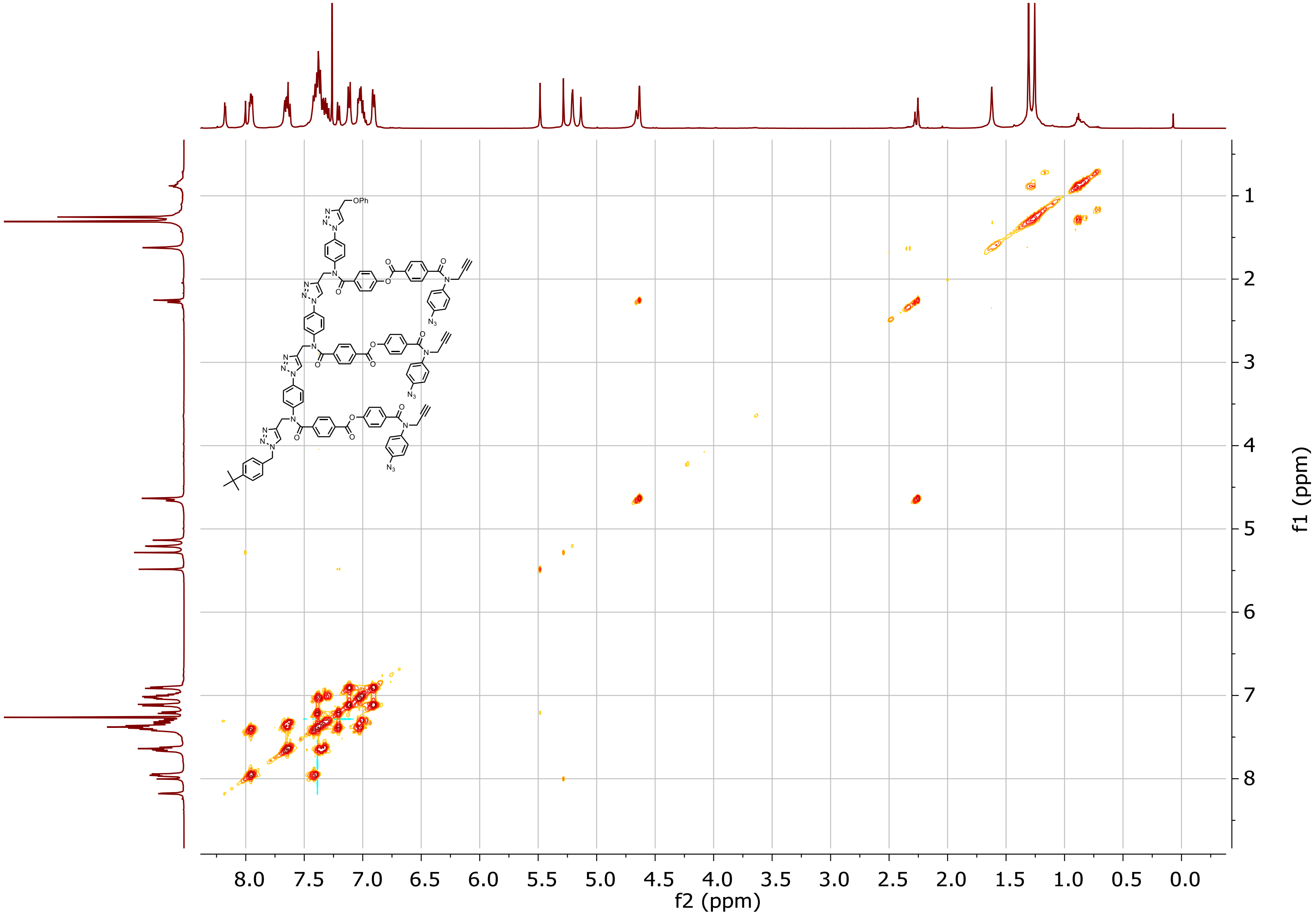

HSQC ($CDCl_3$) Pre-ZIP 28.

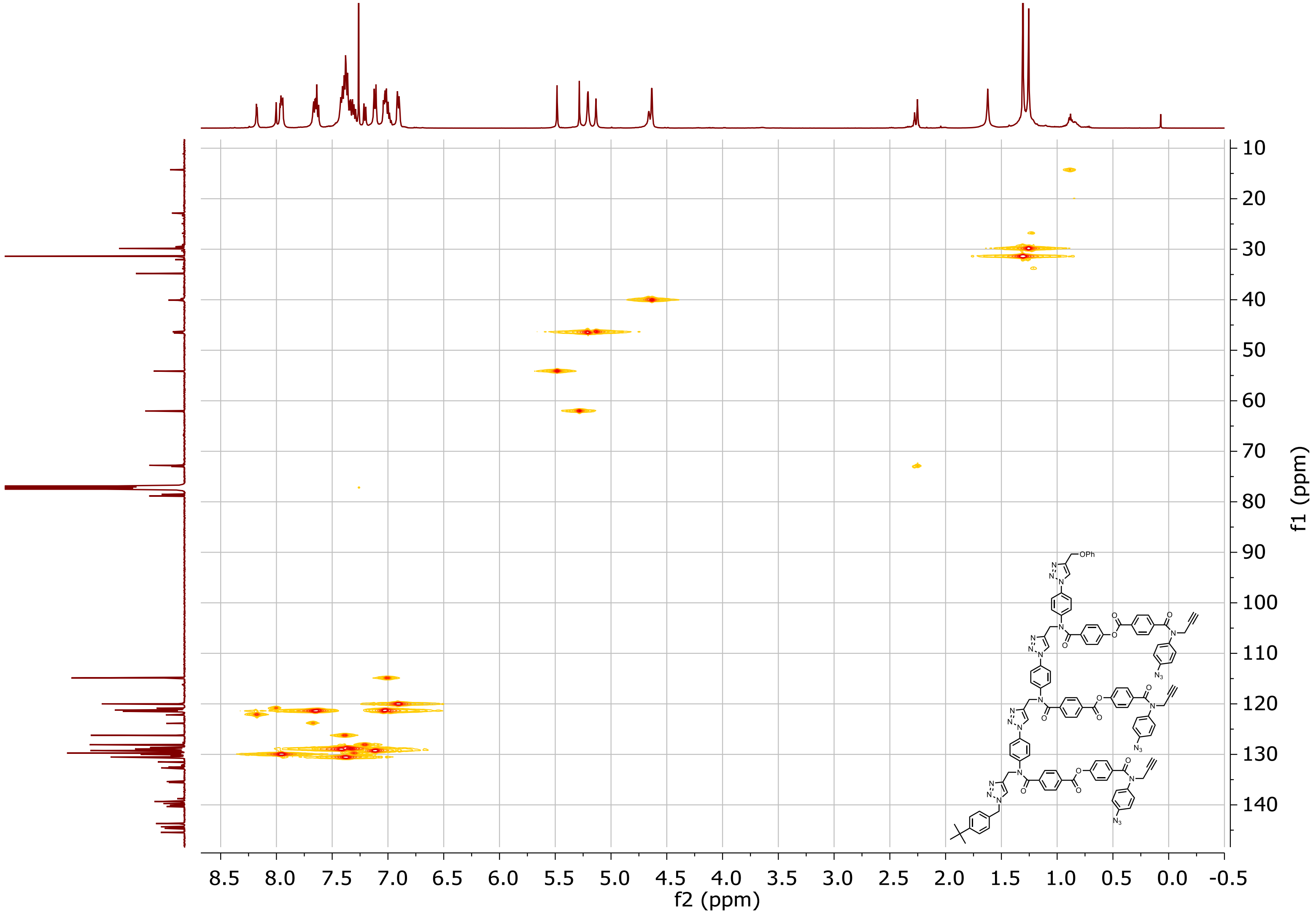

HMBC ($CDCl_3$) Pre-ZIP 28.

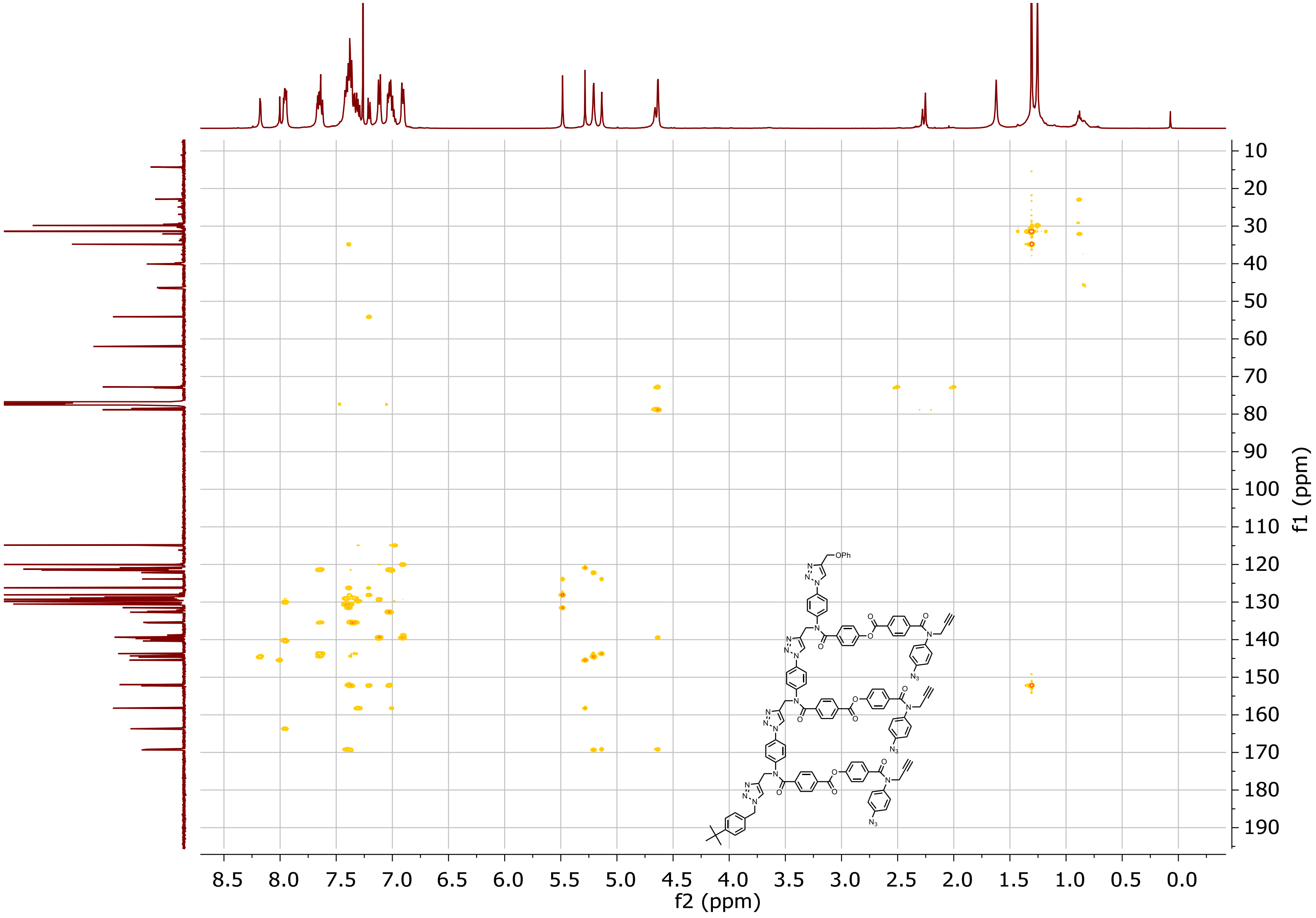

HRMS PreZIP 28.

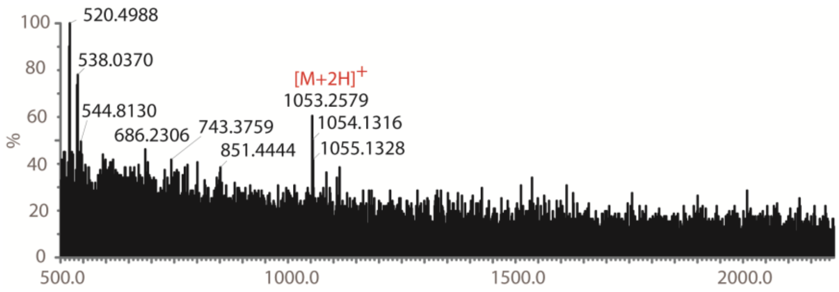

Step 5: ZIP reaction

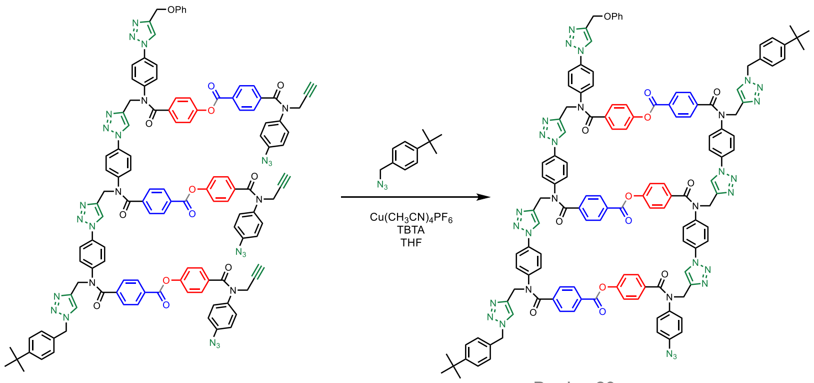

Scheme S17

A solution of 1-(azidomethyl)-4-*tert*-butylbenzene (0.200 g, 1.1 mmol) in dry and degassed THF (5 mL) was added to a solution of Pre-ZIP **28** (0.015 g, $7.1{\cdot}10^{-3}$ mmol) in dry and degassed THF (450 mL) under N_2 atmosphere. A solution of $Cu(CH_3CN)_4PF_6$ (0.079 g, 0.2 mmol) and TBTA (0.113 g, 0.2 mmol) in dry and degassed THF (15 mL) was added to the previous solution and the reaction stirred overnight at room temperature. Then, the solvent was evaporated and the crude dissolved in EtOAc and washed with 0.02M EDTA soln. (2x), 0.1M HCl soln. (1x), H_2O (1x) and brine. The organic layer was dried over $MgSO_4$ and concentrate under vacuum. The obtained crude (**29**) was used in the next step without further purification. Figure S11 shows the UPLC trace and mass spectrum of **29**.

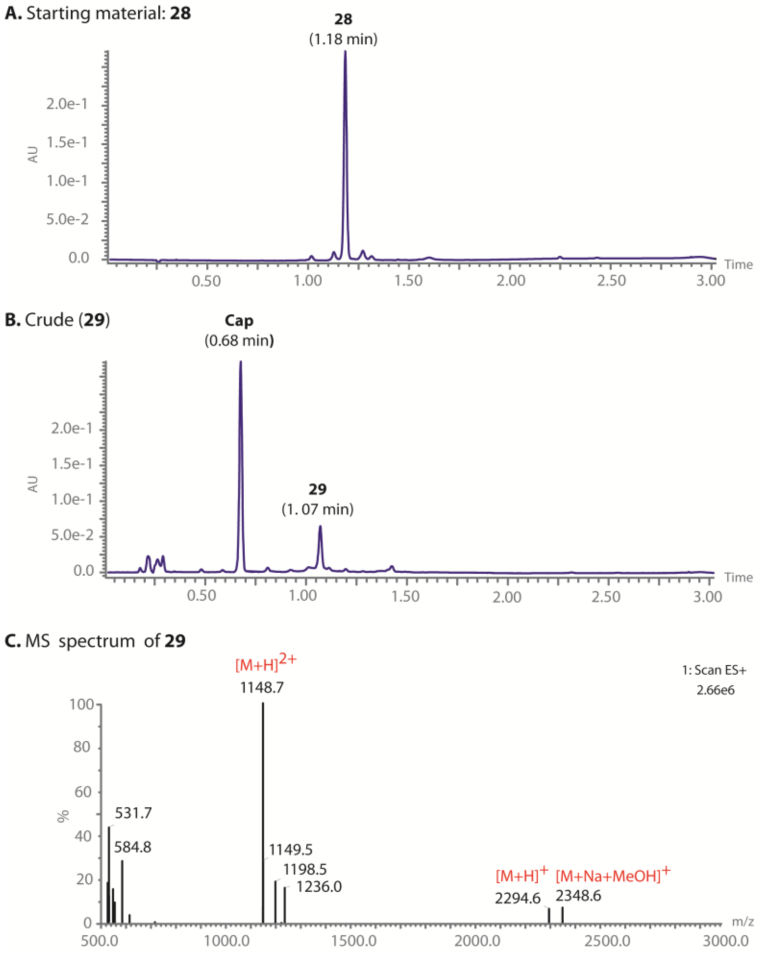

Figure S11. UPLC traces of the starting material **28** (**A**) and crude of step 5 (**B**). *Conditions:* C18 column at 40 °C (254 nm) using water + 0.1% formic acid (A) and CH_3CN + 0.1% formic acid (B) with flow rate of 0.6 ml/min; Gradient of 0-2 minutes 65% -100% B + 1 minute 100% B. **C.** MS spectrum of **29** (MW: 2293.8).

Step 6: Hydrolysis

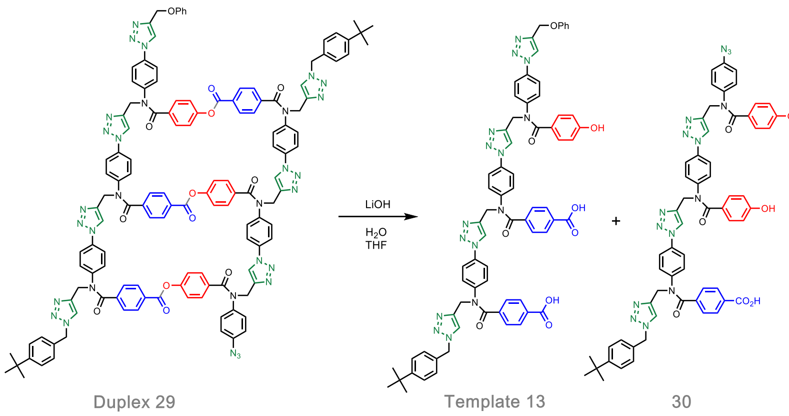

Scheme S18

Crude **29** was dissolved in THF:H_2O 3:1 (1 mL) and 1M LiOH soln. (0.10 mL, 0.10 mmol) was added. The reaction was stirred at room temperature for 10 min. Then, the crude was diluted with H_2O and acidified with 0.1M HCl soln. to pH 2-3. The aqueous phase was extracted with EtOAc (3x) and the combined organic phase was washed with brine, dried over $MgSO_4$ and evaporated to dryness. The residue was purified by flash column chromatography on silica gel (gradient from 0% to 20% of MeOH in CH_2Cl_2) to afford template **13** (0.005 g, 56% over two steps: ZIP and hydrolysis) as a white solid and copy **30** (0.006 g, 77% over two steps: ZIP and hydrolysis) as a white solid. Figure S12 shows the UPLC traces of the reaction crude and pure recovered template **13** and copy **30**, together with their mass spectra. Figure S13 shows the 500 MHz ^1H NMR spectra of recovered template **13** and the starting template used for replication. As highlighted in Figure S14, the 500 MHz ^1H NMR spectrum of **30** showed the existence of three different 3-mers: complementary APP 3-mer (71%, green), PAP 3-mer (16%, blue) and PPA 3-mer (13%, purple).

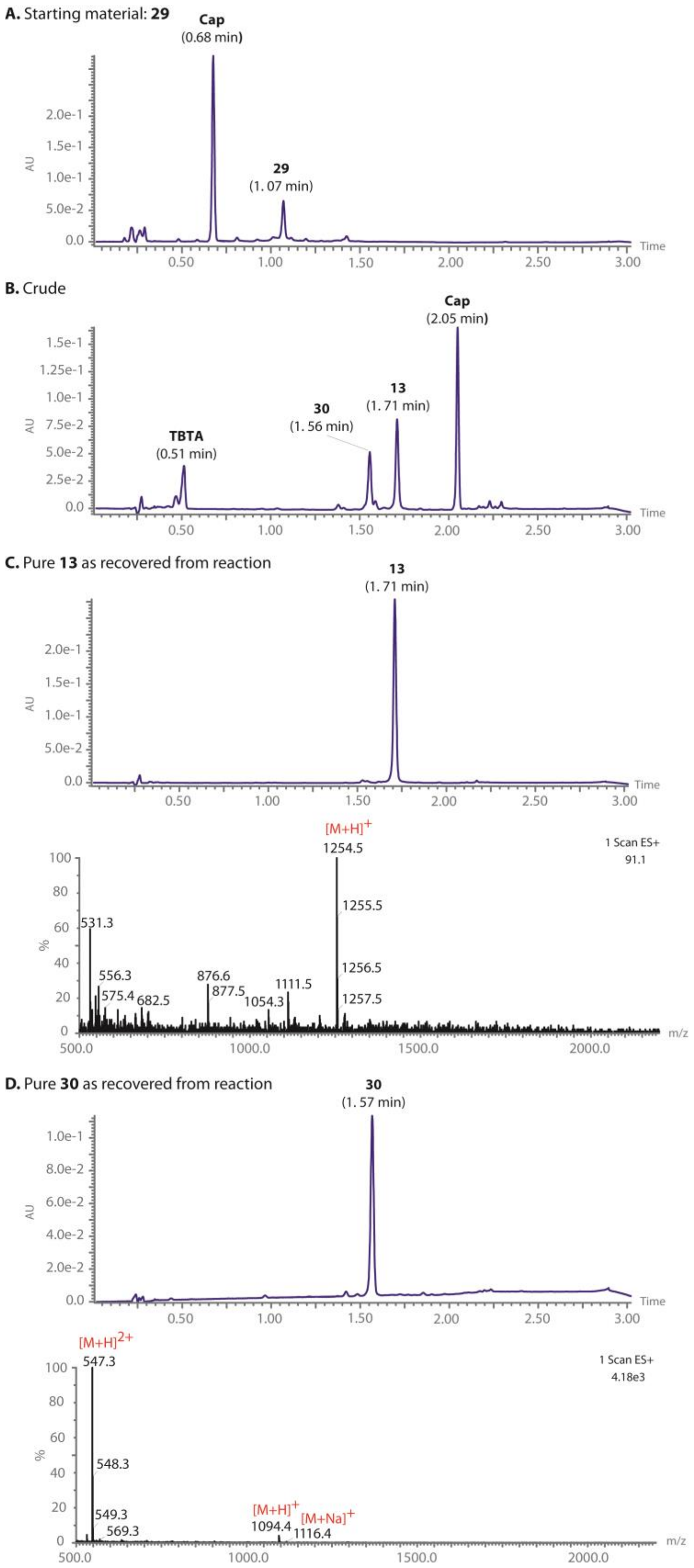

Figure S12. A. UPLC trace of the starting material (**29**). **B.** UPLC trace of crude of step 6. **C.** ULPC trace and MS spectrum of template **13** (MW: 1253.5) as recovered after purification. **D.** UPLC trace and MS spectrum of copy **30** (MW: 1093.4) as recovered after purification. *UPLC Conditions:* C18 column at 40 ^{o}C (254 nm) using water + 0.1% formic acid (A) and CH_3CN + 0.1% formic acid (B) with flow rate of 0.6 ml/min; Gradient of 0-2 minutes 35% -65% B + 1 minute 100% B.

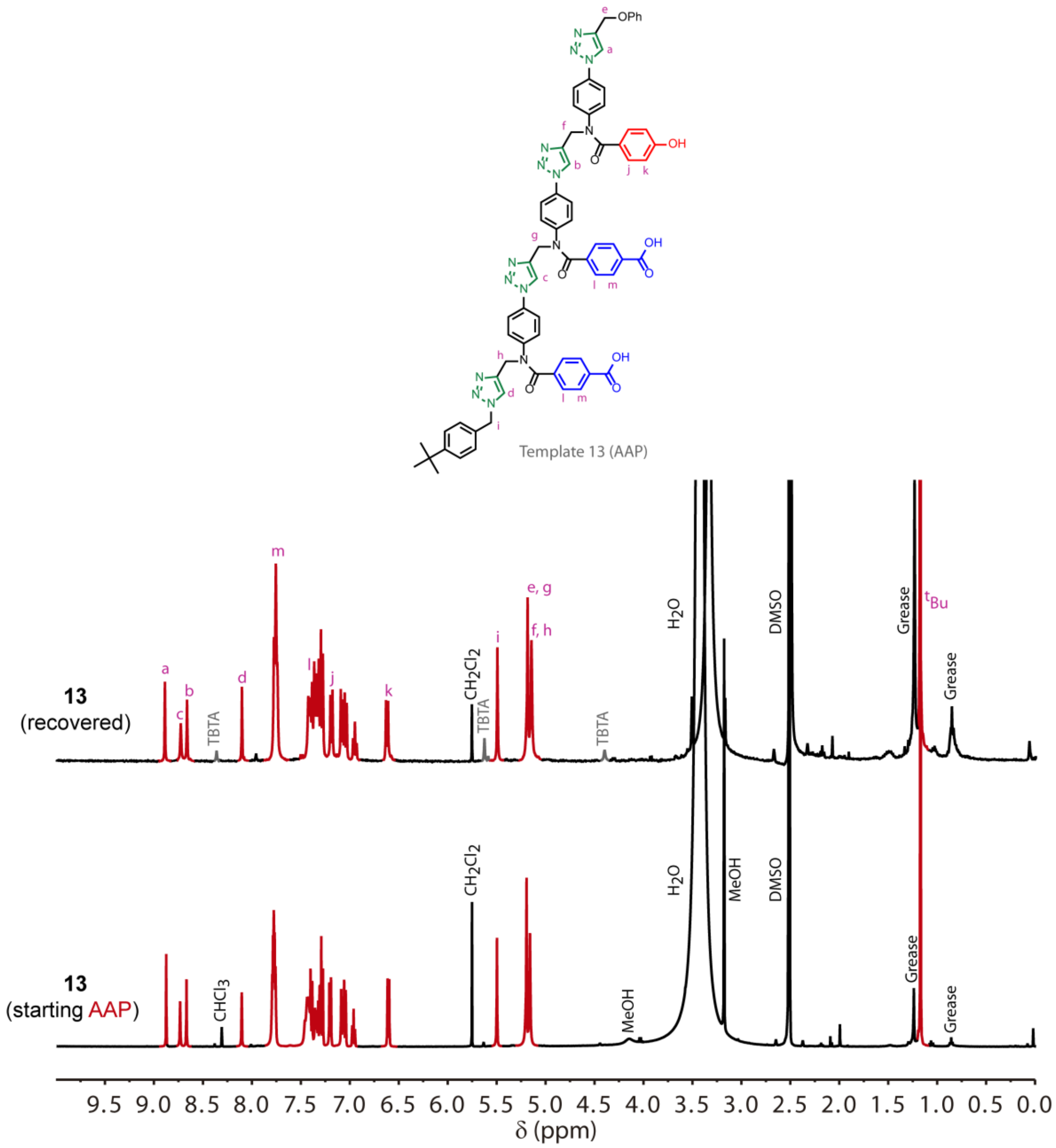

Figure S13. 500 MHz ^{1}H NMR spectra in DMSO-d_6 at 298 K of recovered template **13** (top) and the starting template used for replication (bottom). The signals corresponding to **13** are highlighted in red, and the main proton signals are assigned with letters.

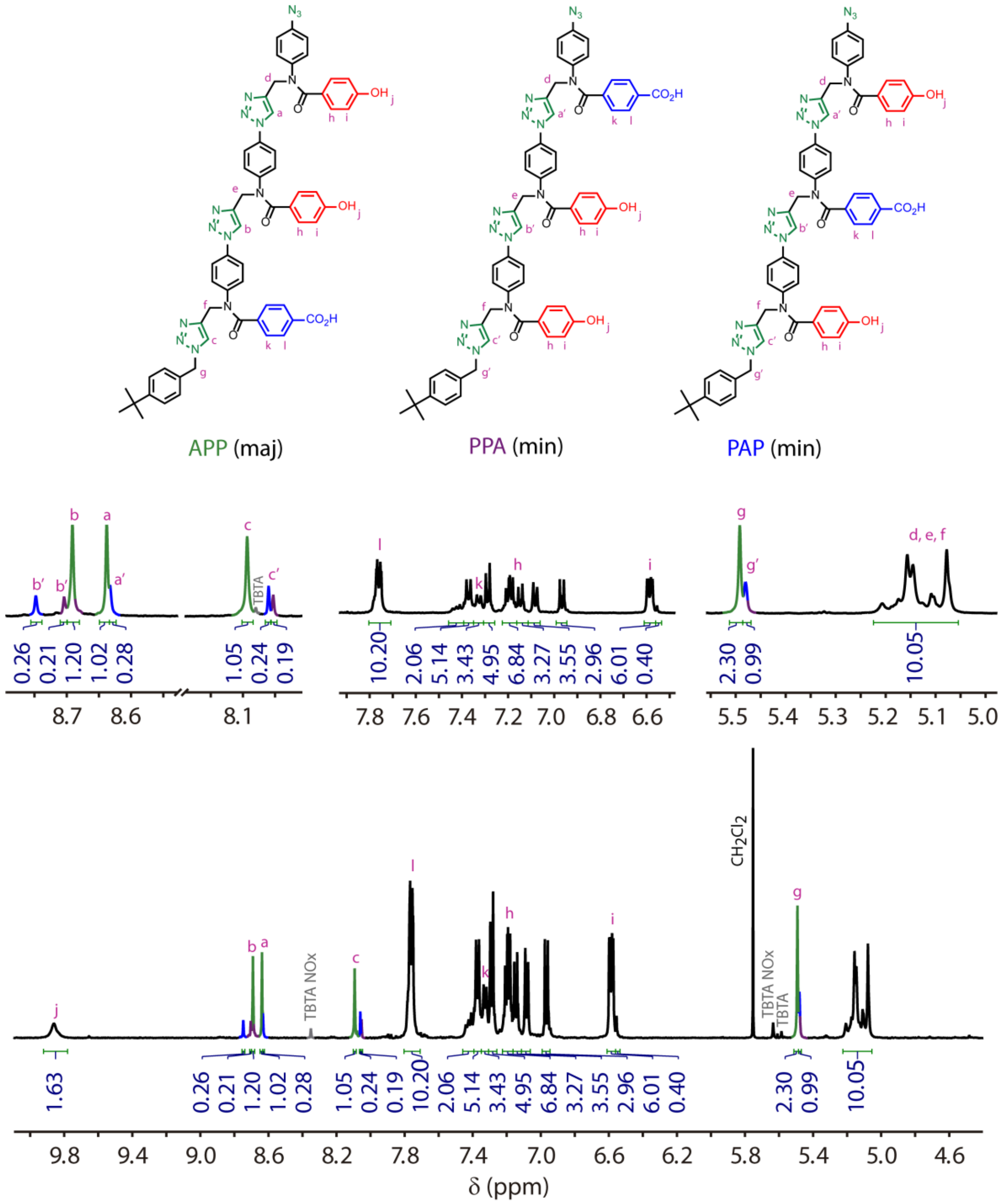

Figure S14. 500 MHz ^{1}H NMR spectra of copy **30** in DMSO-d_6 at 298 K, showing APP (major in green), PPA (minor, purple) and PAP (minor, blue) sequences. Expansions of key regions are shown on top and the main proton signals are assigned with letters.

Full characterization of 30 (APP, major).

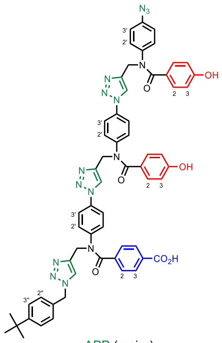

APP (major)

^{1}H NMR (500 MHz, DMSO-d_6): δ_H = 9.87 (s, 2H, OH), 8.69 (s, 1H, CH_{triaz}), 8.64 (s, 1H, CH_{triaz}), 8.09 (s, 1H, CH_{triaz}), 7.77 (m, 6H, 3-H, acid; 3'-H), 7.37 (m, 4H, 2'-H), 7.29 (m, 4H, 2-H, acid; 3''-H), 7.19 (m, 4H, 2-H, phenol; 2'-H, azidophenyl), 7.15 (d, 2H, *J* = 8.5 Hz, 2-H, phenol), 7.08 (d, 2H, *J* = 8.5 Hz, 2''-H), 6.97 (d, 2H, *J* = 9.0 Hz, 3'-H, azidophenyl), 6.59 (d, 2H, *J* = 8.5 Hz, 3-H, phenol), 6.58 (d, 2H, *J* = 8.5 Hz, 3-H, phenol), 5.49 (s, 2H, N-CH_2, ^{t}Bu cap), 5.16 (s, 2H, N-CH_2, acid), 5.15 (s, 2H, N-CH_2, internal phenol), 5.08 (s, 4H, N-CH_2, terminal phenol), 1.18 (s, 9H, ^{t}Bu).

^{13}C NMR (125 MHz, DMSO-d_6): δ_C = 169.3 and 169.2 (CO, amide phenol), 168.9 (CO, amide acid), 167.1 (CO, acid), 159.0 and 159.0 (4-C, phenol), 150.5 (4''-C), 144.8 and 144.6 (C_{triaz}), 143.6 and 143.5 (1'-C), 143.0 (C_{triaz}), 140.6 (1'-C, azidophenyl), 139.1 (1-C, acid), 137.2 (4'-C, azidophenyl), 134.5 and 134.2 (4'-C), 133.2 (4-C, acid), 130.9 and 130.7 (2-C, phenol), 129.2, 129.1, 129.0, 128.8, 128.7, 128.0 and 127.3 (C_{arom}), 125.7 (1-C, phenol), 125.4 (3''-C), 124.1 (CH_{triaz}, ^{t}Bu cap) 121.5 and 121.4 (CH_{triaz}), 120.4 and 120.2 and 120.3 (3'-C), 119.6 (3'-C, azidophenyl), 114.5 and 114.5 (3-C, phenol), 52.4 (N-CH_2, ^{t}Bu cap), 45.6, 45.4 and 44.9 (N-CH_2), 34.2 (C, ^{t}Bu), 31.0 (CH_3, ^{t}Bu).

HRMS (ES+): calcd for $C_{60}H_{52}N_{15}O_7$ 1094.4174 $[M+H]^+$, found 1094.4312 $[M+H]^+$.

FT-IR (ATR): ν_{max} 3647, 3415, 2919, 1634, 1518, 1393, 1280, 1050, 1026, 832 and 765 cm^{-1}.

^{1}H-NMR (500 MHz, DMSO-d_6) compound 30 (APP, major).

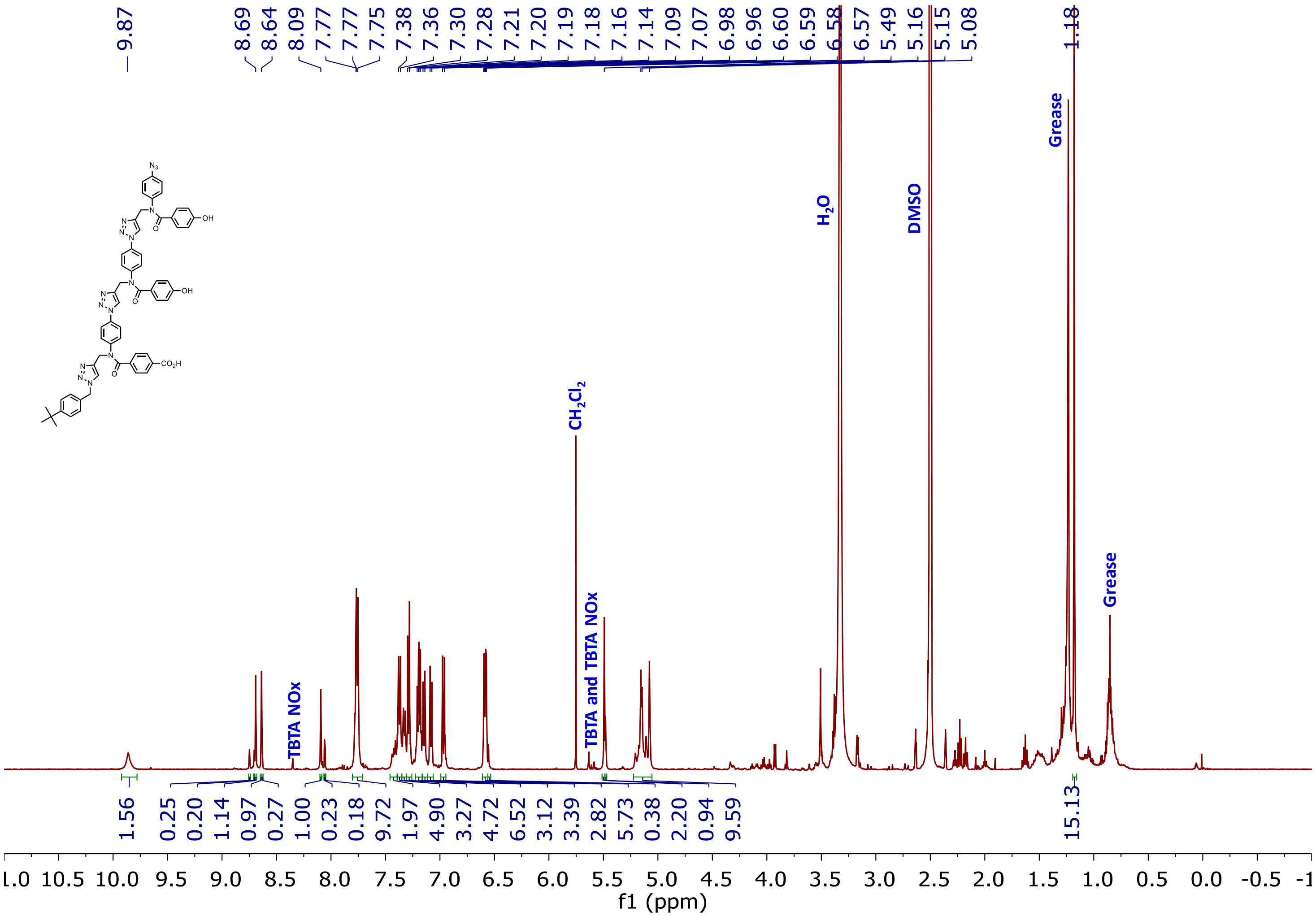

^{13}C-NMR (125MHz, DMSO-d_6) compound 30 (APP, major).

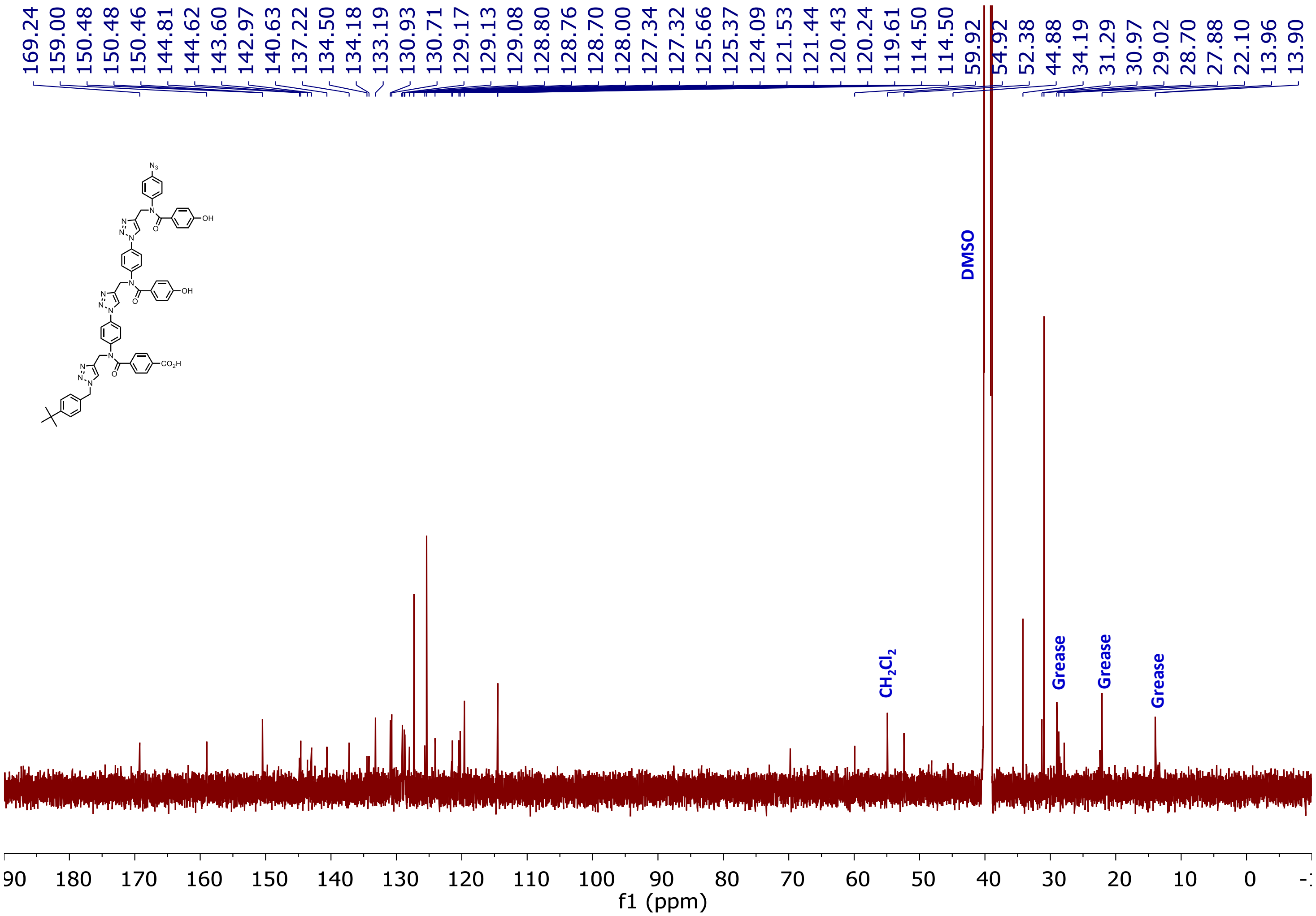

COSY (DMSO-d_6) compound 30 (APP, major).

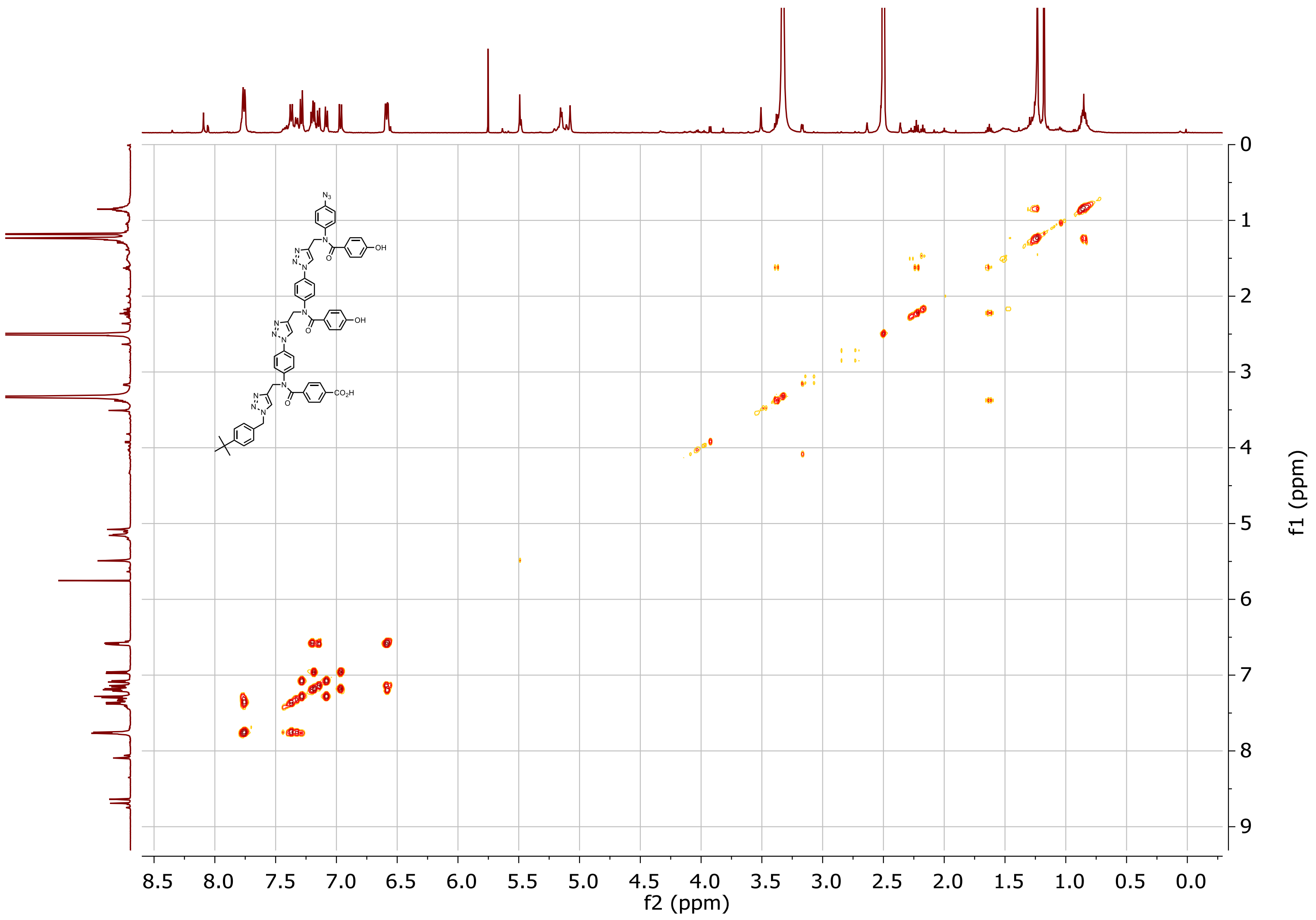

HSQC (DMSO-d_6) compound 30 (APP, major).

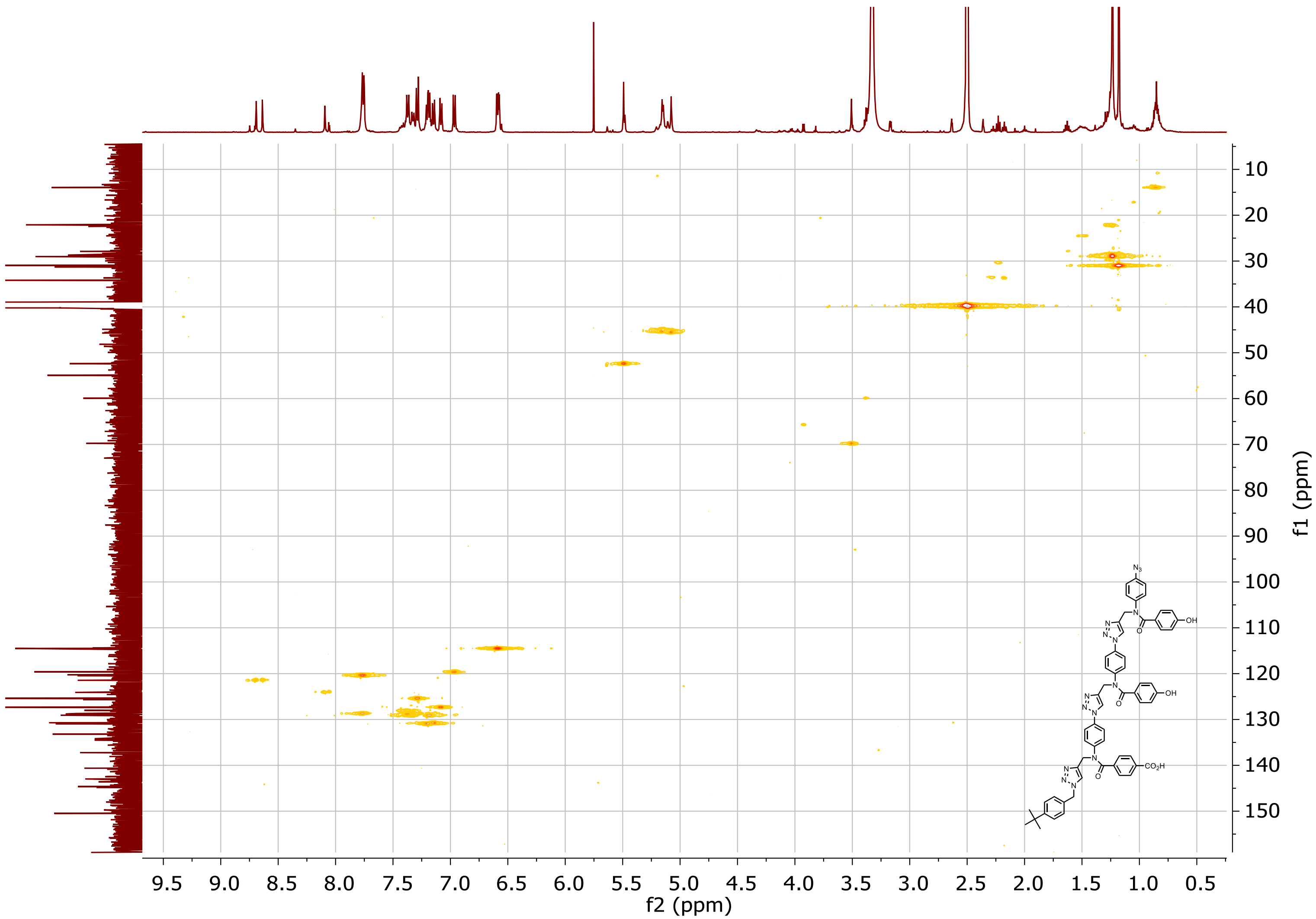

HMBC (DMSO-d_6) compound 30 (APP, major).

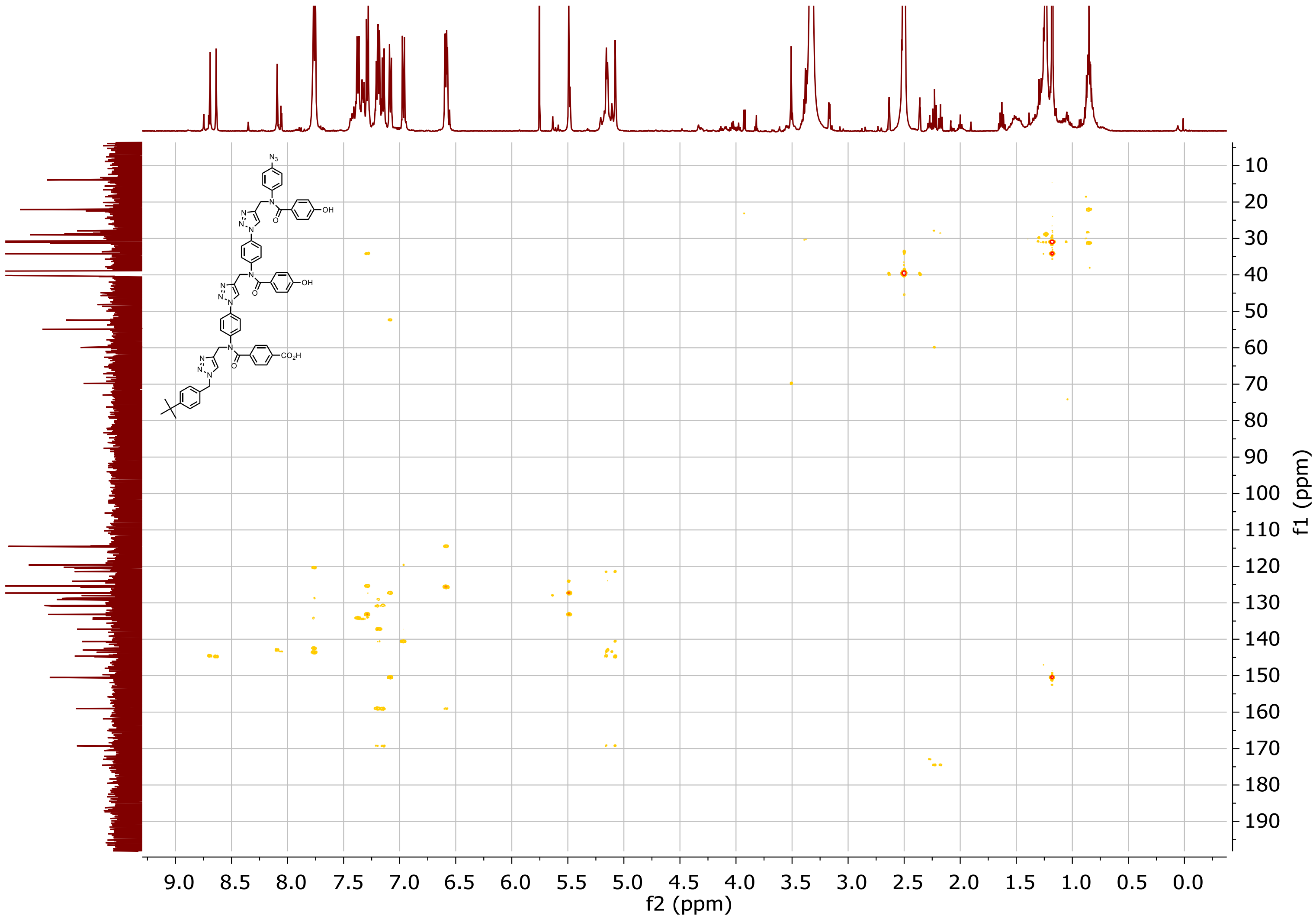

HRMS compound 30 (APP, major).

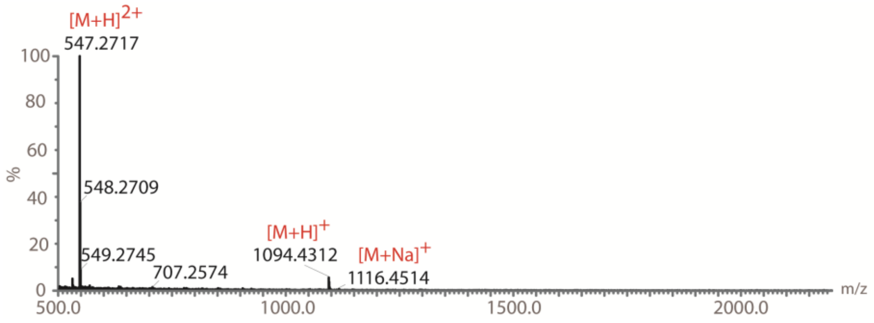

Step 7: Capping reaction with phenyl propargyl ether

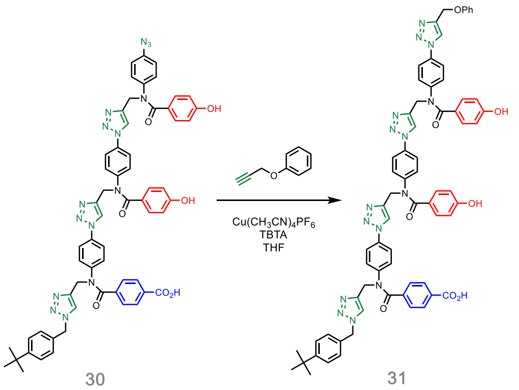

Scheme S19

Phenyl propargyl ether (3.5 uL, 0.03 mmol) was added to a solution of **30** (0.005 g, $4.6{\cdot}10^{-3}$ mmol) in dry and degassed THF (1 mL). $Cu(CH_3CN)_4PF_6$ (0.002, $5.5{\cdot}10^{-3}$ mmol) and TBTA (0.003 g, $5.5{\cdot}10^{-3}$ mmol) were added to the previous solution and the reaction stirred overnight at room temperature. Then, the reaction was diluted EtOAc and washed with 0.02M EDTA soln. (2x), 0.1M HCl soln. (1x), H_2O (1x) and brine. The organic layer was dried over $MgSO_4$ and concentrate under vacuum. The residue was purified by flash column chromatography on silica gel (gradient from 0% to 20% of MeOH in CH_2Cl_2) to afford **31** (0.005 g, 89%) as a white solid. Figure S15 shows the UPLC traces of the reaction crude and pure **31**, and mass spectrum for **31**. Figure S16 shows the 500 MHz ^{1}H NMR spectra of **31**, as a mixture of three different 3-mers: complementary APP 3-mer (72%, green), PAP 3-mer (17%, blue) and PPA 3-mer (11%, purple). Spectra of isolated APP, PAP and PPA sequences are shown for comparison.

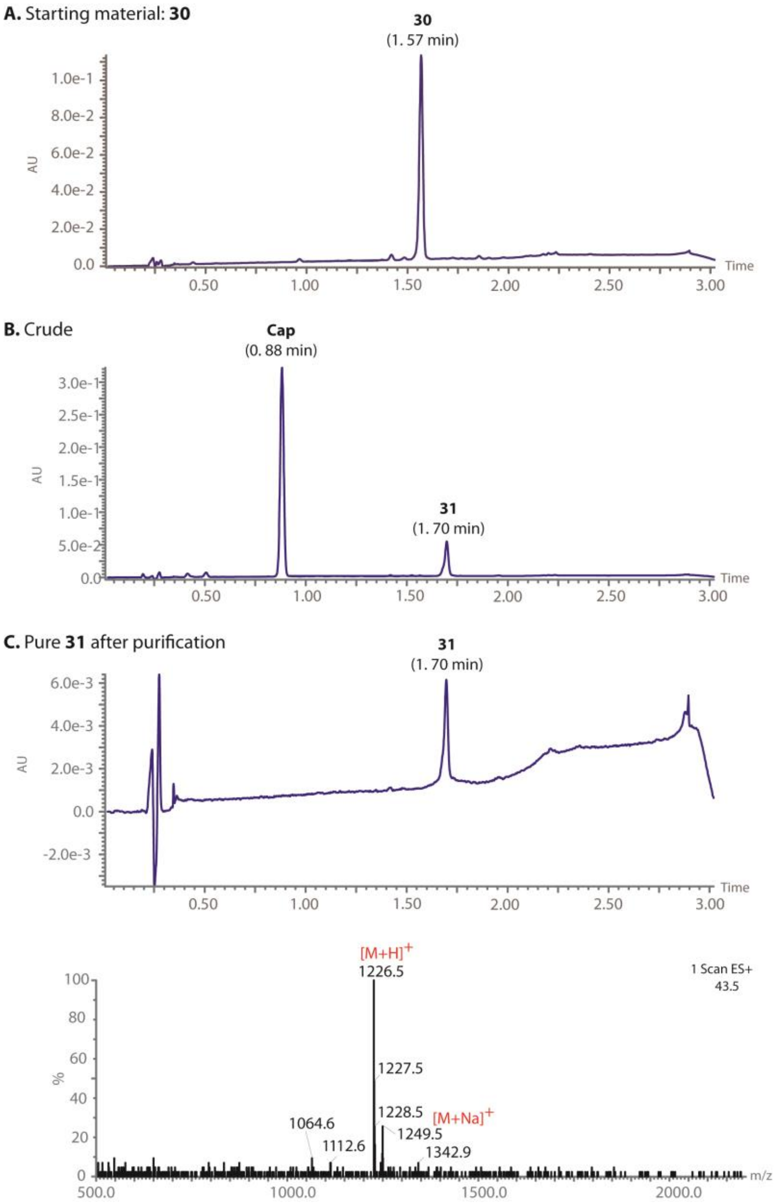

Figure S15. A. UPLC trace of the starting material (**30**)**. B.** UPLC trace of crude of step 7. **C.** ULPC trace and MS spectrum of complementary copy **31** (MW: 1225.5) as recovered after purification. *UPLC Conditions:* C18 column at 40 ^{o}C (254 nm) using water + 0.1% formic acid (A) and CH_3CN + 0.1% formic acid (B) with flow rate of 0.6 ml/min; Gradient of 0-2 minutes 35% -65% B + 1 minute 100% B.

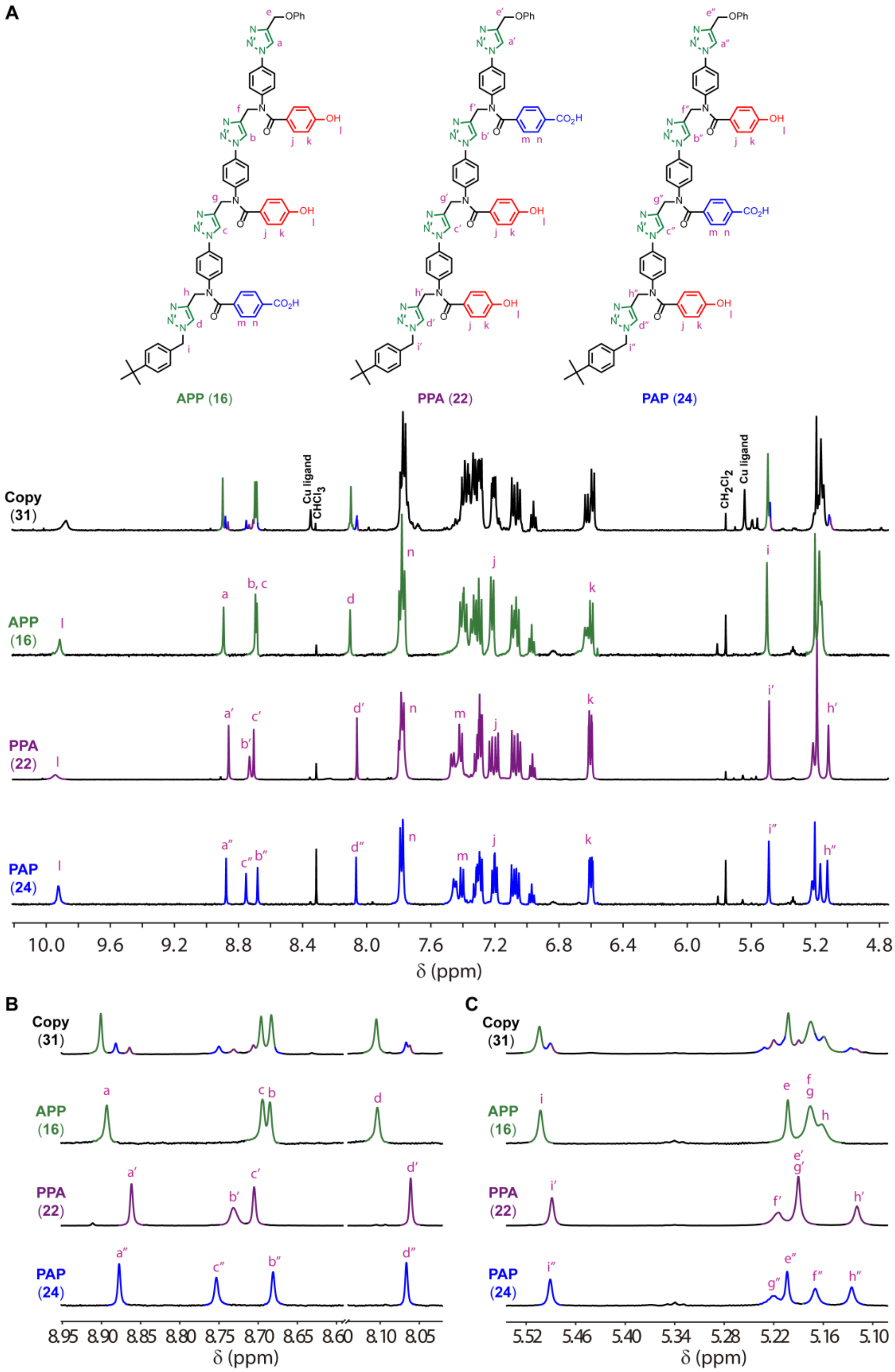

Figure S16. 500 MHz ^{1}H NMR spectra of copy **31** in DMSO-d_6 at 298 K (**A**): APP (green), PPA (purple) and PAP (blue) sequences are present (main protons assigned with letters). Spectra of isolated APP (**16**), PPA (**22**) and PAP (**24**) sequences are shown for comparison. Expansions of the triazole (**B**) and *N*-methylene regions (**C**).

^{1}H-NMR (500 MHz, DMSO-d_6) compound 31.

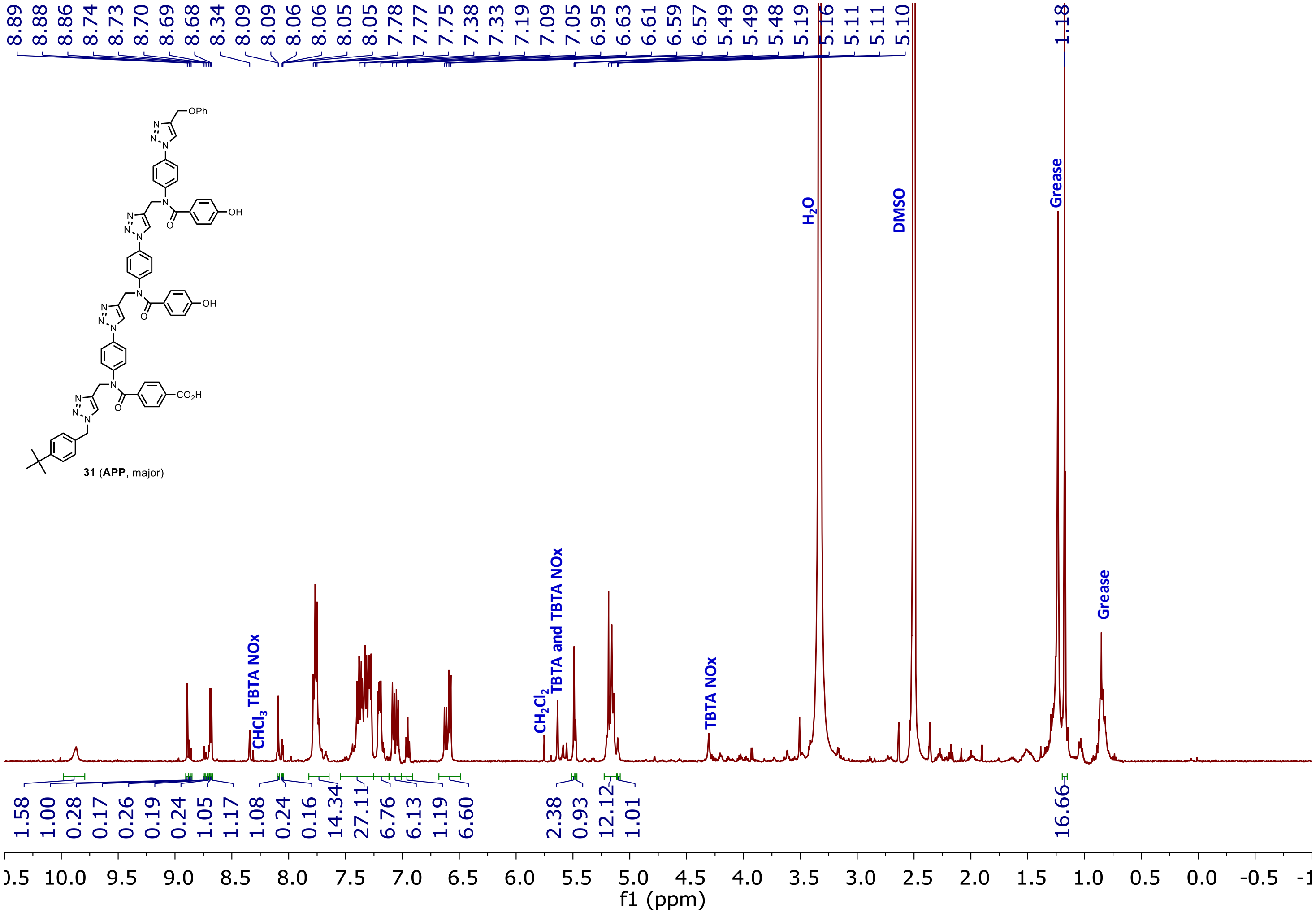

^{13}C-NMR (125MHz, DMSO-d_6) compound 31.

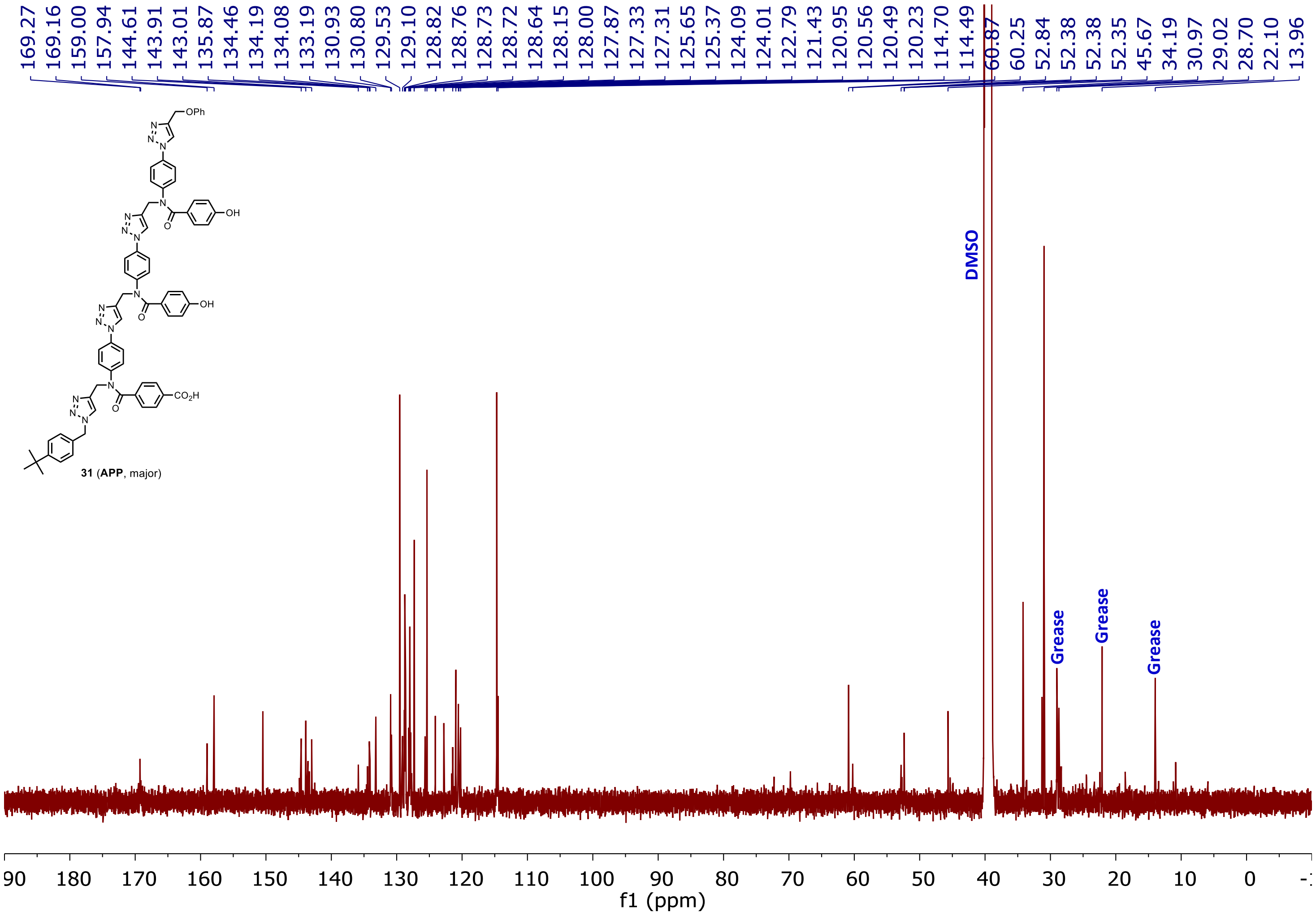

COSY (DMSO-d_6) compound 31.

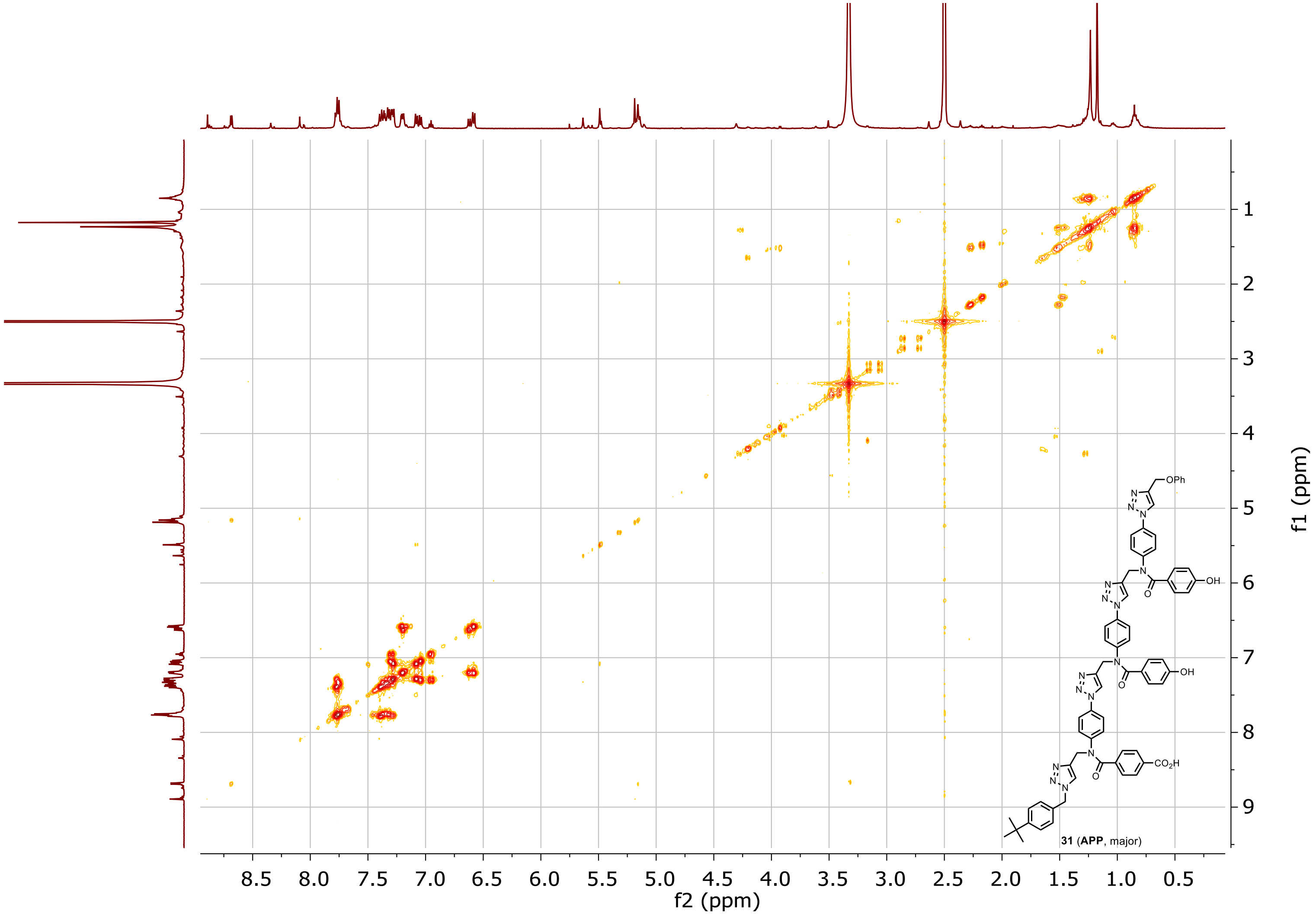

HSQC (DMSO-d_6) compound 31.

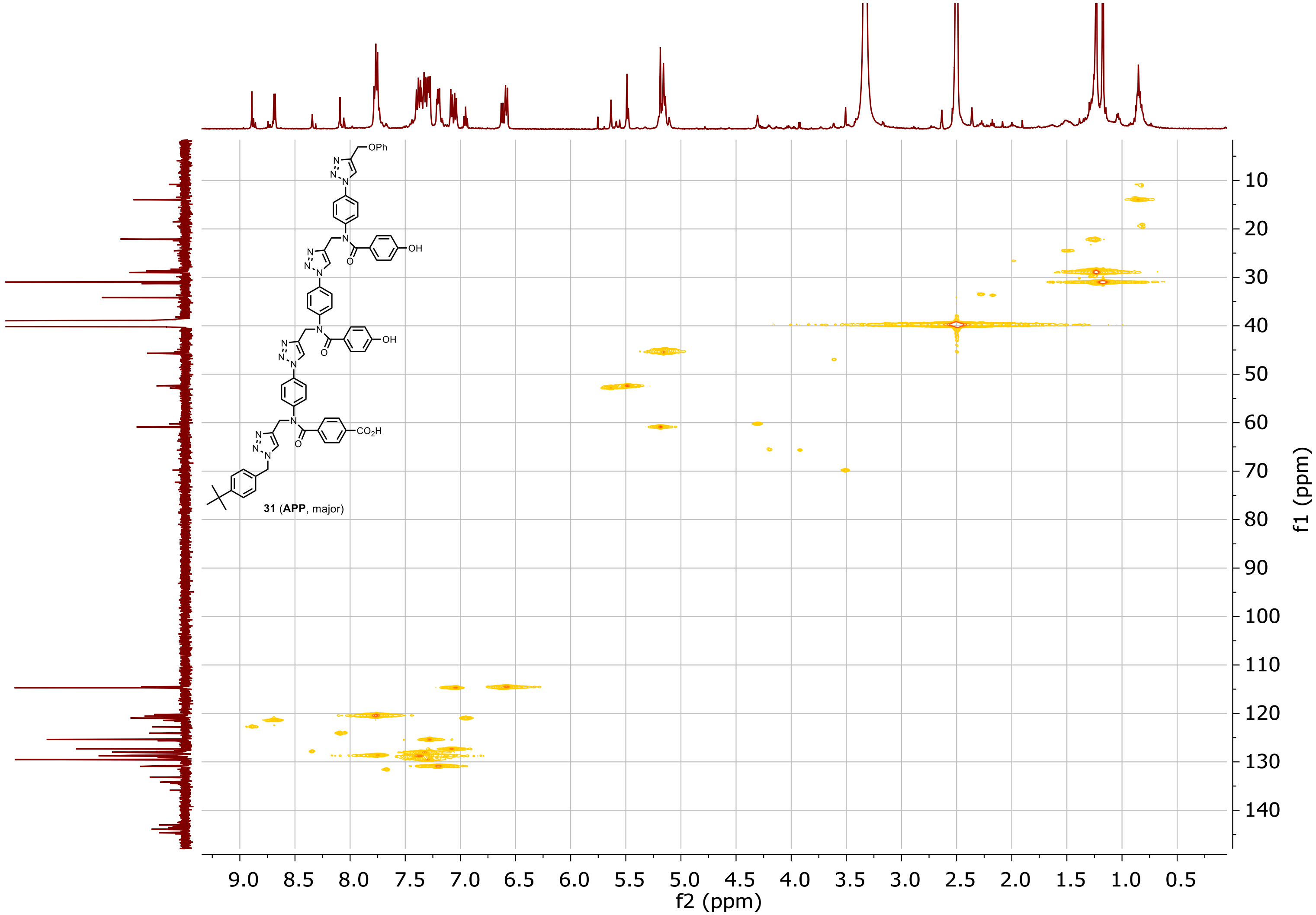

HMBC (DMSO-d_6) compound 31.

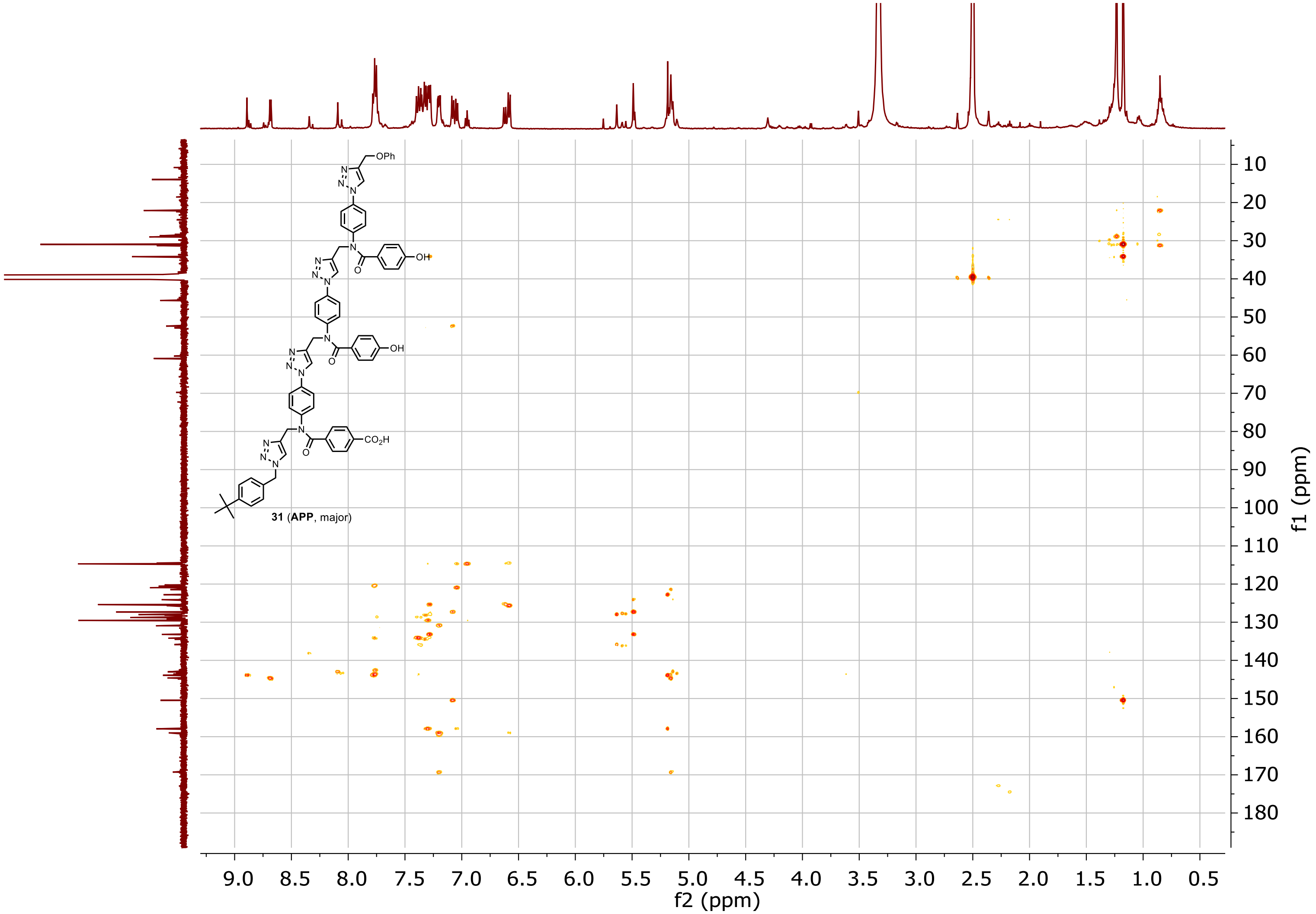

HRMS compound 31.

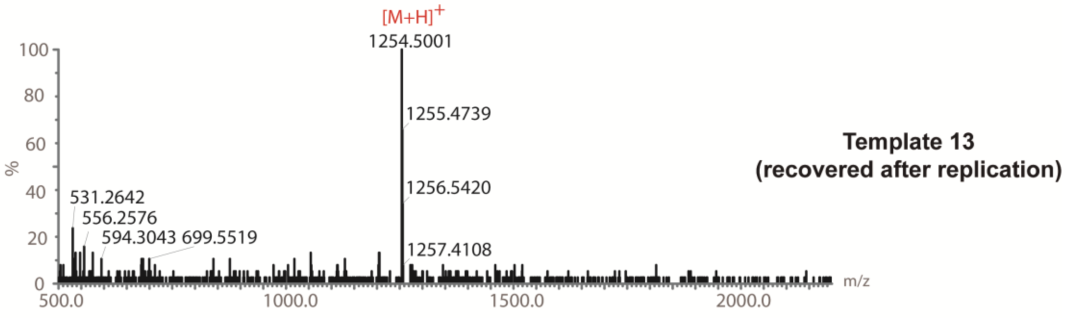

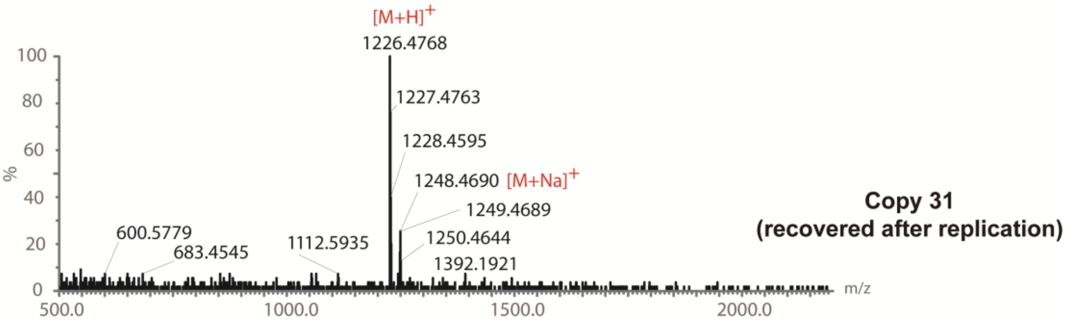

Molecular modelling calculations

General details

Molecular mechanics calculations were performed using MacroModel implemented in Maestro 11 (Schrodinger release 2016-4).[S3] Structures were simplified by replacing the capping groups by methyl and phenyl groups. The structures were minimized first and the minimized structures were then used as the starting molecular structure for the MacroModel conformational search. The conformational search was performed twice from a different starting conformation. The force field used was MMFFs with $CHCl_3$ solvation. The charges were defined by the force field library and no cut off was used for non-covalent interactions. A Polak-Ribiere Conjugate Gradient (PRCG) was used and each minimisation was subjected to 10,000 iterations with a convergence threshold of 0.01. Conformational searches were performed using 100 steps per rotatable bond (maximum number of steps of 10,000). Images were created using PyMol.[S4]

Parallel and antiparallel trimer duplexes

A

B

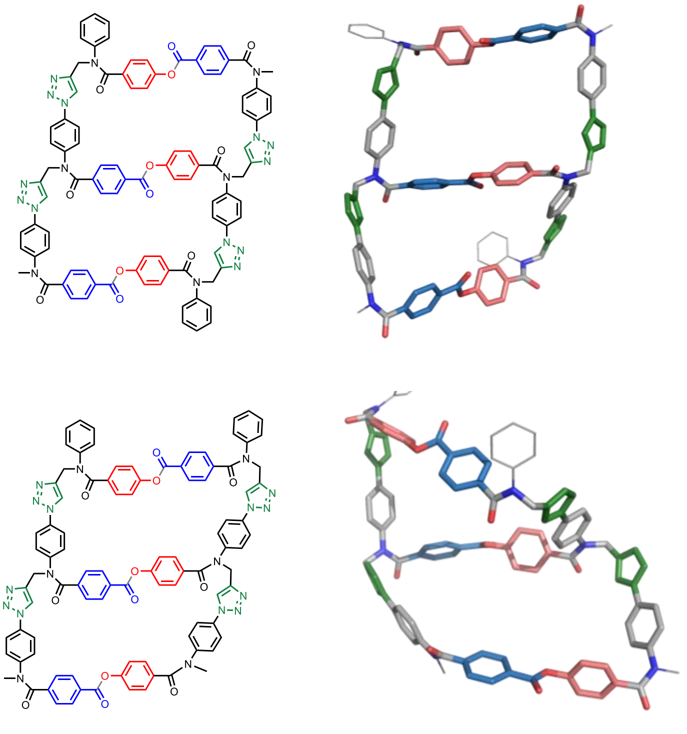

Figure S17. Lowest energy conformation from a conformational search of antiparallel AAP•APP (**A**, 1940.2 kJ·mol^{-1}) and parallel AAP•PPA (**B**, 1944.7 kJ·mol^{-1}) duplexes using molecular mechanics (MMFFs force-field implemented in Macromodel with $CHCl_3$ solvation).[S3,S4]

Estimation of the ring strain for the ZIP reaction

The ring strain for the ZIP reaction was estimated by using the strain energy of the product duplexes (E_{strain}). The disconnection of the triazole rings into the corresponding azide and alkyne was not used due to problems in the parameterisation of azide moieties in the

MMFFs force-field. The method used for disconnecting the macrocyclic structures of the duplexes through the phenyl-triazole bonds is illustrated in Figure S18. This hypothetical transformation provides a method for calculating E_{Bond} as the energy difference between two identical fragments and the connected oligomer backbone (Equation 1).

$$E_{Bond} = E_{product} - (2 \times E_{fragment}) = 467.1 - (2 \times 187.1) = \mathbf{92.9\ kJ \cdot mol^{-1}} \qquad \text{(Eq.1)}$$

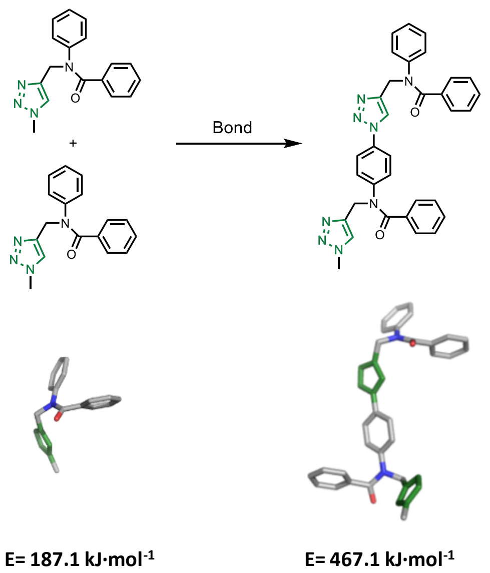

Figure S18. Model system used to calculate bond connection energy E_{Bond} and lowest energy conformations from conformational searches using molecular mechanics (MMFFs force-field implemented in Macromodel with $CHCl_3$ solvation).[S3,S4]

The corresponding disconnection for the antiparallel and parallel dimer duplexes is illustrated in Figure S19. The energy contribution associated with phenyl-triazole bond connection (E_{bond}) was subtracted from the difference between the energy of the product duplex (E_{duplex}) and the energy of the pre-ZIP intermediate (E_{preZIP}) (Equation 2).

$$E_{strain} = E_{duplex} - E_{preZIP} - n\ E_{bond} \qquad \text{(Eq.2)}$$

where *n* is the number of triazole rings formed in the ZIP reaction.

The resulting ring strain is:

Antiparallel: $E_{strain} = E_{ZIP} - E_{preZIP} - E_{bond} = 1217.0 - 1106.5 - 92.9 = \mathbf{17.6\ kJ \cdot mol^{-1}}$

Parallel: $E_{strain} = E_{ZIP} - E_{preZIP} - E_{bond} = 1222.1 - 1106.5 - 92.9 = \mathbf{22.7\ kJ \cdot mol^{-1}}$

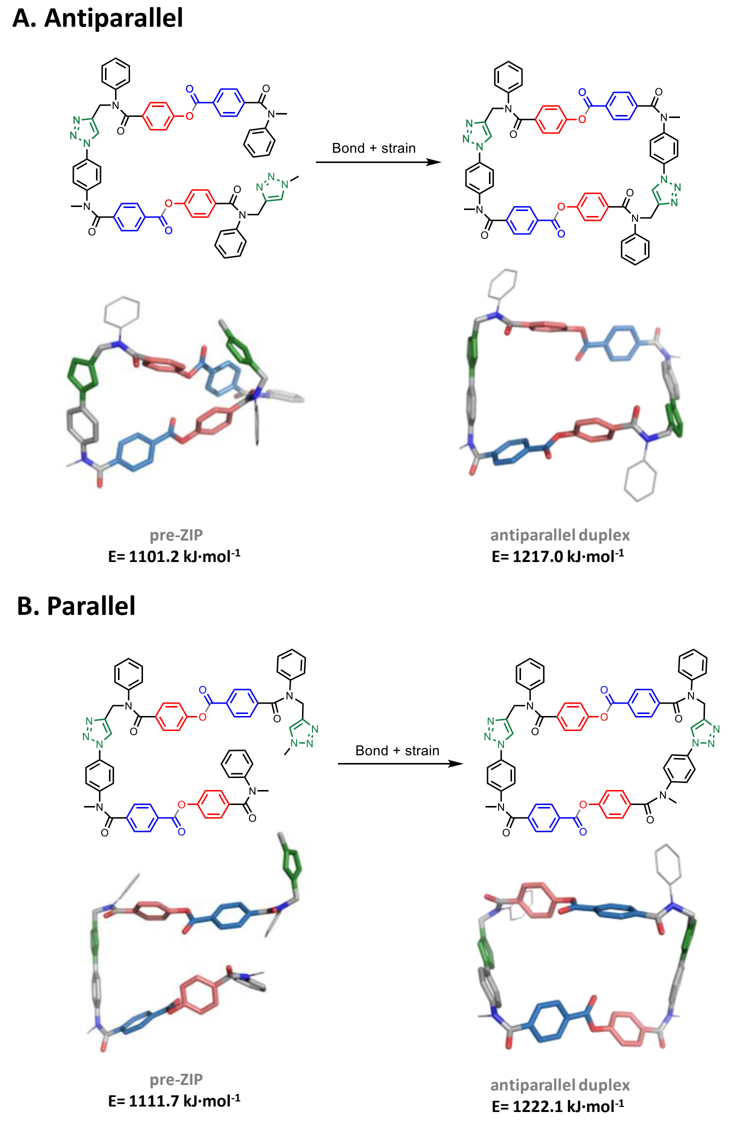

Figure S19. Calculation of ring strain for the antiparallel (A) and parallel (B) dimeric duplexes, showing the lowest energy conformations from conformational searches using molecular mechanics (MMFFs force-field implemented in Macromodel with $CHCl_3$ solvation).[S3,S4]

The ring strain for the antiparallel and parallel trimer duplexes was calculated in the same way using the disconnections illustrated in Figure S20.

Antiparallel: E_{strain} = 1940.2 – 1689.2 – (2x92.9) = **65.3 kJ·mol^{-1}** (32.7 kJ·mol^{-1} per ring).

Parallel: E_{strain} = 1944.7 – 1689.2 – (2x92.9) =**69.8 kJ·mol^{-1}** (34.9 kJ·mol^{-1} per ring)

The calculated ring strain associated with macrocyclisation in the ZIP step is 18-35 kJ·mol^{-1} per ring, which is in the order of the ring strain of common 5- and 6-membered rings.[S5-S8] These results suggest that the ring strain associated with macrocyclisation in the ZIP step is small using this backbone.

A. Antiparallel

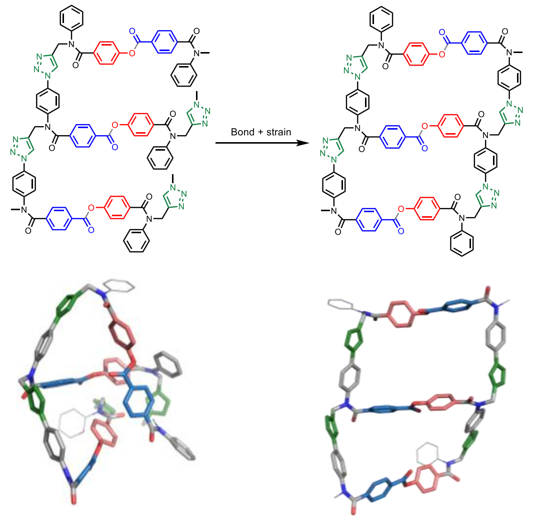

pre-ZIP
E= 1687.4 kJ·mol^{-1}

antiparallel duplex
E= 1940.2 kJ·mol^{-1}

B. Parallel

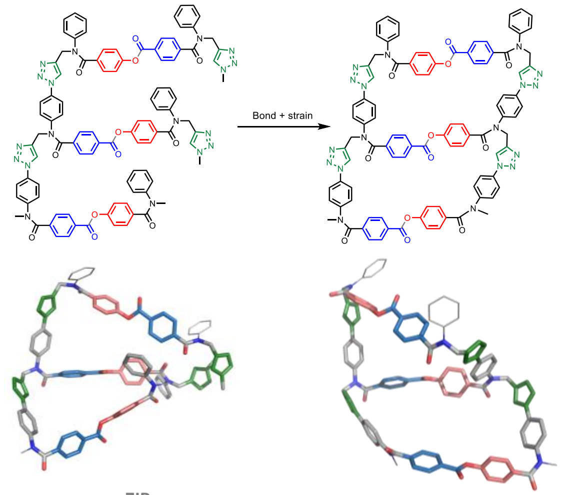

pre-ZIP
E= 1690.9 kJ·mol^{-1}

antiparallel duplex
E= 1944.7 kJ·mol^{-1}

Figure S20. Calculation of ring strain for the antiparallel (A) and parallel (B) trimer duplexes showing the lowest energy conformations from conformational searches using molecular mechanics (MMFFs force-field implemented in Macromodel with $CHCl_3$ solvation).[S3,S4]

References

[S1] W. Li, T. Tian, W. Zhu, J. Cui, Y. Ju and G. Li. *Polym. Chem.* **2013**, *4*, 3057.

[S2] Sequences are written from the t-butyl benzyl terminus using a 2-letter code: A for carboxylic acid and P for phenol.

[S3] Schrödinger Release 2016-4: MacroModel, Schrödinger, LLC, New York, NY, 2016.

[S4] The PyMOL Molecular Graphics System, Version 2.0 Schrödinger, LLC.

[S5] T. Dudev and C. Lim. *J. Am. Chem. Soc.* **1998**, *120*, 4450.

[S6] W. Saiyasombat, R. Molloy, T. M. Nicholson, A. F. Johnson, I. M. Ward and S. Poshyachinda. *Polymer*, **1998**, *39*, 5581.

[S7] P. R. Khoury, J. D. Goddard and W. Tam. *Tetrahedron* **2004**, *60*, 8103.

[S8] A Hejl, O. A. Scherman and R. H. Grubbs. *Macromolecules* **2005**, *38*, 7214.
